# Synthesis of New Functionally Substituted 9-Azabicyclo[4.2.1]nona-2,4,7-trienes by Cobalt(I)-Catalyzed [6π + 2π]-Cycloaddition of *N*-Carbocholesteroxyazepine to Alkynes

**DOI:** 10.3390/molecules26102932

**Published:** 2021-05-14

**Authors:** Gulnara N. Kadikova, Vladimir A. D’yakonov, Usein M. Dzhemilev

**Affiliations:** Laboratory of Catalytic Synthesis, Institute of Petrochemistry and Catalysis, Russian Academy of Sciences, 450075 Ufa, Russia; Kad.Gulnara@gmail.com (G.N.K.); Dzhemilev@anrb.ru (U.M.D.)

**Keywords:** cycloaddition, *N*-carbocholesteroxyazepine, alkynes, 9-azabicyclo[4.2.1]nona-2,4,7-trienes, cobalt(II) acetylacetonate

## Abstract

Catalytic [6π + 2π]-cycloaddition of *N*-carbocholesteroxyazepine with functionally substituted terminal alkynes and 1,4-butynediol was performed for the first time under the action of the Co(acac)_2_(dppe)/Zn/ZnI_2_ three-component catalytic system. The reaction gave previously undescribed but promising 9-azabicyclo[4.2.1]nona-2,4,7-trienes (in 79–95% yields), covalently bound to a natural metabolite, cholesterol. The structure of the synthesized azabicycles was confirmed by analysis of one- and two-dimensional (^1^H, ^13^C, DEPT ^13^C, COSY, NOESY, HSQC, HMBC) NMR spectra.

## 1. Introduction

**Figure 1:**
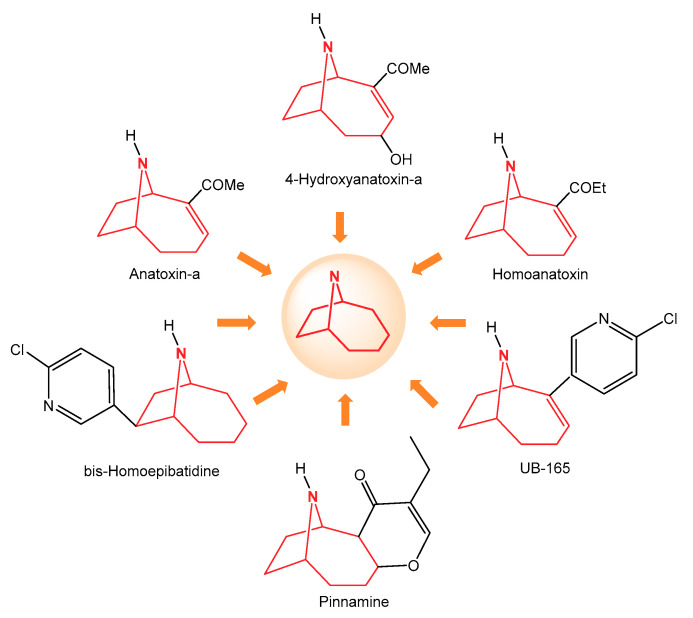
Some biologically active azacycles containing the 9-azabicyclo[4.2.1]nonane skeleton.

Although some 9-azabicyclo[4.2.1]nonane derivatives were described for the first time back in the 1970s [1,2,3,4,5], they are still attracting the attention of synthetic chemists [6,7,8,9,10,11,12,13,14,15], largely related to their pronounced biological activity and high pharmacological potential [14]. The 9-azabicyclo[4.2.1]nonane cage is a key structural component of several important natural and synthetic alkaloids (anatoxin-a [6,7,8,9,10,11,12,13,14], pinnamine [13,16,17], bis-homoepibatidine [18,19], and UB-165 [20,21,22,23,24,25,26,27]), possessing properties of nicotinic acetylcholine receptor agonists in the central and vegetative nervous systems (Figure 1). Therefore, various analogues containing the 9-azabicyclo[4.2.1]nonane cage are actively being studied by pharmaceutical scientists as potential medicinal agents for the treatment of severe neurological disorders such as Parkinson’s and Alzheimer’s diseases, schizophrenia, and depression [21,22,23,24,25,26,27,28,29].

**Scheme 1:**
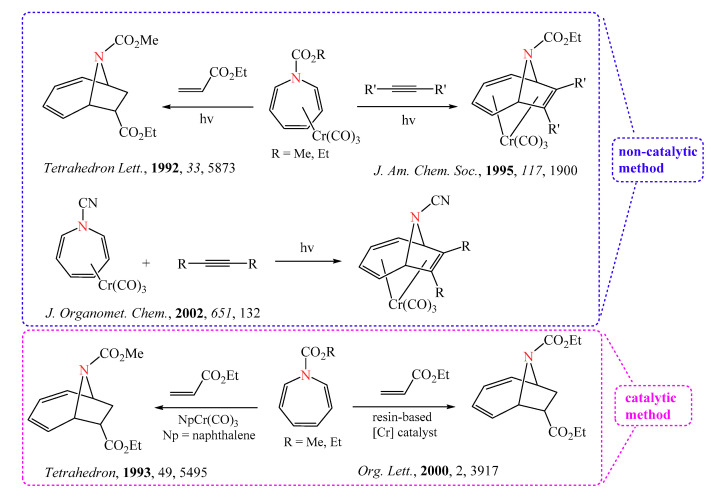
Chromium(0)-promoted and chromium(0)-catalyzed [6π + 2π] cycloadditions of *N*-substituted azepines in the synthesis of 9-azabicyclo[4.2.1]nonadi(tri)enes.

According to previously published data, an efficient method for the synthesis of 9-azabicyclo[4.2.1]nonane cages is based on the cycloaddition reactions of *N*-substituted azepines catalyzed by transition metal complexes [30]. However, these reactions have been studied rather superficially, being addressed in a few publications on the photoinduced cyclo-codimerization of tricarbonyl(η^6^-*N*-carboalkoxyazepine)chromium(0) [31,32,33,34,35,36] and tricarbonyl(η^6^-*N*-cyanoazepine)chromium(0) [37] with alkenes and alkynes. Meanwhile, data on catalytic versions of these reactions are scarcely reported in the literature, except for two examples of Cr(0)-catalyzed cycloaddition of *N*-carbomethoxyazepine [34] and *N*-carbethoxyazepine [38] to ethyl acrylate (Scheme 1). Hence, the catalytic cycloaddition of *N*-substituted azepines is an alternative approach to the synthesis of 9-azabicyclo[4.2.1]nonanes, and therefore, these reactions require further thorough investigation.

**Scheme 2:**
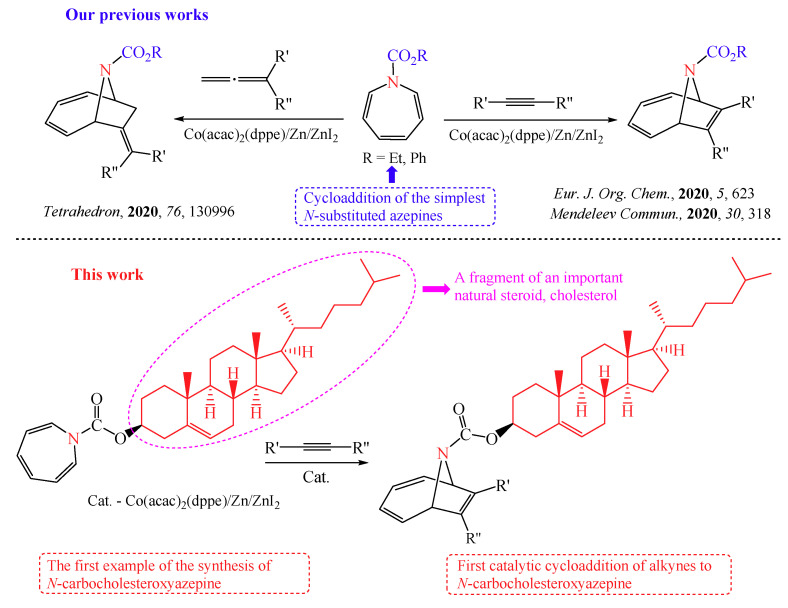
Schematic view of the goals of our investigation in comparison with previously published data.

We previously reported [39,40,41] the development of an efficient one-pot synthesis of some substituted 9-azabicyclo[4.2.1]nona-2,4,7-trienes and 9-azabicyclo[4.2.1]nona-2,4-dienes based on the cobalt(I)-catalyzed cycloaddition of *N*-carbethoxy(phenoxy)azepines to alkynes, 1,3-diynes, and 1,2-dienes (Scheme 2).

In order to further develop the above promising trend towards new 9-azabicyclo[4.2.1]nonanes, and in view of the high relevance of the development of biologically active substances for the synthesis of new-generation pharmaceutical agents, we set ourselves the task of preparing 9-azabicyclo[4.2.1]nona-2,4,7-trienes containing a natural compound fragment in their molecules. It is well known that half of the currently existing medicinal drugs have been, and continue to be, developed on the basis of natural compounds’ skeletons and their numerous synthetic analogues. As the natural compound for the present work, we chose cholesterol, which performs very important functions in the human body [42,43,44,45,46,47,48,49,50,51]. Cholesterol is a structural component of cell membranes and provides their stability, participates in the biosynthesis of steroid sex hormones and corticosteroids, serves as a basis for the formation of bile acids and vitamin D, and also protects red blood cells from the action of hemolytic poisons. Thus, to our knowledge, the present study is the first to report on the catalytic [6π + 2π]-cycloaddition of *N*-carbocholesteroxyazepine to alkynes in order to access new 9-azabicyclo[4.2.1]nona-2,4,7-trienes containing, additionally, cholesterol building blocks (Scheme 2). To this end, we emphasize here the novelty of our planned investigation, since we succeeded in preparing, for the first time, an *N*-carbocholesteroxyazepine system.

## 2. Results

**Scheme 3:**
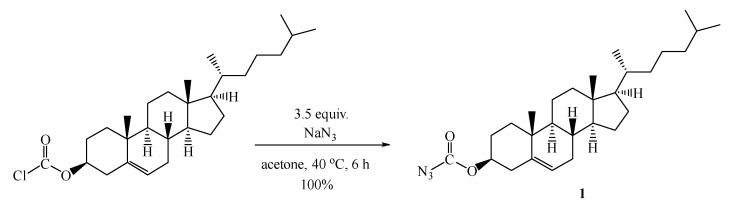
Synthesis of cholesteryl azidoformate.

Keeping this task in mind, we set the goal to prepare the starting monomer, *N*-carbocholesteroxyazepine. First, we carried out the reaction of commercial cholesteryl chloroformate with sodium azide, providing, in quantitative yield, cholesteryl azidoformate **1** in the conditions depicted in Scheme 3. Please see the Supplementary Figures S1–S6.

**Scheme 4:**
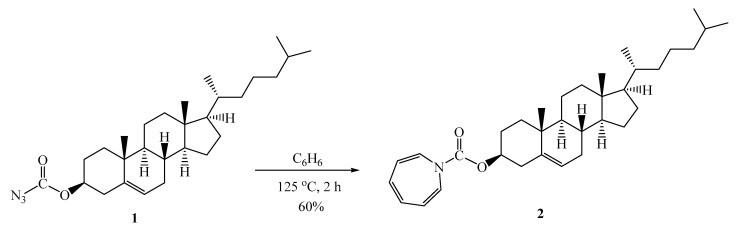
Synthesis of *N*-carbocholesteroxyazepine.

Next, thermolysis of cholesteryl azidoformate **1** in benzene at 125 °С (in an autoclave) gave the target *N*-carbocholesteroxyazepine **2** with a yield of 60% (Scheme 4). Please see the Supplementary Figures S7–S12.

With *N*-carbocholesteroxyazepine **2** in our hands, we investigated its cycloaddition to the terminal alkynes **3a–t**. Thus, we found that the desired [6π + 2π]-cycloaddition process occurred, being catalyzed by the Co(acac)_2_(dppe)/Zn/ZnI_2_ (dppe-1,2-bis(diphenylphosphino)ethane) system [52,53,54,55,56,57] under developed conditions (10 mol% Co(acac)_2_(dppe), 30 mol% Zn, and 20 mol% ZnI_2_, in DCE (1,2-dichloroethane) as solvent, for 20 h at 60 °C) to afford substituted 9-azabicyclo[4.2.1]nona-2,4,7-trienes **4a–t** with 79–95% yields (Scheme 5). The adducts were formed as two N-(CO)O-cholesteryl rotamers [33,34,39,40,41] in a 1:1 ratio, arising due to hindered rotation of the substituent around the CN bond. Please see the Supplementary Figures S13–S112.

**Figure 2:**
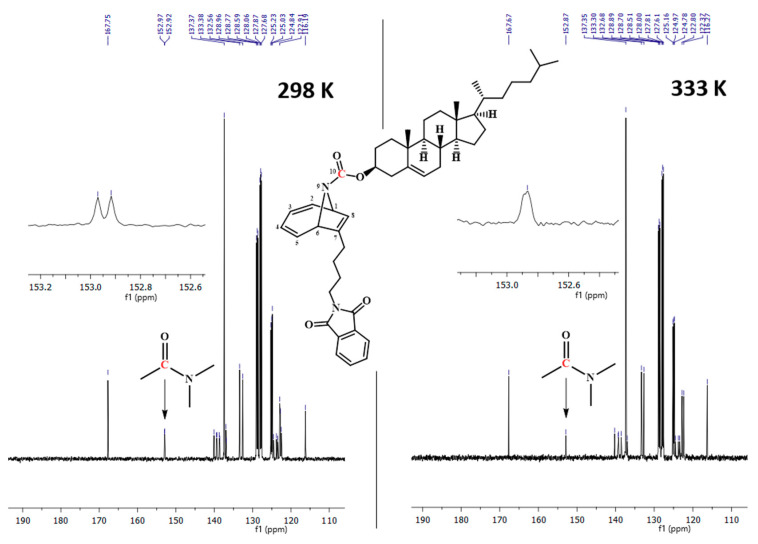
Fragments of temperature-dependent ^13^С NMR spectra of **4r** in C_7_D_8_.

It is well known that at elevated temperatures, the transition from one rotamer to another is accelerated. Therefore, we studied the exchange process between rotamers upon heating and calculated the energy barrier at an operating temperature of 333 K. The investigation of the temperature dependence of the NMR spectra of compound **4r** in C_7_D_8_ at 333 K has shown the presence of coalescence of a number of characteristic signals in the ^13^C NMR spectrum—for example, the signal of the carbamide carbon atom C(10) (Figure 2). In this case, at room temperature, double signals of the carbamide carbon atom C(10) are observed with a difference of 0.05 ppm (*δ*) or 25 Hz in accordance with the frequency scale. The value of the energy barrier at 333 K (*T*_coal._), calculated using the approximate formula or the Eyring equation (1) [58], was about 17 kcal/mol, which corresponds to the values of the barriers to hindered rotation around the amide bond. Please see the Supplementary Figures S118–S121.

∆*G*^‡^ = 19.14*T*_coаl._ (9.97 + log *T*_coal._/*δ*ν) (1)

Our experiments clearly demonstrated the Co(acac)_2_(dppe)/Zn/ZnI_2_ three-component catalytic system [52,53,54,55,56,57] being not only tolerant but equally efficient for a large variety of the substituents (alkyl, phenyl, *p-*halophenyl, alcohol, nitrile, ester, sulfide, phthalimide, cycloalkane, naphthalene, and phenanthrene) in the starting alkynes.

**Scheme 6:**
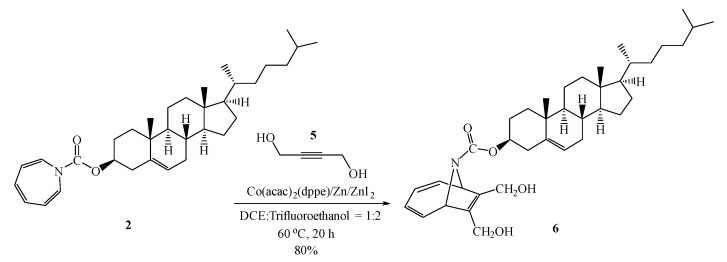
Cycloaddition of *N*-carbocholesteroxyazepine to 1,4-butynediol.

In identical conditions, *N*-carbocholesteroxyazepine **2** reacted as well with symmetrical disubstituted 1,4-butynediol **5** to give the [6π + 2π]-cycloadduct, 9-azabicyclo[4.2.1]nona-2,4,7-triene **6** (80% yield) as a 1:1 mixture of two N-(CO)O-cholesteryl-rotamers (Scheme 6). Please see the Supplementary Figures S113–S117.

## 3. Materials and Methods

### 3.1. General Procedures

Briefly, ^1^Н, ^13^С spectra were measured in CDCl_3_ on a Bruker Avance-500 spectrometer (500 MHz for ^1^H; 125 MHz for ^13^C). High-resolution mass spectra (HRMS) were measured on an instrument (MaXis impact, Bruker Daltonik GmbH, Bremen, Germany) using a time-of-flight mass analyzer (TOF) with electrospray ionization (ESI). In experiments on selective collisional activation, the activation energy was set at the maximum abundance of fragment peaks. A syringe injection was used for solutions in MeCN (flow rate: 5 µL/min). Nitrogen was applied as a dry gas; the interface temperature was set at 180 °C. All solvents were dried and freshly distilled before use. All reactions were carried out under a dry argon atmosphere. Cholesteryl chloroformate, sodium azide, the terminal alkynes, alkynols, and ZnI_2_ were purchased from commercial sources and used without further purification. Co(acac)_2_(dppe), ethyl pent-4-ynoate, 5-bromopent-1-yne, and sulfanylalkynes were synthesized according to procedures described in the literature [59,60,61]. For column chromatography, silica gel from Acros Organics (Thermo Fisher Scientific, Geel, Belgium) (0.060–0.200 mm) was used.

### 3.2. Synthesis of Cholesteryl Azidoformate

A mixture of cholesteryl chloroformate (2.25 g, 5 mmol) and sodium azide (1.14 g, 17.5 mmol) in dry acetone (97 mL) was heated at 40 °С for 6 h with vigorous stirring. After this period, the reaction mixture was left to reach room temperature, when minerals were filtered off. The organic filtrate was concentrated under reduced pressure to dryness to provide crude сholesteryl azidoformate 1 (2.278 g, 100% yield with respect to cholesteryl chloroformate) as a white solid. This material was used as is in the next experiments without further purification.

### 3.3. Synthesis of N-Carbocholesteroxyazepine

A solution of cholesteryl azidoformate **1** (2.28 g, 5 mmol) in dry benzene (106 mL) was heated in an autoclave at 125 °C for 2 h with stirring, under autogenous pressure. After this period, the cooled reaction solution was stripped of benzene under reduced pressure. Chromatographic purification over silica gel (petroleum ether/ethyl acetate 20:1) afforded the target product **2** (1.517 g, 60% yield with respect to cholesteryl azidoformate) as a yellow solid.

### 3.4. Cycloaddition of N-Carbocholesteroxyazepine to Alkynes

Zinc powder (0.020 g, 0.3 mmol) was added to a solution of Co(acac)_2_(dppe) (0.066 g, 0.1 mmol) in DCE (1.5 mL) for **3a–f,h,j–l,n–p,r–t** (in 1 mL DCE for **3g,i,m,q,5**) in a Schlenk tube under a dry argon atmosphere, and the mixture was stirred at room temperature for 2 min. Next, *N*-carbocholesteroxyazepine (0.505 g, 1.0 mmol), the alkyne (1.5 mmol) in DCE (1.5 mL) for **3a–f,h,j–l,n–p,r–t** (in 2 mL trifluoroethanol for **3g,i,m,q,5**), and dry ZnI_2_ (0.064 g, 0.2 mmol) were added successively. After heating at 60 °C for 20 h, the reaction was stopped by the addition of petroleum ether and stirring in air for 10 min to deactivate the catalyst. After filtration through a short pad of silica, the volatiles were removed under vacuum. Chromatographic purification over silica gel (petroleum ether/ethyl acetate 5:1 as eluent for **4a–p,s,t,6**; petroleum ether/ethyl acetate 2:1 for **4q,r**) afforded the target products **4a–t, 6**.

### 3.5. Characterization of the Products

*Сholesterylazidoformate* (**1**)**:** Yield 100% (2.278 g), white solid, m. p. = 96–97 °С, [α]_D_^17^—30.4 (*c* 0.48, CHCl_3_). ^1^Н NMR (500 MHz, CDCl_3_): *δ*_H_ 5.42 (d, J = 3.5 Hz, 1Н), 4.57–4.66 (m, 1Н), 2.35–2.47 (m, 2Н), 1.80–2.08 (m, 5Н), 1.24–1.73 (m, 11Н), 1.07–1.23 (m, 7Н), 0.99–1.06 (m, 5Н), 0.93 (d, J = 6.4 Hz, 4Н), 0.88 (d, J = 6.3 Hz, 6Н), 0.70 (s, 3Н) ppm. ^13^С NMR (125 MHz, CDCl_3_): *δ*_C_ 156.9, 138.9, 123.4, 78.8, 56.7, 56.2, 50.0, 42.3, 39.7, 39.5, 37.8, 36.8, 36.5, 36.2, 35.8, 31.9, 31.8, 28.2, 28.0, 27.5, 24.3, 23.9, 22.8, 22.6, 21.1, 19.3, 18.7, 11.9 ppm. HRMS (ESI-TOF): calcd. for C_28_H_45_N_3_O_2_Na [M + Na]^+^ 478.3409, found 478.3416.

*N-Carbocholesteroxyazepine* (**2**): Yield 60% (1.517 g), yellow solid, m. p. = 124–125 °С, [α]_D_^23^—13.9 (*c* 0.50, CHCl_3_), *R*_f_ = 0.40 (petroleum ether/ethyl acetate 20:1). ^1^Н NMR (500 MHz, CDCl_3_): *δ***_H_** 6.07 (s, 2Н), 5.91 (s, 1Н), 5.84 (s, 1H), 5.55 (s, 1Н), 5.47 (s, 1Н), 5.40 (s, 1Н), 4.59–4.68 (m, 1Н), 2.33–2.47 (m, 2Н), 1.92–2.08 (m, 3Н), 1.80–1.91 (m, 2Н), 1.23–1.69 (m, 11Н), 1.08–1.22 (m, 7Н), 0.99–1.07 (m, 5Н), 0.93 (d, J = 6.3 Hz, 4Н), 0.88 (d, J = 6.5 Hz, 6Н), 0.69 (s, 3Н) ppm. ^13^С NMR (125 MHz, CDCl_3_): *δ*_C_ 153.0, 139.6, 131.0 (2C), 130.6 (2C), 122.8, 119.4, 119.0, 75.9, 56.7, 56.1, 50.0, 42.3, 39.7, 39.5, 38.4, 37.0, 36.6, 36.2, 35.8, 31.9, 31.87, 28.2, 28.1, 28.0, 24.3, 23.8, 22.8, 22.6, 21.1, 19.4, 18.7, 11.9 ppm. HRMS (ESI-TOF): calcd. for C_34_H_51_NO_2_Na [M + Na]^+^ 528.3817, found 528.3824.

Сholesteryl (1S*,6R*)-7-butyl-9-azabicyclo[4.2.1]nona-2,4,7-triene-9-carboxylate equivalent with Сholesteryl (1R*,6S*)-7-butyl-9-azabicyclo[4.2.1]nona-2,4,7-triene-9-carboxylate (**4a**): Yield 91% (0.535 g), yellowish solid, m. p. = 94–95 °С, [α]_D_^17^—17.6 (c 0.49, CHCl_3_), exists as two N-(CO)O-cholesteryl rotamers. R_f_ = 0.45 (petroleum ether/ethyl acetate 5:1). ^1^Н NMR (500 MHz, CDCl_3_): δ_H_ 6.23–6.37 (m, 4Н), 5.84–5.99 (m, 4Н), 5.33–5.39 (m, 2H), 5.20 (d, J = 11.5 Hz, 2Н), 4.94 (d, J = 5.1 Hz, 1Н), 4.88 (d, J = 5.1 Hz, 1Н), 4.77 (d, J = 3.5 Hz, 1Н), 4.73 (d, J = 3.4 Hz, 1Н), 4.43–4.52 (m, 2Н), 2.23–2.39 (m, 4Н), 2.13–2.22 (m, 4Н), 1.93–2.05 (m, 4Н), 1.76–1.89 (m, 6Н), 1.23–1.61 (m, 30Н), 1.05–1.22 (m, 14Н), 0.99–1.04 (m, 10Н), 0.93 (d, J = 6.5 Hz, 8Н), 0.90–0.92 (m, 6Н), 0.89 (d, J = 2.2 Hz, 6Н), 0.88 (d, J = 2.2 Hz, 6Н), 0.69 (s, 6Н) ppm. ^13^С NMR (125 MHz, CDCl_3_): δ_C_ 153.3 (2С), 140.0, 139.9, 138.5 (2C), 138.4 (2С), 137.7, 137.65, 124.5 (2С), 123.4 (2С), 122.4, 122.3, 115.6, 115.5, 74.34, 74.3, 62.3, 62.2, 60.3, 60.2, 56.7 (2С), 56.1 (2С), 50.0 (2С), 42.3 (2С), 39.8 (2С), 39.5 (2С), 38.6, 38.4, 37.0, 36.96, 36.5 (2C), 36.2 (2C), 35.8 (2C), 31.9 (4C), 30.4, 30.3, 28.2 (3С), 28.18, 28.0 (2С), 26.5, 26.4, 24.3 (2C), 23.9 (2C), 22.8 (2C), 22.6 (2C), 22.4, 22.3, 21.0 (2C), 19.4 (2C), 18.7 (2C), 13.9 (2C), 11.9 (2C) ppm. HRMS (ESI-TOF): calcd. for C_40_H_61_NO_2_Na [M + Na]^+^ 610.4600, found 610.4606.

Сholesteryl (1S*,6R*)-7-phenyl-9-azabicyclo[4.2.1]nona-2,4,7-triene-9-carboxylate equivalent with Сholesteryl (1R*,6S*)-7-phenyl-9-azabicyclo[4.2.1]nona-2,4,7-triene-9-carboxylate (**4b**): Yield 89% (0.541 g), yellowish solid, m. p. = 155–156 °С, [α]_D_^17^—18.5 (c 0.18, CHCl_3_), exists as two N-(CO)O-cholesteryl rotamers. R_f_ = 0.47 (petroleum ether/ethyl acetate 5:1). ^1^Н NMR (500 MHz, CDCl_3_): δ_H_ 7.47 (d, J = 7.4 Hz, 4Н), 7.35 (t, J = 7.5 Hz, 4Н), 7.24–7.30 (m, 2Н), 6.30–6.47 (m, 4Н), 5.88–6.02 (m, 6Н), 5.60 (d, J = 4.8 Hz, 1H), 5.53 (d, J =5.0 Hz, 1Н), 5.39 (s, 2Н), 5.01 (d, J = 2.3 Hz, 1Н), 4.97 (d, J = 2.3 Hz, 1Н), 4.47–4.62 (m, 2Н), 2.21–2.46 (m, 4Н), 1.79–2.08 (m, 10Н), 1.24–1.71 (m, 22Н), 1.08–1.23 (m, 14Н), 0.98–1.07 (m, 10Н), 0.94 (d, J = 6.4 Hz, 8Н), 0.90 (d, J = 1.3 Hz, 6Н), 0.89 (s, 6Н), 0.70 (s, 6Н) ppm. ^13^С NMR (125 MHz, CDCl_3_): δ_C_ 153.4 (2С), 139.9 (2С), 138.9 (2C), 137.2 (2С), 135.4, 135.2, 131.9, 131.7, 128.7 (2C), 128.6 (2C), 127.9 (2С), 126.8 (4C), 124.8 (2C), 124.2 (2C), 122.5, 122.4, 115.6 (2С), 74.6 (2C), 60.9 (2С), 60.7 (2С), 56.7 (2С), 56.1 (2С), 50.00 (2С), 42.3 (2С), 39.8 (2С), 39.5 (2С), 38.6, 38.5, 37.0 (2C), 36.6 (2C), 36.2 (2C), 35.8 (2C), 31.9 (4C), 28.3 (4С), 28.0 (2С), 24.3 (2C), 23.9 (2C), 22.8 (2C), 22.6 (2C), 21.1 (2C), 19.4 (2C), 18.7 (2C), 11.9 (2C) ppm. HRMS (ESI-TOF): calcd. for C_42_H_57_NO_2_Na [M + Na]^+^ 630.4287, found 630.4305.

Сholesteryl (1S*,6R*)-7-(o-tolyl)-9-azabicyclo[4.2.1]nona-2,4,7-triene-9-carboxylate equivalent with Сholesteryl (1R*,6S*)–7-(o-tolyl)-9-azabicyclo[4.2.1]nona-2,4,7-triene-9-carboxylate (**4c**): Yield 87% (0.541 g), yellowish solid, m. p. = 115–116 °С, [α]_D_^18^—10.4 (c 0.49, CHCl_3_), exists as two N-(CO)O-cholesteryl rotamers. R_f_ = 0.44 (petroleum ether/ethyl acetate 5:1). ^1^Н NMR (500 MHz, CDCl_3_): δ_H_ 7.13–7.28 (m, 8Н), 6.35–6.46 (m, 2Н), 6.15–6.26 (m, 2Н), 5.97–6.06 (m, 4Н), 5.52 (d, J = 1.5 Hz, 1Н), 5.49 (d, J = 2.0 Hz, 1H), 5.33–5.43 (m, 4Н), 5.01 (dd, J = 5.1 Hz, J = 2.3 Hz, 1Н), 4.96 (dd, J = 5.1 Hz, J = 2.3 Hz, 1Н), 4.48–4.57 (m, 2Н), 2.21–2.43 (m, 10Н), 1.78–2.07 (m, 10Н), 1.23–1.65 (m, 22Н), 1.07–1.22 (m, 14Н), 0.98–1.07 (m, 10Н), 0.94 (d, J = 6.5 Hz, 8Н), 0.90 (d, J = 2.1 Hz, 6Н), 0.88 (d, J = 2.1 Hz, 6Н), 0.70 (s, 6Н) ppm. ^13^С NMR (125 MHz, CDCl_3_): δ_C_ 153.4 (2С), 139.9 (2С), 138.3 (2C), 137.6 (2С), 136.8 (2C), 134.1 (2C), 132.1 (2C), 130.6, 130.56, 129.8, 129.7, 127.9, 127.8, 125.6, 125.58, 124.7 (2C), 124.2 (2C), 122.4, 122.37, 118.6 (2С), 74.6, 74.55, 62.9 (2C), 60.9, 60.7, 56.7 (2С), 56.1 (2С), 50.0 (2С), 42.3 (2С), 39.8 (2С), 39.5 (2С), 38.6, 38.5, 37.0, 36.96, 36.6 (2C), 36.2 (2C), 35.8 (2C), 31.9 (4C), 28.2 (4С), 28.0 (2С), 24.3 (2C), 23.8 (2C), 22.8 (2C), 22.6 (2C), 21.1 (2C), 20.6, 20.57, 19.4 (2C), 18.7 (2C), 11.9 (2C) ppm. HRMS (ESI-TOF): calcd. for C_43_H_59_NO_2_Na [M + Na]^+^ 644.4443, found 644.4457.

Сholesteryl (1S*,6R*)-7-(4-bromophenyl)-9-azabicyclo[4.2.1]nona-2,4,7-triene-9-carboxylate equivalent with Сholesteryl (1R*,6S*)-7-(4-bromophenyl)-9-azabicyclo[4.2.1]nona-2,4,7-triene-9-carboxylate (**4d**): Yield 81% (0.556 g), yellow solid, m. p. = 127–128 °С, [α]_D_^18^—19.1 (c 0.50, CHCl_3_), exists as two N-(CO)O-cholesteryl rotamers. R_f_ = 0.48 (petroleum ether/ethyl acetate 5:1). ^1^Н NMR (500 MHz, CDCl_3_): δ_H_ 7.45 (d, J = 8.3 Hz, 4Н), 7.26–7.35 (m, 4Н), 6.29–6.42 (m, 4Н), 5.92–6.01 (m, 4Н), 5.89 (d, J = 12.4 Hz, 2Н), 5.54 (d, J = 4.9 Hz, 1H), 5.47 (d, J = 5.0 Hz, 1Н), 5.37 (s, 2Н), 4.99 (d, J = 2.4 Hz, 1Н), 4.95 (dd, J = 4.7 Hz, J = 2.3 Hz, 1Н), 4.48–4.58 (m, 2Н), 2.21–2.43 (m, 4Н), 1.78–2.07 (m, 10Н), 1.23–1.64 (m, 22Н), 1.07–1.22 (m, 14Н), 1.00–1.06 (m, 10Н), 0.94 (d, J = 6.3 Hz, 8Н), 0.89 (s, 6Н), 0.88 (d, J = 1.5 Hz, 6Н), 0.69 (s, 6Н) ppm. ^13^С NMR (125 MHz, CDCl_3_): δ_C_ 153.3 (2С), 139.8 (2С), 138.6 (2C), 137.1 (2С), 134.3, 134.1, 131.8 (2C), 131.76 (2C), 130.9, 130.7, 128.3 (4C), 125.1 (2C), 124.2 (2C), 122.5, 122.4, 121.7 (2С), 116.2 (2C), 74.7, 74.65, 60.8, 60.7 (3C), 56.7 (2С), 56.1 (2С), 50.0 (2С), 42.3 (2С), 39.7 (2С), 39.5 (2С), 38.6, 38.4, 37.0 (2C), 36.6 (2C), 36.2 (2C), 35.8 (2C), 31.9 (2C), 31.88 (2C), 28.3 (3С), 28.2, 28.0 (2С), 24.3 (2C), 23.9 (2C), 22.9 (2C), 22.6 (2C), 21.1 (2C), 19.4 (2C), 18.7 (2C), 11.9 (2C) ppm. HRMS (ESI-TOF): calcd. for C_42_H_56_BrNO_2_Na [M + Na]^+^ 708.3392, found 708.3401.

Сholesteryl (1S*,6R*)-7-(4-fluorophenyl)-9-azabicyclo[4.2.1]nona-2,4,7-triene-9-carboxylate equivalent with Сholesteryl (1R*,6S*)-7-(4-fluorophenyl)-9-azabicyclo[4.2.1]nona-2,4,7-triene-9-carboxylate (**4e**): Yield 86% (0.538 g), yellowish solid, m. p. = 125–126 °С, [α]_D_^18^—17.7 (c 0.48, CHCl_3_), exists as two N-(CO)O-cholesteryl rotamers. R_f_ = 0.43 (petroleum ether/ethyl acetate 5:1). ^1^Н NMR (500 MHz, CDCl_3_): δ_H_ 7.40–7.46 (m, 4Н), 7.03 (t, J = 8.6 Hz, 4Н), 6.31–6.42 (m, 4Н), 5.91–6.02 (m, 4Н), 5.83 (dd, J = 13.4 Hz, J = 2.0 Hz, 2Н), 5.55 (d, J = 5.0 Hz, 1H), 5.49 (d, J = 5.1 Hz, 1Н), 5.38 (s, 2Н), 5.00 (dd, J = 5.0 Hz, J = 2.5 Hz, 1Н), 4.95 (dd, J = 5.2 Hz, J = 2.6 Hz, 1Н), 4.48–4.59 (m, 2Н), 2.21–2.43 (m, 4Н), 1.77–2.08 (m, 10Н), 1.24–1.64 (m, 22Н), 1.08–1.23 (m, 14Н), 1.00–1.07 (m, 10Н), 0.94 (d, J = 6.5 Hz, 8Н), 0.90 (d, J = 2.1 Hz, 6Н), 0.88 (d, J = 2.0 Hz, 6Н), 0.70 (s, 6Н) ppm. ^13^С NMR (125 MHz, CDCl_3_): δ_C_ 162.3 (d, J = 246.3 Hz, 2C), 153.3 (2С), 139.8 (2С), 138.8, 138.7, 137.3, 137.25, 134.4, 134.2, 128.5 (2C), 128.4 (2C), 128.1, 128.0, 125.0, 124.9, 124.1 (2C), 122.5, 122.4, 115.7, 115.69, 115.5 (4C), 74.7, 74.6, 61.0, 60.92, 60.8, 60.7, 56.7 (2С), 56.2 (2С), 50.00 (2С), 42.3 (2С), 39.8 (2С), 39.5 (2С), 38.6, 38.5, 37.0, 36.97, 36.6 (2C), 36.2 (2C), 35.8 (2C), 31.9 (2С), 31.89 (2С), 28.3 (3С), 28.2, 28.0 (2С), 24.3 (2C), 23.9 (2C), 22.8 (2C), 22.6 (2C), 21.1 (2C), 19.4 (2C), 18.7 (2C), 11.9 (2C) ppm. HRMS (ESI-TOF): calcd. for C_42_H_56_FNO_2_Na [M + Na]^+^ 648.4193, found 648.4202.

Сholesteryl (1S*,6R*)-7-(trimethylsilyl)-9-azabicyclo[4.2.1]nona-2,4,7-triene-9-carboxylate equivalent with Сholesteryl (1R*,6S*)-7-(trimethylsilyl)-9-azabicyclo[4.2.1]nona-2,4,7-triene-9-carboxylate (**4f**): Yield 85% (0.513 g), white solid, m. p. = 124–125 °С, [α]_D_^18^—20 (c 0.50, CHCl_3_), exists as two N-(CO)O-cholesteryl rotamers. R_f_ = 0.41 (petroleum ether/ethyl acetate 5:1). ^1^Н NMR (500 MHz, CDCl_3_): δ_H_ 6.18–6.30 (m, 4Н), 5.87–6.00 (m, 4Н), 5.52 (d, J = 7.8 Hz, 2Н), 5.34 (s, 2H), 5.00 (d, J = 5.0 Hz, 1Н), 4.91 (d, J = 5.0 Hz, 2Н), 4.86 (d, J = 3.5 Hz, 1Н), 4.41–4.50 (m, 2Н), 2.17–2.38 (m, 4Н), 1.92–2.06 (m, 4Н), 1.74–1.91 (m, 6Н), 1.22–1.62 (m, 22Н), 1.05–1.21 (m, 14Н), 0.98–1.04 (m, 10Н), 0.92 (d, J = 6.5 Hz, 8Н), 0.88 (d, J = 1.7 Hz, 6Н), 0.87 (d, J = 1.7 Hz, 6Н), 0.68 (s, 6Н), 0.13 (d, J = 4.0 Hz, 18Н) ppm. ^13^С NMR (125 MHz, CDCl_3_): δ_C_ 153.2 (2C), 139.9 (2С), 137.7 (2C), 136.2, 136.1, 135.1, 135.0, 126.6, 126.5, 124.3, 124.27, 123.7, 123.6, 122.3, 122.26, 74.3, 74.26, 63.4, 63.3, 61.5, 61.3, 56.7 (2С), 56.1 (2С), 50.0 (2С), 42.3 (2С), 39.8 (2С), 39.5 (2С), 38.6, 38.4, 37.0, 36.97, 36.5 (2C), 36.2 (2C), 35.8 (2C), 31.9 (2С), 31.88 (2С), 28.3 (3С), 28.1, 28.0 (2С), 24.3 (2C), 23.9 (2C), 22.9 (2C), 22.6 (2C), 21.1 (2C), 19.4 (2C), 18.7 (2C), 11.9 (2C), −0.6 (3C), −0.7 (3C) ppm. HRMS (ESI-TOF): calcd. for C_39_H_61_NO_2_SiNa [M + Na]^+^ 626.4369, found 626.4376.

Сholesteryl (1S*,6R*)-7-(2-hydroxyethyl)-9-azabicyclo[4.2.1]nona-2,4,7-triene-9-carboxylate equivalent with Сholesteryl (1R*,6S*)-7-(2-hydroxyethyl)-9-azabicyclo[4.2.1]nona-2,4,7-triene-9-carboxylate (**4g**): Yield 79% (0.455 g), yellowish solid, m. p. = 134–135 °С, [α]_D_^18^—16.9 (c 0.49, CHCl_3_), exists as two N-(CO)O-cholesteryl rotamers. R_f_ = 0.50 (petroleum ether/ethyl acetate 5:1). ^1^Н NMR (500 MHz, CDCl_3_): δ_H_ 6.25–6.36 (m, 4Н), 5.85–6.01 (m, 4Н), 5.28–5.38 (m, 4Н), 4.95 (d, J = 5.0 Hz, 1H), 4.91 (d, J = 5.1 Hz, 1Н), 4.78 (s, 1Н), 4.75 (s, 1Н), 4.38–4.51 (m, 2Н), 3.69 (d, J = 5.8 Hz, 4Н), 2.35–2.50 (m, 4Н), 2.15–2.34 (m, 4Н), 1.91–2.04 (m, 4Н), 1.73–1.90 (m, 6Н), 1.21–1.60 (m, 22Н), 1.04–1.19 (m, 14Н), 0.97–1.03 (m, 10Н), 0.91 (d, J = 6.4 Hz, 8Н), 0.87 (d, J = 1.6 Hz, 6Н), 0.86 (s, 6Н), 0.67 (s, 6Н) ppm. ^13^С NMR (125 MHz, CDCl_3_): δ_C_ 153.3 (2C), 139.8 (2С), 138.5, 138.4, 138.1 (2C), 133.5 (2C), 124.8 (2C), 123.6, 123.55, 122.4, 122.36, 117.9, 117.6, 74.5 (2C), 62.1, 62.06, 61.1, 61.0, 60.2, 60.15, 56.7 (2С), 56.1 (2С), 50.0 (2С), 42.3 (2С), 39.7 (2С), 39.5 (2С), 38.6, 38.4, 37.0, 36.9, 36.5 (2C), 36.2 (2C), 35.8 (2С), 31.9 (2С), 31.86 (2C), 30.5, 30.4, 28.2 (3С), 28.17, 28.0 (2С), 24.3 (2C), 23.9 (2C), 22.8 (2C), 22.6 (2C), 21.0 (2C), 19.4 (2C), 18.7 (2C), 11.9 (2C) ppm. HRMS (ESI-TOF): calcd. for C_38_H_57_NO_3_Na [M + Na]^+^ 598.4236, found 598.4238.

Сholesteryl (1S*,6R*)-7-(3-hydroxypropyl)-9-azabicyclo[4.2.1]nona-2,4,7-triene-9-carboxylate equivalent with Сholesteryl (1R*,6S*)-7-(3-hydroxypropyl)-9-azabicyclo[4.2.1]nona-2,4,7-triene-9-carboxylate (**4h**): Yield 89% (0.525 g), yellowish solid, m. p. = 142–143 °С, [α]_D_^18^—17.2 (c 0.49, CHCl_3_), exists as two N-(CO)O-cholesteryl rotamers. R_f_ = 0.52 (petroleum ether/ethyl acetate 5:1). ^1^Н NMR (500 MHz, CDCl_3_): δ_H_ 6.20–6.35 (m, 4Н), 5.82–5.98 (m, 4Н), 5.34 (s, 2Н), 5.22 (d, J = 9.0 Hz, 2H), 4.92 (d, J = 4.8 Hz, 1H), 4.88 (d, J = 4.9 Hz, 1Н), 4.75 (d, J = 2.8 Hz, 1Н), 4.72 (s, 1Н), 4.39–4.50 (m, 2Н), 3.60 (s, 4Н), 2.10–2.37 (m, 8Н), 1.91–2.04 (m, 4Н), 1.65–1.90 (m, 10Н), 1.21–1.60 (m, 22Н), 1.04–1.19 (m, 14Н), 0.97–1.03 (m, 10Н), 0.91 (d, J = 6.4 Hz, 8Н), 0.87 (d, J = 1.4 Hz, 6Н), 0.86 (d, J = 1.2 Hz, 6Н), 0.67 (s, 6Н) ppm. ^13^С NMR (125 MHz, CDCl_3_): δ_C_ 153.4 (2C), 139.8 (2С), 138.4 (2С), 138.2 (2С), 136.8, 136.7, 124.7 (2C), 123.5 (2С), 122.4, 122.3, 116.0, 115.8, 74.5 (2C), 62.3 (2С), 62.0, 61.9, 60.3, 60.2, 56.7 (2С), 56.1 (2С), 50.0 (2С), 42.3 (2С), 39.7 (2С), 39.5 (2С), 38.6, 38.4, 37.0, 36.9, 36.5 (2C), 36.2 (2C), 35.8 (2С), 31.9 (2С), 31.86 (2C), 31.0 (2C), 28.2 (4С), 28.0 (2С), 24.3 (2C), 23.8 (2C), 23.1, 23.0, 22.8 (2C), 22.6 (2C), 21.0 (2C), 19.4 (2C), 18.7 (2C), 11.9 (2C) ppm. HRMS (ESI-TOF): calcd for C_39_H_59_NO_3_Na [M + Na]^+^ 612.4392, found 612.4389.

Сholesteryl(1S*,6R*)-7-(2-cyanoethyl)-9-azabicyclo[4.2.1]nona-2,4,7-triene-9-carboxylate equivalent with Сholesteryl (1R*,6S*)-7-(2-cyanoethyl)-9-azabicyclo[4.2.1]nona-2,4,7-triene-9-carboxylate (**4i**): Yield 86% (0.503 g), white solid, m. p. = 168–169 °С, [α]_D_^23^—23 (c 0.51, CHCl_3_), exists as two N-(CO)O-cholesteryl rotamers. R_f_ = 0.48 (petroleum ether/ethyl acetate 5:1). ^1^Н NMR (500 MHz, CDCl_3_): δ_H_ 6.24–6.37 (m, 4Н), 5.89–6.04 (m, 4Н), 5.33–5.41 (m, 4Н), 4.96 (d, J = 5.1 Hz, 1H), 4.93 (d, J = 5.1 Hz, 1Н), 4.83 (d, J = 3.4 Hz, 1Н), 4.79 (d, J = 3.3 Hz, 1Н), 4.42–4.52 (m, 2Н), 2.55 (d, J = 6.7 Hz, 4Н), 2.50 (dd, J = 10.9 Hz, J = 4.2 Hz, 4Н), 2.17–2.39 (m, 4Н), 1.92–2.05 (m, 4Н), 1.74–1.91 (m, 6Н), 1.22–1.62 (m, 22Н), 1.05–1.20 (m, 14Н), 0.98–1.04 (m, 10Н), 0.92 (d, J = 6.5 Hz, 8Н), 0.88 (d, J = 2.1 Hz, 6Н), 0.87 (d, J = 2.1 Hz, 6Н), 0.68 (s, 6Н) ppm. ^13^С NMR (125 MHz, CDCl_3_): δ_C_ 153.2 (2C), 139.8 (2С), 138.2, 138.15, 137.4 (2C), 132.8, 132.6, 125.6 (2C), 123.9 (2С), 122.5, 122.4, 118.8, 118.7, 117.9, 117.6, 74.6 (2C), 61.8 (2C), 60.2, 60.1, 56.7 (2С), 56.1 (2С), 50.0 (2С), 42.3 (2С), 39.7 (2С), 39.5 (2С), 38.6, 38.4, 37.0, 36.9, 36.5 (2C), 36.2 (2C), 35.8 (2С), 31.9 (2С), 31.87 (2C), 28.2 (3С), 28.1, 28.0 (2С), 24.3 (2C), 23.8 (2C), 22.9, 22.8 (3C), 22.6 (2C), 21.0 (2C), 19.4 (2C), 18.7 (2C), 16.53, 16.5, 11.9 (2C) ppm. HRMS (ESI-TOF): calcd. for C_39_H_56_N_2_O_2_Na [M + Na]^+^ 607.4239, found 607.4238.

Сholesteryl (1S*,6R*)-7-(3-cyanopropyl)-9-azabicyclo[4.2.1]nona-2,4,7-triene-9-carboxylate equivalent with Сholesteryl (1R*,6S*)-7-(3-cyanopropyl)-9-azabicyclo[4.2.1]nona-2,4,7-triene-9-carboxylate (**4j**): Yield 92% (0.551 g), yellowish solid, m. p. = 162–163 °С, [α]_D_^23^—27.6 (c 0.49, CHCl_3_), exists as two N-(CO)O-cholesteryl rotamers. R_f_ = 0.50 (petroleum ether/ethyl acetate 5:1). ^1^Н NMR (500 MHz, CDCl_3_): δ_H_ 6.23–6.38 (m, 4Н), 5.87–6.02 (m, 4Н), 5.36 (d, J = 5.0 Hz, 2Н), 5.29 (s, 2H), 4.92 (d, J = 5.1 Hz, 1Н), 4.88 (d, J = 5.1 Hz, 1Н), 4.80 (d, J = 3.5 Hz, 1Н), 4.76 (d, J = 3.4 Hz, 1Н), 4.42–4.51 (m, 2Н), 2.18–2.42 (m, 12Н), 1.92–2.05 (m, 4Н), 1.75–1.91 (m, 10Н), 1.22–1.62 (m, 22Н), 1.06–1.21 (m, 14Н), 0.99–1.05 (m, 10Н), 0.92 (d, J = 6.4 Hz, 8Н), 0.88 (d, J = 2.0 Hz, 6Н), 0.87 (d, J = 2.1 Hz, 6Н), 0.68 (s, 6Н) ppm. ^13^С NMR (125 MHz, CDCl_3_): δ_C_ 153.2 (2C), 139.8 (2С), 138.4 (2С), 137.8 (2С), 134.4, 134.2, 125.1 (2C), 123.6 (2С), 122.4, 122.37, 119.2 (2С), 117.5, 117.2, 74.5 (2C), 62.0, 61.9, 60.2, 60.1, 56.7 (2С), 56.1 (2С), 50.0 (2С), 42.3 (2С), 39.7 (2С), 39.5 (2С), 38.6, 38.4, 37.0, 36.9, 36.5 (2C), 36.2 (2C), 35.8 (2С), 31.9 (2С), 31.87 (2C), 28.2 (3С), 28.1, 28.0 (2С), 25.6, 25.5, 24.3 (2C), 24.1, 24.07, 23.8 (2C), 22.8 (2C), 22.6 (2C), 21.0 (2C), 19.4 (2C), 18.7 (2C), 16.4, 16.3, 11.9 (2C) ppm. HRMS (ESI-TOF): calcd. for C_40_H_58_N_2_O_2_Na [M + Na]^+^ 621.4396, found 621.4406.

Сholesteryl (1S*,6R*)-7-(3-bromopropyl)-9-azabicyclo[4.2.1]nona-2,4,7-triene-9-carboxylate equivalent with Сholesteryl (1R*,6S*)-7-(3-bromopropyl)-9-azabicyclo[4.2.1]nona-2,4,7-triene-9-carboxylate (**4k**): Yield 88% (0.574 g), yellowish solid, m. p. = 106–107 °С, [α]_D_^18^—18.8 (c 0.49, CHCl_3_), exists as two N-(CO)O-cholesteryl rotamers. R_f_ = 0.50 (petroleum ether/ethyl acetate 5:1). ^1^Н NMR (500 MHz, CDCl_3_): δ_H_ 6.24–6.38 (m, 4Н), 5.87–6.01 (m, 4Н), 5.33–5.39 (m, 2Н), 5.28 (d, J = 6.1 Hz, 2H), 4.94 (d, J = 5.1 Hz, 1Н), 4.89 (d, J = 5.1 Hz, 1Н), 4.79 (d, J = 3.2 Hz, 1Н), 4.75 (d, J = 3.0 Hz, 1Н), 4.43–4.52 (m, 2Н), 3.33–3.43 (m, 4Н), 2.19–2.42 (m, 8Н), 1.93–2.07 (m, 8Н), 1.75–1.92 (m, 6Н), 1.22–1.62 (m, 22Н), 1.06–1.22 (m, 14Н), 0.99–1.05 (m, 10Н), 0.93 (d, J = 6.5 Hz, 8Н), 0.89 (d, J = 2.3 Hz, 6Н), 0.88 (d, J = 2.2 Hz, 6Н), 0.69 (s, 6Н) ppm. ^13^С NMR (125 MHz, CDCl_3_): δ_C_ 153.3 (2C), 139.9 (2С), 138.4 (2С), 138.0 (2С), 135.3, 135.1, 124.9 (2C), 123.6 (2С), 122.4, 122.35, 116.9, 116.6, 74.5 (2C), 62.2, 62.1, 60.3, 60.2, 56.7 (2С), 56.1 (2С), 50.0 (2С), 42.3 (2С), 39.7 (2С), 39.5 (2С), 38.6, 38.4, 37.0, 36.9, 36.6 (2C), 36.2 (2C), 35.8 (2С), 32.8, 32.77, 31.9 (4C), 31.2, 31.1, 28.2 (3С), 28.16, 28.0 (2С), 25.2, 25.1, 24.3 (2C), 23.8 (2C), 22.8 (2C), 22.6 (2C), 21.0 (2C), 19.4 (2C), 18.7 (2C), 11.9 (2C) ppm. HRMS (ESI-TOF): calcd. for C_39_H_58_BrNO_2_Na [M + Na]^+^ 674.3548, found 674.3558.

Сholesteryl (1S*,6R*)-7-(3-ethoxy-3-oxopropyl)-9-azabicyclo[4.2.1]nona-2,4,7-triene-9-carboxylate equivalent with Сholesteryl (1R*,6S*)-7-(3-ethoxy-3-oxopropyl)-9-azabicyclo[4.2.1]nona-2,4,7-triene-9-carboxylate (**4l**): Yield 90% (0.569 g), yellowish viscous oil, [α]_D_^17^—21.2 (c 0.50, CHCl_3_), exists as two N-(CO)O-cholesteryl rotamers. R_f_ = 0.45 (petroleum ether/ethyl acetate 5:1). ^1^Н NMR (500 MHz, CDCl_3_): δ_H_ 6.23–6.35 (m, 4Н), 5.84–5.99 (m, 4Н), 5.35 (d, J = 5.5 Hz, 2Н), 5.24 (d, J = 9.2 Hz, 2H), 4.94 (d, J = 5.1 Hz, 1Н), 4.89 (d, J = 5.1 Hz, 1Н), 4.77 (d, J = 3.4 Hz, 1Н), 4.73 (d, J = 3.3 Hz, 1Н), 4.42–4.51 (m, 2Н), 4.13 (qd, J = 7.1 Hz, J = 3.0 Hz, 4Н), 2.44–2.55 (m, 8Н), 2.16–2.38 (m, 4Н), 1.92–2.05 (m, 4Н), 1.73–1.91 (m, 6Н), 0.96–1.63 (m, 52Н), 0.92 (d, J = 6.4 Hz, 8Н), 0.88 (d, J = 3.1 Hz, 6Н), 0.87 (d, J = 1.8 Hz, 6Н), 0.68 (s, 6Н) ppm. ^13^С NMR (125 MHz, CDCl_3_): δ_C_ 172.6, 172.5, 153.3 (2C), 139.9 (2С), 138.3 (2С), 138.1 (2С), 135.6, 135.5, 124.9 (2C), 123.7 (2С), 122.4, 122.3, 116.3, 116.2, 74.44, 74.4, 62.2 (2C), 60.5 (2C), 60.2, 60.1, 56.7 (2С), 56.1 (2С), 50.0 (2С), 42.3 (2С), 39.7 (2С), 39.5 (2С), 38.6, 38.4, 37.0, 36.9, 36.5 (2C), 36.2 (2C), 35.8 (2С), 32.9 (2C), 31.9 (4C), 28.2 (3С), 28.16, 28.0 (2С), 24.3 (2C), 23.8 (2C), 22.8 (2C), 22.6 (2C), 22.0, 21.9, 21.0 (2C), 19.4 (2C), 18.7 (2C), 14.2 (2C), 11.8 (2C) ppm. HRMS (ESI-TOF): calcd. for C_41_H_61_NO_4_Na [M + Na]^+^ 654.4498, found 654.4515.

Сholesteryl (1S*,6R*)-7-(2-(tert-butylthio)ethyl)-9-azabicyclo[4.2.1]nona-2,4,7-triene-9-carboxylate equivalent with Сholesteryl (1R*,6S*)-7-(2-(tert-butylthio)ethyl)-9-azabicyclo[4.2.1]nona-2,4,7-triene-9-carboxylate (**4m**): Yield 81% (0.525 g), yellowish solid, m. p. = 149–150 °С, [α]_D_^18^—19.3 (c 0.48, CHCl_3_), exists as two N-(CO)O-cholesteryl rotamers. R_f_ = 0.51 (petroleum ether/ethyl acetate 5:1). ^1^Н NMR (500 MHz, CDCl_3_): δ_H_ 6.23–6.37 (m, 4Н), 5.86–6.00 (m, 4Н), 5.36 (dd, J = 10.6 Hz, J = 2.7 Hz, 2Н), 5.28 (d, J = 11.7 Hz, 2H), 4.99 (d, J = 5.1 Hz, 1Н), 4.92 (d, J = 5.1 Hz, 1Н), 4.79 (d, J = 3.4 Hz, 1Н), 4.75 (d, J = 3.7 Hz, 1Н), 4.43–4.52 (m, 2Н), 2.59–2.71 (m, 4Н), 2.19–2.52 (m, 8Н), 1.99 (dd, J = 25.1 Hz, J = 15.0 Hz, 4Н), 1.74–1.92 (m, 6Н), 1.40–1.62 (m, 12Н), 1.34 (d, J = 3.4 Hz, 24Н), 1.22–1.29 (m, 4Н), 1.06–1.21 (m, 14Н), 0.99–1.05 (m, 10Н), 0.93 (d, J = 6.5 Hz, 8Н), 0.88 (d, J = 2.1 Hz, 6Н), 0.87 (d, J = 1.9 Hz, 6Н), 0.69 (s, 6Н) ppm. ^13^С NMR (125 MHz, CDCl_3_): δ_C_ 153.3 (2C), 139.9 (2С), 138.3 (2С), 138.1 (2С), 135.8, 135.6, 124.9 (2C), 123.7 (2С), 122.4, 122.3, 116.6, 116.4, 74.5, 74.4, 62.0, 61.9, 60.2, 60.1, 56.7 (2С), 56.1 (2С), 50.0 (2С), 42.3 (2С), 42.2 (2C), 39.7 (2С), 39.5 (2С), 38.6, 38.4, 36.9 (2C), 36.5 (2C), 36.2 (2C), 35.8 (2С), 31.9 (4C), 31.0 (6C), 28.2 (4С), 28.0 (2С), 27.4, 27.3, 26.9 (2C), 24.3 (2C), 23.8 (2C), 22.8 (2C), 22.6 (2C), 21.0 (2C), 19.4 (2C), 18.7 (2C), 11.9 (2C) ppm. HRMS (ESI-TOF): calcd. for C_42_H_65_NO_2_SNa [M + Na]^+^ 670.4633, found 670.4639.

Сholesteryl (1S*,6R*)-7-(3-(tert-butylthio)propyl)-9-azabicyclo[4.2.1]nona-2,4,7-triene-9-carboxylate equivalent with Сholesteryl (1R*,6S*)-7-(3-(tert-butylthio)propyl)-9-azabicyclo[4.2.1]nona-2,4,7-triene-9-carboxylate (**4n**): Yield 88% (0.583 g), yellowish solid, m. p. = 135–136 °С, [α]_D_^18^—23.5 (c 0.49, CHCl_3_), exists as two N-(CO)O-cholesteryl rotamers. R_f_ = 0.53 (petroleum ether/ethyl acetate 5:1). ^1^Н NMR (500 MHz, CDCl_3_): δ_H_ 6.22–6.38 (m, 4Н), 5.84–6.01 (m, 4Н), 5.35 (s, 2Н), 5.24 (d, J = 11.1 Hz, 2H), 4.93 (d, J = 4.8 Hz, 1Н), 4.88 (d, J = 4.9 Hz, 1Н), 4.78 (s, 1Н), 4.74 (s, 1Н), 4.42–4.52 (m, 2Н), 2.50 (dd, J = 12.0 Hz, J = 6.7 Hz, 4Н), 2.19–2.38 (m, 8Н), 1.92–2.07 (m, 4Н), 1.70–1.91 (m, 10Н), 1.41–1.63 (m, 12Н), 1.22–1.40 (m, 28Н), 1.06–1.21 (m, 14Н), 0.98–1.05 (m, 10Н), 0.93 (d, J = 6.3 Hz, 8Н), 0.88 (s, 6Н), 0.87 (s, 6Н), 0.69 (s, 6Н) ppm. ^13^С NMR (125 MHz, CDCl_3_): δ_C_ 153.3 (2C), 139.9 (2С), 138.4 (2С), 138.2 (2С), 136.3 (2C), 124.7 (2C), 123.5 (2С), 122.4, 122.3, 116.2, 116.16, 74.4 (2C), 62.2 (2C), 60.3, 60.2, 56.7 (2С), 56.1 (2С), 50.0 (2С), 42.3 (2С), 42.0 (2C), 39.7 (2С), 39.5 (2С), 38.6, 38.4, 37.0, 36.95, 36.5 (2C), 36.2 (2C), 35.8 (2С), 31.9 (4C), 31.0 (6C), 28.4, 28.37, 28.2 (4С), 28.0 (2С), 27.6 (2C), 26.2, 26.1, 24.3 (2C), 23.8 (2C), 22.8 (2C), 22.6 (2C), 21.0 (2C), 19.4 (2C), 18.7 (2C), 11.9 (2C) ppm. HRMS (ESI-TOF): calcd. for C_43_H_67_NO_2_SNa [M + Na]^+^ 684.4790, found 684.4810.

Сholesteryl (1S*,6R*)-7-cyclopentyl-9-azabicyclo[4.2.1]nona-2,4,7-triene-9-carboxylate equivalent with Сholesteryl (1R*,6S*)-7-cyclopentyl-9-azabicyclo[4.2.1]nona-2,4,7-triene-9-carboxylate (**4o**): Yield 85% (0.510 g), yellowish solid, m. p. = 125–126 °С, [α]_D_^18^—13.3 (c 0.50, CHCl_3_), exists as two N-(CO)O-cholesteryl rotamers. R_f_ = 0.48 (petroleum ether/ethyl acetate 5:1). ^1^Н NMR (500 MHz, CDCl_3_): δ_H_ 6.22–6.37 (m, 4Н), 5.84–5.97 (m, 4Н), 5.36 (d, J = 5.0 Hz, 2Н), 5.22 (d, J = 11.7 Hz, 2H), 4.98 (d, J = 5.1 Hz, 1Н), 4.91 (d, J = 5.1 Hz, 1Н), 4.76–4.79 (m, 1Н), 4.74 (dd, J = 4.9 Hz, J = 2.1 Hz, 1Н), 4.42–4.53 (m, 2Н), 2.56–2.66 (m, 2Н), 2.17–2.40 (m, 4Н), 1.75–2.05 (m, 14Н), 1.64–1.73 (m, 8Н), 1.22–1.63 (m, 26Н), 1.06–1.21 (m, 14Н), 0.99–1.05 (m, 10Н), 0.93 (d, J = 6.5 Hz, 8Н), 0.89 (d, J = 2.2 Hz, 6Н), 0.88 (d, J = 2.1 Hz, 6Н), 0.69 (s, 6Н) ppm. ^13^С NMR (125 MHz, CDCl_3_): δ_C_ 153.4, 153.3, 141.7 (2С), 140.0 (2С), 138.6 (2С), 138.3 (2C), 124.2 (2C), 123.5 (2С), 122.4 (2C), 114.2 (2C), 74.3 (2C), 62.0 (2C), 60.2, 60.15, 56.7 (2С), 56.1 (2С), 50.0 (2С), 42.3 (2С), 39.7 (2С), 39.5 (2С), 38.6, 38.4, 38.0, 37.9, 37.0 (2C), 36.6 (2C), 36.2 (2C), 35.8 (2С), 32.8, 32.7, 32.4, 32.2, 31.9 (4C), 28.2 (4С), 28.0 (2С), 24.9 (2C), 24.8 (2C), 24.3 (2C), 23.8 (2C), 22.8 (2C), 22.6 (2C), 21.0 (2C), 19.4 (2C), 18.7 (2C), 11.9 (2C) ppm. HRMS (ESI-TOF): calcd. for C_41_H_61_NO_2_Na [M + Na]^+^ 622.4600, found 622.4599.

Сholesteryl (1S*,6R*)-7-cyclohexyl-9-azabicyclo[4.2.1]nona-2,4,7-triene-9-carboxylate equivalent with Сholesteryl (1R*,6S*)-7-cyclohexyl-9-azabicyclo[4.2.1]nona-2,4,7-triene-9-carboxylate (**4p**): Yield 82% (0.503 g), yellowish solid, m. p. = 128–129 °С, [α]_D_^18^—23 (c 0.50, CHCl_3_), exists as two N-(CO)O-cholesteryl rotamers. R_f_ = 0.45 (petroleum ether/ethyl acetate 5:1). ^1^Н NMR (500 MHz, CDCl_3_): δ_H_ 6.21–6.35 (m, 4Н), 5.83–5.96 (m, 4Н), 5.36 (d, J = 4.6 Hz, 2Н), 5.19 (d, J = 11.1 Hz, 2H), 5.02 (d, J = 5.0 Hz, 1Н), 4.95 (d, J = 5.2 Hz, 1Н), 4.77 (dd, J = 5.0 Hz, J = 2.1 Hz, 1Н), 4.73 (dd, J = 5.0 Hz, J = 2.1 Hz, 1Н), 4.42–4.53 (m, 2Н), 2.12–2.40 (m, 6Н), 1.90–2.04 (m, 6Н), 1.72–1.89 (m, 14Н), 1.68 (d, J = 14.2 Hz, 2Н), 1.33–1.63 (m, 18Н), 1.06–1.32 (m, 26Н), 0.99–1.05 (m, 10Н), 0.93 (d, J = 6.5 Hz, 8Н), 0.89 (d, J = 2.1 Hz, 6Н), 0.88 (d, J = 2.1 Hz, 6Н), 0.69 (s, 6Н) ppm. ^13^С NMR (125 MHz, CDCl_3_): δ_C_ 153.4 (2C), 143.4 (2С), 140.0, 139.9, 138.6 (2С), 138.2 (2С), 124.0 (2C), 123.4 (2С), 122.3, 122.27, 113.9, 113.8, 74.3, 74.29, 61.1 (2C), 60.2, 60.1, 56.7 (2С), 56.1 (2С), 50.0 (2С), 42.3 (2С), 39.7 (2С), 39.5 (2С), 38.6, 38.4, 37.0, 36.96, 36.6 (2C), 36.2 (2C), 36.1, 35.9, 35.8 (2С), 33.1 (2C), 32.7, 32.6, 31.9 (4C), 28.2 (4С), 28.0 (2С), 26.4 (2C), 26.2, 26.16, 26.1 (2C), 24.3 (2C), 23.8 (2C), 22.8 (2C), 22.6 (2C), 21.0 (2C), 19.4 (2C), 18.7 (2C), 11.9 (2C) ppm. HRMS (ESI-TOF): calcd. for C_42_H_63_NO_2_Na [M + Na]^+^ 636.4756, found 636.4765.

Сholesteryl(1S*,6R*)-7-(2-(1,3-dioxoisoindolin-2-yl)ethyl)-9-azabicyclo[4.2.1]nona-2,4,7-triene-9-carboxylate equivalent with Сholesteryl (1R*,6S*)-7-(2-(1,3-dioxoisoindolin-2-yl)ethyl)-9-azabicyclo[4.2.1]nona-2,4,7-triene-9-carboxylate  (**4q**): Yield 87% (0.613 g), yellowish solid, m. p. = 127–128 °С, [α]_D_^18^—18.7 (c 0.49, CHCl_3_), exists as two N-(CO)O-cholesteryl rotamers. R_f_ = 0.56 (petroleum ether/ethyl acetate 2:1). ^1^Н NMR (500 MHz, CDCl_3_): δ_H_ 7.79–7.84 (m, 4Н), 7.68–7.73 (m, 4Н), 6.19–6.30 (m, 4Н), 5.71–5.84 (m, 4Н), 5.29–5.38 (m, 4Н), 4.96 (d, J = 4.9 Hz, 1Н), 4.93 (d, J = 5.1 Hz, 1Н), 4.75 (d, J = 3.6 Hz, 1Н), 4.71 (d, J = 4.1 Hz, 1Н), 4.39–4.50 (m, 2Н), 3.73–3.89 (m, 4Н), 2.51–2.69 (m, 4Н), 2.12–2.38 (m, 4Н), 1.70–2.03 (m, 10Н), 1.20–1.60 (m, 22Н), 1.03–1.19 (m, 14Н), 0.97–1.02 (m, 10Н), 0.90 (d, J = 6.4 Hz, 8Н), 0.86 (d, J = 1.9 Hz, 6Н), 0.85 (d, J = 2.0 Hz, 6Н), 0.66 (d, J = 2.4 Hz, 6Н) ppm. ^13^С NMR (125 MHz, CDCl_3_): δ_C_ 168.1 (4C), 153.2 (2C), 139.9 (2С), 138.1 (2С), 137.8 (2С), 134.0 (2C), 133.9 (2C), 132.4, 132.3, 132.0 (4C), 125.0 (2C), 123.6 (2С), 123.2 (4C), 122.3, 122.27, 117.4, 117.3, 74.4, 74.37, 62.2, 62.0, 60.3 (2C), 56.7 (2С), 56.1 (2С), 50.0 (2С), 42.3 (2С), 39.7 (2С), 39.5 (2С), 38.6, 38.4, 37.0 (2C), 36.5 (4C), 36.2 (2C), 35.8 (2C), 31.9 (2C), 31.85 (2C), 28.2 (3С), 28.1, 28.0 (2С), 25.4, 25.3, 24.3 (2C), 23.8 (2C), 22.8 (2C), 22.6 (2C), 21.0 (2C), 19.4 (2C), 18.7 (2C), 11.9 (2C) ppm. HRMS (ESI-TOF): calcd. for C_46_H_60_N_2_O_4_Na [M + Na]^+^ 727.4451, found 727.4463.

Сholesteryl (1S*,6R*)-7-(4-(1,3-dioxoisoindolin-2-yl)butyl)-9-azabicyclo[4.2.1]nona-2,4,7-triene-9-carboxylate equivalent with Сholesteryl (1R*,6S*)-7-(4-(1,3-dioxoisoindolin-2-yl)butyl)-9-azabicyclo[4.2.1]nona-2,4,7-triene-9-carboxylate (**4r**): Yield 95% (0.696 g), yellowish solid, m. p. = 122–123 °С, [α]_D_^18^—15 (c 0.49, CHCl_3_), exists as two N-(CO)O-cholesteryl rotamers. R_f_ = 0.60 (petroleum ether/ethyl acetate 2:1). ^1^Н NMR (500 MHz, CDCl_3_): δ_H_ 7.82 (d, J = 2.7 Hz, 4Н), 7.70 (s, 4Н), 6.19–6.33 (m, 4Н), 5.81–5.94 (m, 4Н), 5.32 (s, 2Н), 5.20 (d, J = 10.0 Hz, 2Н), 4.89 (d, J = 4.7 Hz, 1Н), 4.85 (d, J = 4.9 Hz, 1Н), 4.73 (s, 1Н), 4.69 s, 1Н), 4.38–4.49 (m, 2Н), 3.67 (t, J = 6.2 Hz, 4Н), 2.13–2.37 (m, 8Н), 1.61–2.02 (m, 14Н), 1.20–1.60 (m, 26Н), 1.03–1.19 (m, 14Н), 0.93–1.02 (m, 10Н), 0.90 (d, J = 6.2 Hz, 8Н), 0.86 (s, 6Н), 0.85 (s, 6Н), 0.66 (s, 6Н) ppm. ^13^С NMR (125 MHz, CDCl_3_): δ_C_ 168.3 (4C), 153.3 (2C), 139.9 (2С), 138.4 (2С), 138.2 (2C), 136.7, 136.6, 133.9 (4C), 132.1 (4C), 124.6 (2C), 123.5 (2С), 123.2 (4C), 122.3, 122.25, 116.2, 116.0, 74.4, 74.3, 62.1 (2C), 60.2, 60.16, 56.7 (2С), 56.1 (2С), 50.0 (2С), 42.3 (2С), 39.7 (2С), 39.5 (2С), 38.6, 38.4, 37.6 (2C), 37.0 (2C), 36.5 (2C), 36.2 (2C), 35.8 (2C), 31.9 (4C), 28.2 (2С), 28.1 (4C), 28.0 (2С), 26.3, 26.2, 25.5 (2C), 24.3 (2C), 23.8 (2C), 22.8 (2C), 22.6 (2C), 21.0 (2C), 19.4 (2C), 18.7 (2C), 11.8 (2C) ppm. HRMS (ESI-TOF): calcd. for C_48_H_64_N_2_O_4_Na [M + Na]^+^ 755.4764, found 755.4787.

Сholesteryl (1S*,6R*)-7-(naphthalen-1-yl)-9-azabicyclo[4.2.1]nona-2,4,7-triene-9-carboxylate equivalent with Сholesteryl (1R*,6S*)-7-(naphthalen-1-yl)-9-azabicyclo[4.2.1]nona-2,4,7-triene-9-carboxylate (**4s**): Yield 84% (0.553 g), yellowish solid, m. p. = 123–124 °С, [α]_D_^17^—6.7 (c 0.31, CHCl_3_), exists as two N-(CO)O-cholesteryl rotamers. R_f_ = 0.55 (petroleum ether/ethyl acetate 5:1). ^1^Н NMR (500 MHz, CDCl_3_): δ_H_ 8.74 (dd, J = 8.1 Hz, J = 4.1 Hz, 2Н), 8.69 (dd, J = 8.1 Hz, J = 2.4 Hz, 2Н), 8.04 (d, J = 8.0 Hz, 2Н), 7.86 (t, J = 7.5 Hz, 2Н), 7.59–7.72 (m, 6Н), 6.48–6.59 (m, 2Н), 6.05–6.24 (m, 6Н), 5.69–5.74 (m, 2Н), 5.65 (s, 1Н), 5.55 (s, 1Н), 5.36–5.46 (m, 2Н), 5.12–5.16 (m, 1Н), 5.07–5.11 (m, 1Н), 4.59 (s, 2Н), 2.23–2.51 (m, 4Н), 1.81–2.07 (m, 10Н), 1.24–1.72 (m, 22Н), 0.99–1.23 (m, 24Н), 0.96 (s, 8Н), 0.89–0.93 (m, 12Н), 0.71 (d, J = 8.3 Hz, 6Н) ppm. ^13^С NMR (125 MHz, CDCl_3_): δ_C_ 153.5 (2C), 139.9 (2С), 138.2 (2С), 137.6 (2C), 133.2 (2C), 131.1 (2C), 130.6, 130.3, 129.2, 129.0, 128.6 (2C), 128.0 (2С), 127.0 (2С), 126.9 (2C), 126.7 (2C), 126.3 (2C), 124.8 (2C), 124.5 (2C), 123.0 (2C), 122.5 (2C), 119.4 (2C), 74.7, 74.66, 63.6 (2C), 61.0, 60.8, 56.7 (2С), 56.2 (2С), 50.0 (2С), 42.3 (2С), 39.8 (2С), 39.6 (2С), 38.7, 38.5, 37.1 (2C), 36.6 (2C), 36.2 (2C), 35.8 (2C), 31.9 (4C), 28.3 (4С), 28.1 (2С), 24.3 (2C), 23.9 (2C), 22.9 (2C), 22.6 (2C), 21.1 (2C), 19.4 (2C), 18.8 (2C), 11.9 (2C) ppm. HRMS (ESI-TOF): calcd. for C_46_H_59_NO_2_Na [M + Na]^+^ 680.4443, found 680.4451.

Сholesteryl (1S*,6R*)-7-(phenanthren-9-yl)-9-azabicyclo[4.2.1]nona-2,4,7-triene-9-carboxylate equivalent with Сholesteryl (1R*,6S*)-7-(phenanthren-9-yl)-9-azabicyclo[4.2.1]nona-2,4,7-triene-9-carboxylate (**4t**): Yield 79% (0.559 g), yellowish solid, m. p. = 167–168 °С, [α]_D_^18^—11.8 (c 0.49, CHCl_3_), exists as two N-(CO)O-cholesteryl rotamers. R_f_ = 0.57 (petroleum ether/ethyl acetate 5:1). ^1^Н NMR (500 MHz, CDCl_3_): δ_H_ 7.97 (d, J = 4.0 Hz, 2Н), 7.78–7.92 (m, 6Н), 7.39–7.57 (m, 10Н), 6.45–6.56 (m, 2Н), 6.02–6.22 (m, 6Н), 5.66 (d, J = 11.3 Hz, 2Н), 5.58 (s, 1Н), 5.48 (s, 1Н), 5.36–5.45 (m, 2Н), 5.11 (s, 1Н), 5.06 (d, J = 2.2 Hz, 1Н), 4.57 (s, 2Н), 2.22–2.49 (m, 4Н), 1.80–2.09 (m, 10Н), 1.24–1.71 (m, 22Н), 0.99–1.24 (m, 24Н), 0.96 (s, 8Н), 0.91 (d, J = 5.6 Hz, 12Н), 0.71 (d, J = 5.5 Hz, 6Н) ppm. ^13^С NMR (125 MHz, CDCl_3_): δ_C_ 153.5 (2C), 139.9 (2С), 138.1 (2С), 137.6 (2C), 133.7 (2C), 132.8 (4C), 132.2 (2C), 130.7 (2С), 130.6 (2С), 128.4 (2C), 128.3 (2C), 127.0 (2C), 126.4 (4C), 126.0 (4C), 125.4 (2C), 125.1 (2C), 124.8 (2C), 124.4 (2C), 122.4 (2C), 119.4 (2C), 74.7 (2C), 63.6 (2C), 61.0, 60.8, 56.7 (2С), 56.2 (2С), 50.0 (2С), 42.3 (2С), 39.8 (2С), 39.6 (2С), 38.7, 38.5, 37.1 (2C), 36.6 (2C), 36.2 (2C), 35.8 (2C), 31.9 (4C), 28.3 (4С), 28.0 (2С), 24.3 (2C), 23.9 (2C), 22.9 (2C), 22.6 (2C), 21.1 (2C), 19.4 (2C), 18.8 (2C), 11.9 (2C) ppm. HRMS (ESI-TOF): calcd. for C_50_H_61_NO_2_Na [M + Na]^+^ 730.4600, found 248.4612.

*Сholesteryl7,8-bis(hydroxymethyl)-9-azabicyclo[4.2.1]nona-2,4,7-triene-9-carboxylate* (**6**): Yield 80% (0.473 g), white solid, m. p. = 188–189 °С, [α]_D_^18^—20.6 (*c* 0.34, CHCl_3_), exists as two N-(CO)O-cholesteryl rotamers. *R*_f_ = 0.58 (petroleum ether/ethyl acetate 5:1). ^1^Н NMR (500 MHz, CDCl_3_): *δ*_H_ 6.29–6.39 (m, 4Н), 5.86–5.95 (m, 4Н), 5.36 (d, J = 7.4 Hz, 2H), 5.09 (dd, J = 9.2 Hz, J = 5.2 Hz, 4Н), 4.41–4.51 (m, 2Н), 4.25–4.34 (m, 8Н), 2.19–2.37 (m, 4Н), 1.92–2.06 (m, 4Н), 1.75–1.91 (m, 6Н), 1.22–1.63 (m, 22Н), 1.06–1.21 (m, 14Н), 0.96–1.05 (m, 10Н), 0.93 (d, J = 6.5 Hz, 8Н), 0.89 (d, J = 2.2 Hz, 6Н), 0.87 (d, J = 2.2 Hz, 6Н), 0.69 (s, 6Н) ppm. ^13^С NMR (125 MHz, CDCl_3_): *δ*_C_ 153.6 (2С), 139.7 (2C), 138.8 (2C), 138.7 (2С), 132.9 (2C), 132.6 (2C), 124.7 (2С), 124.6 (2С), 122.6, 122.5, 75.0 (2С), 61.8 (2C), 61.75 (2C), 56.7 (2С), 56.1 (2С), 54.9 (2C), 54.8 (2С), 50.0 (2С), 42.3 (2С), 39.7 (2С), 39.5 (2C), 38.6, 38.4, 37.0, 36.9, 36.5 (2C), 36.2 (2C), 35.8 (2C), 31.9 (2C), 31.86 (2C), 28.2 (2С), 28.17 (2C), 28.0 (2С), 24.3 (2C), 23.9 (2C), 22.8 (2C), 22.6 (2C), 21.0 (2C), 19.4 (2C), 18.7 (2C), 11.9 (2C) ppm. HRMS (ESI-TOF): calcd. for C_38_H_57_NO_4_Na [M + Na]^+^ 614.4185, found 614.4199.

## 4. Conclusions

In summary, we synthesized, for the first time, *N*-carbocholesteroxyazepine and studied its [6π + 2π]-cycloaddition reactions with functionally substituted terminal alkynes and 1,4-butynediol under the action of the Co(acac)_2_(dppe)/Zn/ZnI_2_ three-component catalytic system. Our strategy provided a new 9-azabicyclo[4.2.1]nona-2,4,7-triene series bearing, at C-7, a large variety of substituents in high yields (79–95%, 20 examples of feasibility). The synthesized azabicycles possess a high potential for practical application in pharmacology and medicine, as they can be used as key precursors in the targeted search for and development of innovative drugs and other practically significant compounds.

## Figures and Tables

**Scheme 5:**
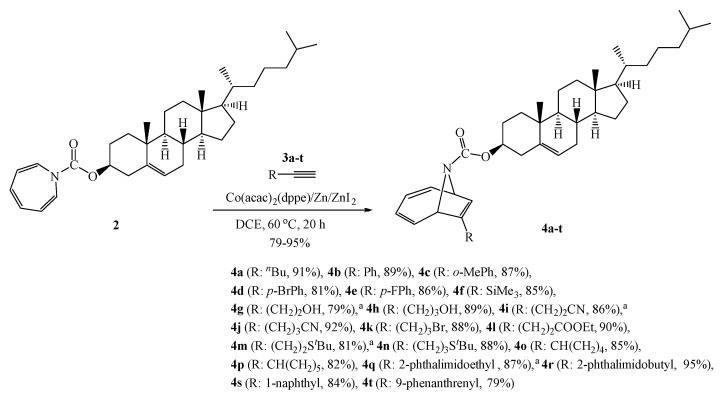
Cycloaddition of *N*-carbocholesteroxyazepine to alkynes. Reaction conditions: **2** (1 mmol), **3** (1.5 mmol), Co(acac)_2_(dppe) (0.10 mmol), Zn (0.3 mmol), ZnI_2_ (0.20 mmol), DCE (3 mL), 60 °C, 20 h. Yields calculated based on effective amounts of material isolated by column chromatography. ^a^ Solvent: DCE:Trifluoroethanol 1:2 *v/v*.

## Data Availability

The data presented in this study are available in the Supplementary Materials.

## Supplementary Materials

The following are available online: 1D (^1^H and ^13^C NMR) and 2D (NOESY, COSY, HSQC, HMBC) spectra of the products synthesized in this work.
