# Supplementary material for: Synthesis of New Functionally Substituted 9-Azabicyclo[4.2.1]nona-2,4,7-trienes by Cobalt(I)-Catalyzed [6π + 2π]-Cycloaddition of N-Carbocholesteroxyazepine to Alkynes
Source: Molecules. 2021 May 14;26(10):2932. doi: 10.3390/molecules26102932 (PMC8156619; doi:10.3390/molecules26102932)

SUPPORTING INFORMATION

Synthesis of New Functionally Substituted 9-Azabicyclo[4.2.1]nona-2,4,7-Trienes by Cobalt(I)-Catalyzed [6π+2π]-Cycloaddition of *N*-Carbocholesteroxyazepine to Alkynes

Gulnara N. Kadikova, Vladimir A. D'yakonov * and Usein M. Dzhemilev

Laboratory of Catalytic Synthesis, Institute of Petrochemistry and Catalysis, Russian Academy of Sciences, Ufa 450075, Russia; Kad.Gulnara@gmail.com (G.N.K.); Dzhemilev@anrb.ru (U.M.D.)

* Correspondence: DyakonovVA@gmail.com; Tel.: +791-7457-8193

Table of Contents

Figure S1. ^{13}C NMR Spectrum of compound **1** S6

Figure S2. ^{1}H NMR Spectrum of compound **1** S6

Figure S3. NOESY Spectrum of compound **1** S7

Figure S4. COSY Spectrum of compound **1** S7

Figure S5. HSQC spectrum of compound **1** S8

Figure S6. HMBC spectrum of compound **1** S8

Figure S7. ^{13}C NMR Spectrum of compound **2** S9

Figure S8. ^{1}H NMR Spectrum of compound **2** S9

Figure S9. NOESY Spectrum of compound **2** S10

Figure S10. COSY Spectrum of compound **2** S10

Figure S11. HSQC spectrum of compound **2** S11

Figure S12. HMBC spectrum of compound **2** S11

Figure S13. ^{13}C NMR Spectrum of compound **4a** S12

Figure S14. ^{1}H NMR Spectrum of compound **4a** S12

Figure S15. NOESY Spectrum of compound **4a** S13
Figure S16. COSY Spectrum of compound **4a** S13
Figure S17. HSQC spectrum of compound **4a** S14
Figure S18. HMBC spectrum of compound **4a** S14
Figure S19. ^{13}C NMR Spectrum of compound **4b** S15
Figure S20. ^{1}H NMR Spectrum of compound **4b** S15
Figure S21. NOESY Spectrum of compound **4b** S16
Figure S22. COSY Spectrum of compound **4b** S16
Figure S23. HSQC spectrum of compound **4b** S17
Figure S24. HMBC spectrum of compound **4b** S17
Figure S25. ^{13}C NMR Spectrum of compound **4c** S18
Figure S26. ^{1}H NMR Spectrum of compound **4c** S18
Figure S27. COSY Spectrum of compound **4c** S19
Figure S28. HSQC spectrum of compound **4c** S19
Figure S29. HMBC spectrum of compound **4c** S20
Figure S30. ^{13}C NMR Spectrum of compound **4d** S21
Figure S31. ^{1}H NMR Spectrum of compound **4d** S21
Figure S32. COSY Spectrum of compound **4d** S22
Figure S33. HSQC spectrum of compound **4d** S22
Figure S34. HMBC spectrum of compound **4d** S23
Figure S35. ^{13}C NMR Spectrum of compound **4e** S24
Figure S36. ^{1}H NMR Spectrum of compound **4e** S24
Figure S37. NOESY Spectrum of compound **4e** S25
Figure S38. COSY Spectrum of compound **4e** S25
Figure S39. HSQC spectrum of compound **4e** S26
Figure S40. HMBC spectrum of compound **4e** S26
Figure S41. ^{13}C NMR Spectrum of compound **4f** S27
Figure S42. ^{1}H NMR Spectrum of compound **4f** S27
Figure S43. COSY Spectrum of compound **4f** S28
Figure S44. HSQC spectrum of compound **4f** S28
Figure S45. HMBC spectrum of compound **4f** S29
Figure S46. ^{13}C NMR Spectrum of compound **4g** S30
Figure S47. ^{1}H NMR Spectrum of compound **4g** S30
Figure S48. NOESY Spectrum of compound **4g** S31

Figure S49. COSY Spectrum of compound **4g** S31

Figure S50. HSQC spectrum of compound **4g** S32

Figure S51. HMBC spectrum of compound **4g** S32

Figure S52. ^{13}C NMR Spectrum of compound **4h** S33

Figure S53. ^{1}H NMR Spectrum of compound **4h** S33

Figure S54. NOESY Spectrum of compound **4h** S34

Figure S55. COSY Spectrum of compound **4h** S34

Figure S56. HSQC spectrum of compound **4h** S35

Figure S57. HMBC spectrum of compound **4h** S35

Figure S58. ^{13}C NMR Spectrum of compound **4i** S36

Figure S59. ^{1}H NMR Spectrum of compound **4i** S36

Figure S60. NOESY Spectrum of compound **4i** S37

Figure S61. COSY Spectrum of compound **4i** S37

Figure S62. HSQC spectrum of compound **4i** S38

Figure S63. HMBC spectrum of compound **4i** S38

Figure S64. ^{13}C NMR Spectrum of compound **4j** S39

Figure S65. ^{1}H NMR Spectrum of compound **4j** S39

Figure S66. ^{13}C NMR Spectrum of compound **4k** S40

Figure S67. ^{1}H NMR Spectrum of compound **4k** S40

Figure S68. NOESY Spectrum of compound **4k** S41

Figure S69. COSY Spectrum of compound **4k** S41

Figure S70. HSQC spectrum of compound **4k** S42

Figure S71. HMBC spectrum of compound **4k** S42

Figure S72. ^{13}C NMR Spectrum of compound **4l** S43

Figure S73. ^{1}H NMR Spectrum of compound **4l** S43

Figure S74. NOESY Spectrum of compound **4l** S44

Figure S75. COSY Spectrum of compound **4l** S44

Figure S76. HSQC spectrum of compound **4l** S45

Figure S77. HMBC spectrum of compound **4l** S45

Figure S78. ^{13}C NMR Spectrum of compound **4m** S46

Figure S79. ^{1}H NMR Spectrum of compound **4m** S46

Figure S80. ^{13}C NMR Spectrum of compound **4n** S47

Figure S81. ^{1}H NMR Spectrum of compound **4n** S47

Figure S82. NOESY Spectrum of compound **4n** S48

Figure S83. COSY Spectrum of compound **4n** S48
Figure S84. HSQC spectrum of compound **4n** S49
Figure S85. HMBC spectrum of compound **4n** S49
Figure S86. ^{13}C NMR Spectrum of compound **4o** S50
Figure S87. ^{1}H NMR Spectrum of compound **4o** S50
Figure S88. ^{13}C NMR Spectrum of compound **4p** S51
Figure S89. ^{1}H NMR Spectrum of compound **4p** S51
Figure S90. COSY Spectrum of compound **4p** S52
Figure S91. HSQC spectrum of compound **4p** S52
Figure S92. HMBC spectrum of compound **4p** S53
Figure S93. ^{13}C NMR Spectrum of compound **4q** S54
Figure S94. ^{1}H NMR Spectrum of compound **4q** S54
Figure S95. COSY Spectrum of compound **4q** S55
Figure S96. HSQC spectrum of compound **4q** S55
Figure S97. HMBC spectrum of compound **4q** S56
Figure S98. ^{13}C NMR Spectrum of compound **4r** S57
Figure S99. ^{1}H NMR Spectrum of compound **4r** S57
Figure S100. COSY Spectrum of compound **4r** S58
Figure S101. HSQC spectrum of compound **4r** S58
Figure S102. HMBC spectrum of compound **4r** S59
Figure S103. ^{13}C NMR Spectrum of compound **4s** S60
Figure S104. ^{1}H NMR Spectrum of compound **4s** S60
Figure S105. COSY Spectrum of compound **4s** S61
Figure S106. HSQC spectrum of compound **4s** S61
Figure S107. HMBC spectrum of compound **4s** S62
Figure S108. ^{13}C NMR Spectrum of compound **4t** S63
Figure S109. ^{1}H NMR Spectrum of compound **4t** S63
Figure S110. COSY Spectrum of compound **4t** S64
Figure S111. HSQC spectrum of compound **4t** S64
Figure S112. HMBC spectrum of compound **4t** S65
Figure S113. ^{13}C NMR Spectrum of compound **6** S66
Figure S114. ^{1}H NMR Spectrum of compound **6** S66
Figure S115. COSY Spectrum of compound **6** S67
Figure S116. HSQC spectrum of compound **6** S67

Figure S117. HMBC spectrum of compound **6** S68
Figure S118. ^{13}C NMR Spectrum of compound **4r** (temperature 298 K) S69
Figure S119. ^{1}H NMR Spectrum of compound **4r** (temperature 298 K) S69
Figure S120. ^{13}C NMR Spectrum of compound **4r** (temperature 333 K) S70
Figure S121. ^{1}H NMR Spectrum of compound **4r** (temperature 333 K) S70

Figure S1. ^{13}C NMR Spectrum of compound **1** (125 MHz, $CDCl_3$)

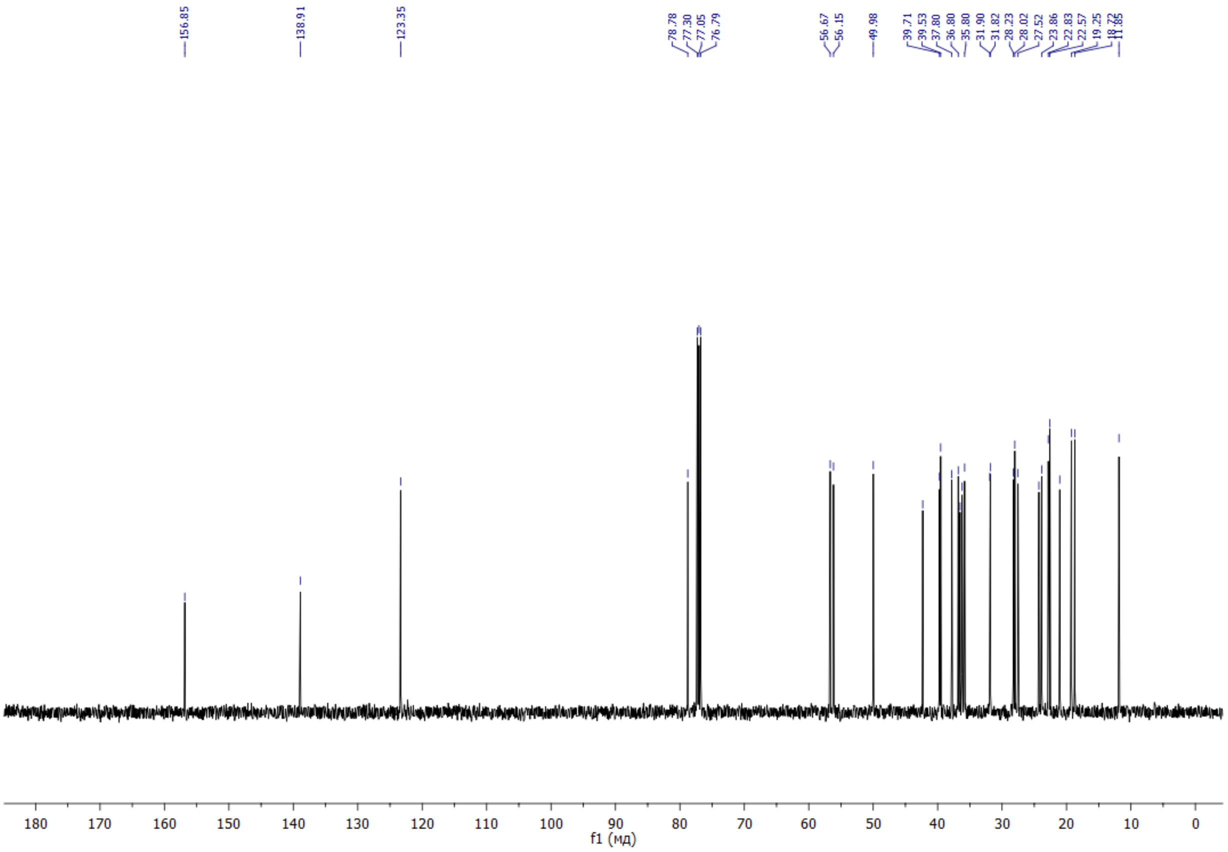

Figure S2. ^{1}H NMR Spectrum of compound **1** (500 MHz, $CDCl_3$)

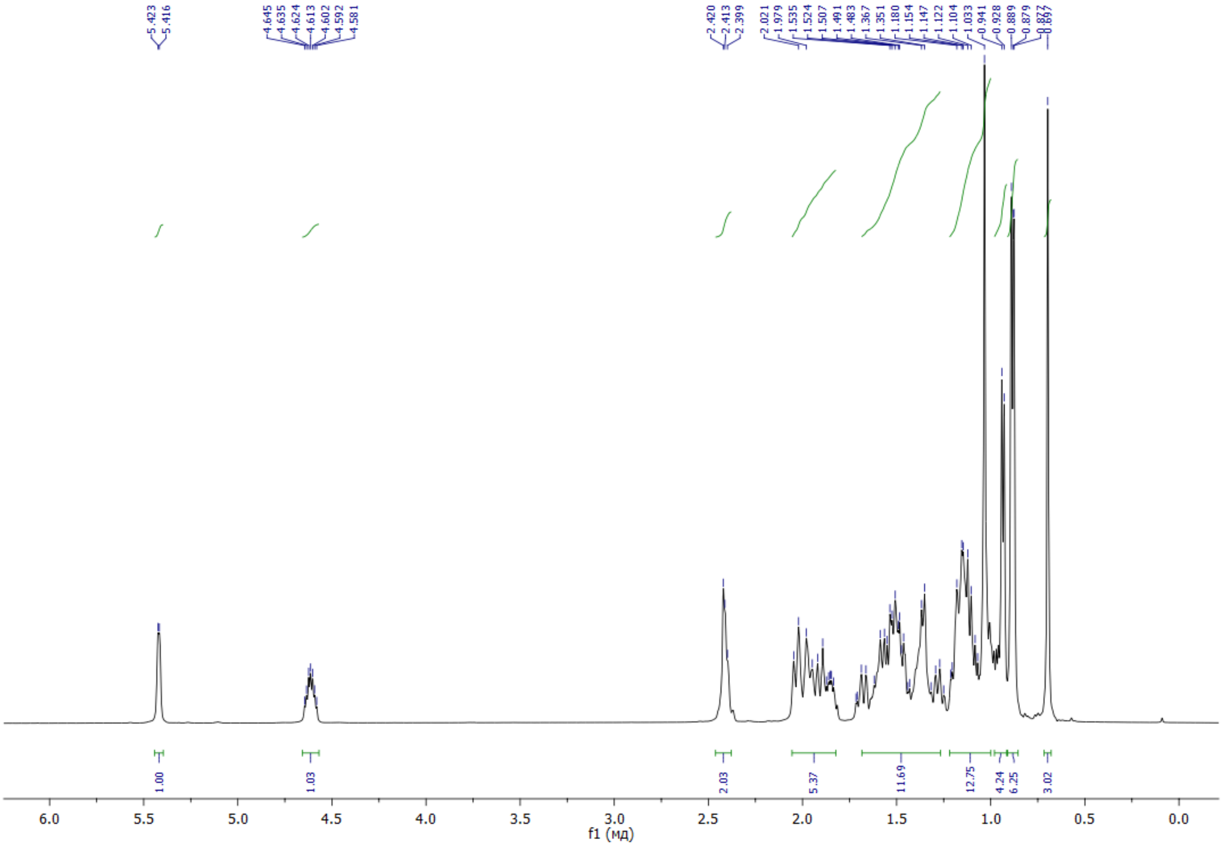

Figure S3. NOESY Spectrum of compound **1** (500 MHz, $CDCl_3$)

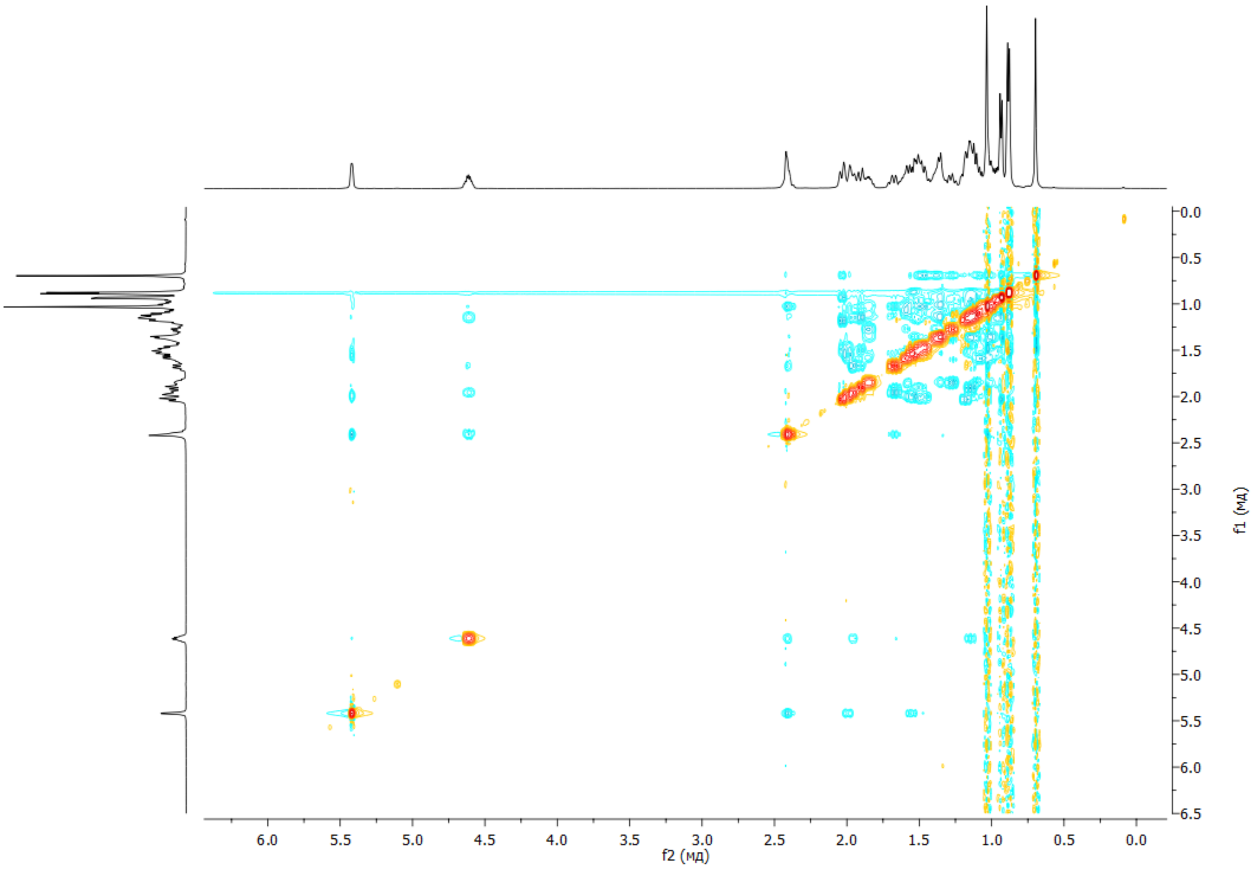

Figure S4. COSY Spectrum of compound **1** (500 MHz, $CDCl_3$)

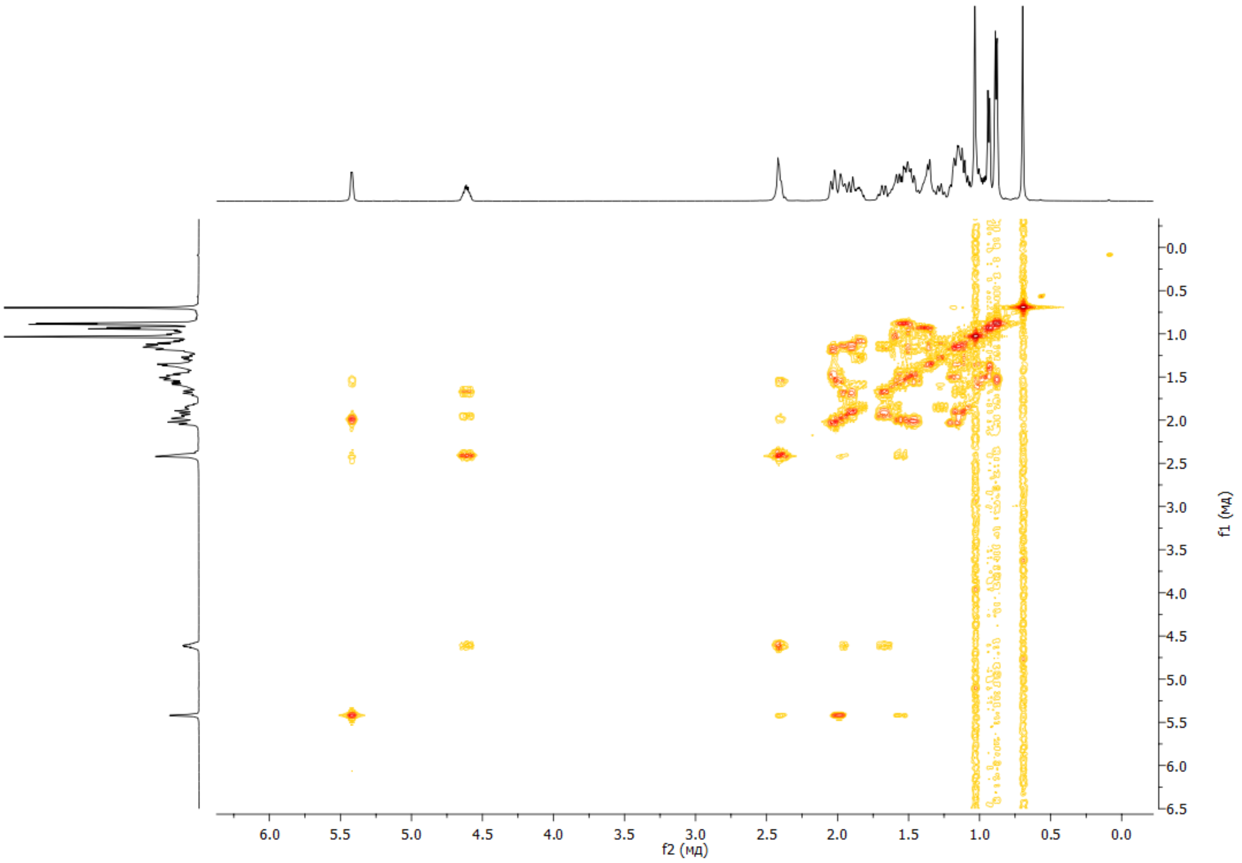

Figure S5. HSQC Spectrum of compound **1** (500 MHz, $CDCl_3$)

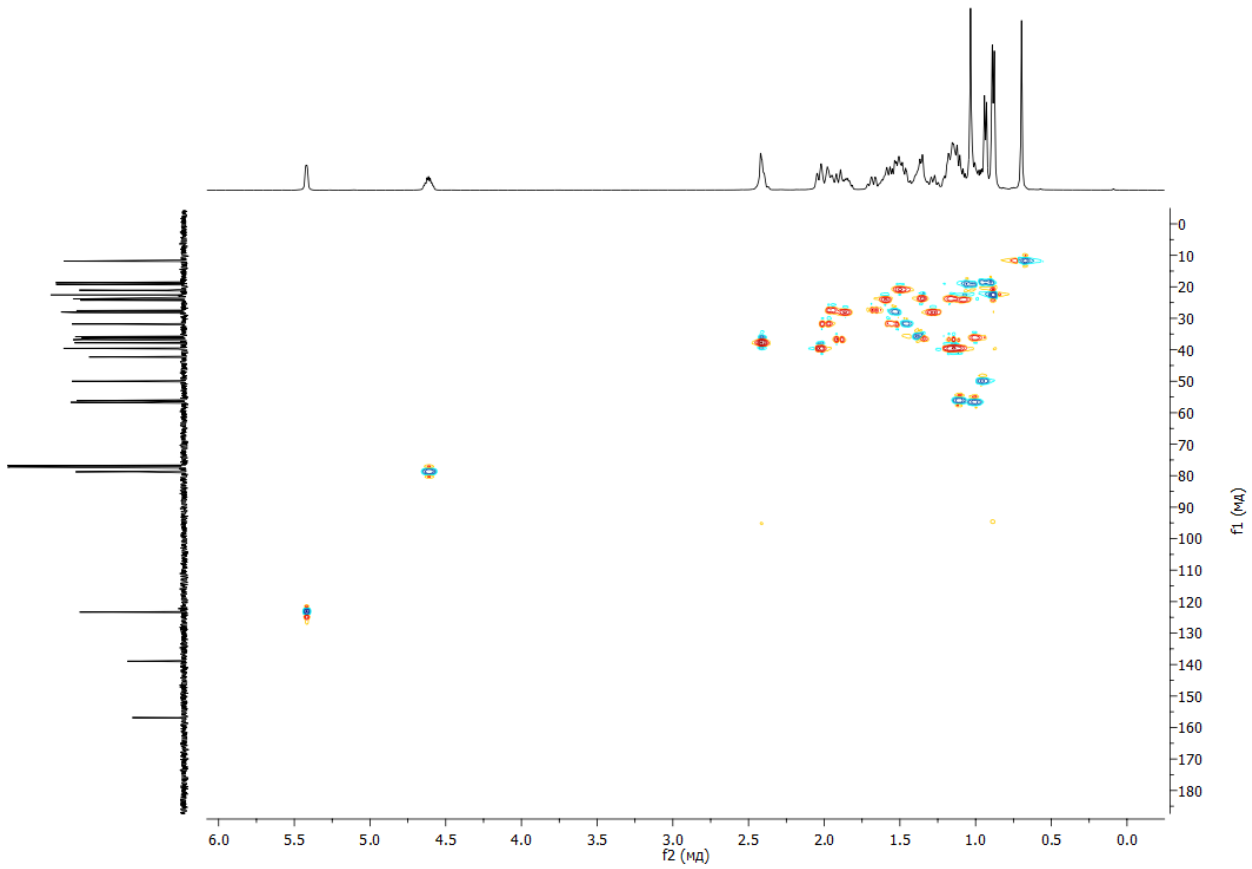

Figure S6. HMBC spectrum of compound **1** (500 MHz, $CDCl_3$)

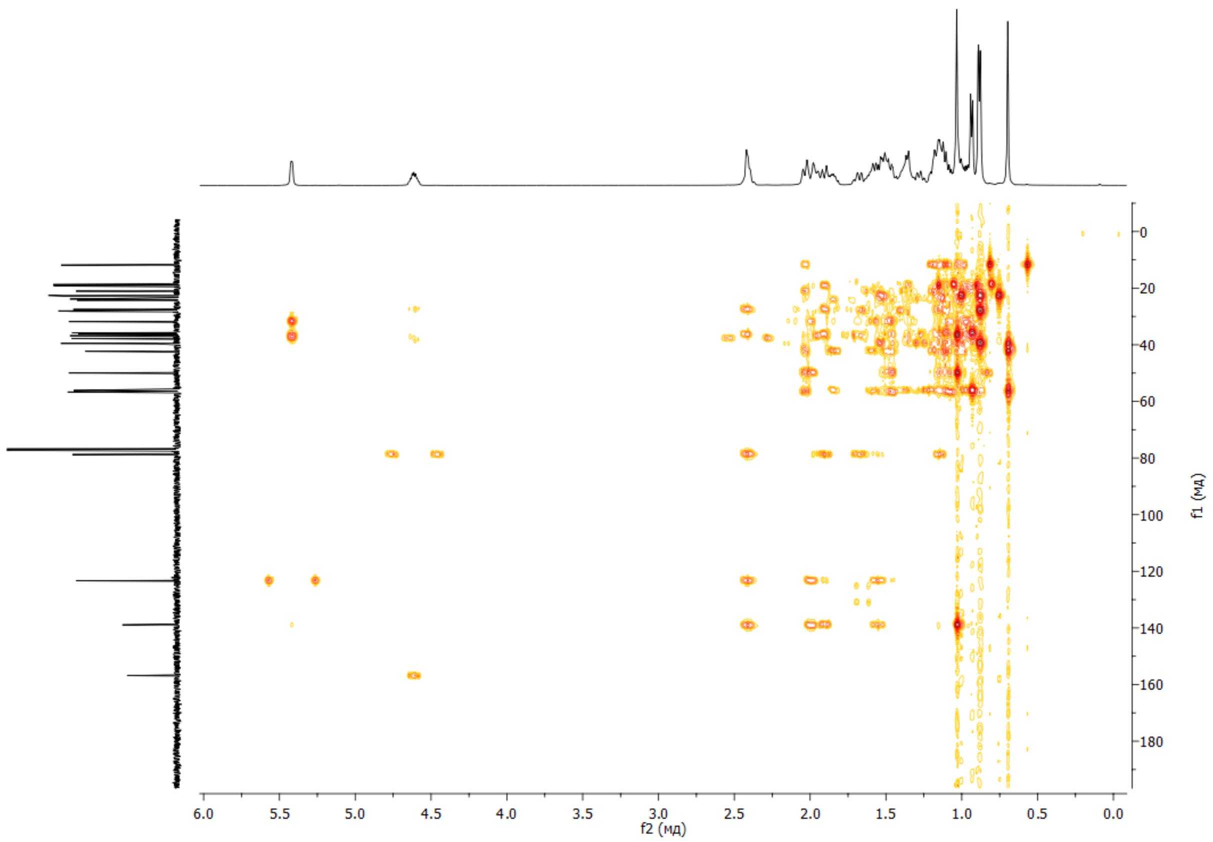

Figure S7. ^{13}C NMR Spectrum of compound **2** (125 MHz, $CDCl_3$)

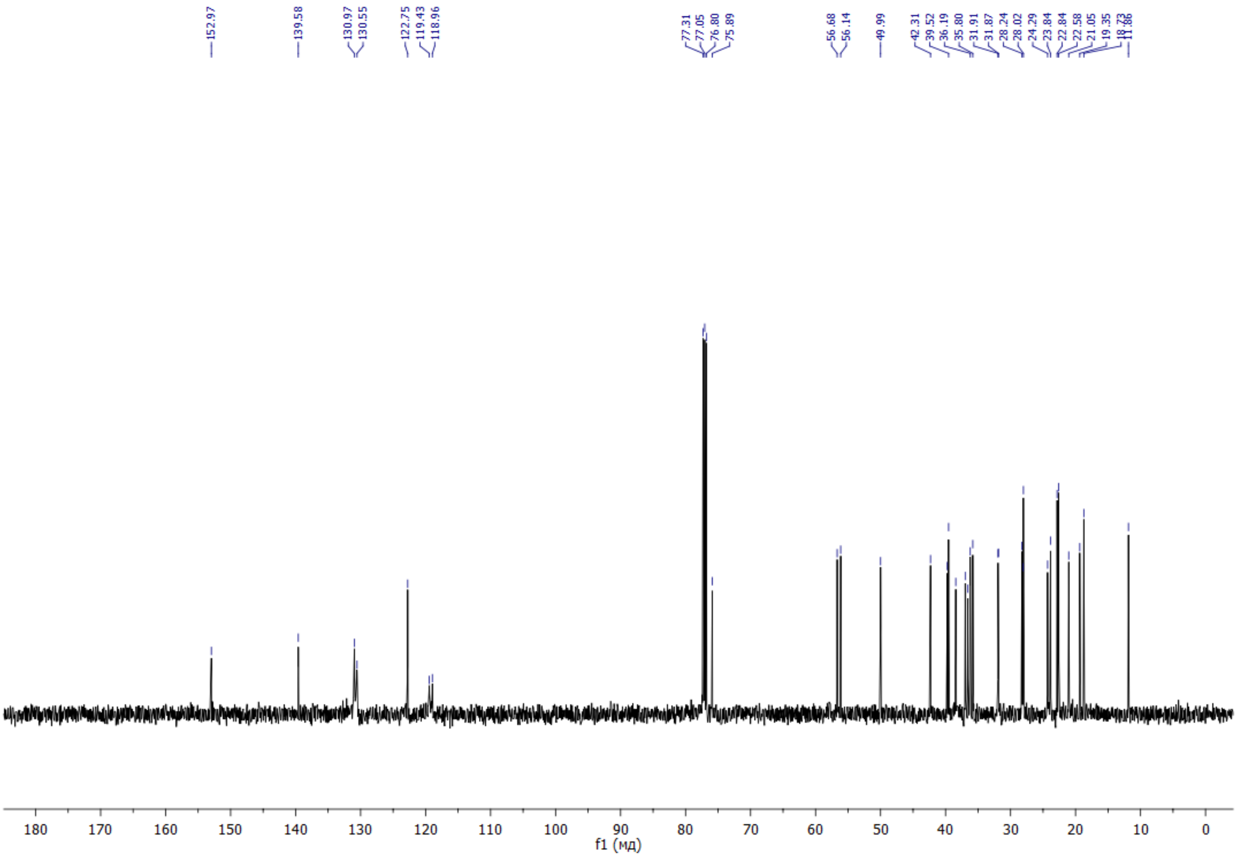

Figure S8. ^{1}H NMR Spectrum of compound **2** (500 MHz, $CDCl_3$)

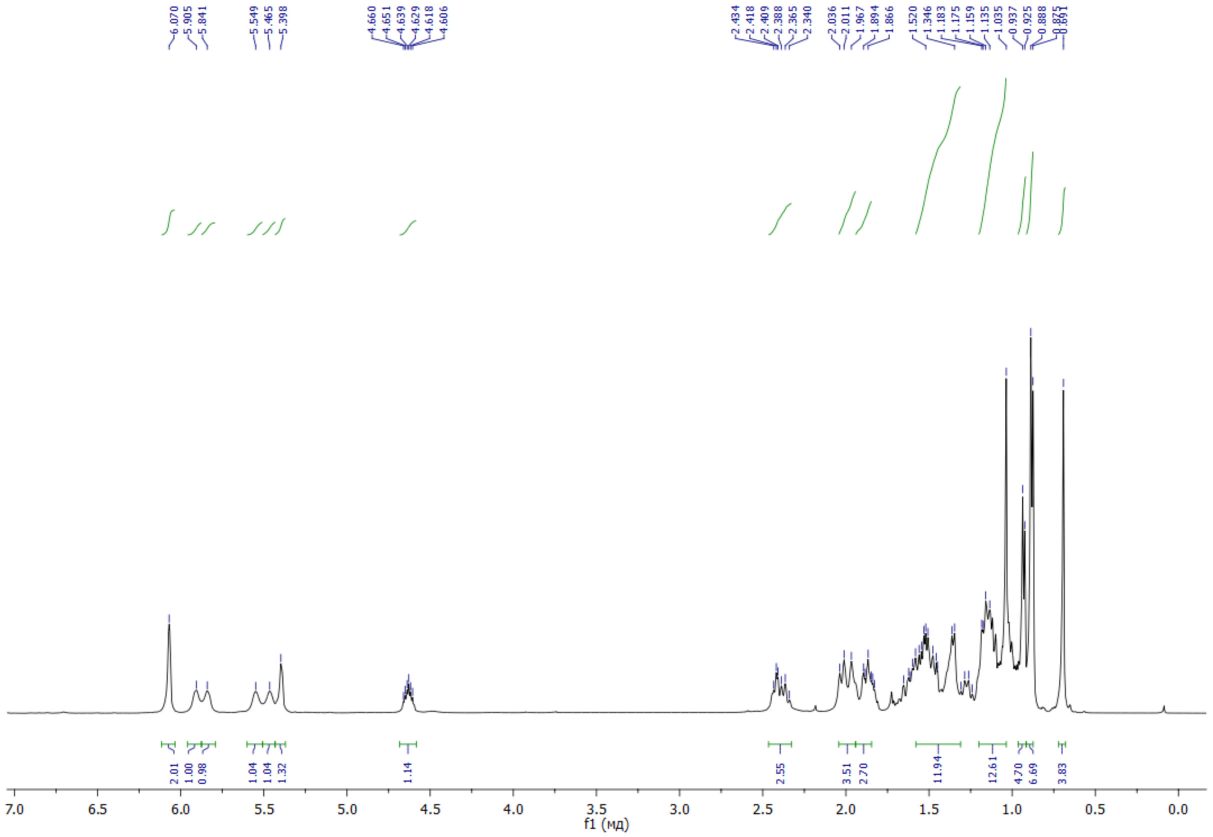

Figure S9. NOESY Spectrum of compound **2** (500 MHz, $CDCl_3$)

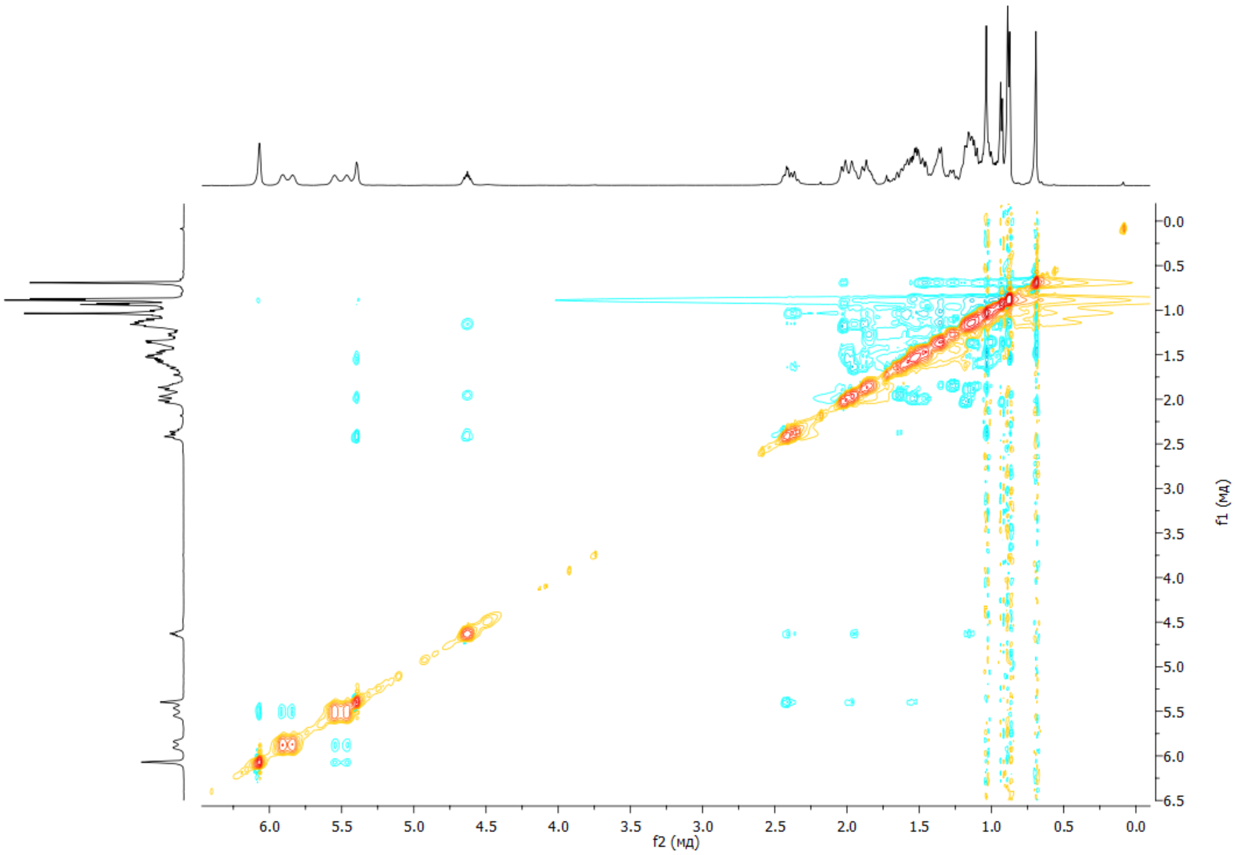

Figure S10. COSY Spectrum of compound **2** (500 MHz, $CDCl_3$)

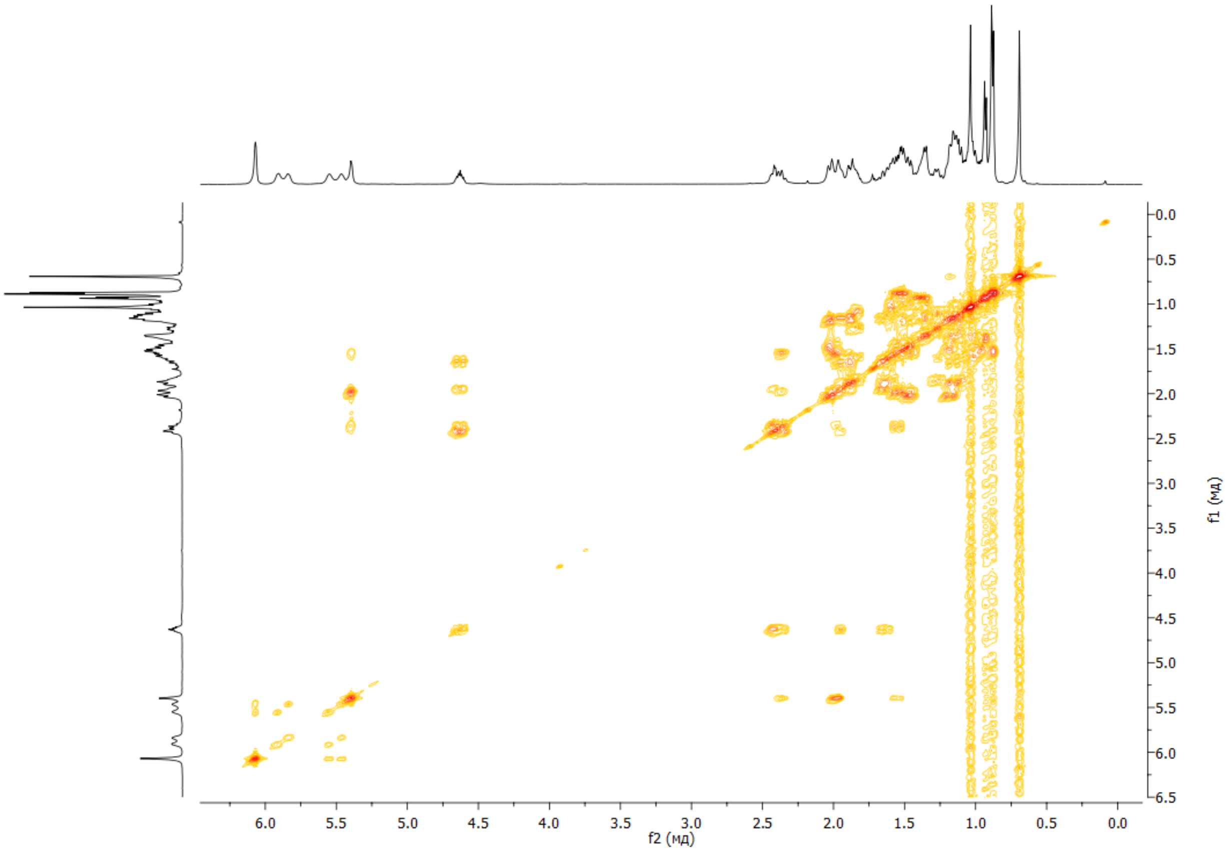

Figure S11. HSQC spectrum of compound **2** (500 MHz, $CDCl_3$)

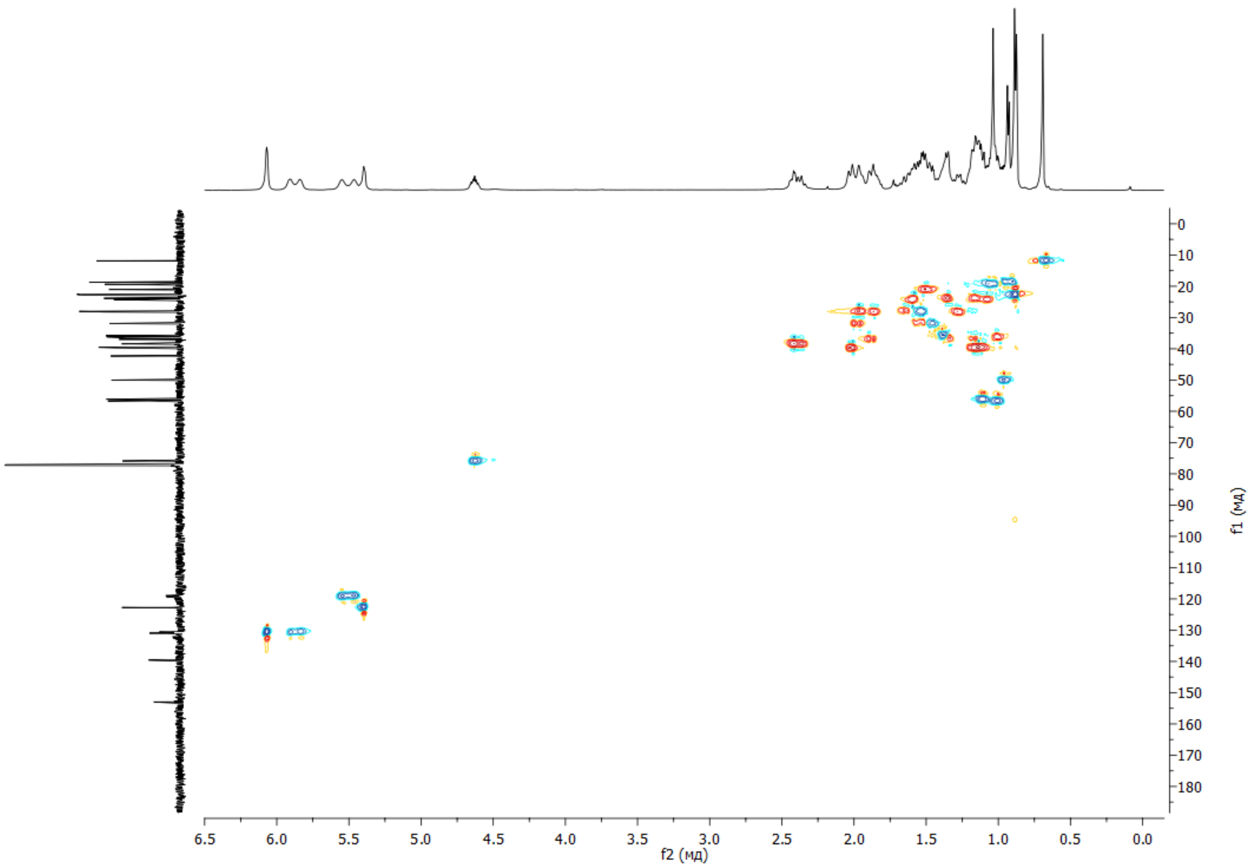

Figure S12. HMBC spectrum of compound **2** (500 MHz, $CDCl_3$)

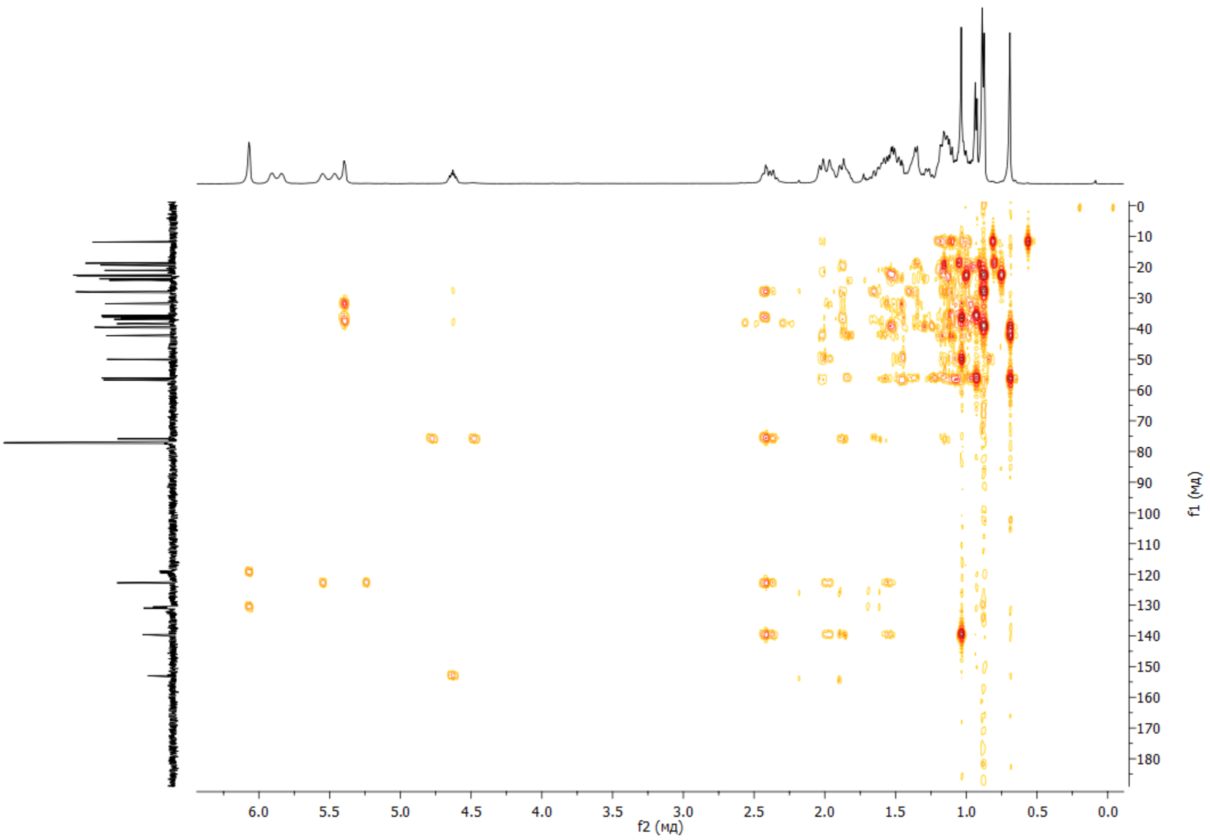

Figure S13. ^{13}C NMR Spectrum of compound **4a** (125 MHz, $CDCl_3$)

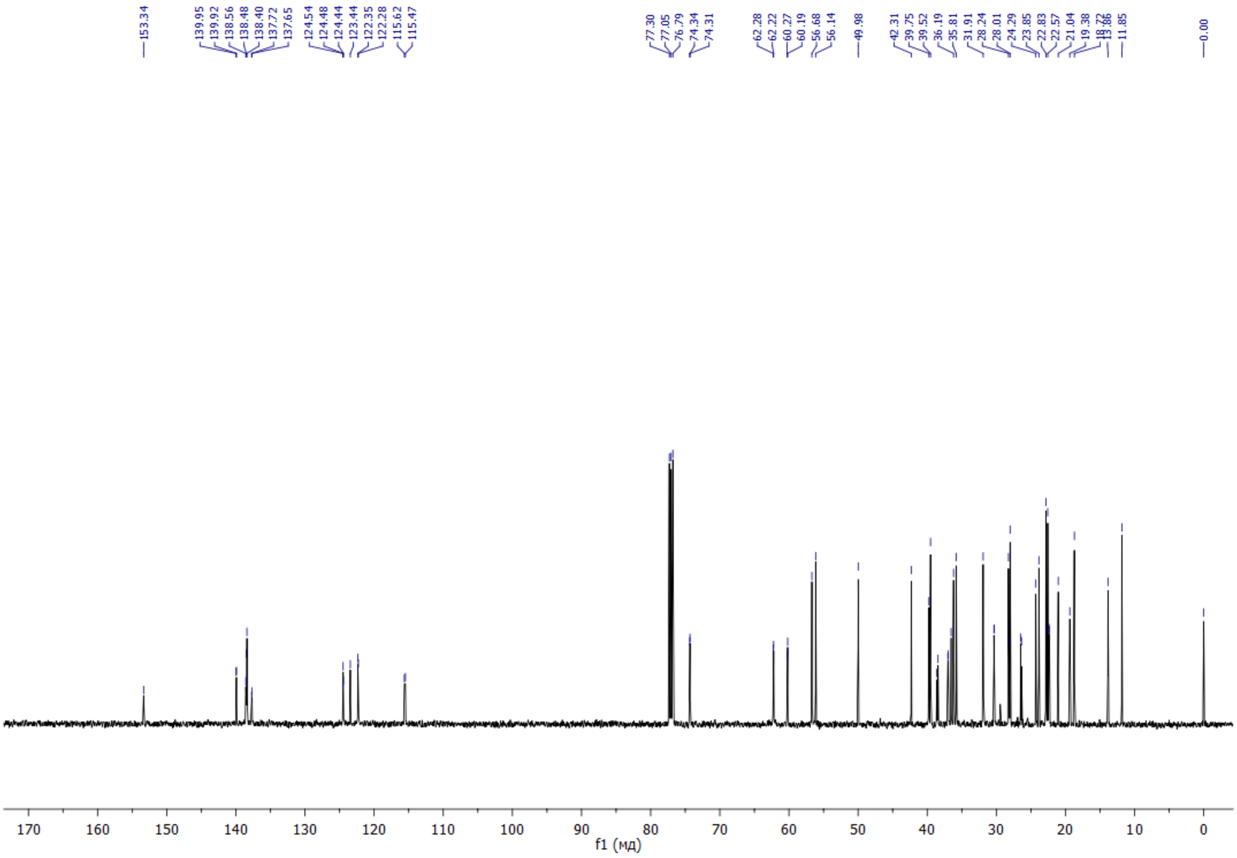

Figure S14. ^{1}H NMR Spectrum of compound **4a** (500 MHz, $CDCl_3$)

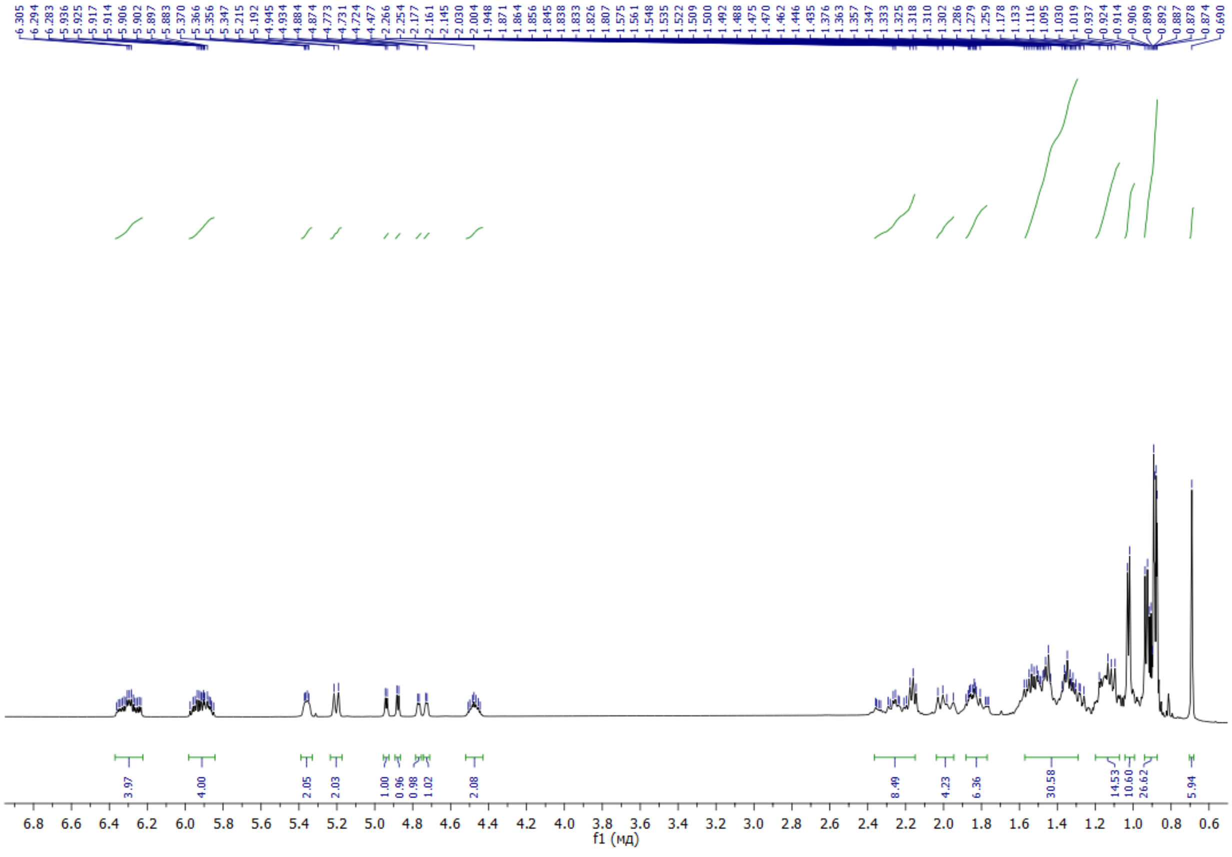

Figure S15. NOESY Spectrum of compound **4a** (500 MHz, $CDCl_3$)

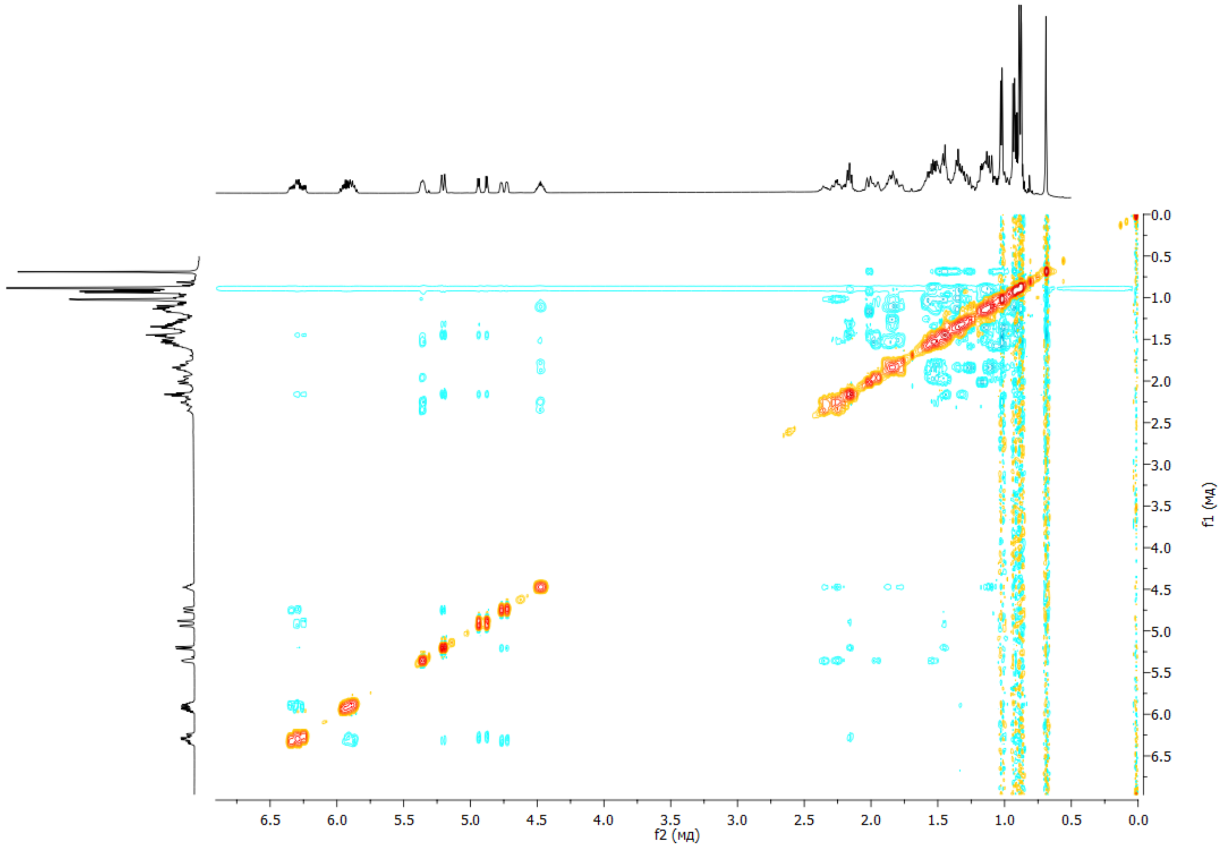

Figure S16. COSY Spectrum of compound **4a** (500 MHz, $CDCl_3$)

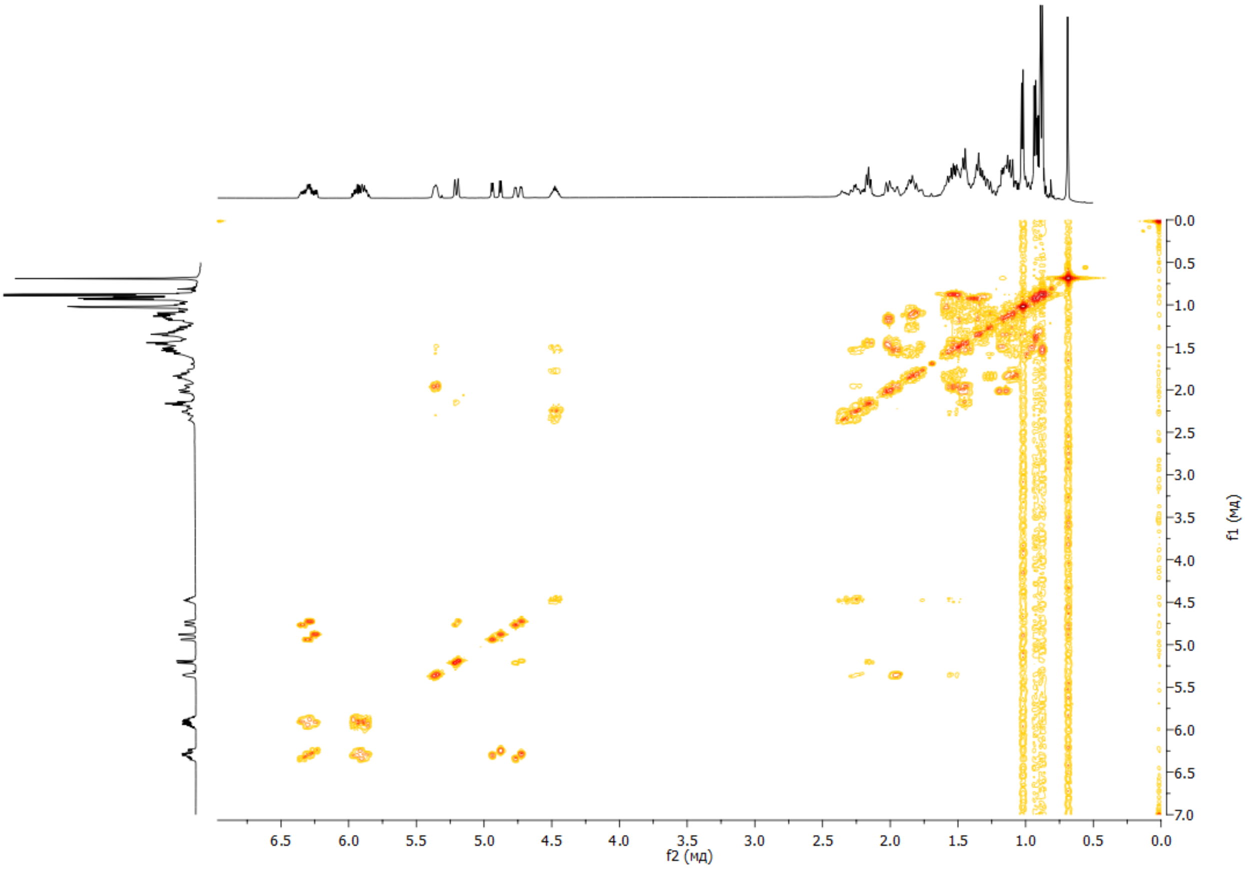

Figure S17. HSQC spectrum of compound **4a** (500 MHz, $CDCl_3$)

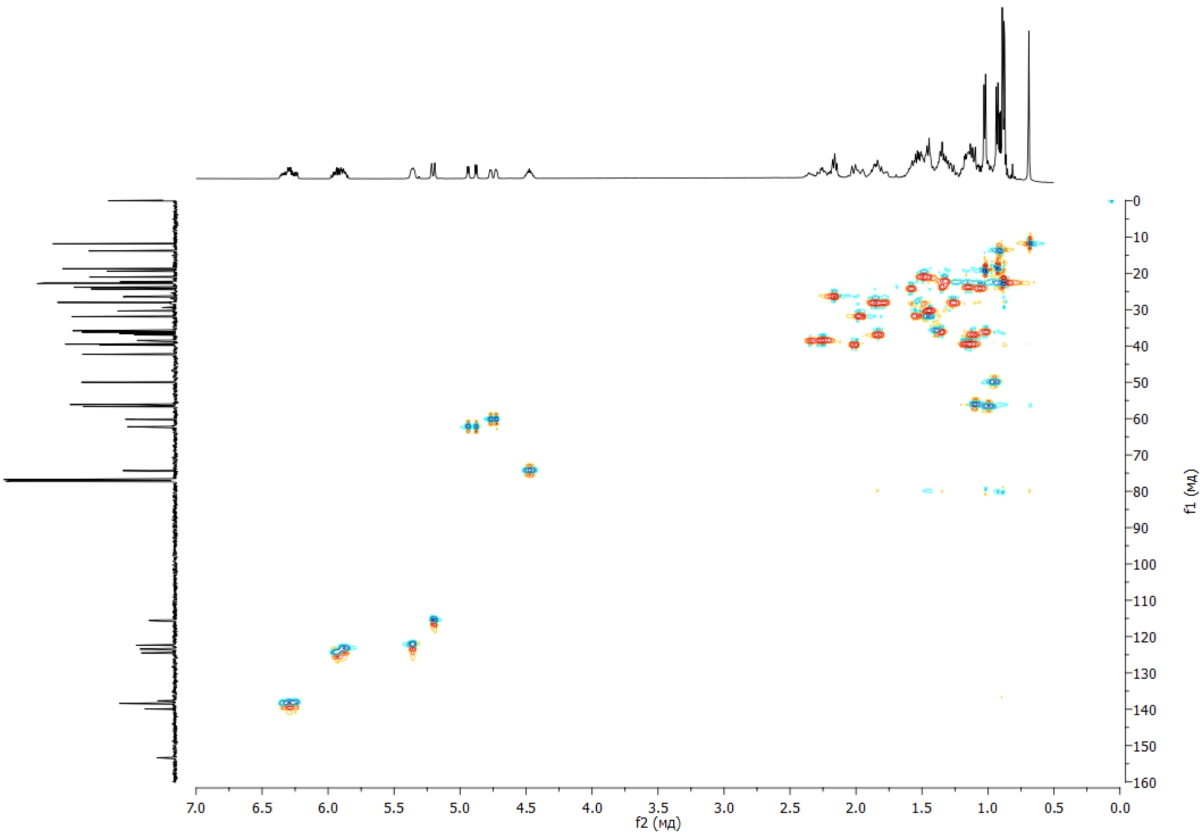

Figure S18. HMBC spectrum of compound **4a** (500 MHz, $CDCl_3$)

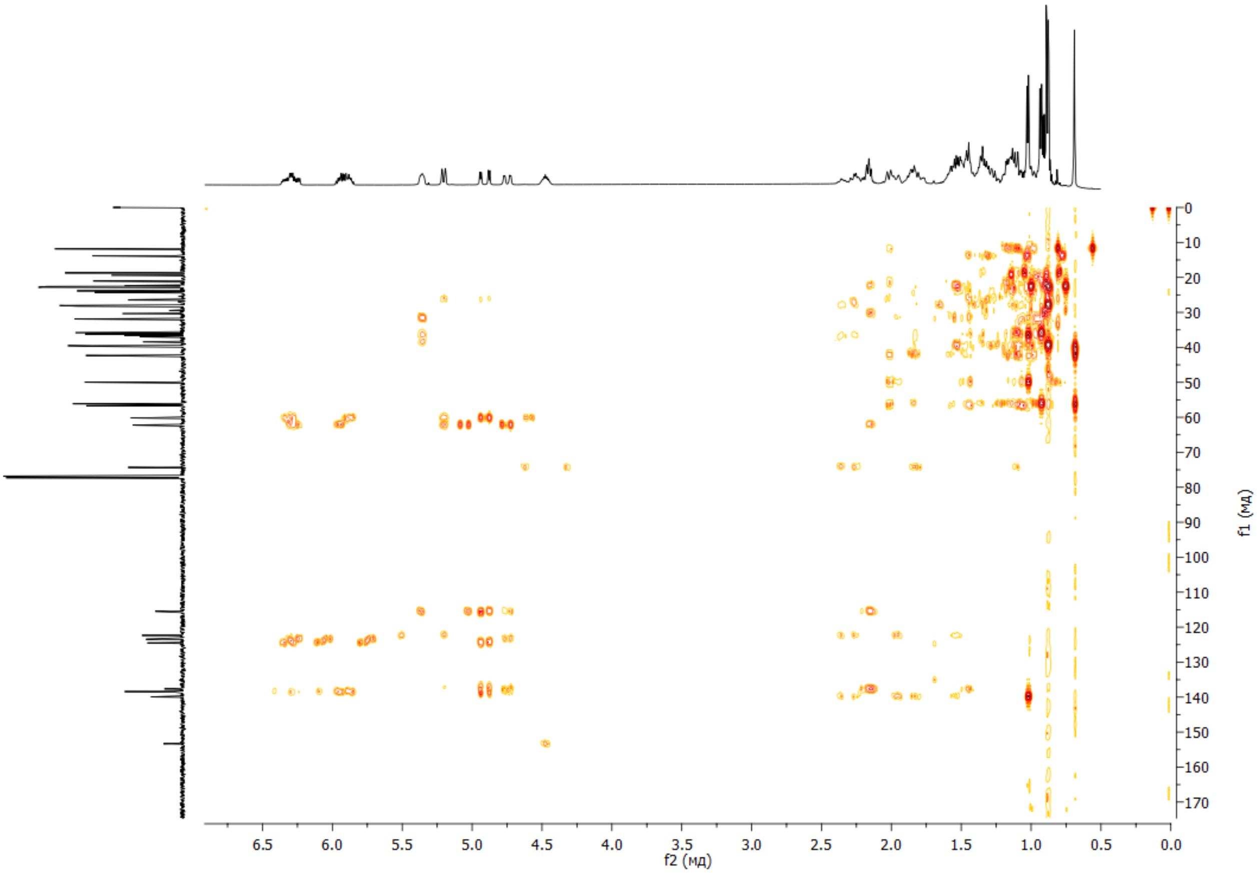

Figure S19. ^{13}C NMR Spectrum of compound **4b** (125 MHz, $CDCl_3$)

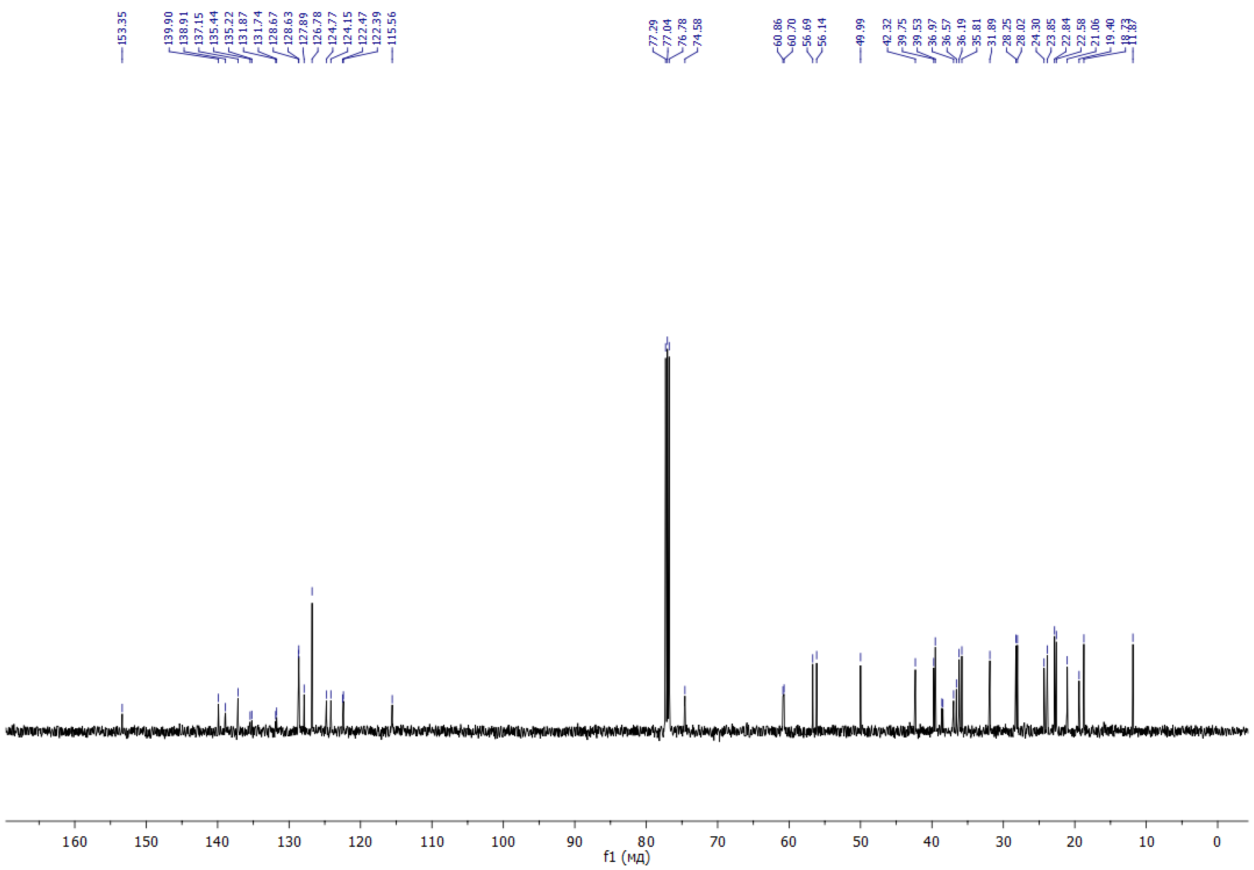

Figure S20. ^{1}H NMR Spectrum of compound **4b** (500 MHz, $CDCl_3$)

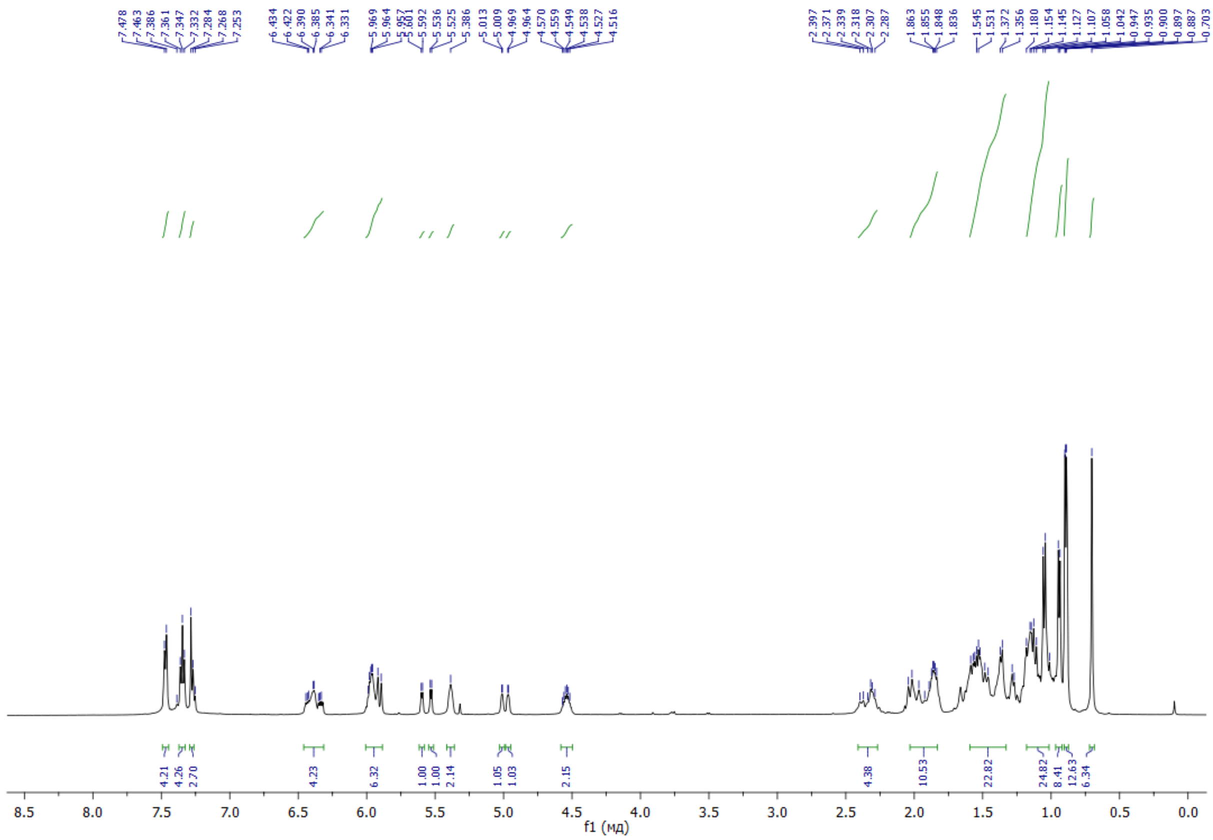

Figure S21. NOESY Spectrum of compound **4b** (500 MHz, $CDCl_3$)

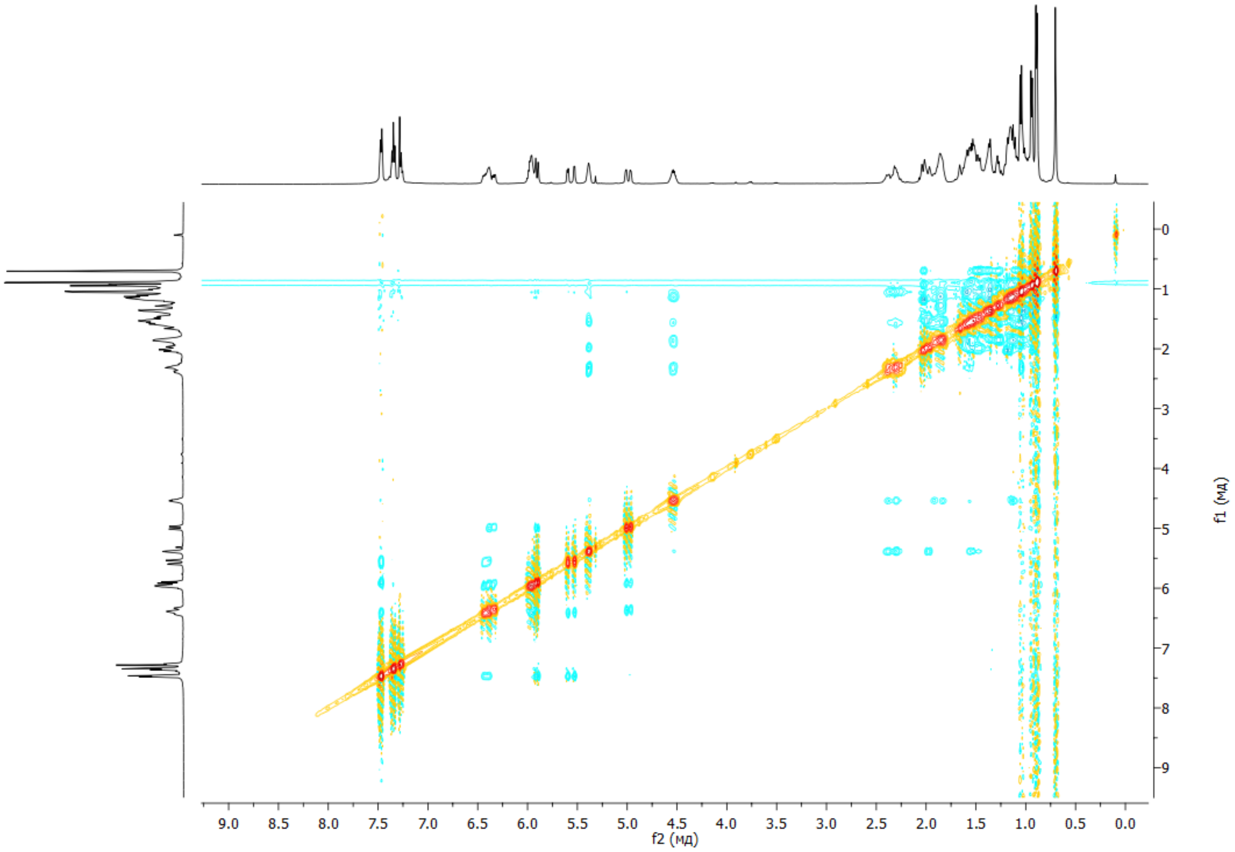

Figure S22. COSY Spectrum of compound **4b** (500 MHz, $CDCl_3$)

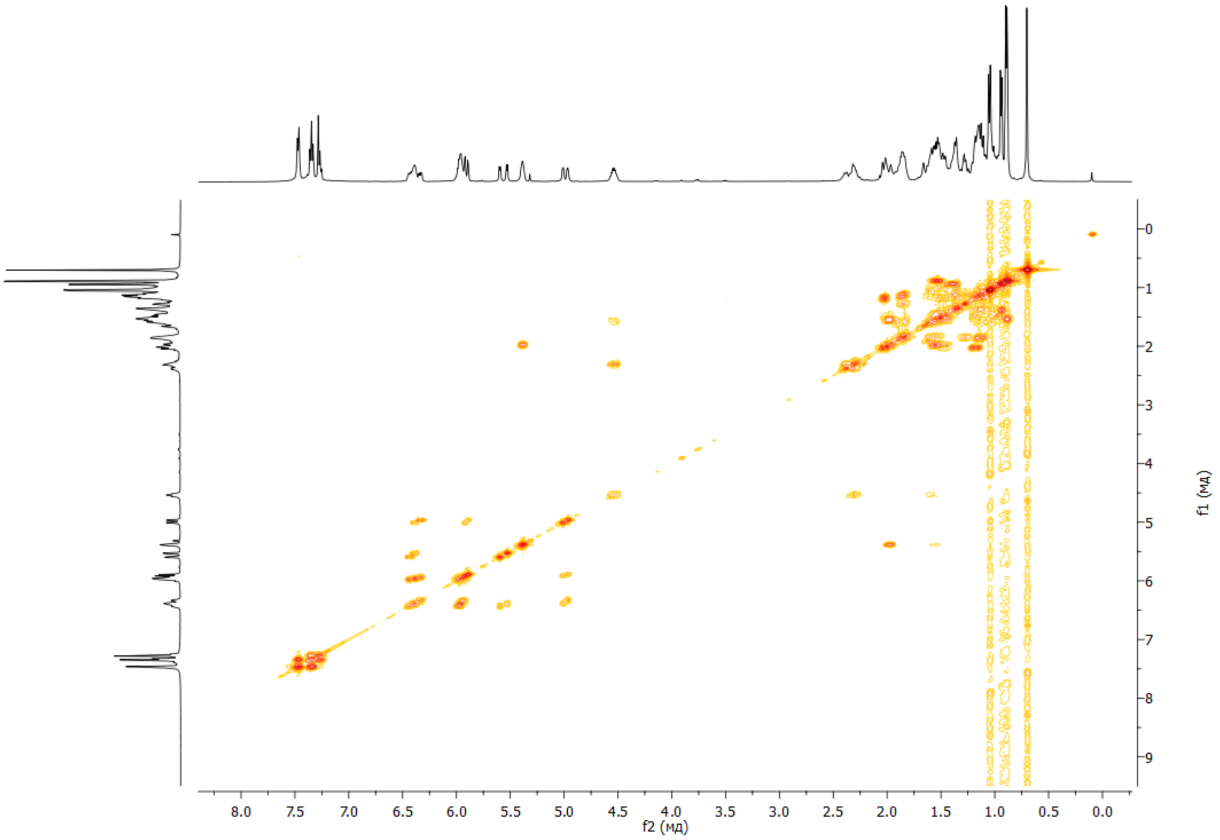

Figure S23. HSQC spectrum of compound **4b** (500 MHz, $CDCl_3$)

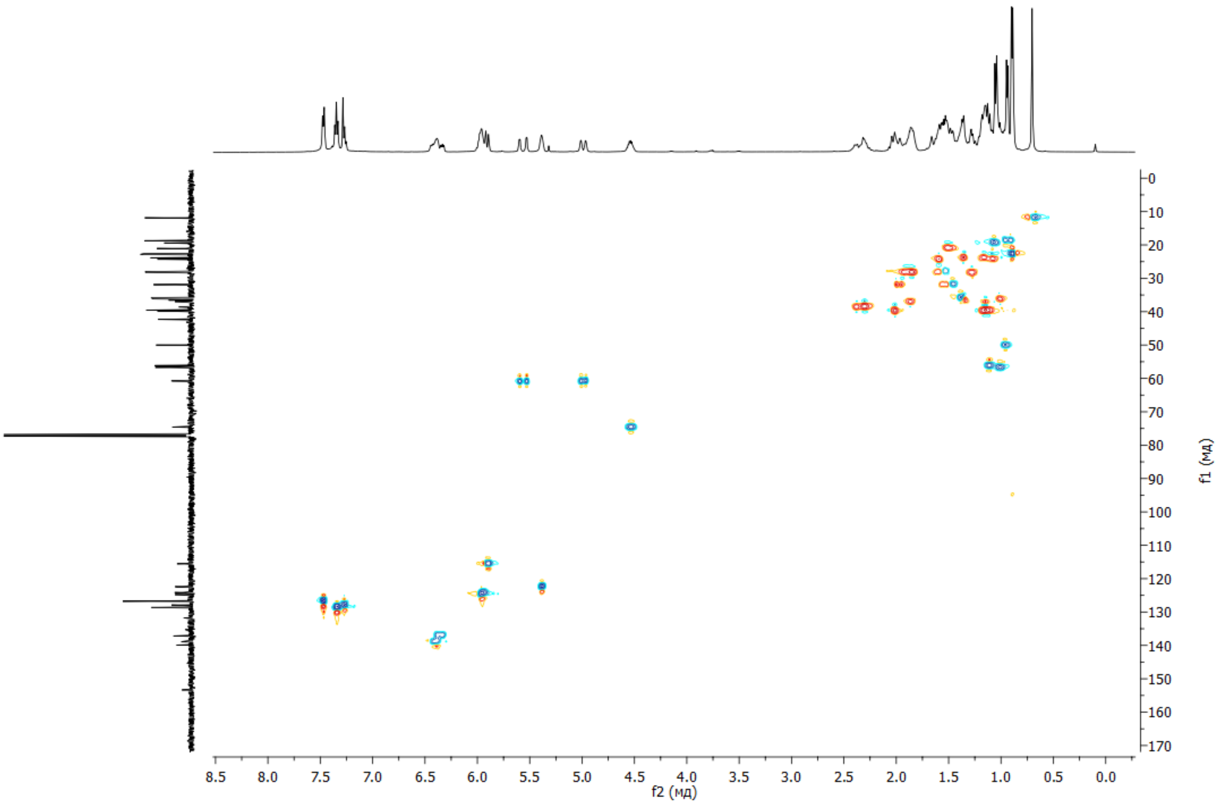

Figure S24. HMBC spectrum of compound **4b** (500 MHz, $CDCl_3$)

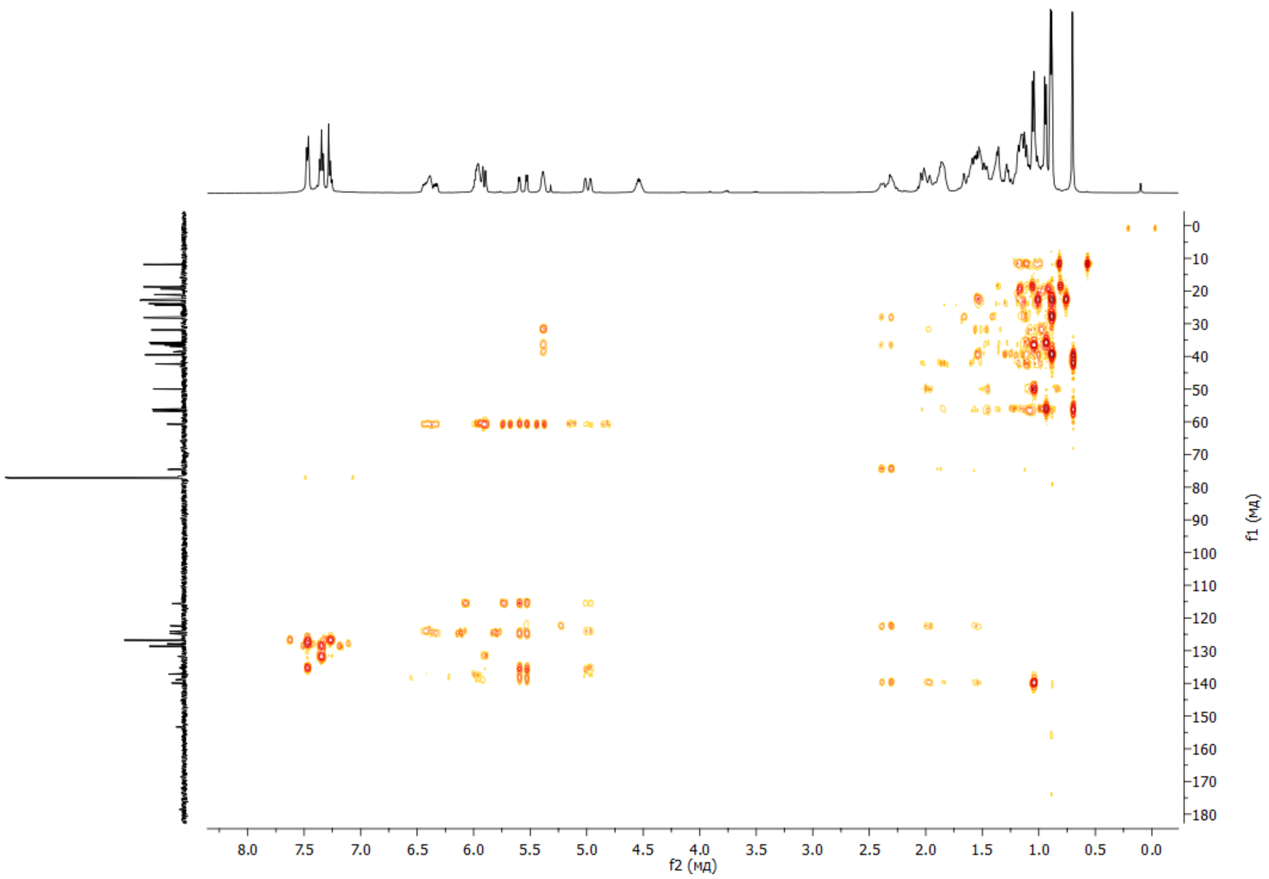

Figure S25. ^{13}C NMR Spectrum of compound **4c** (125 MHz, $CDCl_3$)

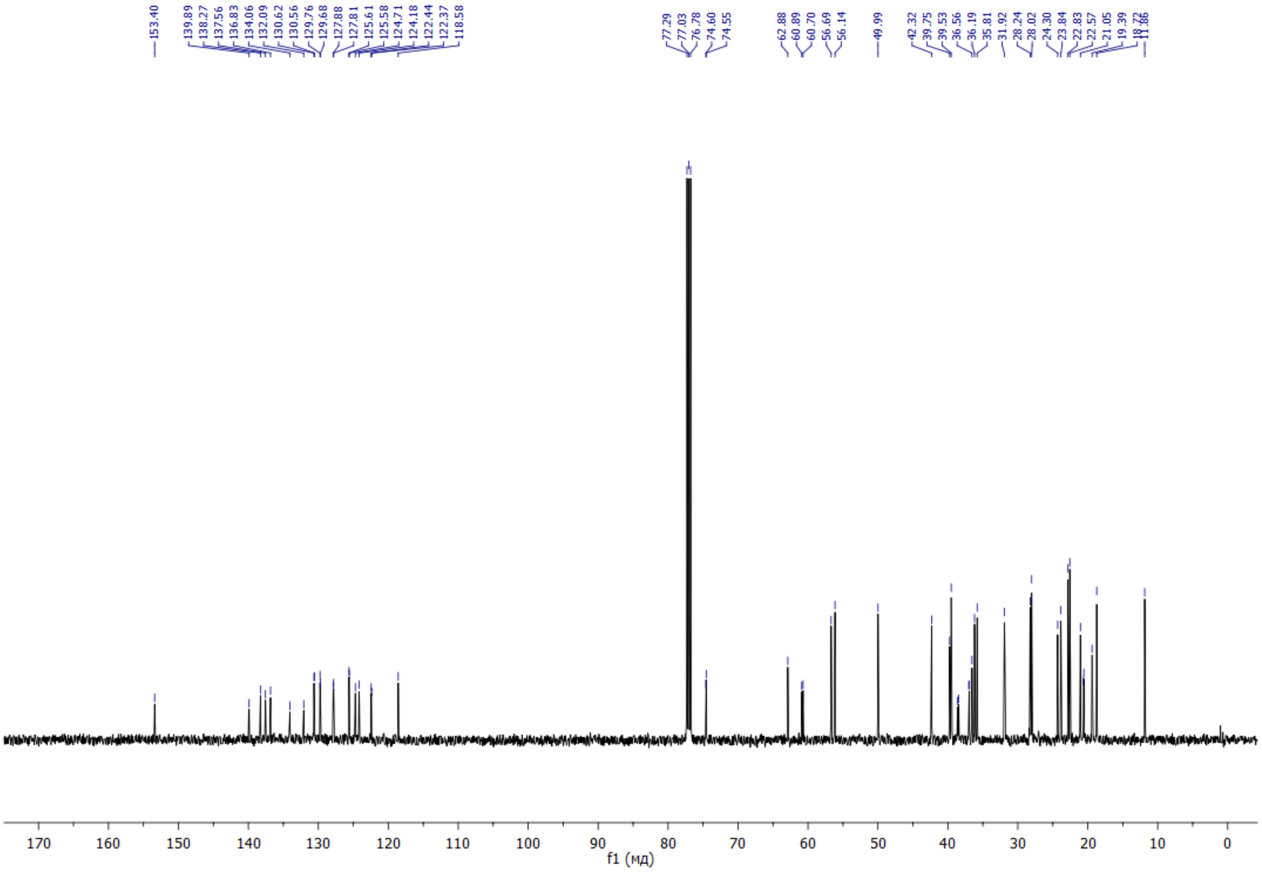

Figure S26. ^{1}H NMR Spectrum of compound **4c** (500 MHz, $CDCl_3$)

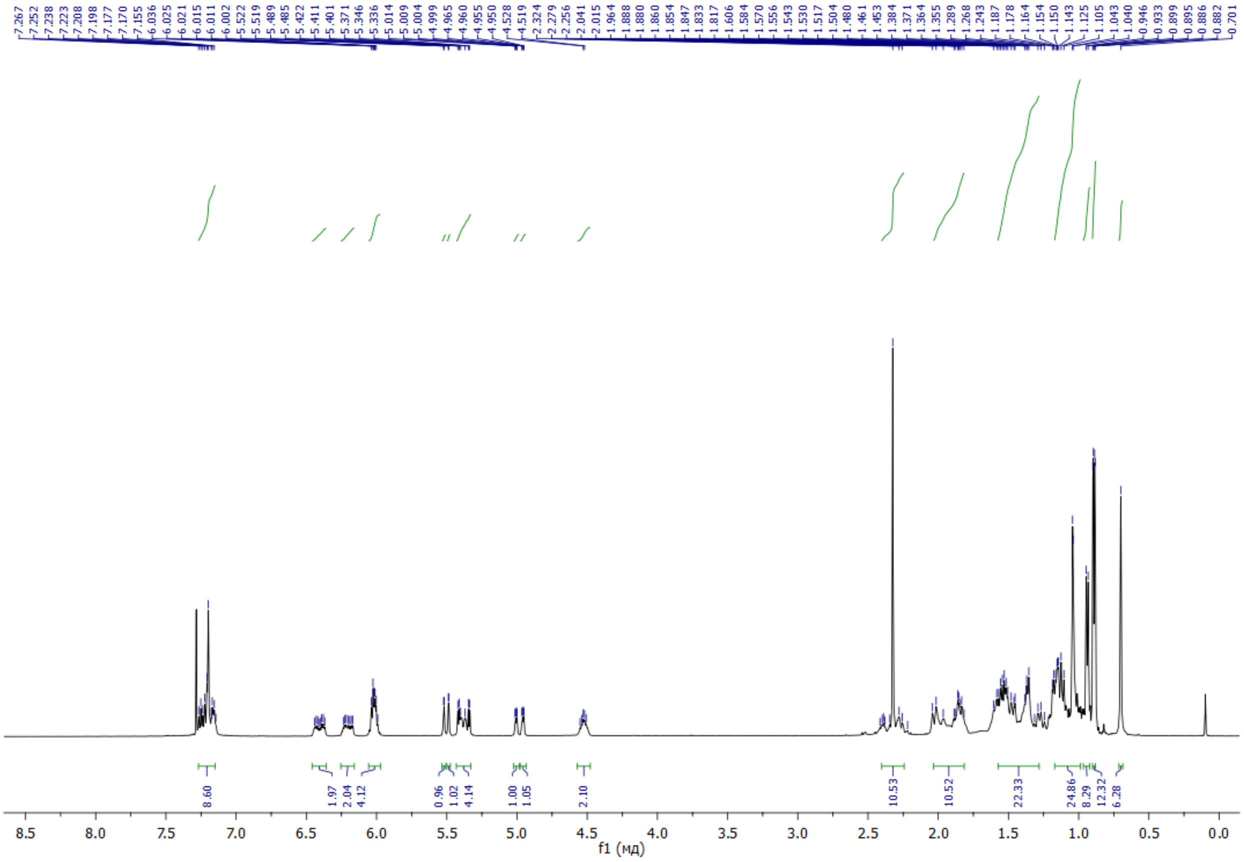

Figure S27. COSY Spectrum of compound **4c** (500 MHz, $CDCl_3$)

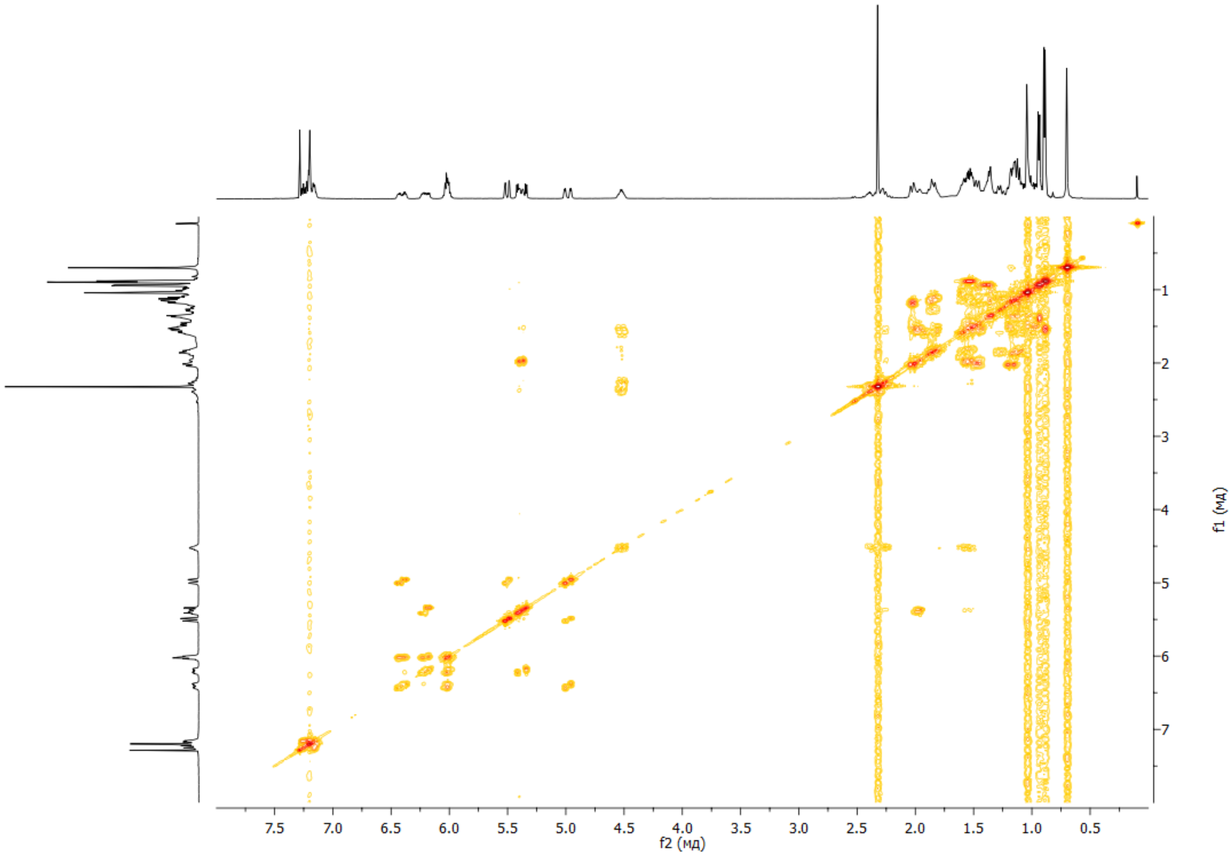

Figure S28. HSQC spectrum of compound **4c** (500 MHz, $CDCl_3$)

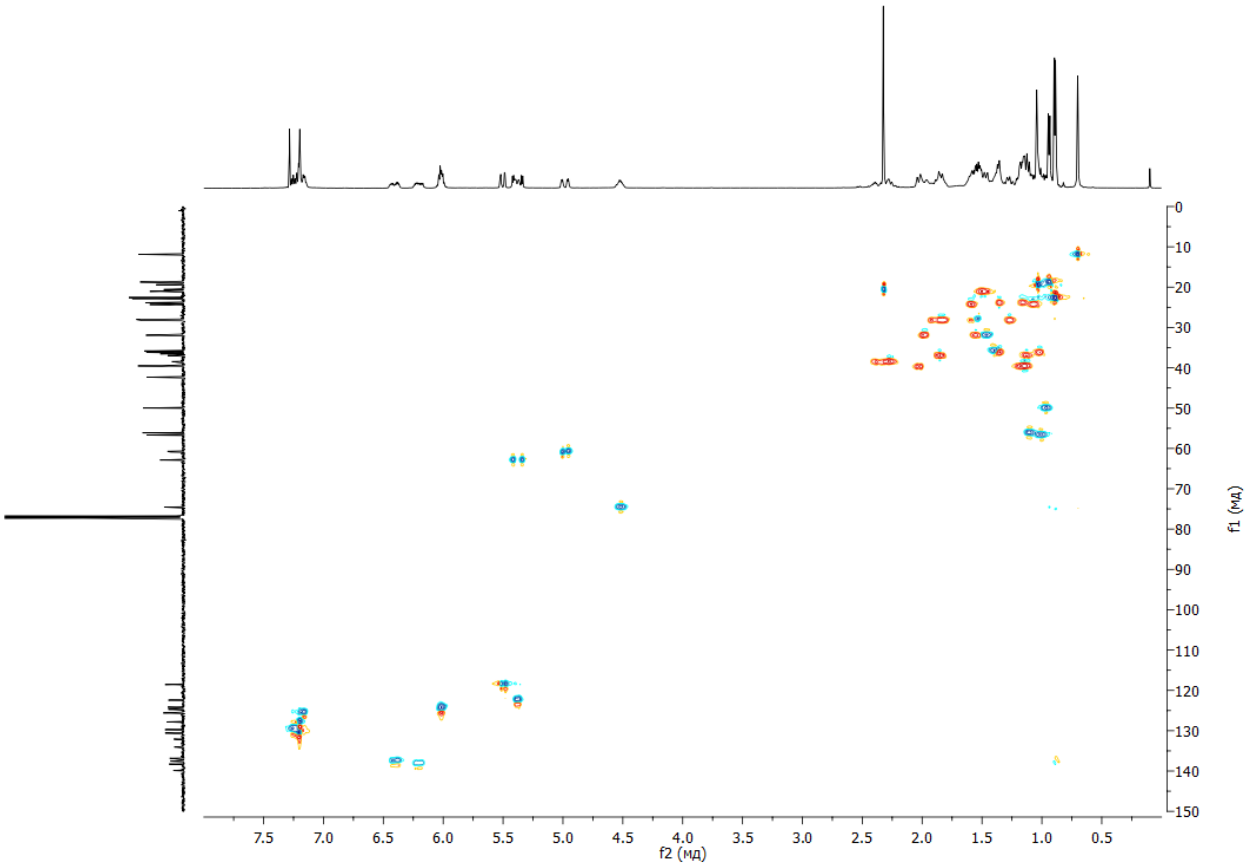

Figure S29. HMBC spectrum of compound **4c** (500 MHz, $CDCl_3$)

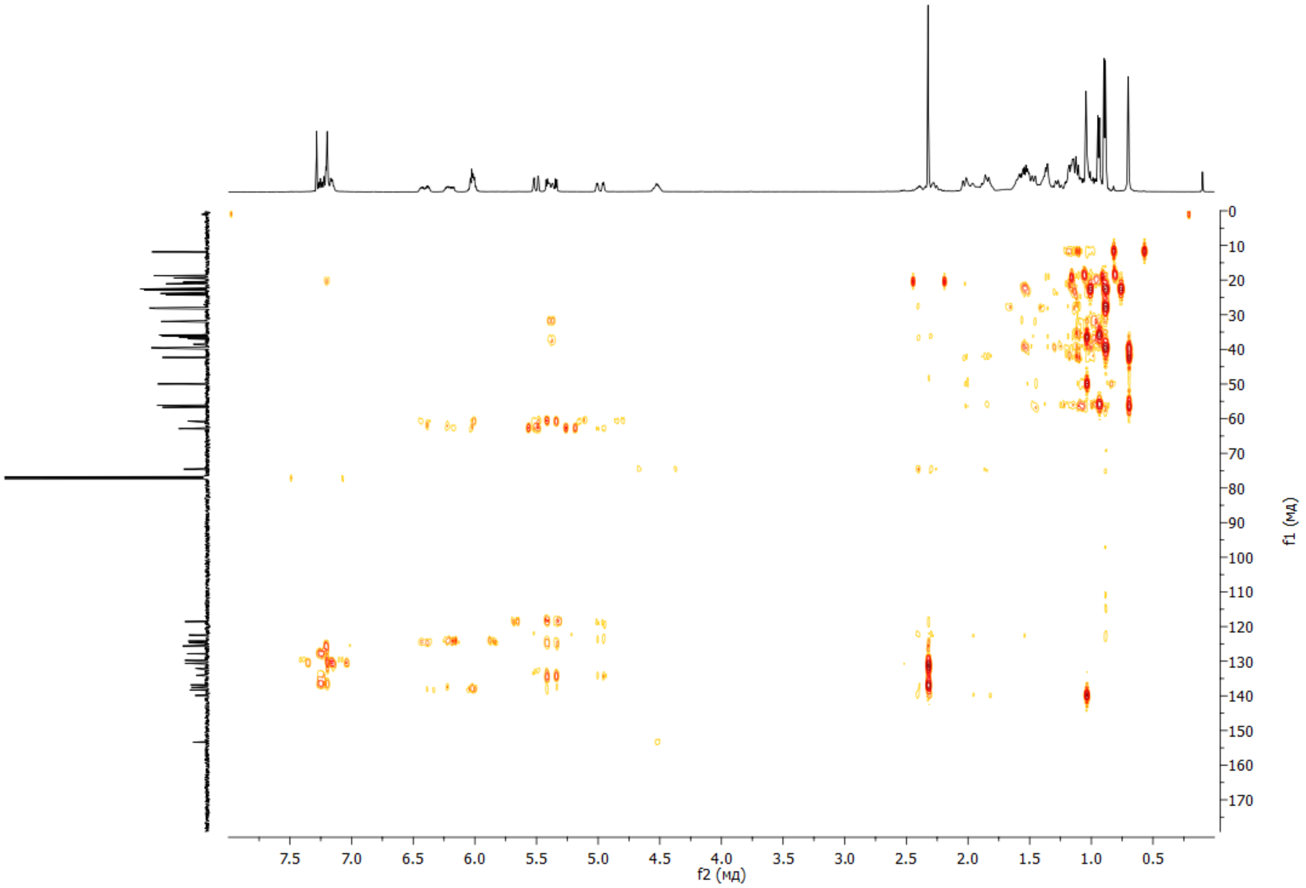

Figure S30. ^{13}C NMR Spectrum of compound **4d** (125 MHz, $CDCl_3$)

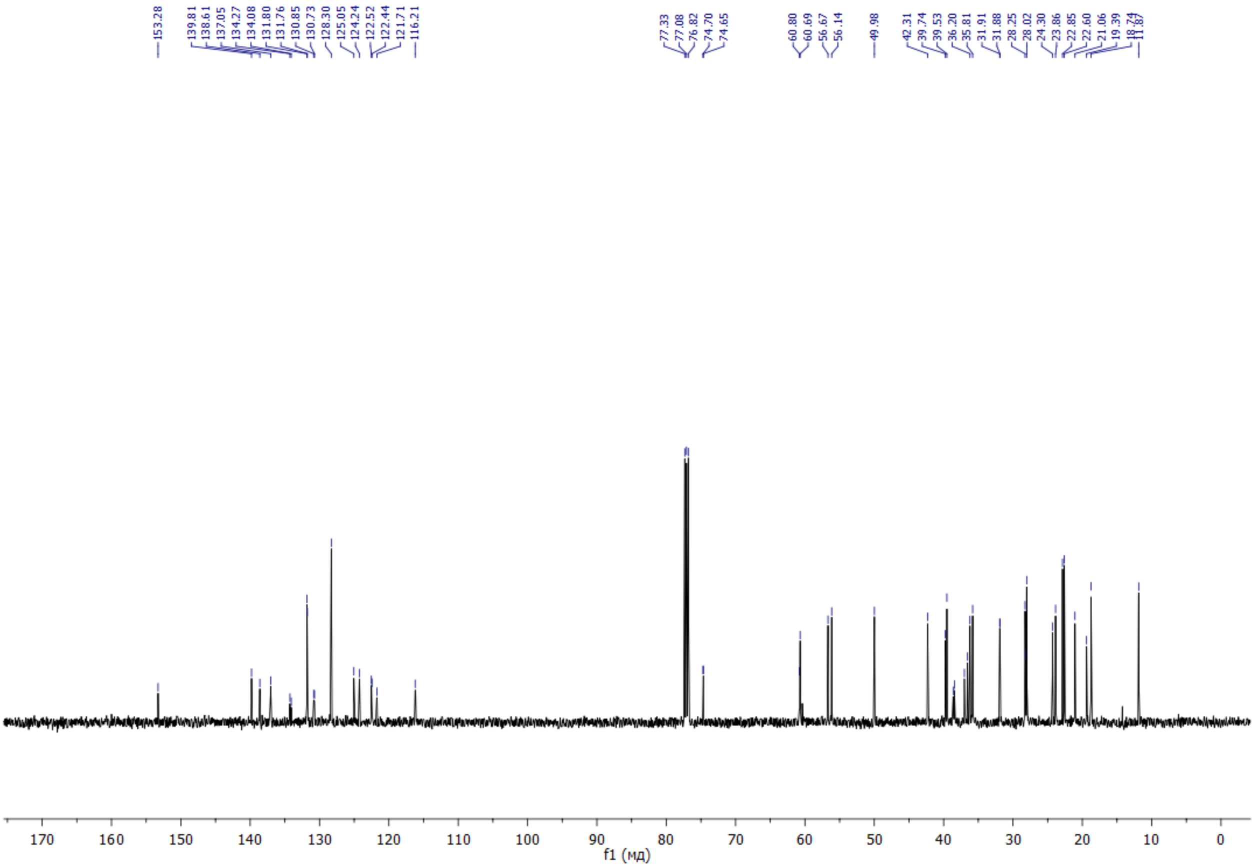

Figure S31. ^{1}H NMR Spectrum of compound **4d** (500 MHz, $CDCl_3$)

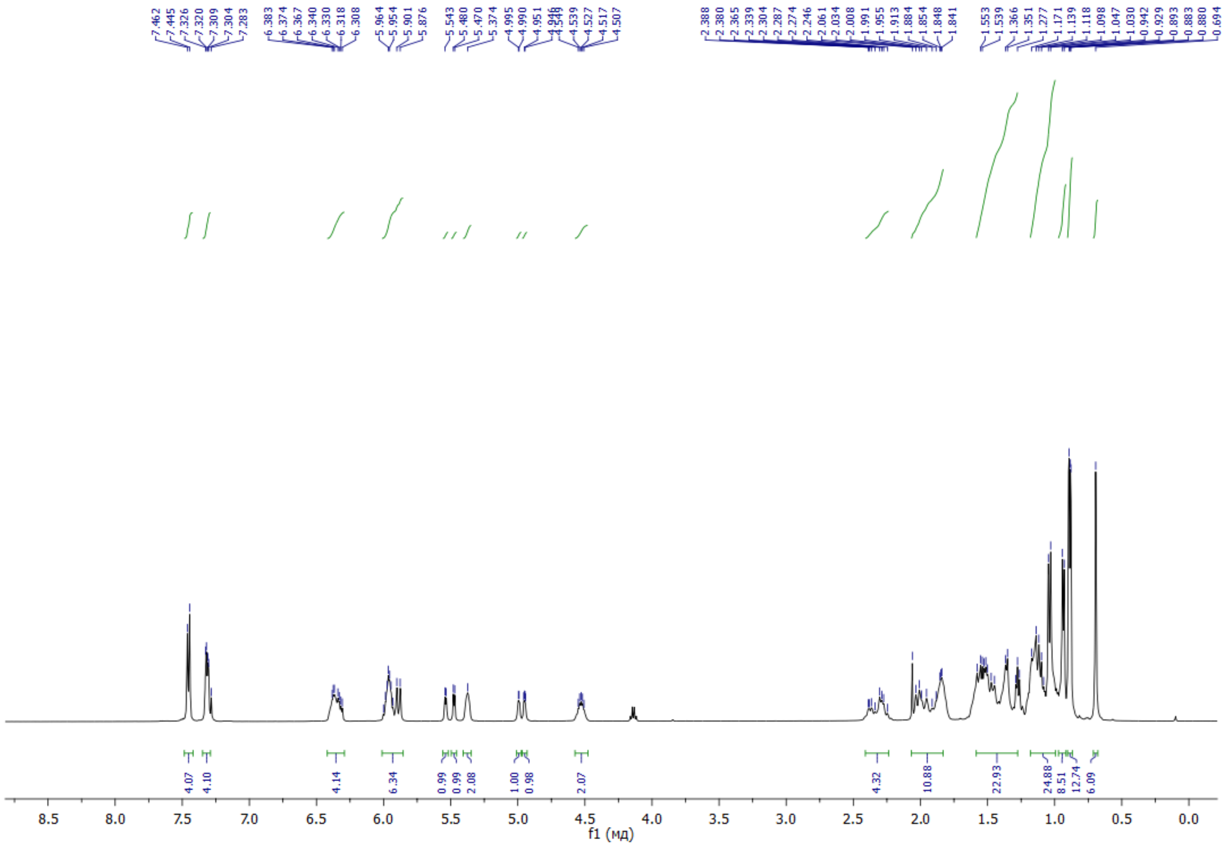

Figure S32. COSY Spectrum of compound **4d** (500 MHz, $CDCl_3$)

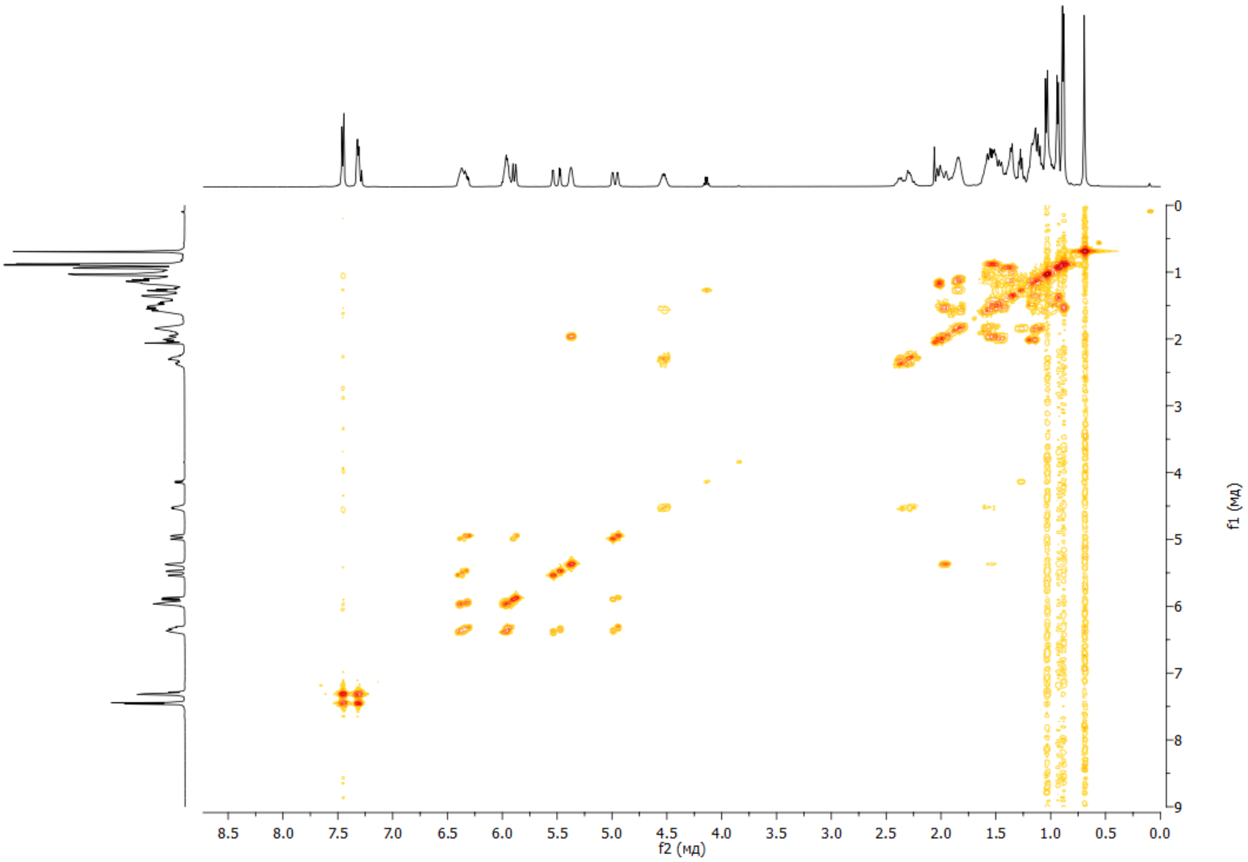

Figure S33. HSQC spectrum of compound **4d** (500 MHz, $CDCl_3$)

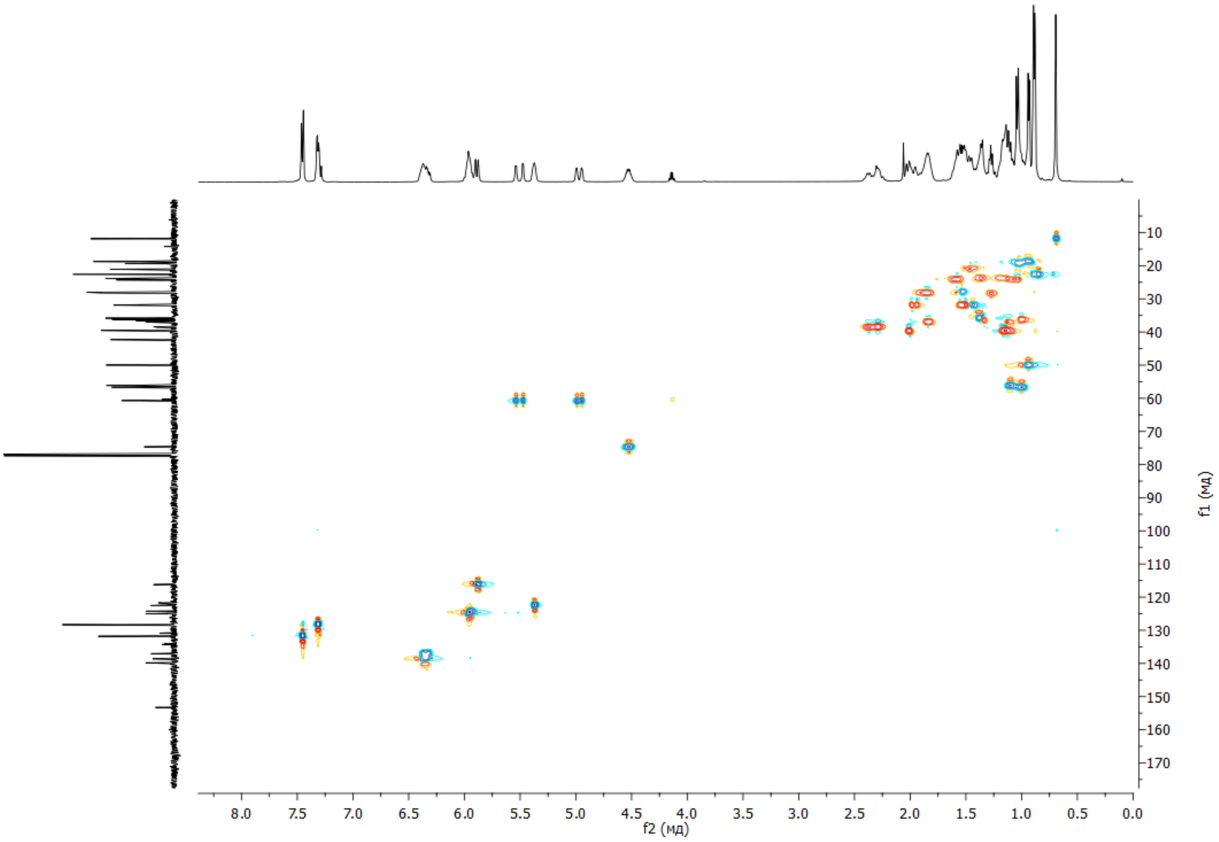

Figure S34. HMBC spectrum of compound **4d** (500 MHz, $CDCl_3$)

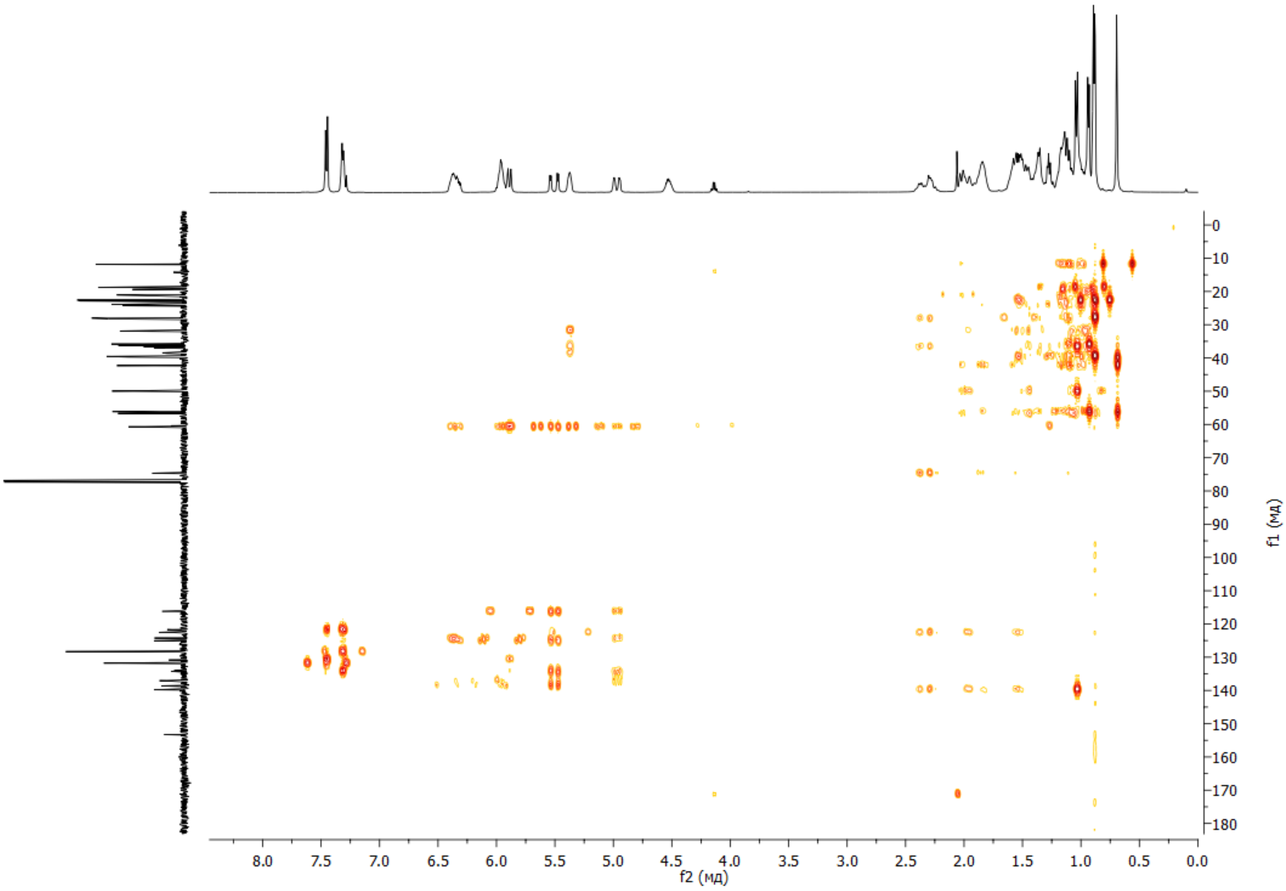

Figure S35. ^{13}C NMR Spectrum of compound **4e** (125 MHz, $CDCl_3$)

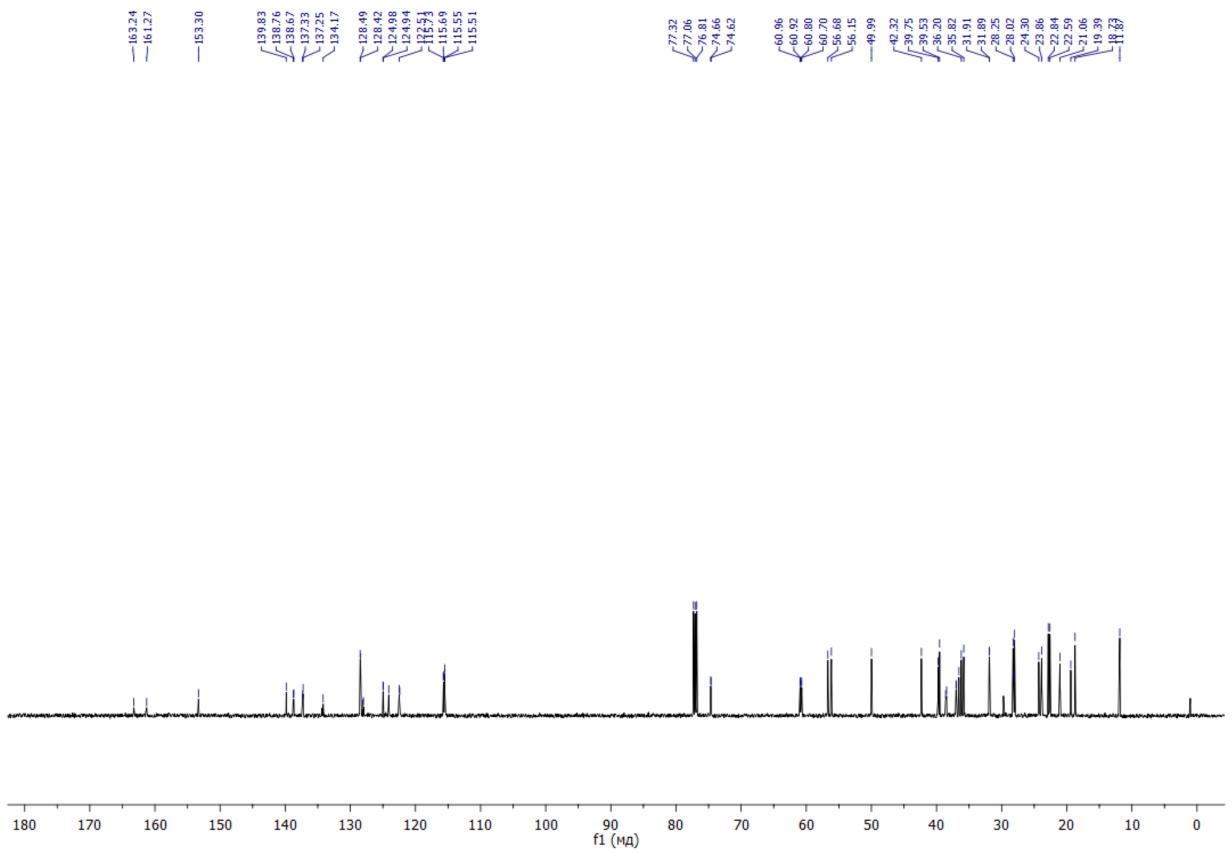

Figure S36. ^{1}H NMR Spectrum of compound **4e** (500 MHz, $CDCl_3$)

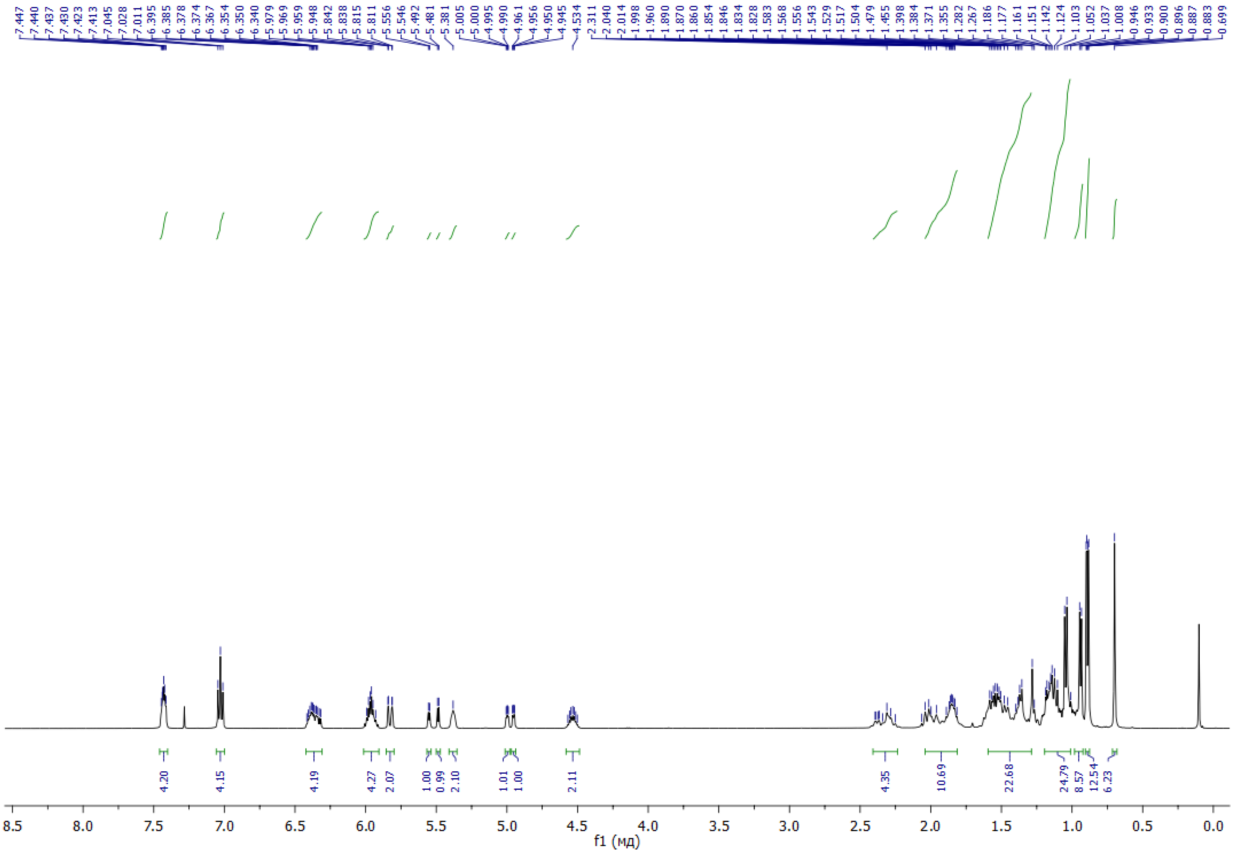

Figure S37. NOESY Spectrum of compound **4e** (500 MHz, $CDCl_3$)

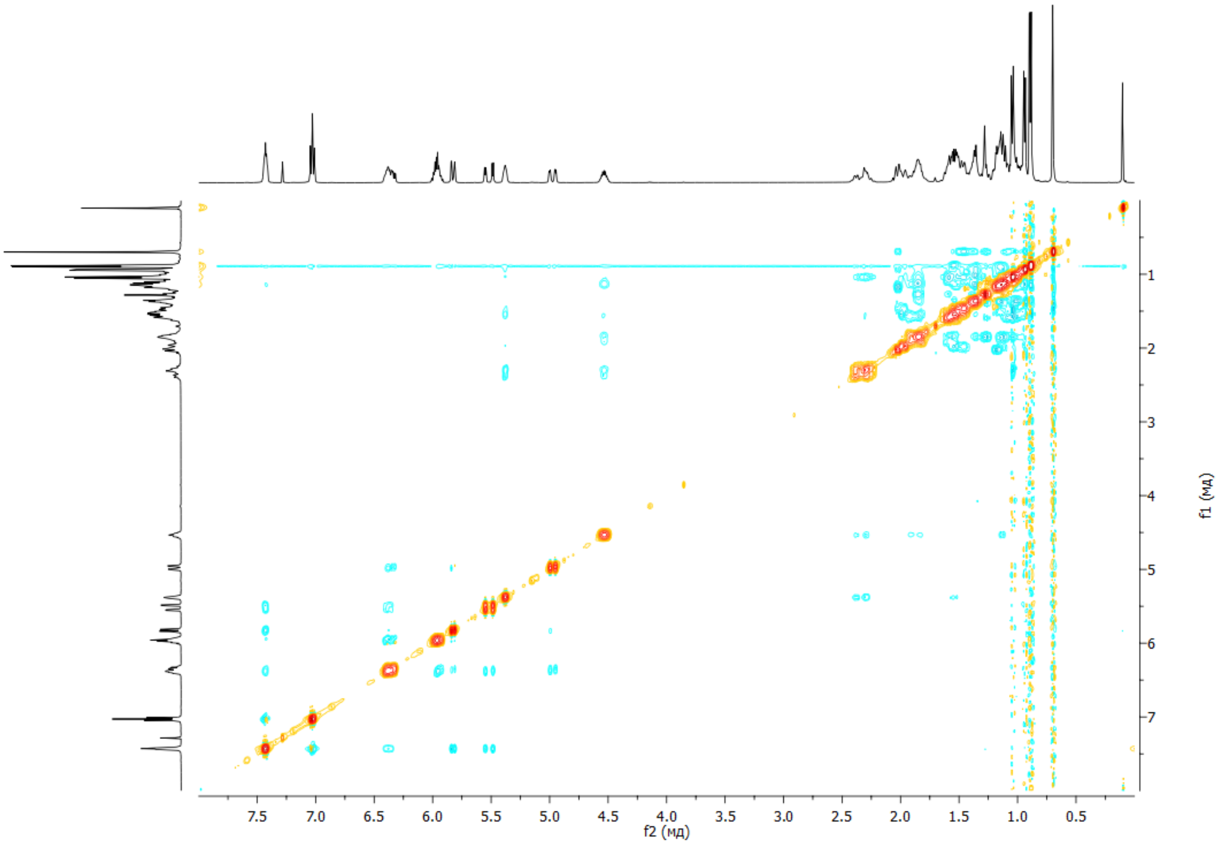

Figure S38. COSY Spectrum of compound **4e** (500 MHz, $CDCl_3$)

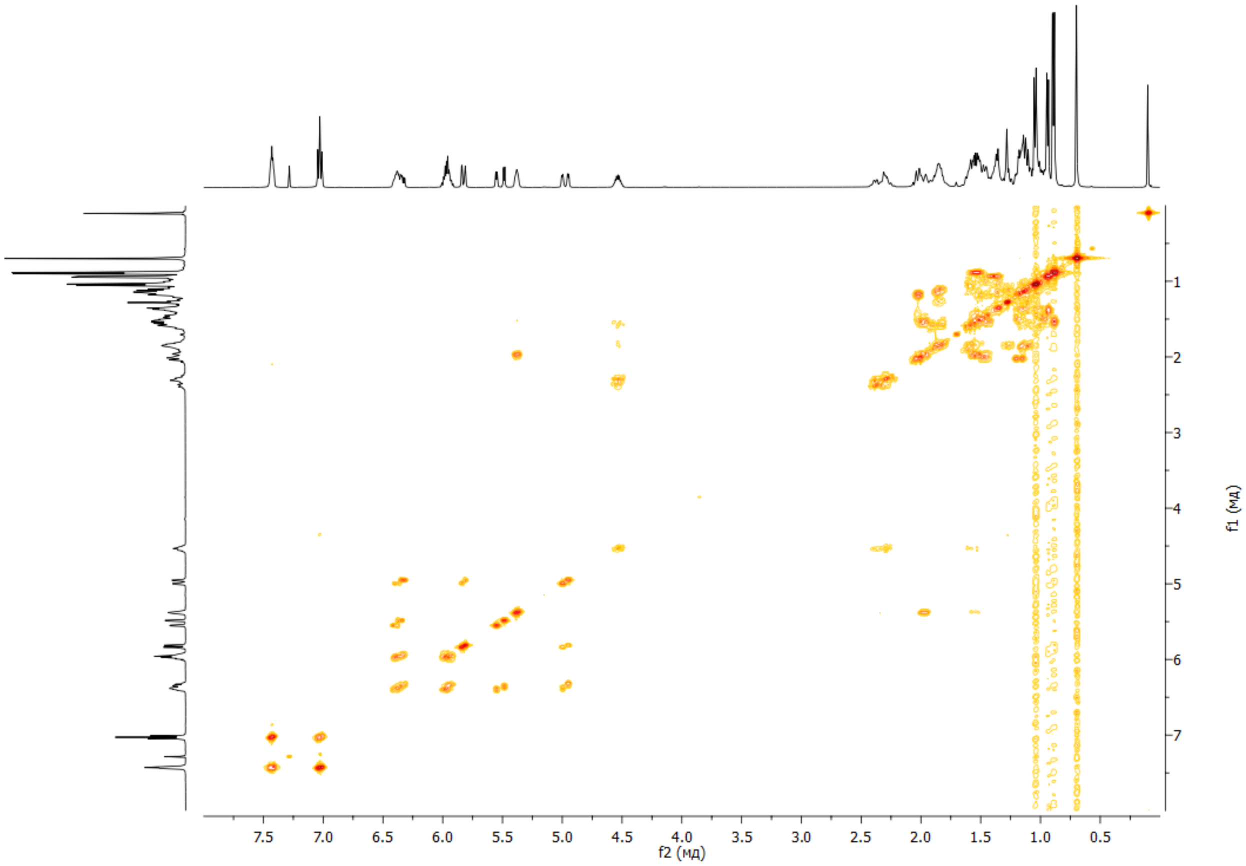

Figure S39. HSQC spectrum of compound **4e** (500 MHz, $CDCl_3$)

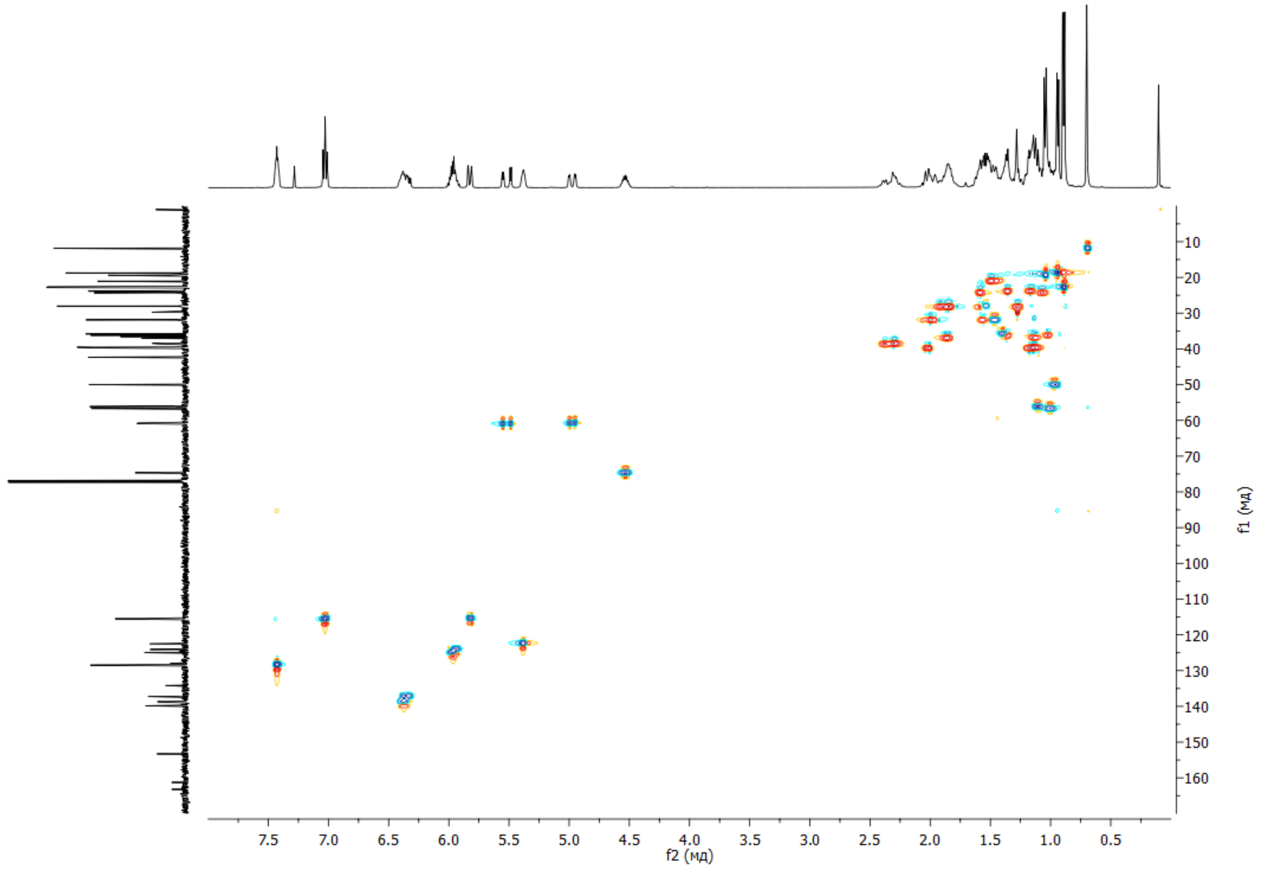

Figure S40. HMBC spectrum of compound **4e** (500 MHz, $CDCl_3$)

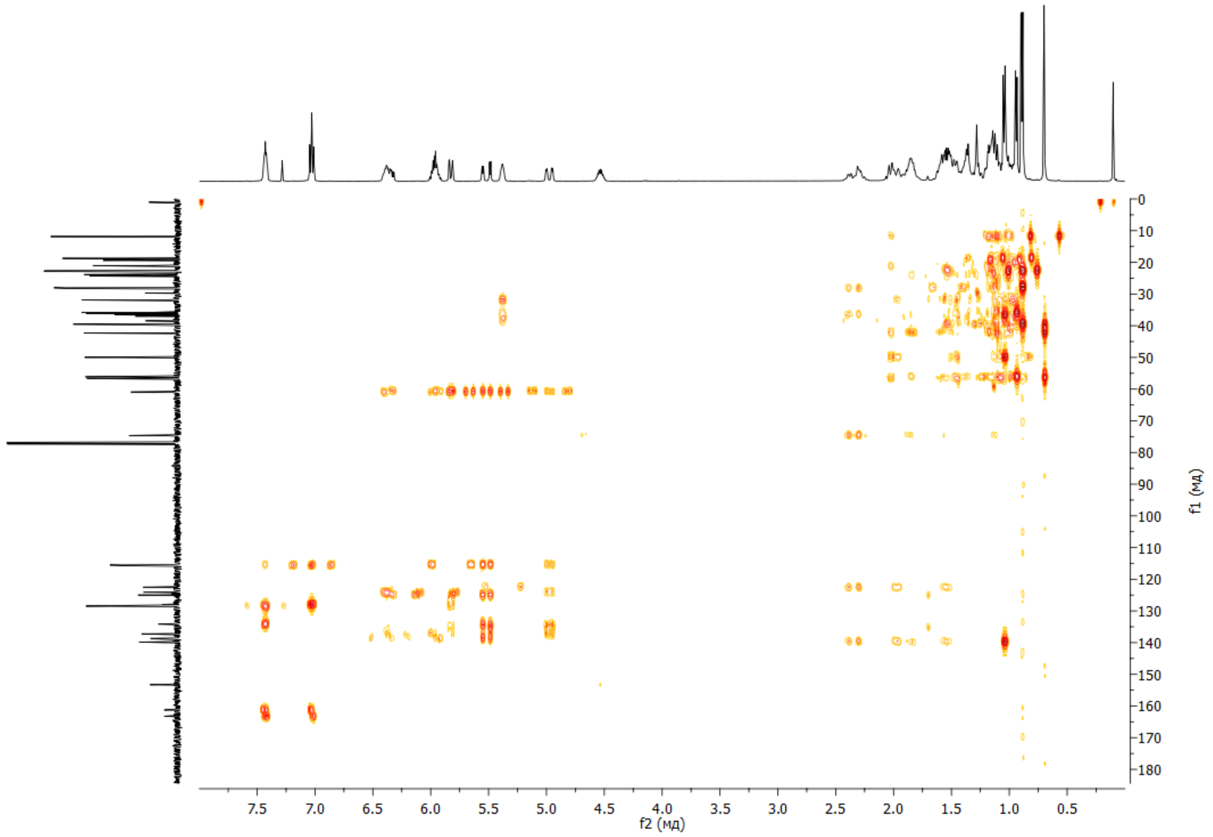

Figure S41. ^{13}C NMR Spectrum of compound **4f** (125 MHz, $CDCl_3$)

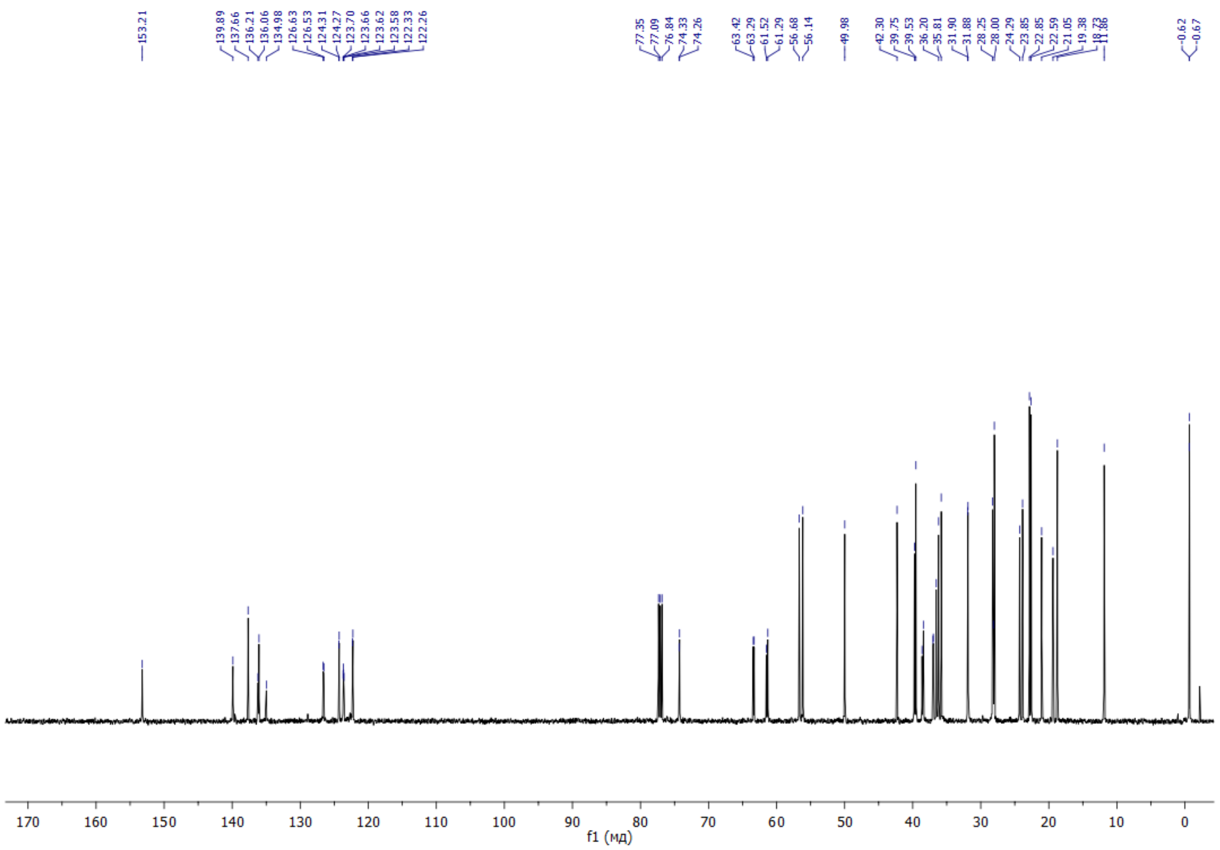

Figure S42. ^{1}H NMR Spectrum of compound **4f** (500 MHz, $CDCl_3$)

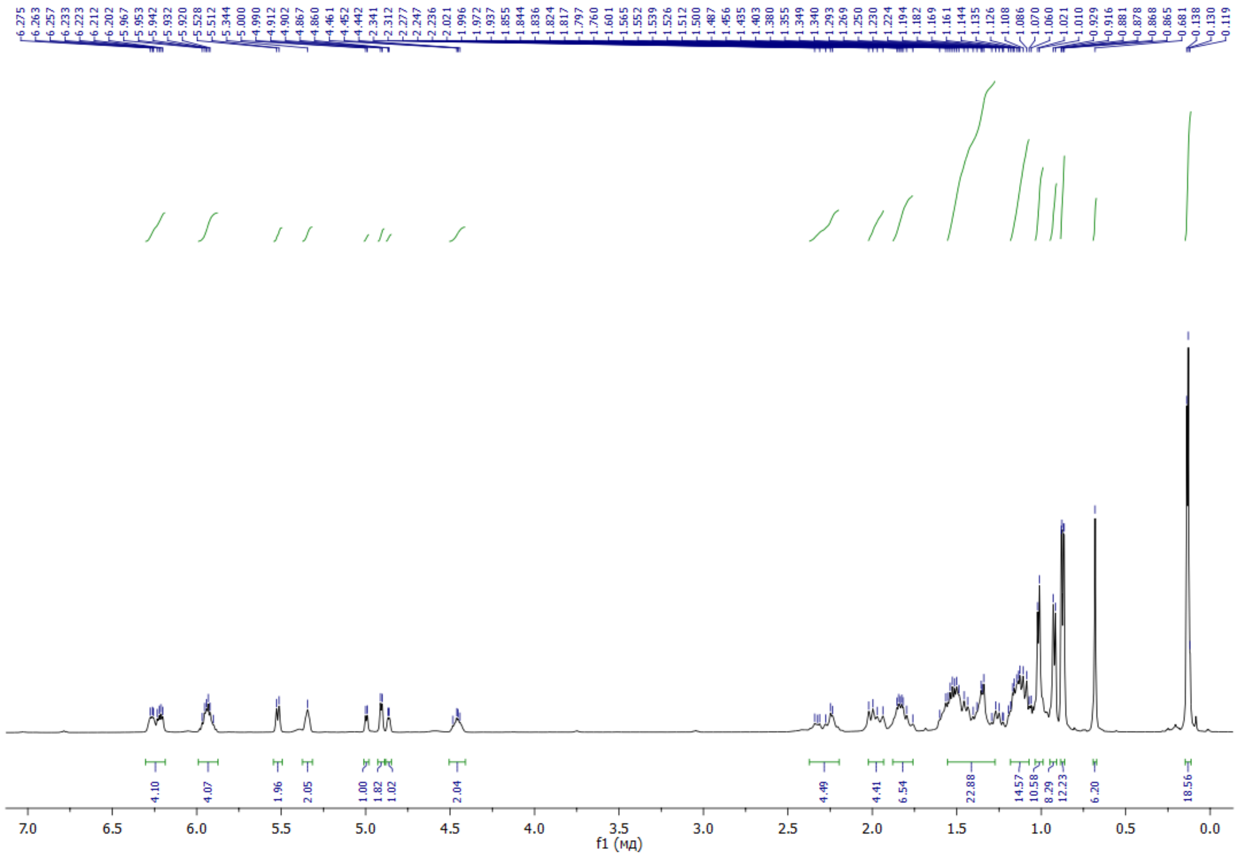

Figure S43. COSY Spectrum of compound **4f** (500 MHz, $CDCl_3$)

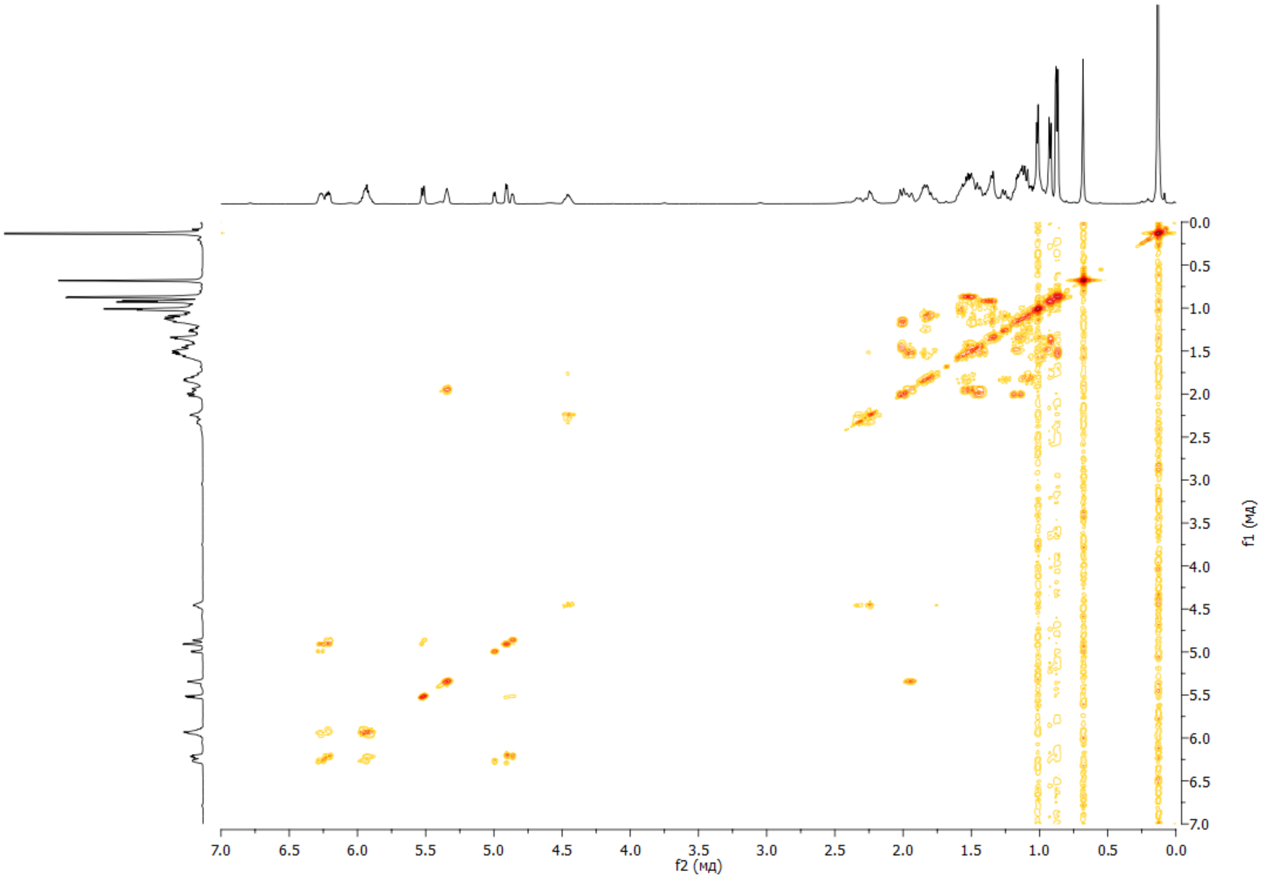

Figure S44. HSQC spectrum of compound **4f** (500 MHz, $CDCl_3$)

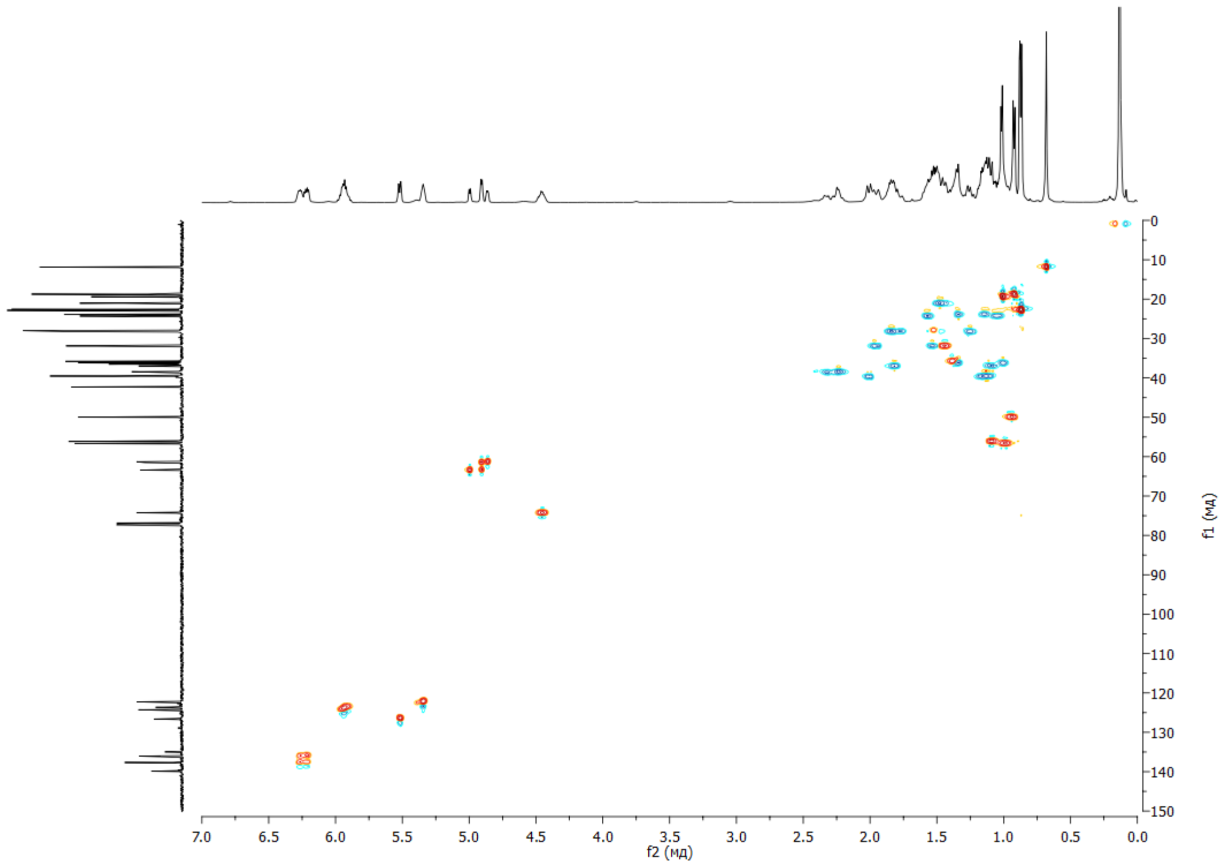

Figure S45. HMBC spectrum of compound **4f** (500 MHz, $CDCl_3$)

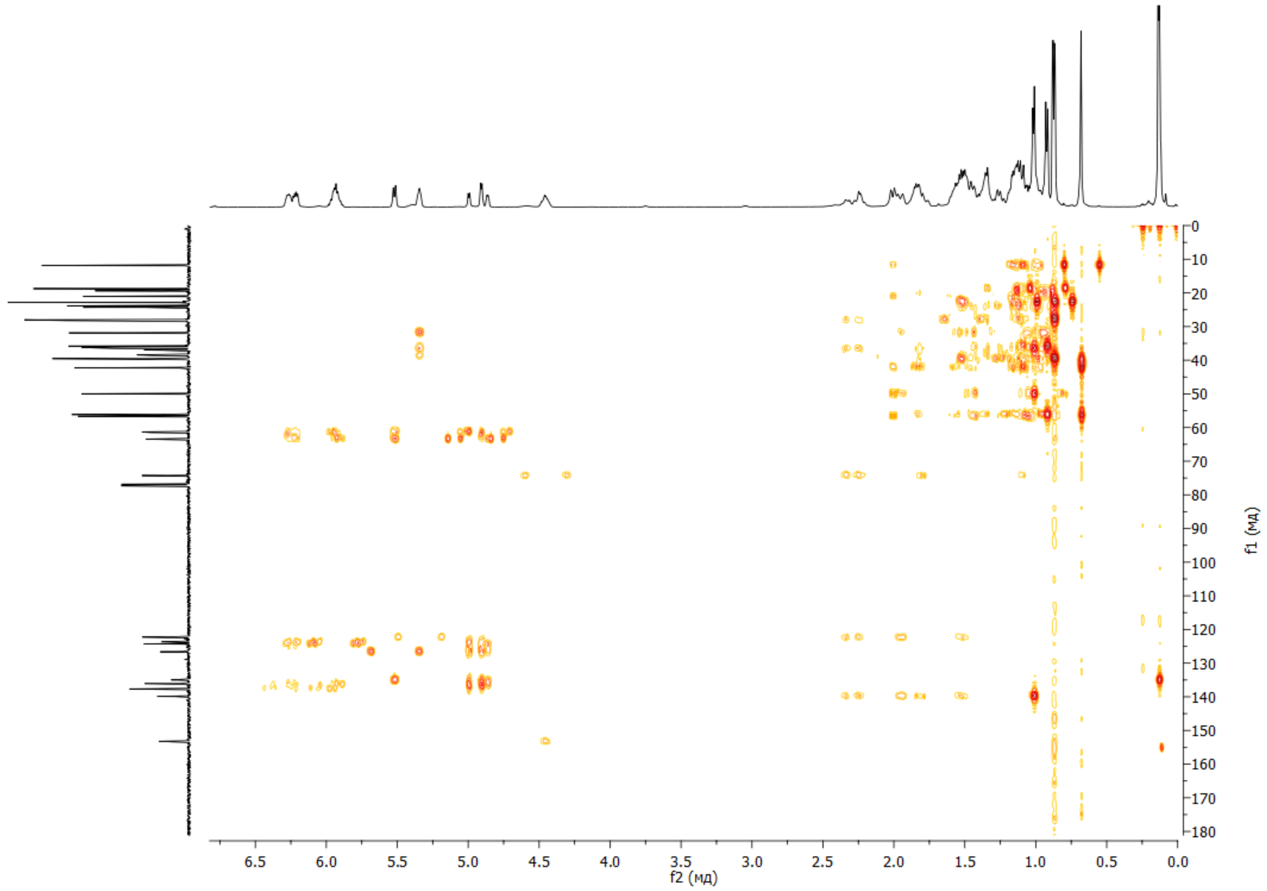

Figure S46. ^{13}C NMR Spectrum of compound **4g** (125 MHz, $CDCl_3$)

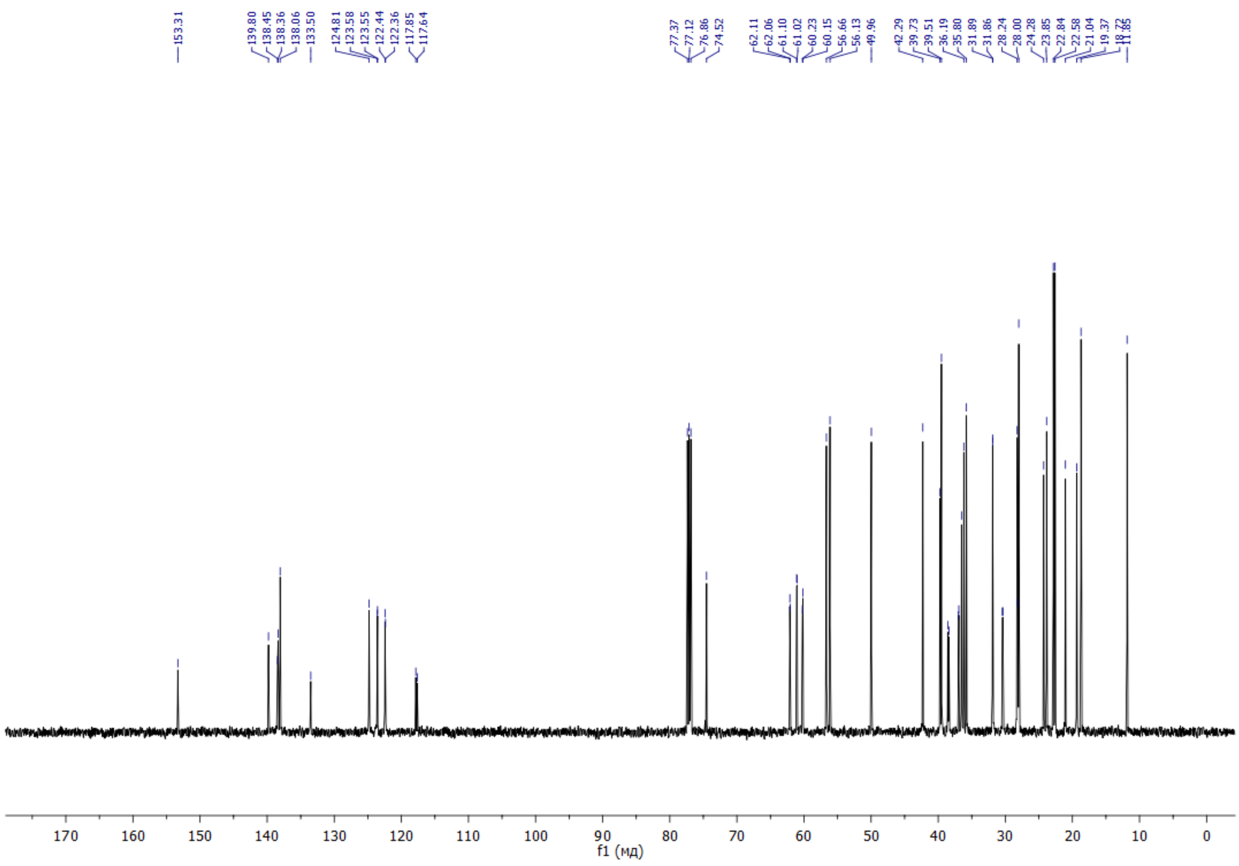

Figure S47. ^{1}H NMR Spectrum of compound **4g** (500 MHz, $CDCl_3$)

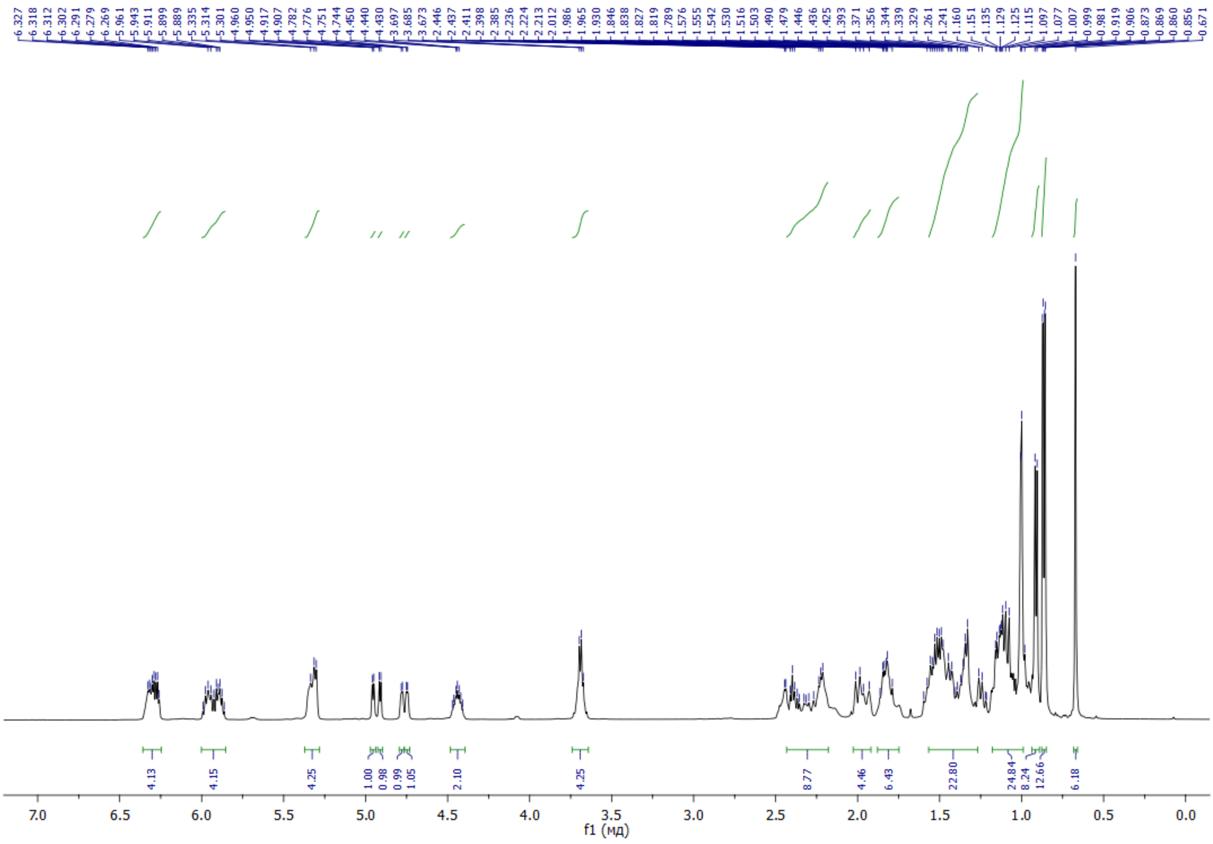

Figure S48. NOESY Spectrum of compound **4g** (500 MHz, $CDCl_3$)

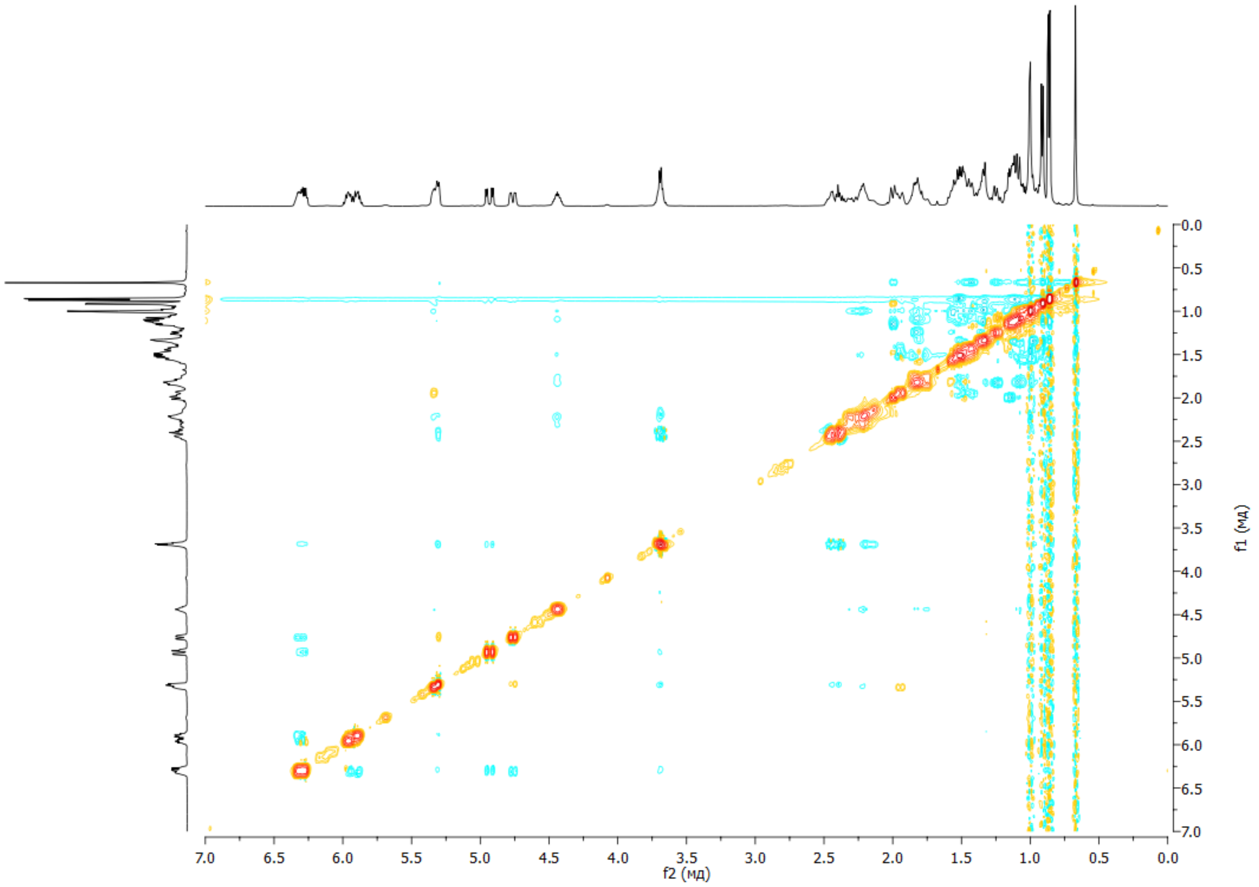

Figure S49. COSY Spectrum of compound **4g** (500 MHz, $CDCl_3$)

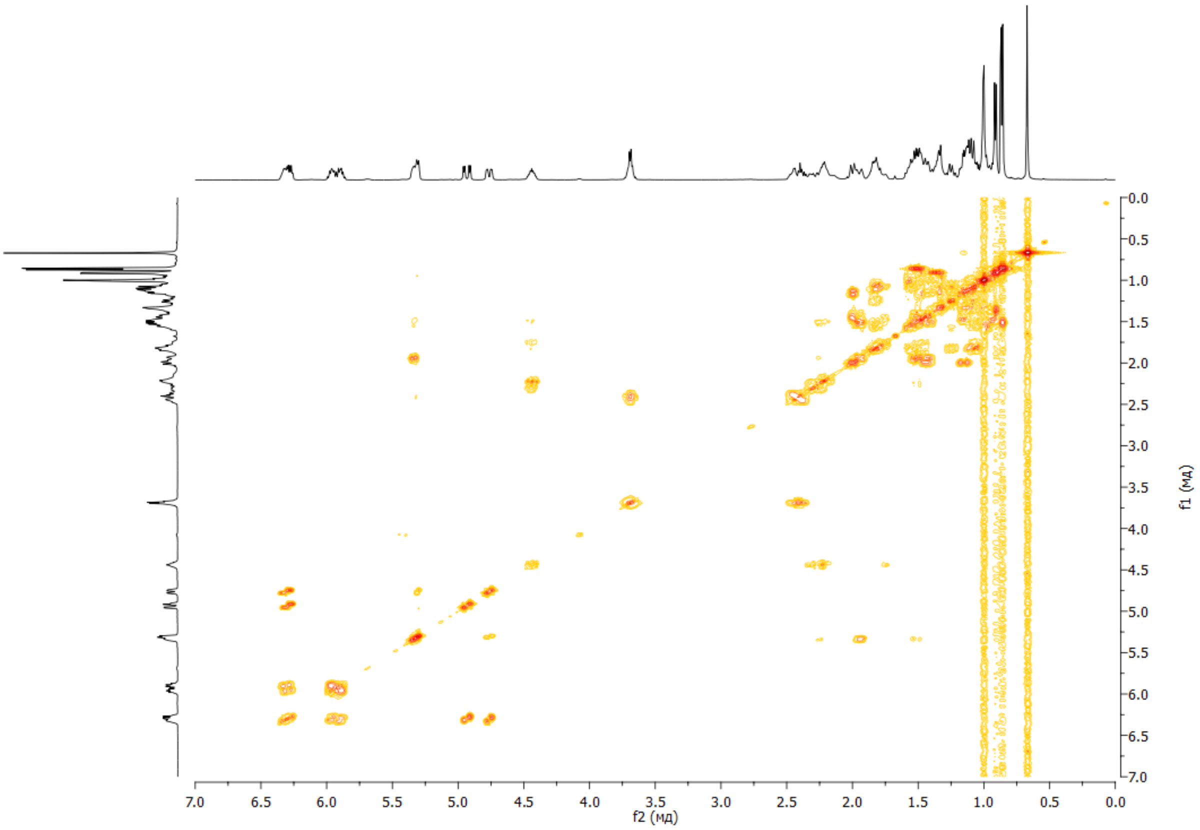

Figure S50. HSQC spectrum of compound **4g** (500 MHz, $CDCl_3$)

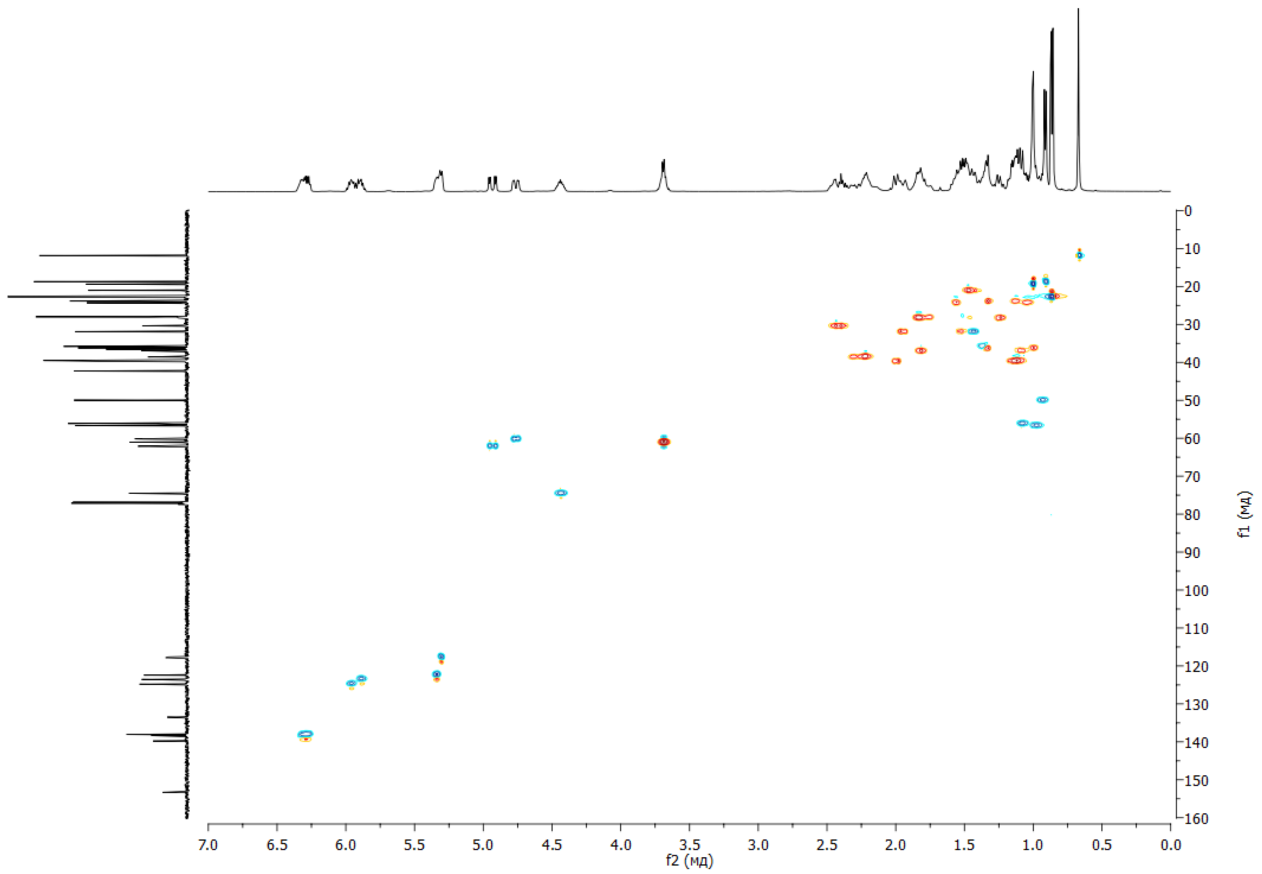

Figure S51. HMBC spectrum of compound **4g** (500 MHz, $CDCl_3$)

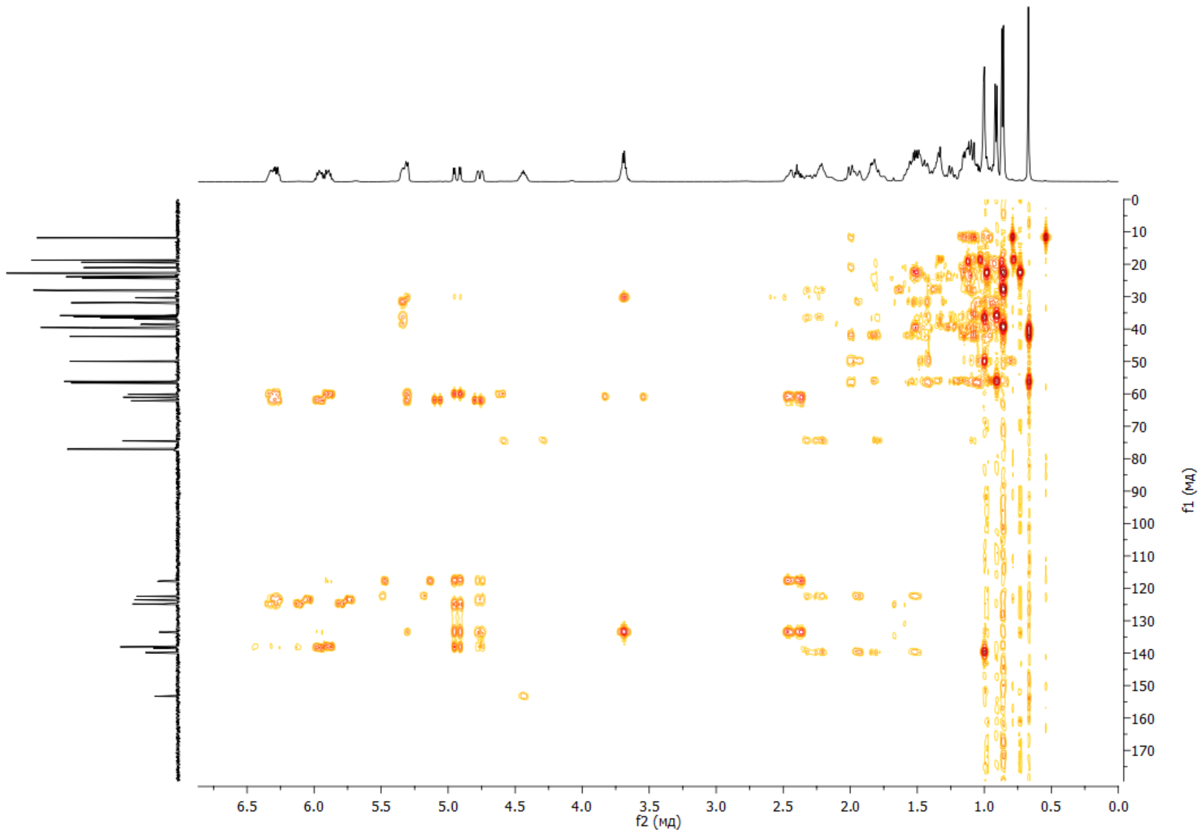

Figure S52. ^{13}C NMR Spectrum of compound **4h** (125 MHz, $CDCl_3$)

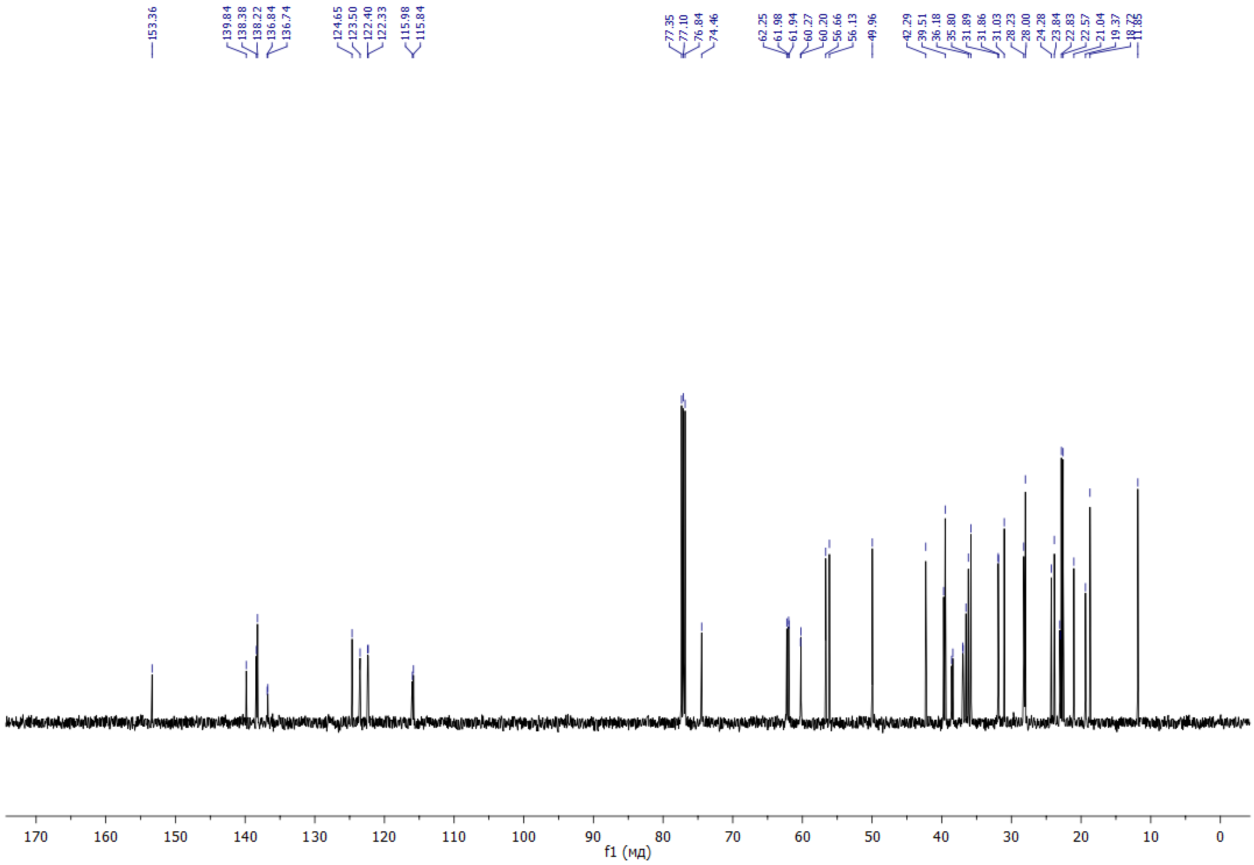

Figure S53. ^{1}H NMR Spectrum of compound **4h** (500 MHz, $CDCl_3$)

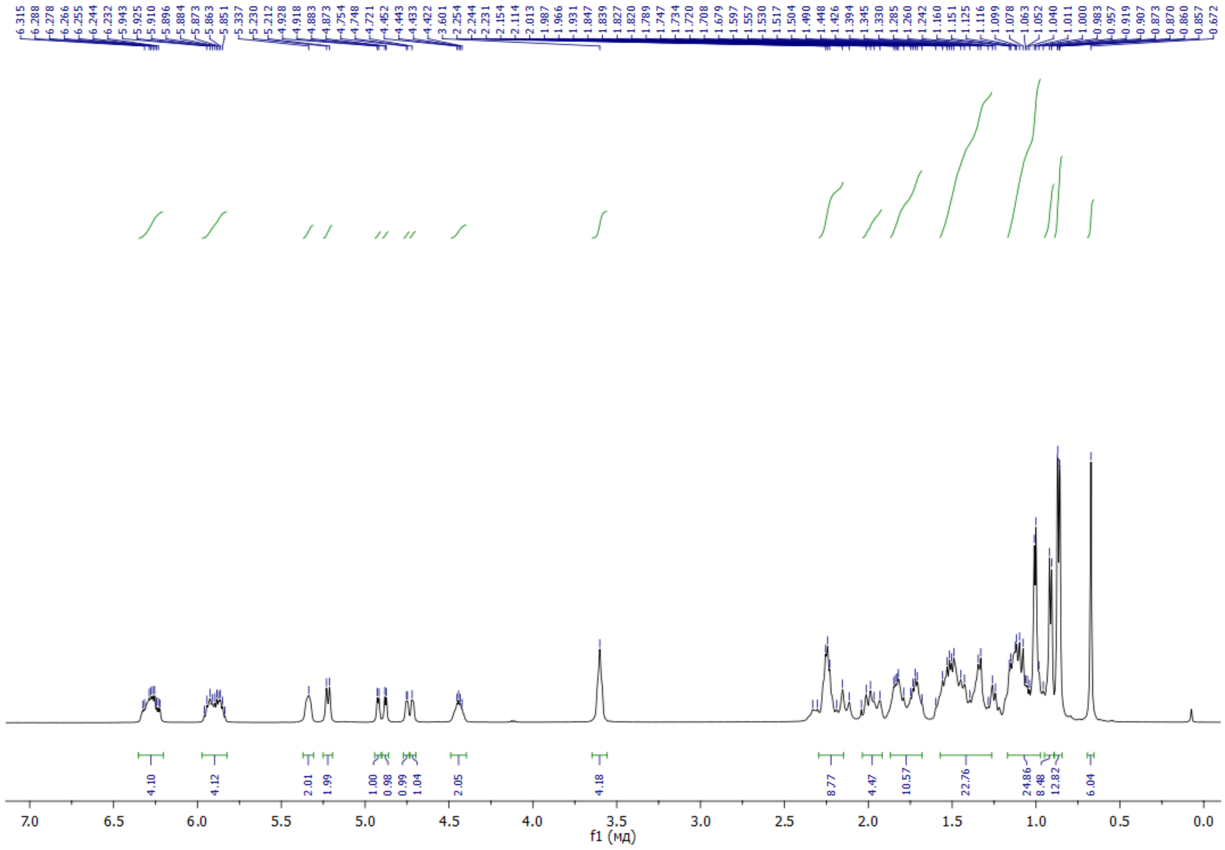

Figure S54. NOESY Spectrum of compound **4h** (500 MHz, $CDCl_3$)

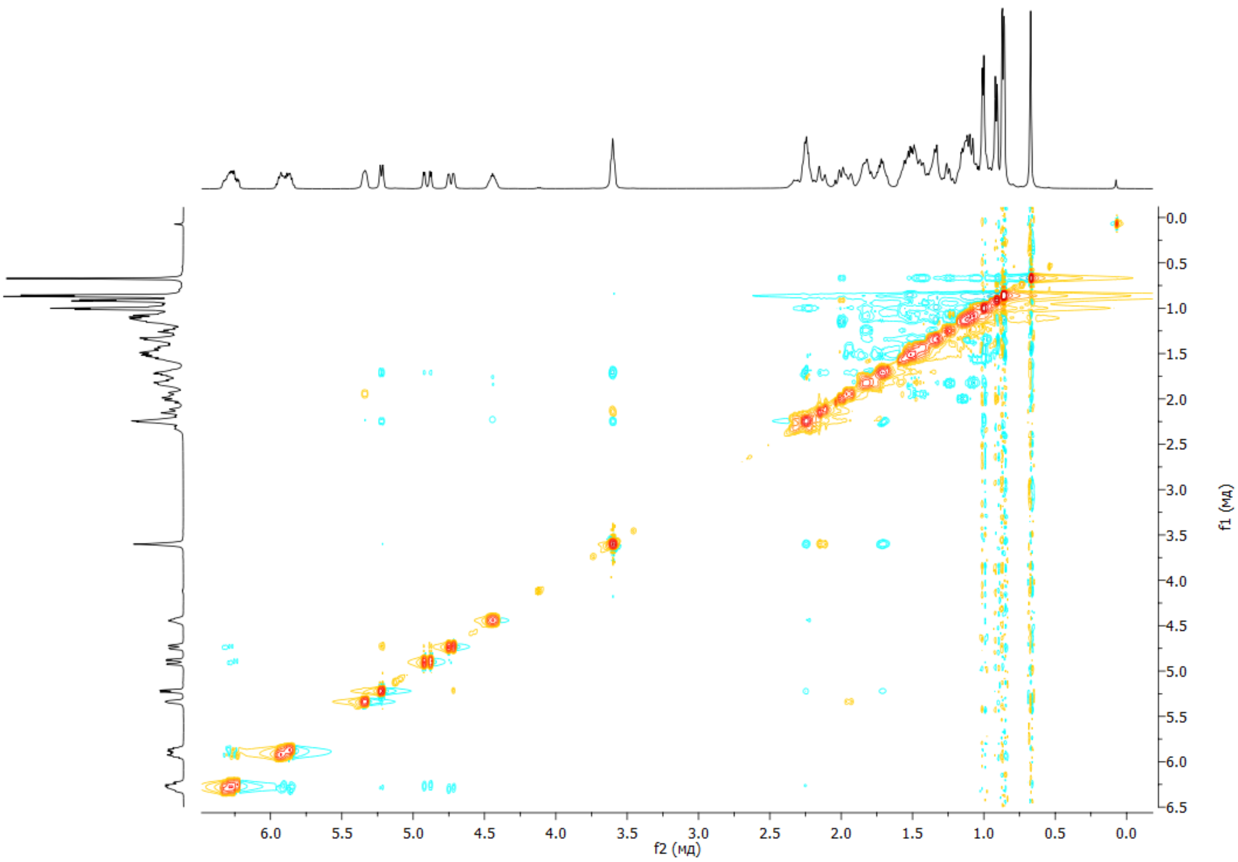

Figure S55. COSY Spectrum of compound **4h** (500 MHz, $CDCl_3$)

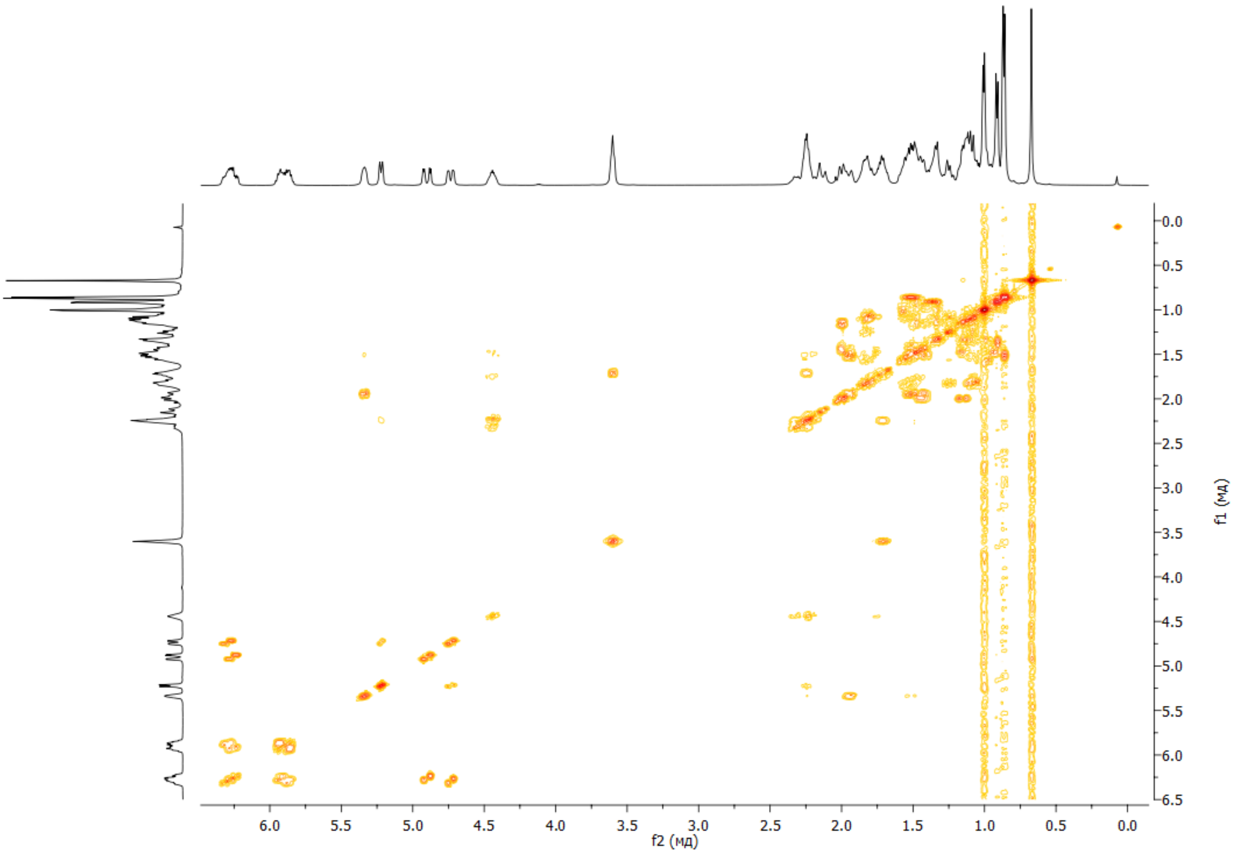

Figure S56. HSQC spectrum of compound **4h** (500 MHz, $CDCl_3$)

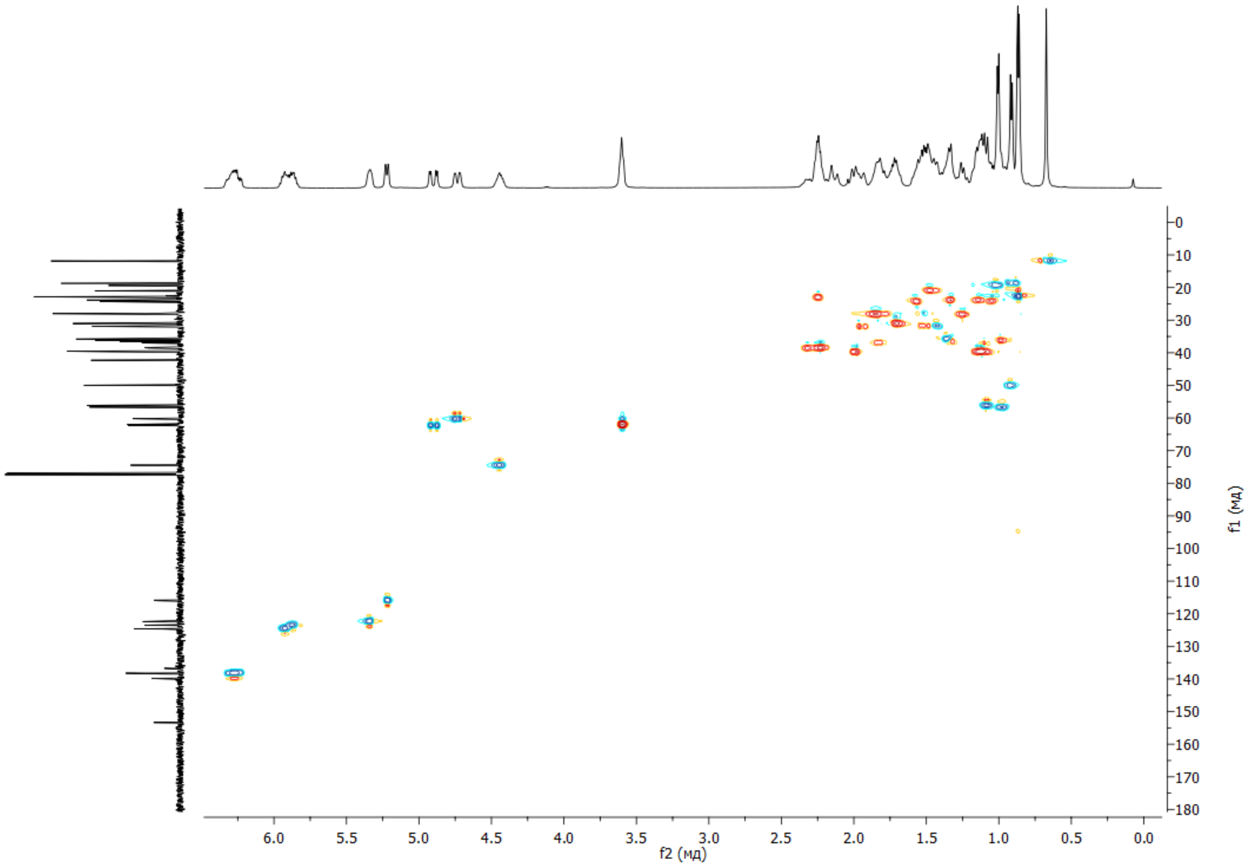

Figure S57. HMBC spectrum of compound **4h** (500 MHz, $CDCl_3$)

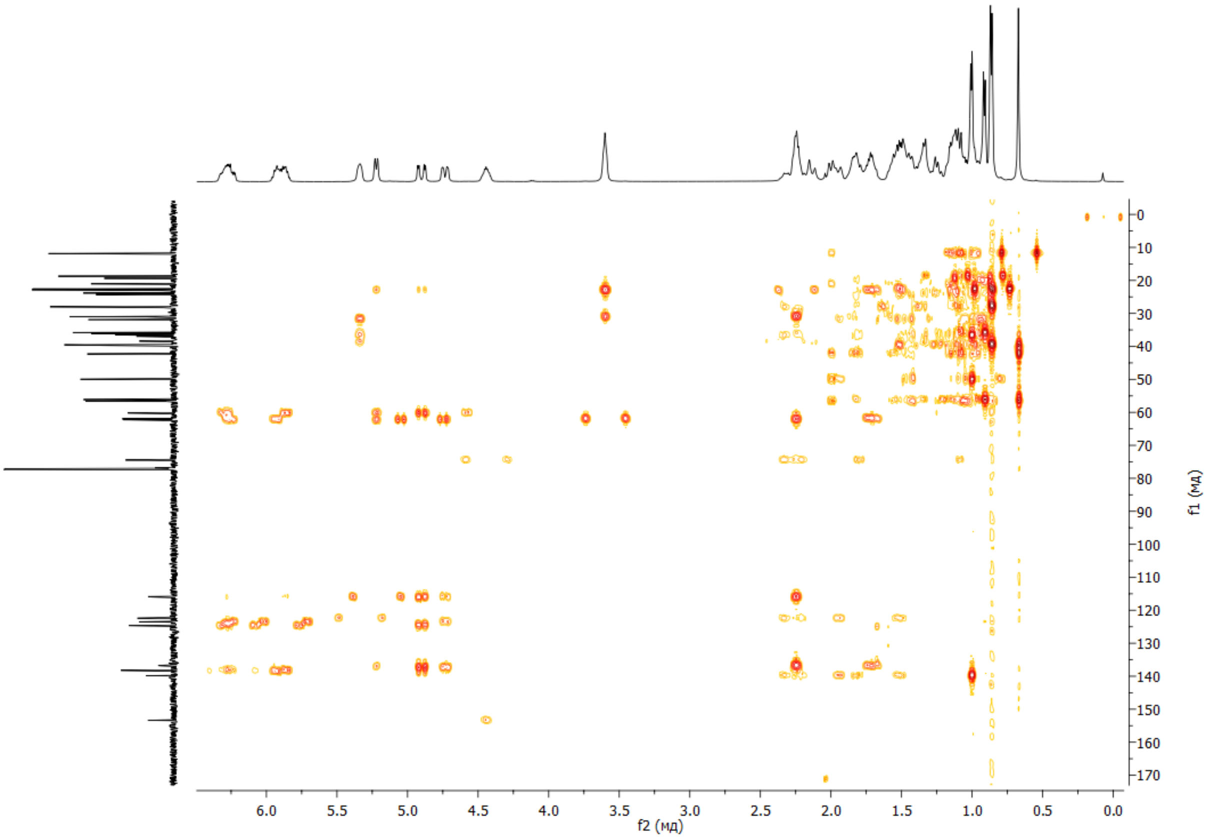

Figure S58. ^{13}C NMR Spectrum of compound **4i** (125 MHz, $CDCl_3$)

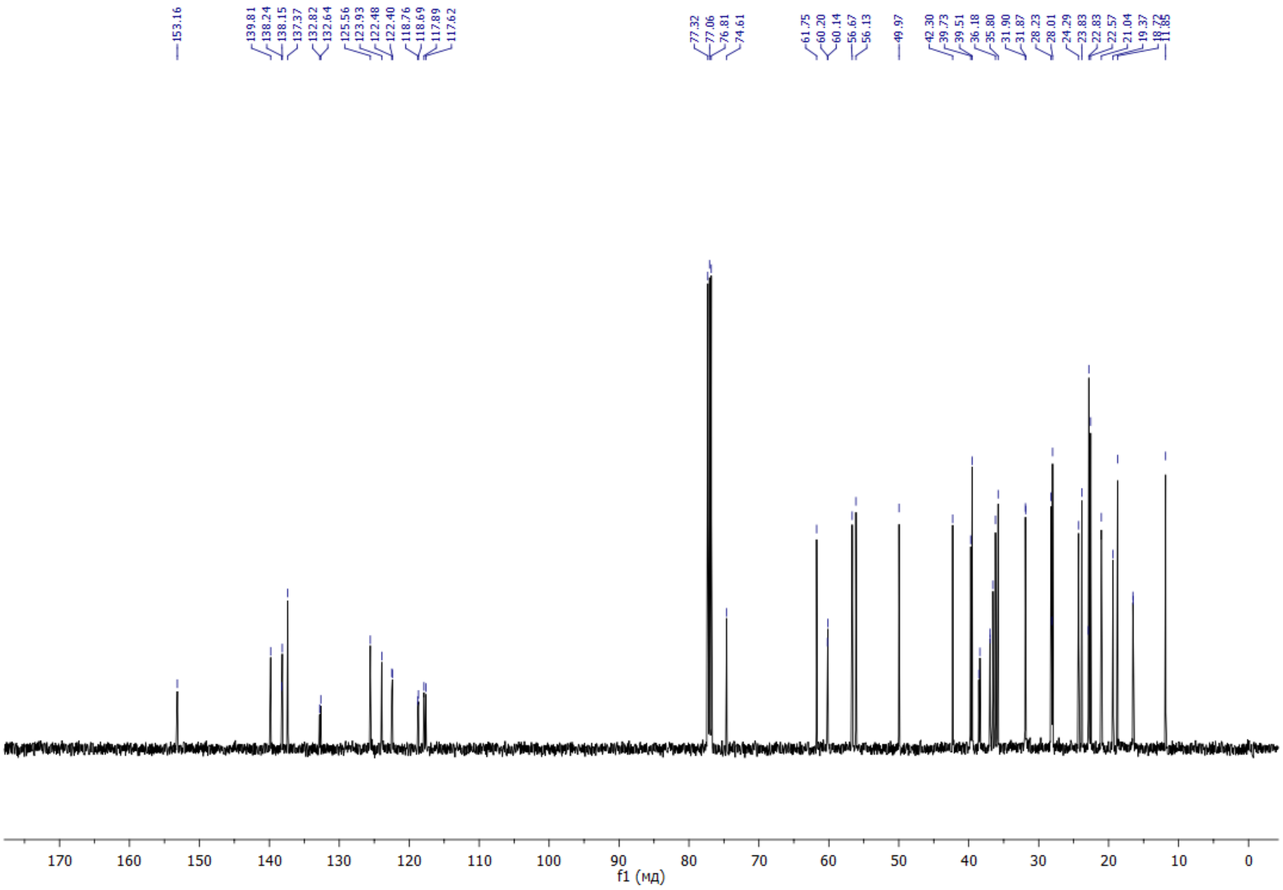

Figure S59. ^{1}H NMR Spectrum of compound **4i** (500 MHz, $CDCl_3$)

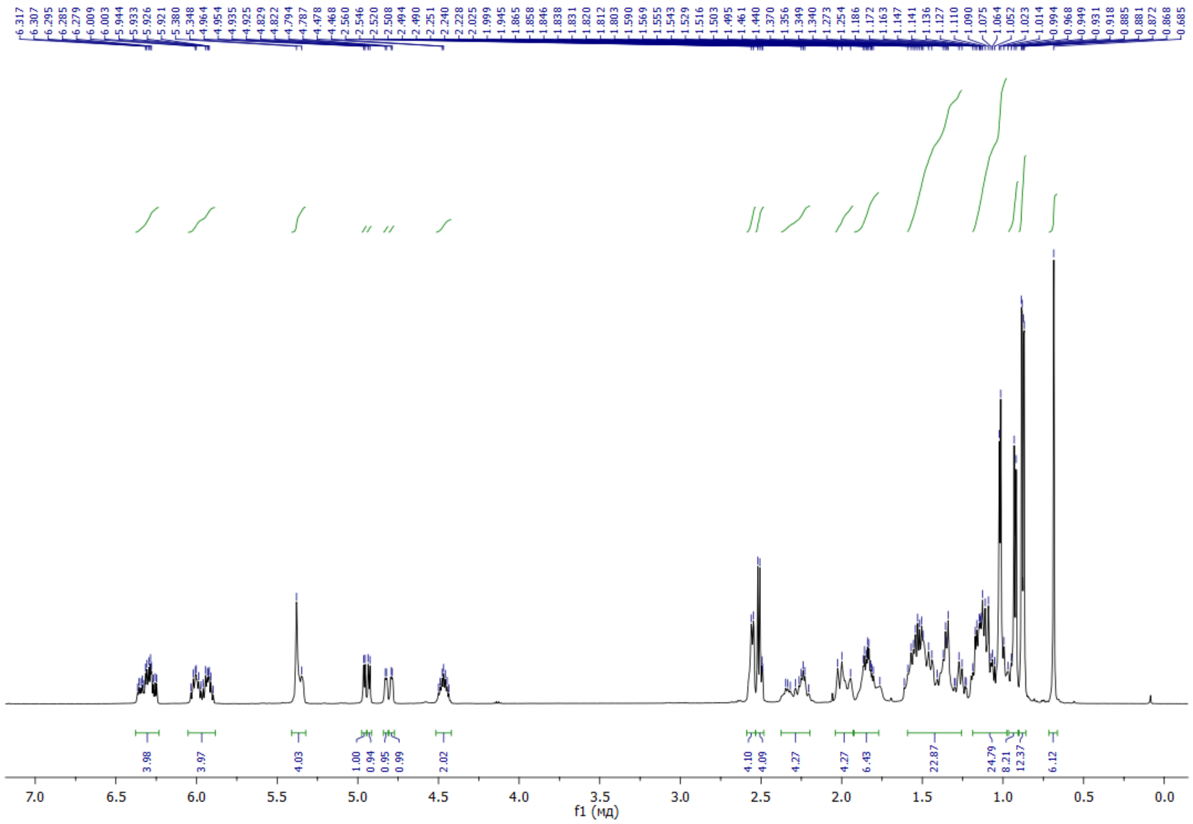

Figure S60. NOESY Spectrum of compound **4i** (500 MHz, $CDCl_3$)

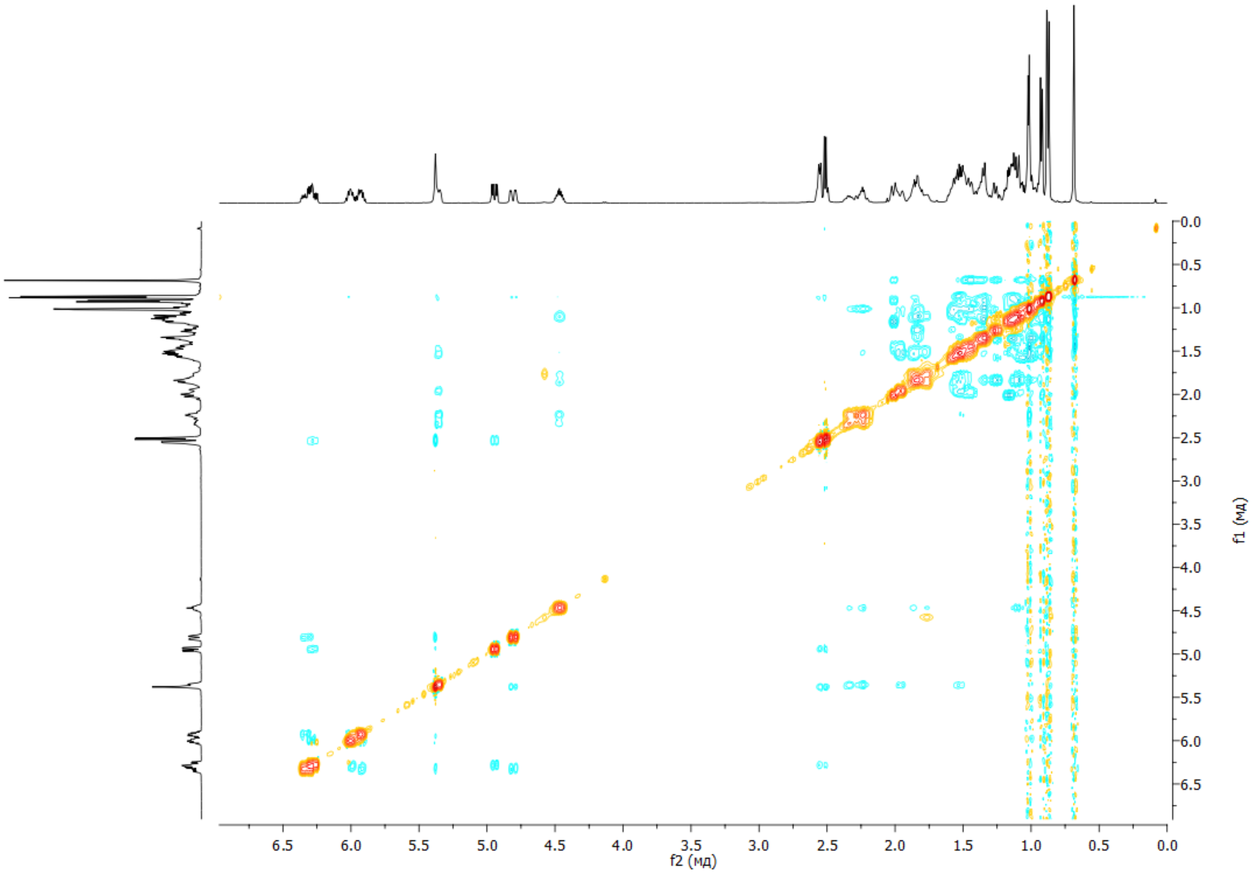

Figure S61. COSY Spectrum of compound **4i** (500 MHz, $CDCl_3$)

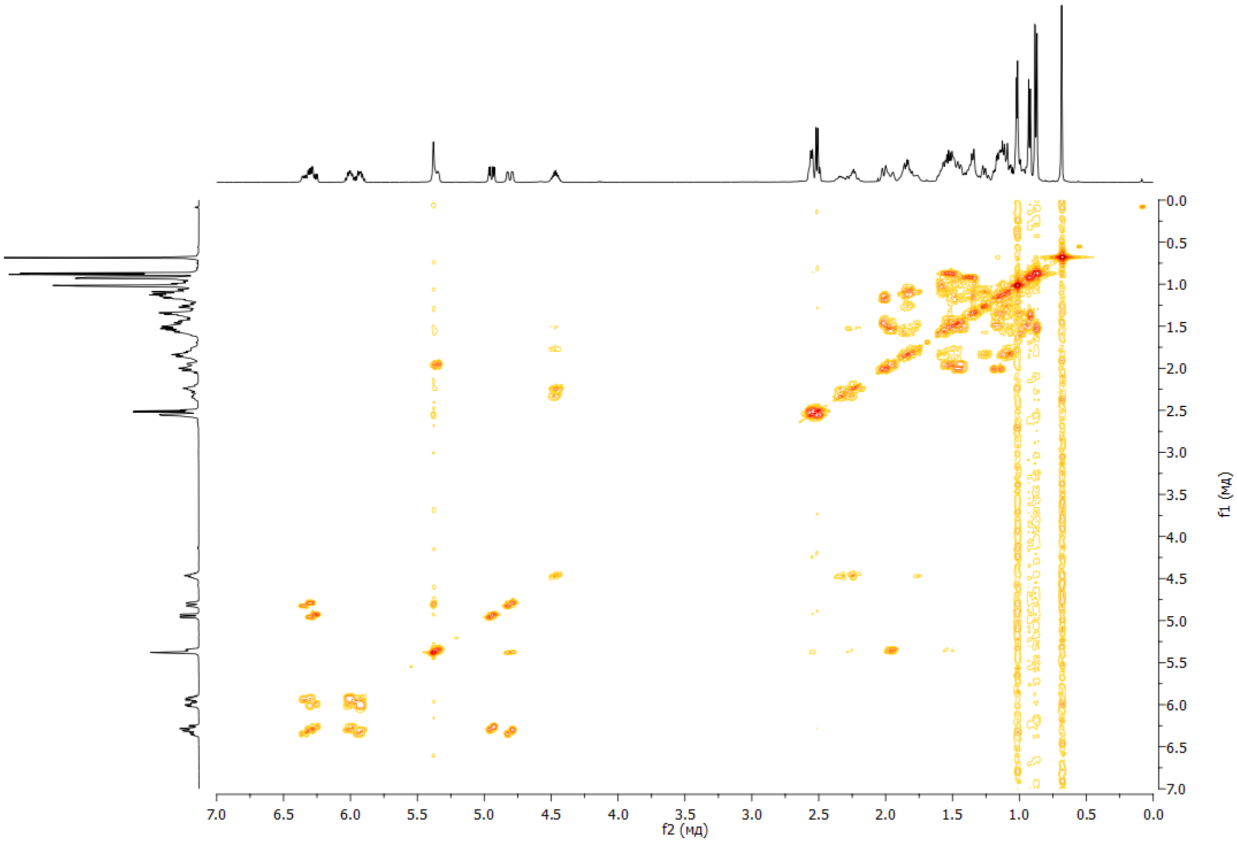

Figure S62. HSQC spectrum of compound **4i** (500 MHz, $CDCl_3$)

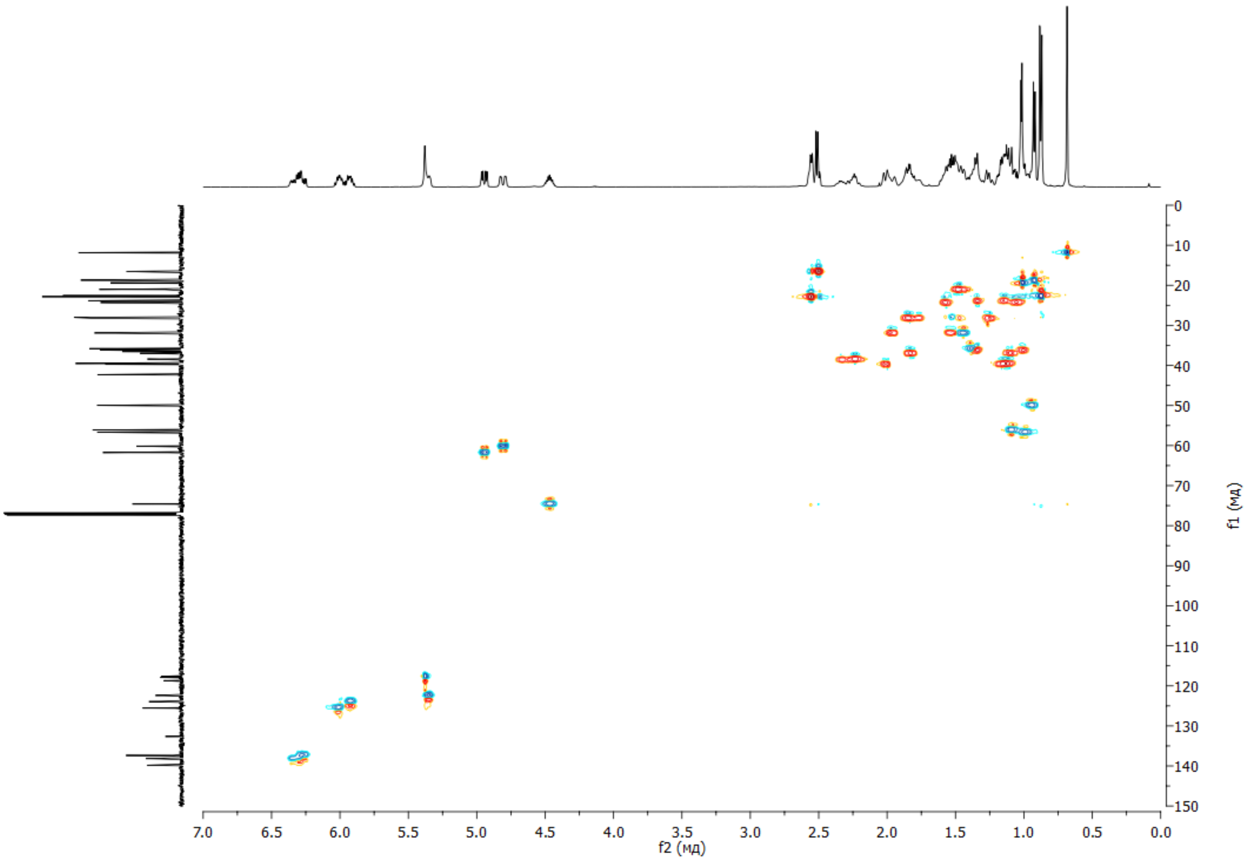

Figure S63. HMBC spectrum of compound **4i** (500 MHz, $CDCl_3$)

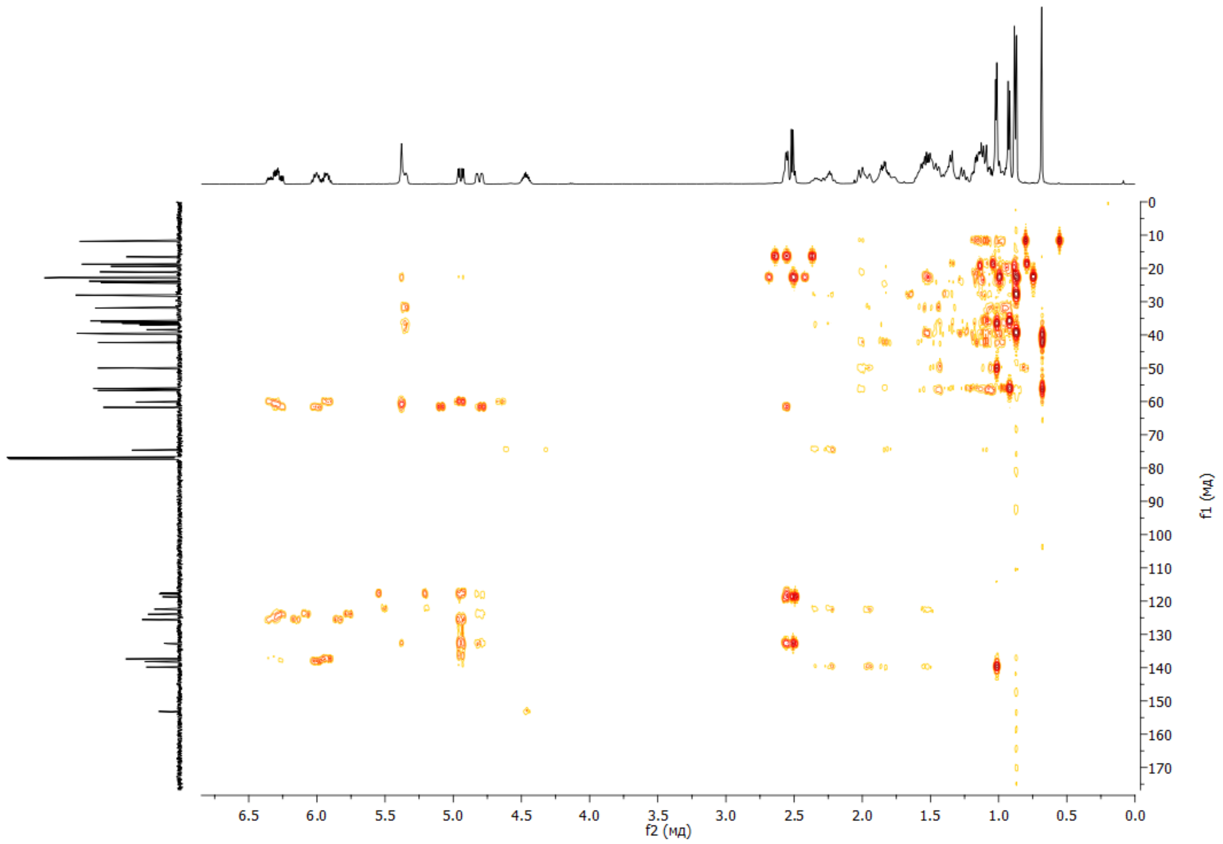

Figure S64. ^{13}C NMR Spectrum of compound **4j** (125 MHz, $CDCl_3$)

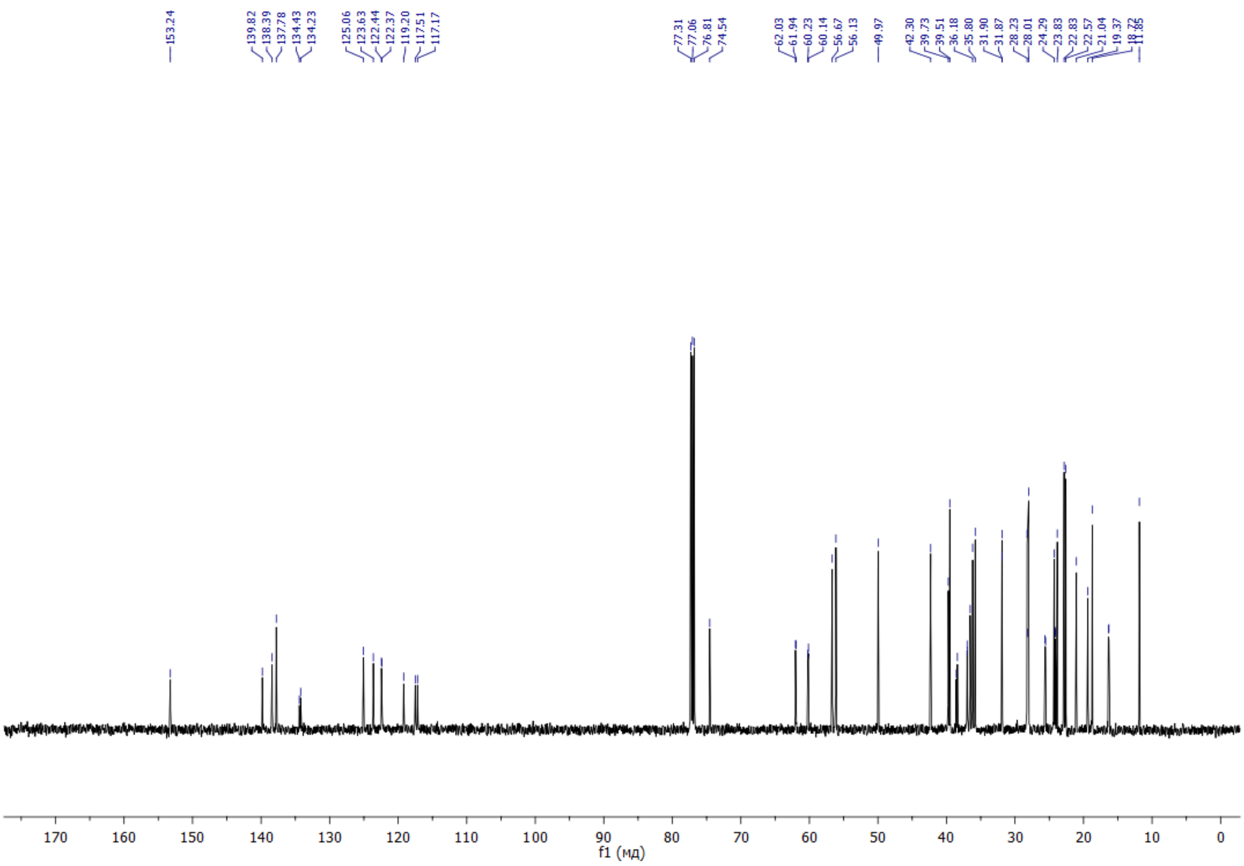

Figure S65. ^{1}H NMR Spectrum of compound **4j** (500 MHz, $CDCl_3$)

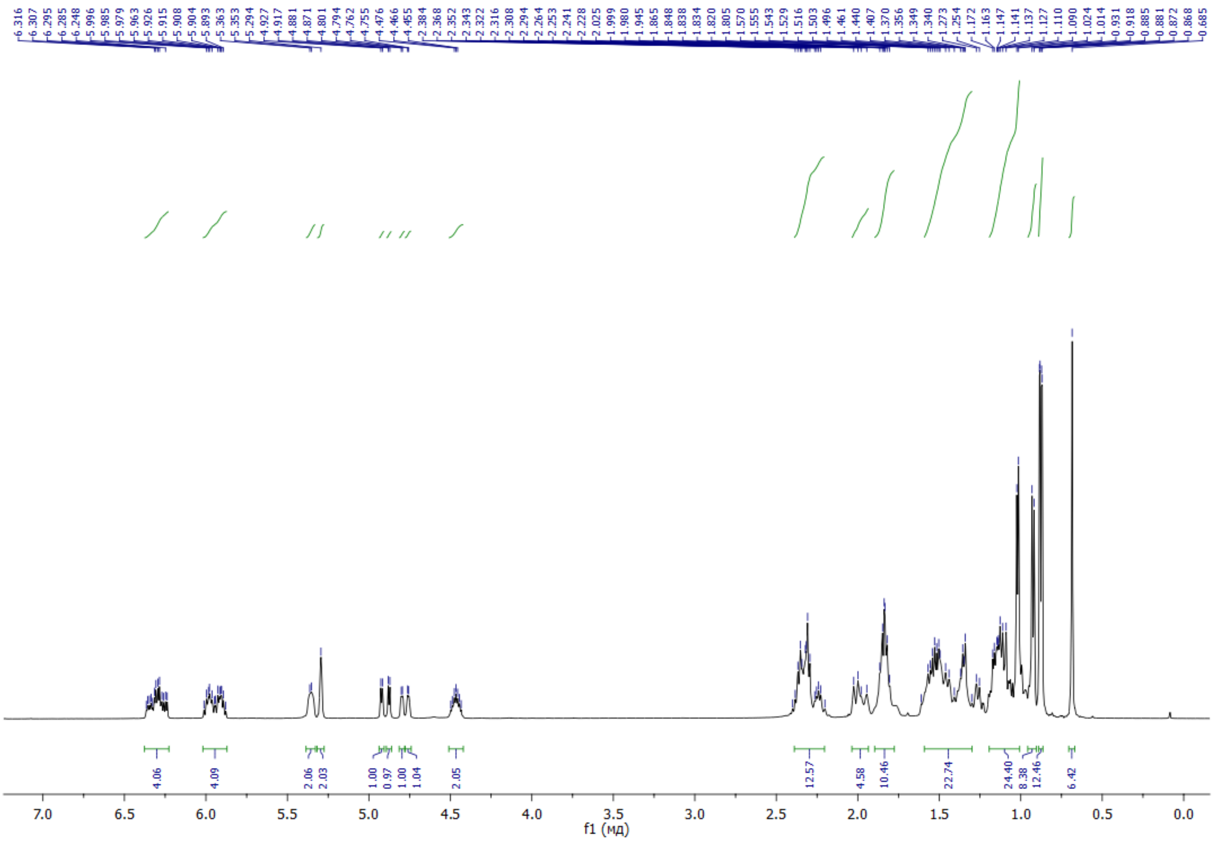

Figure S66. ^{13}C NMR Spectrum of compound **4k** (125 MHz, $CDCl_3$)

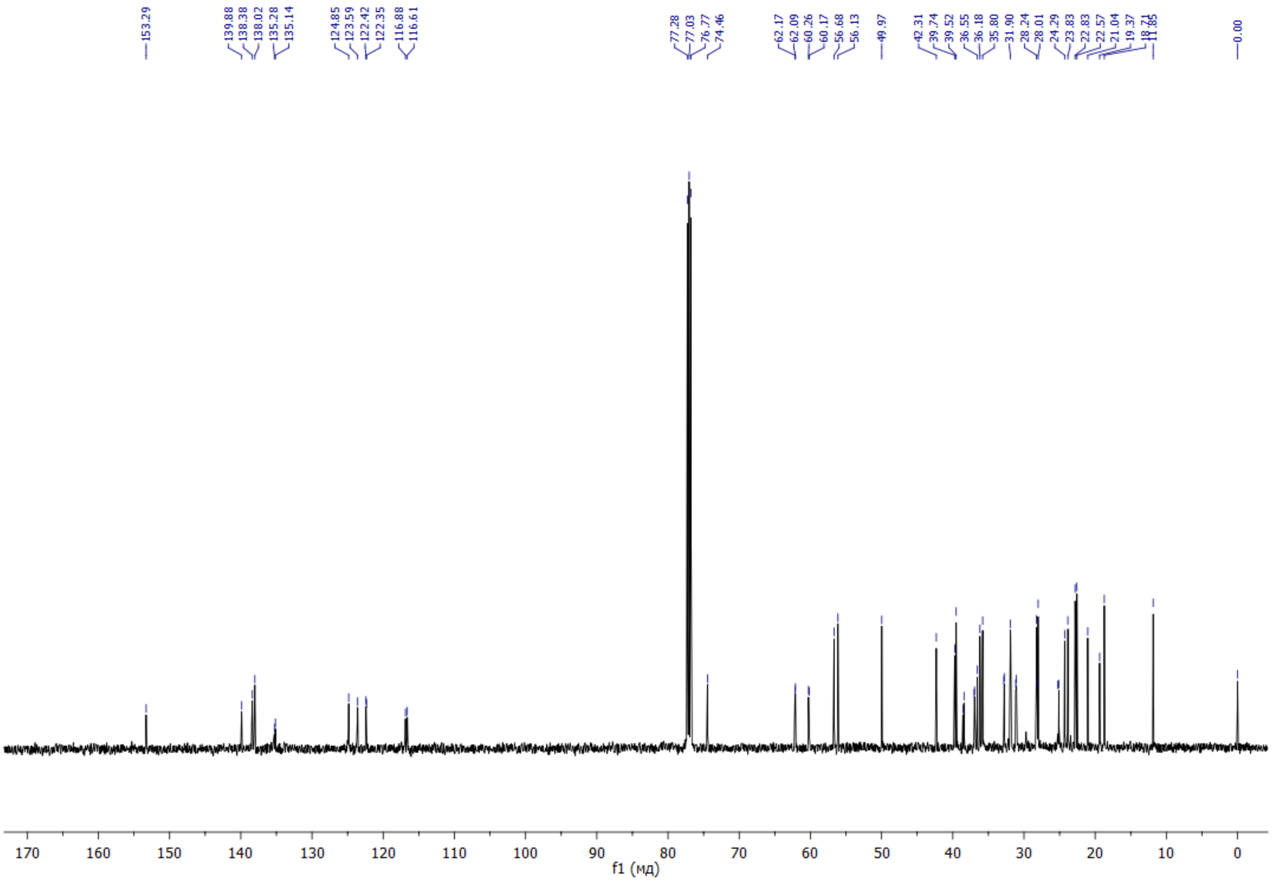

Figure S67. ^{1}H NMR Spectrum of compound **4k** (500 MHz, $CDCl_3$)

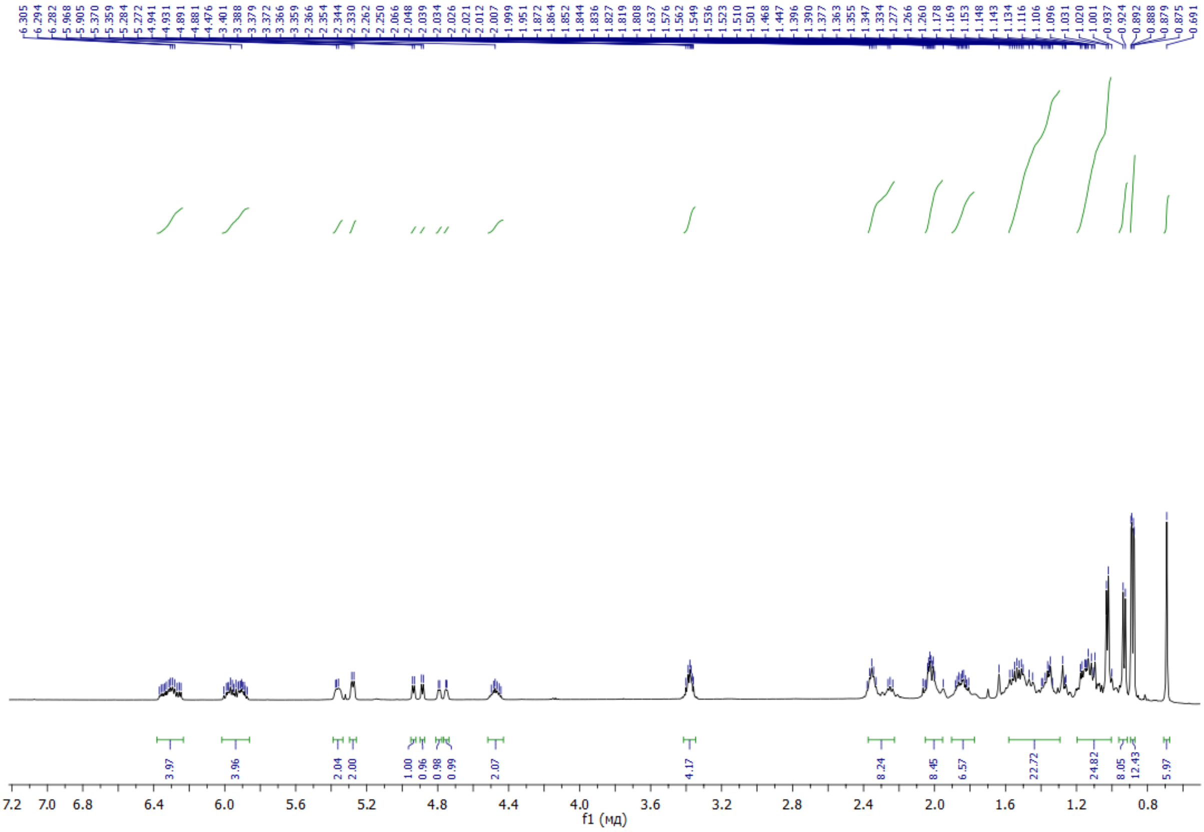

Figure S68. NOESY Spectrum of compound **4k** (500 MHz, $CDCl_3$)

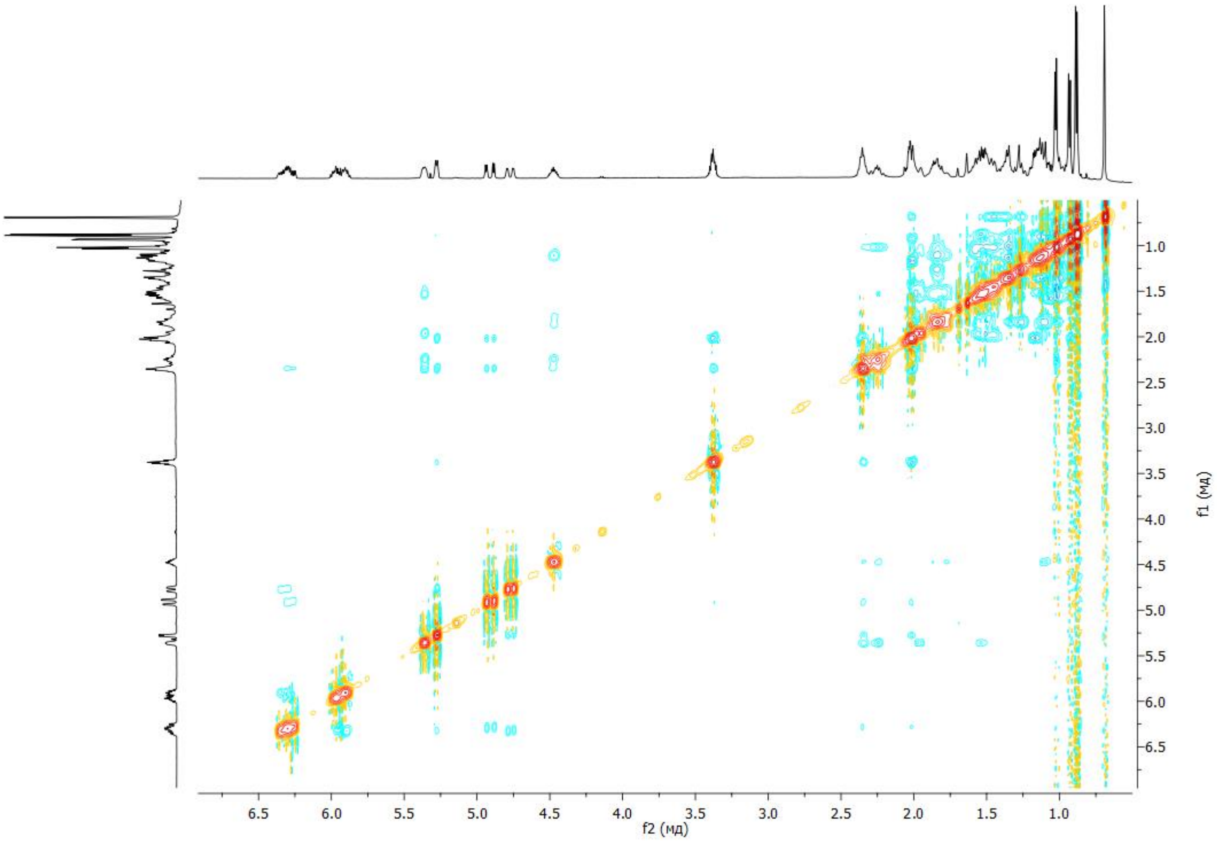

Figure S69. COSY Spectrum of compound **4k** (500 MHz, $CDCl_3$)

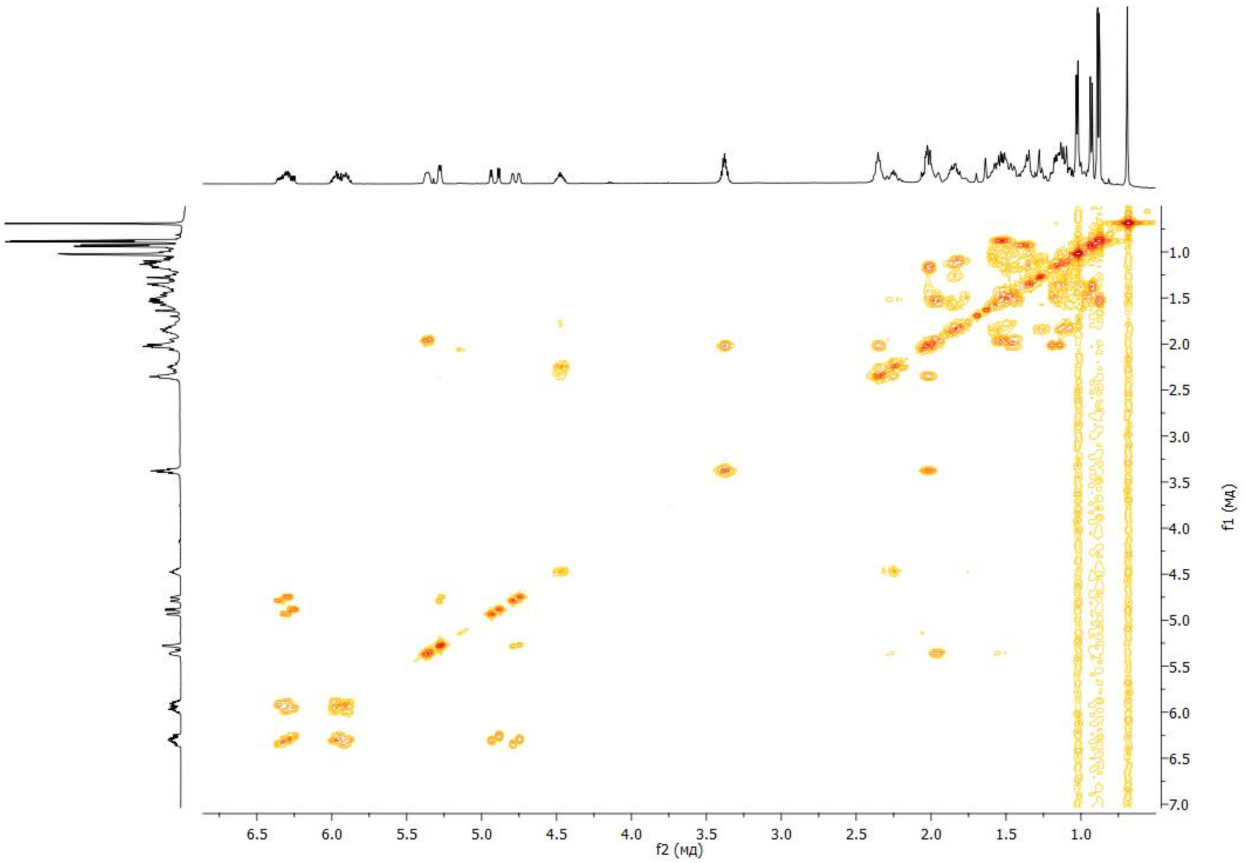

Figure S70. HSQC spectrum of compound **4k** (500 MHz, $CDCl_3$)

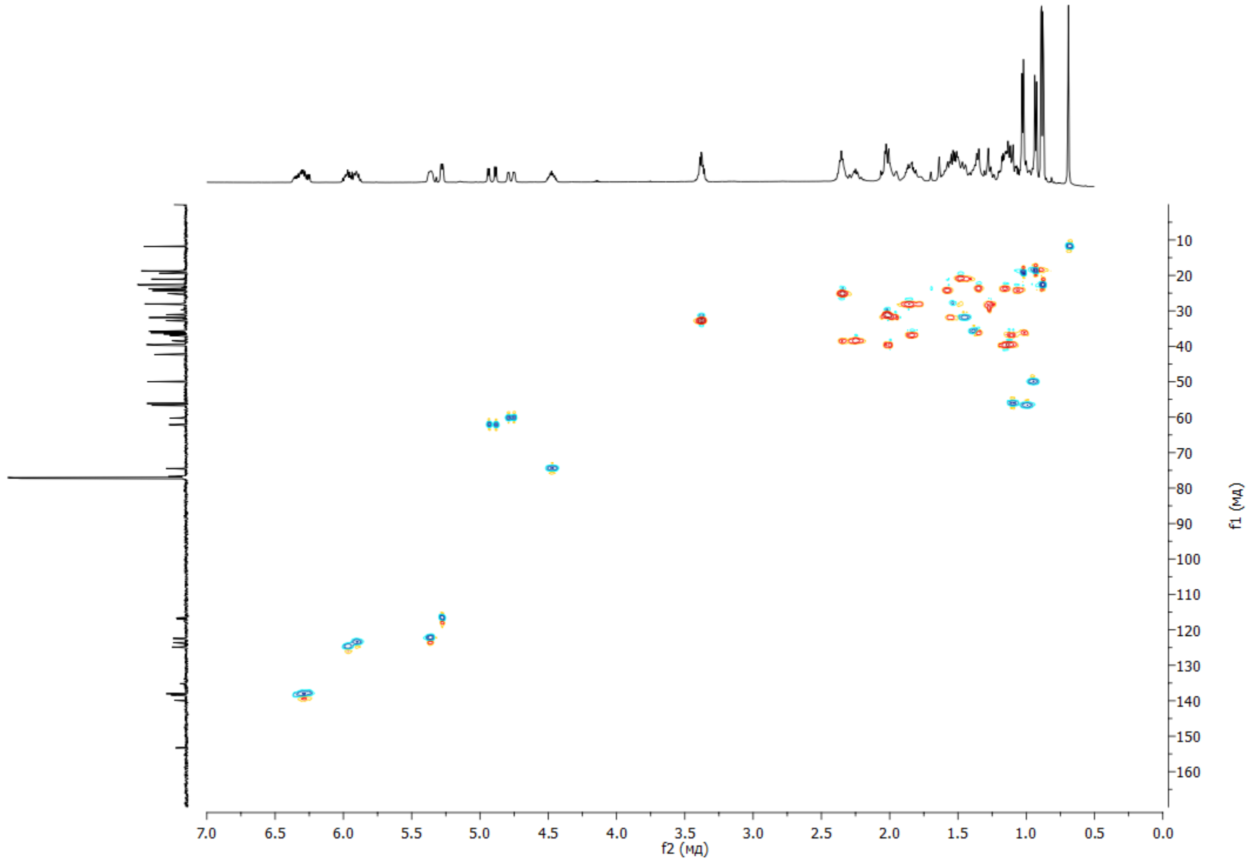

Figure S71. HMBC spectrum of compound **4k** (500 MHz, $CDCl_3$)

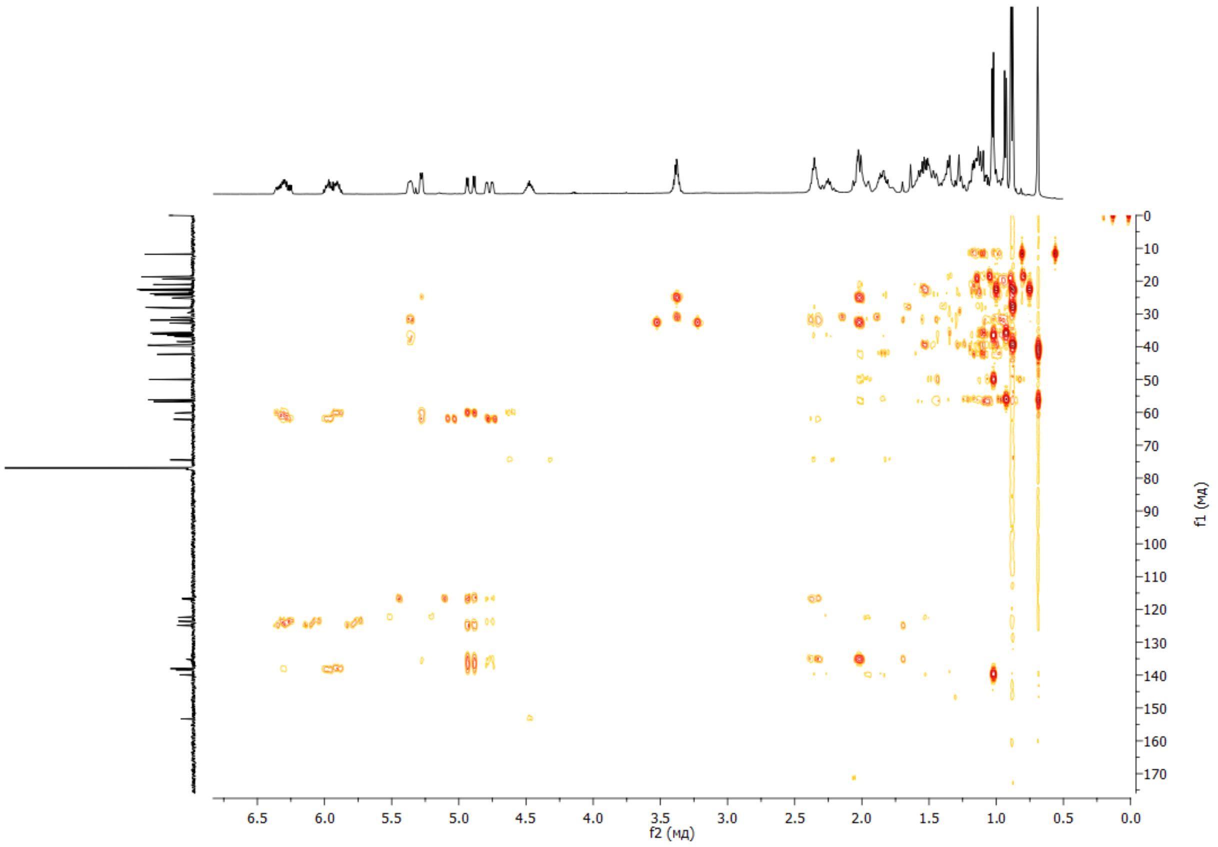

Figure S72. ^{13}C NMR Spectrum of compound **4l** (125 MHz, $CDCl_3$)

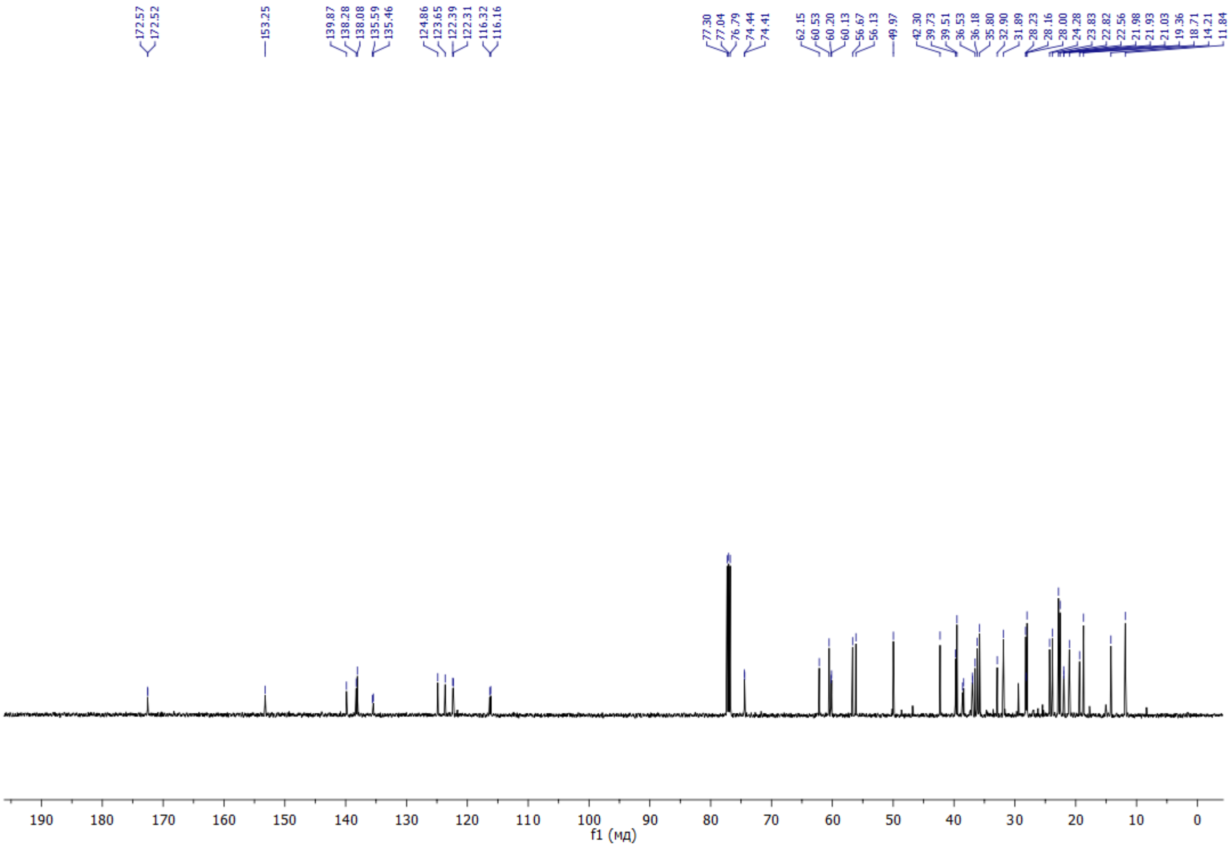

Figure S73. ^{1}H NMR Spectrum of compound **4l** (500 MHz, $CDCl_3$)

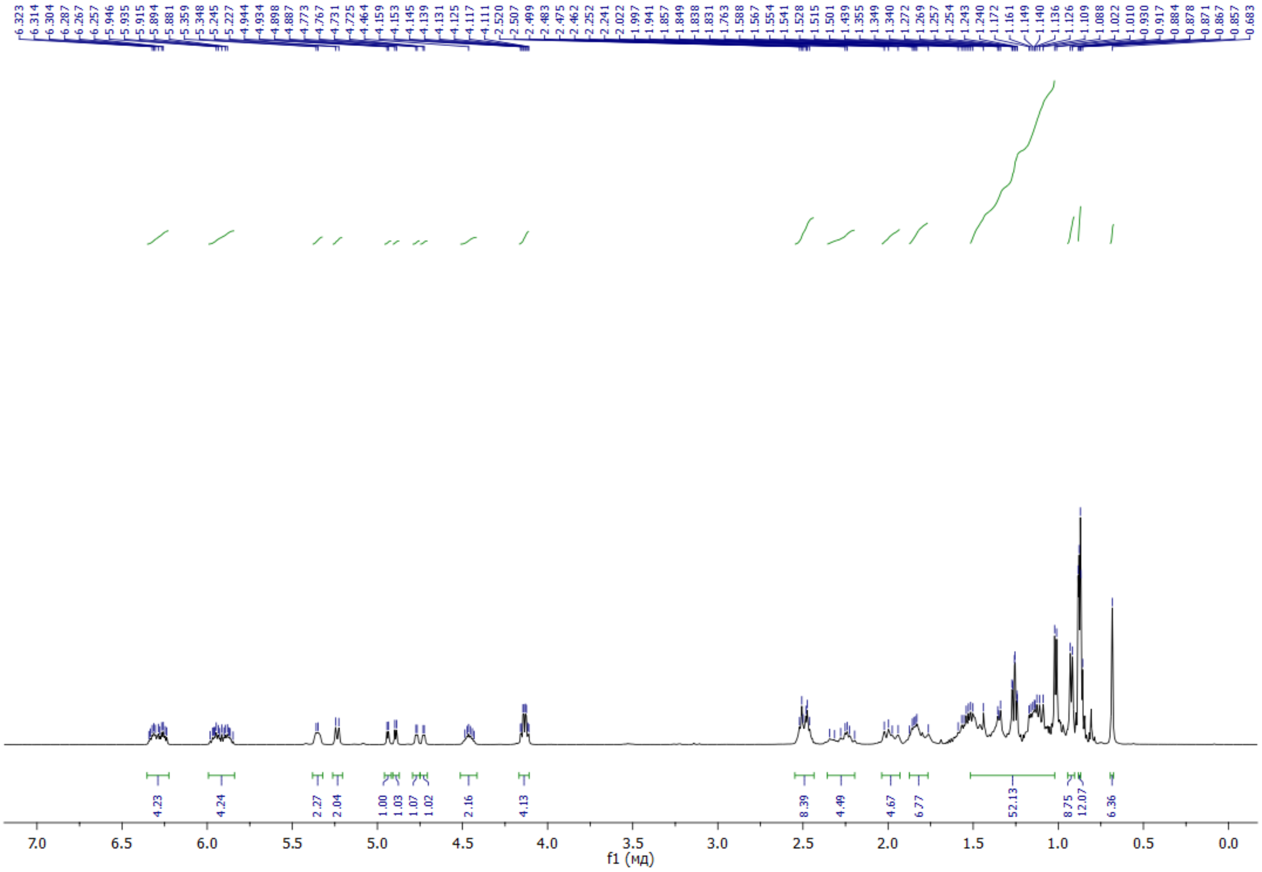

Figure S74. NOESY Spectrum of compound **4l** (500 MHz, $CDCl_3$)

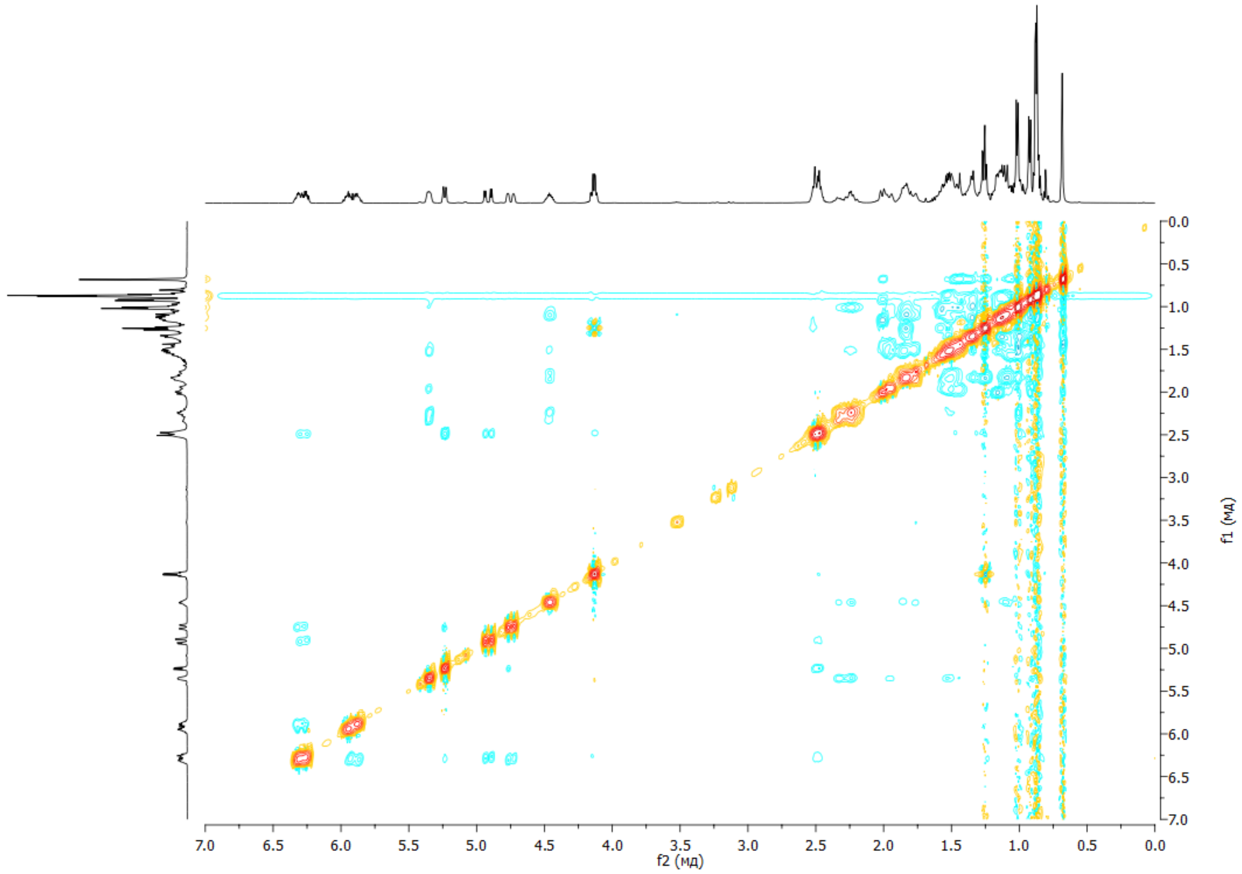

Figure S75. COSY Spectrum of compound **4l** (500 MHz, $CDCl_3$)

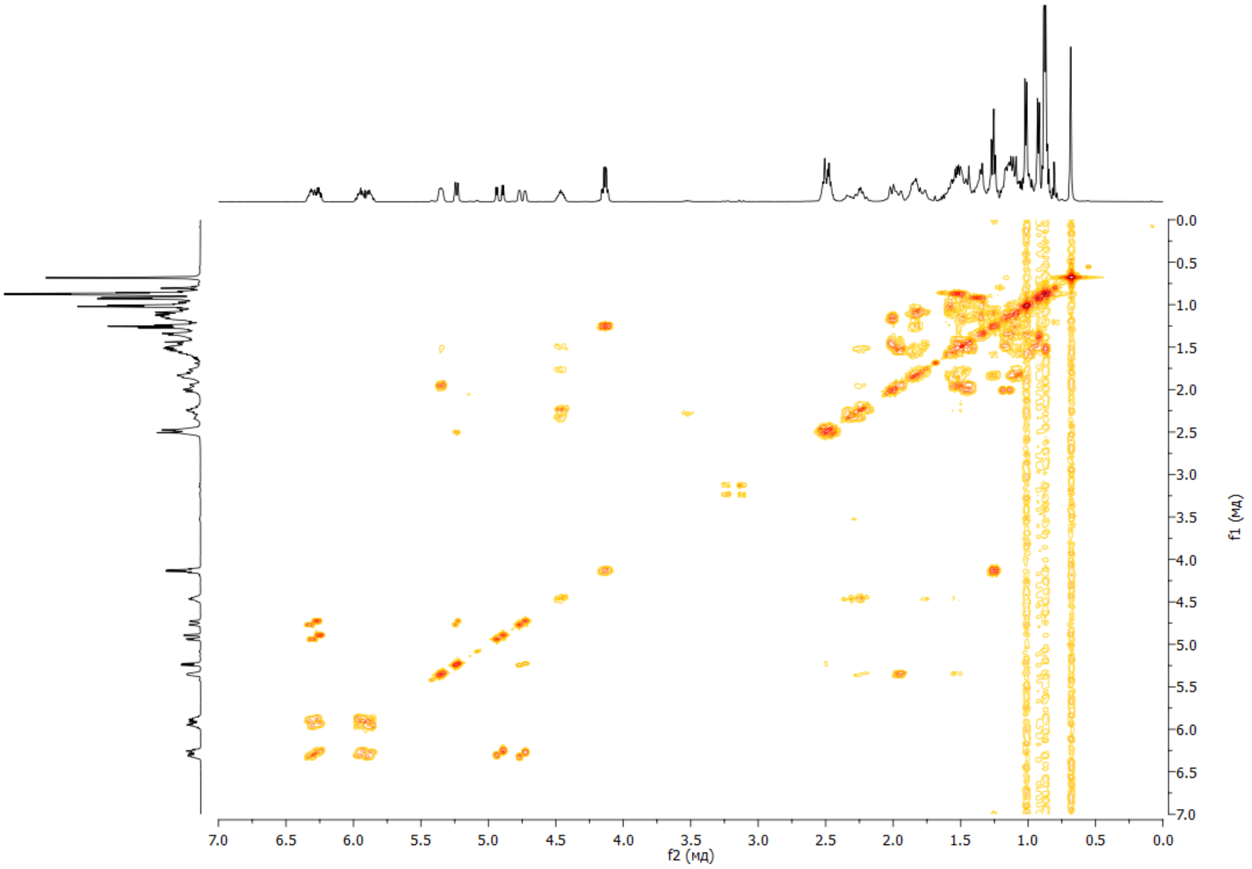

Figure S76. HSQC spectrum of compound **4l** (500 MHz, $CDCl_3$)

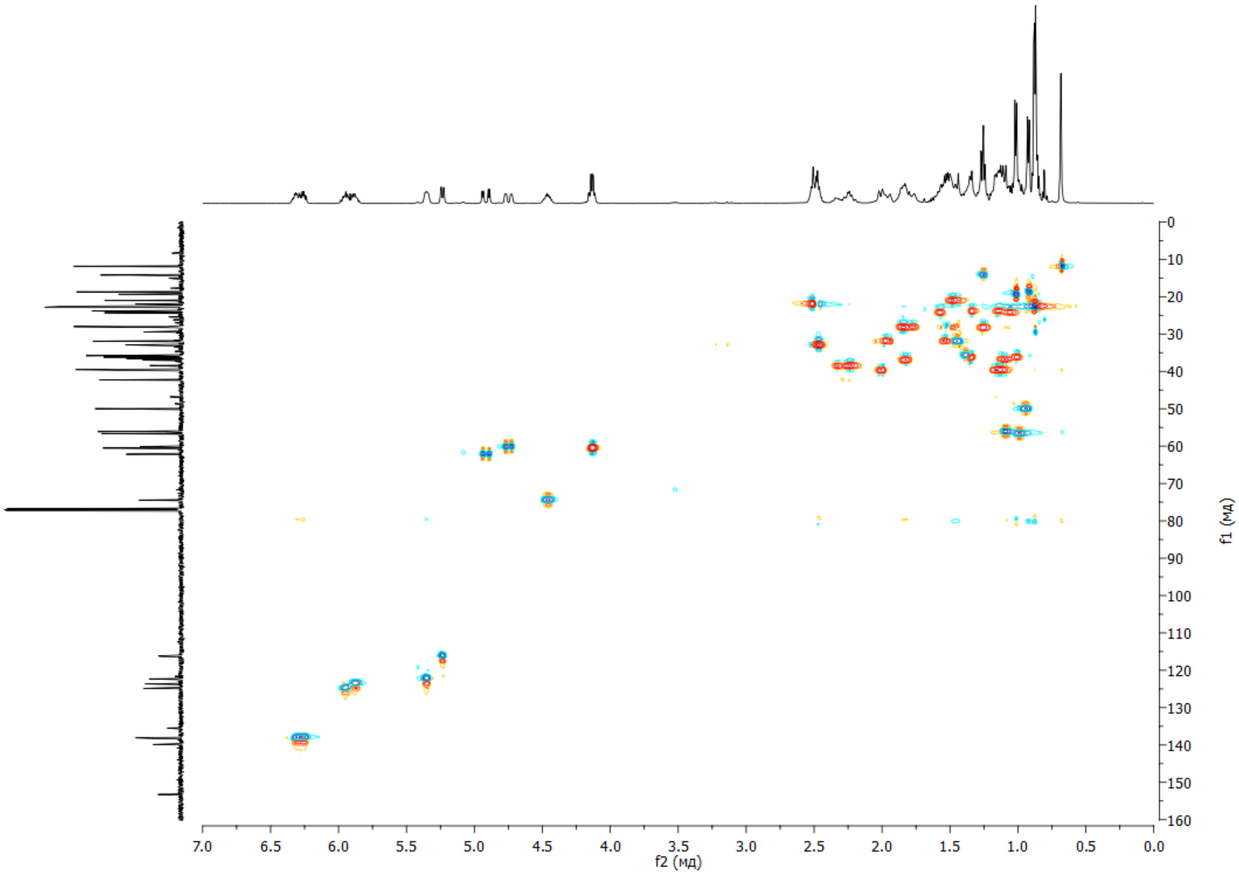

Figure S77. HMBC spectrum of compound **4l** (500 MHz, $CDCl_3$)

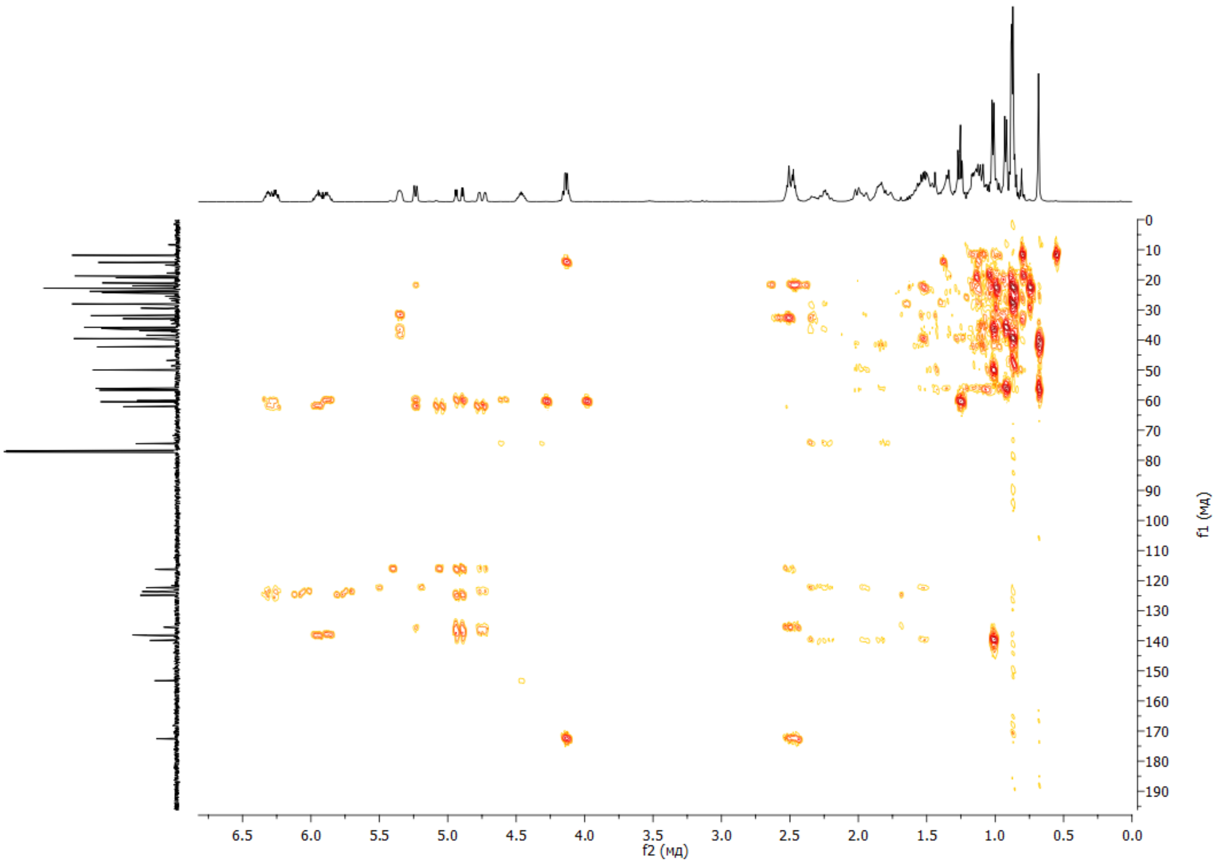

Figure S78. ^{13}C NMR Spectrum of compound **4m** (125 MHz, $CDCl_3$)

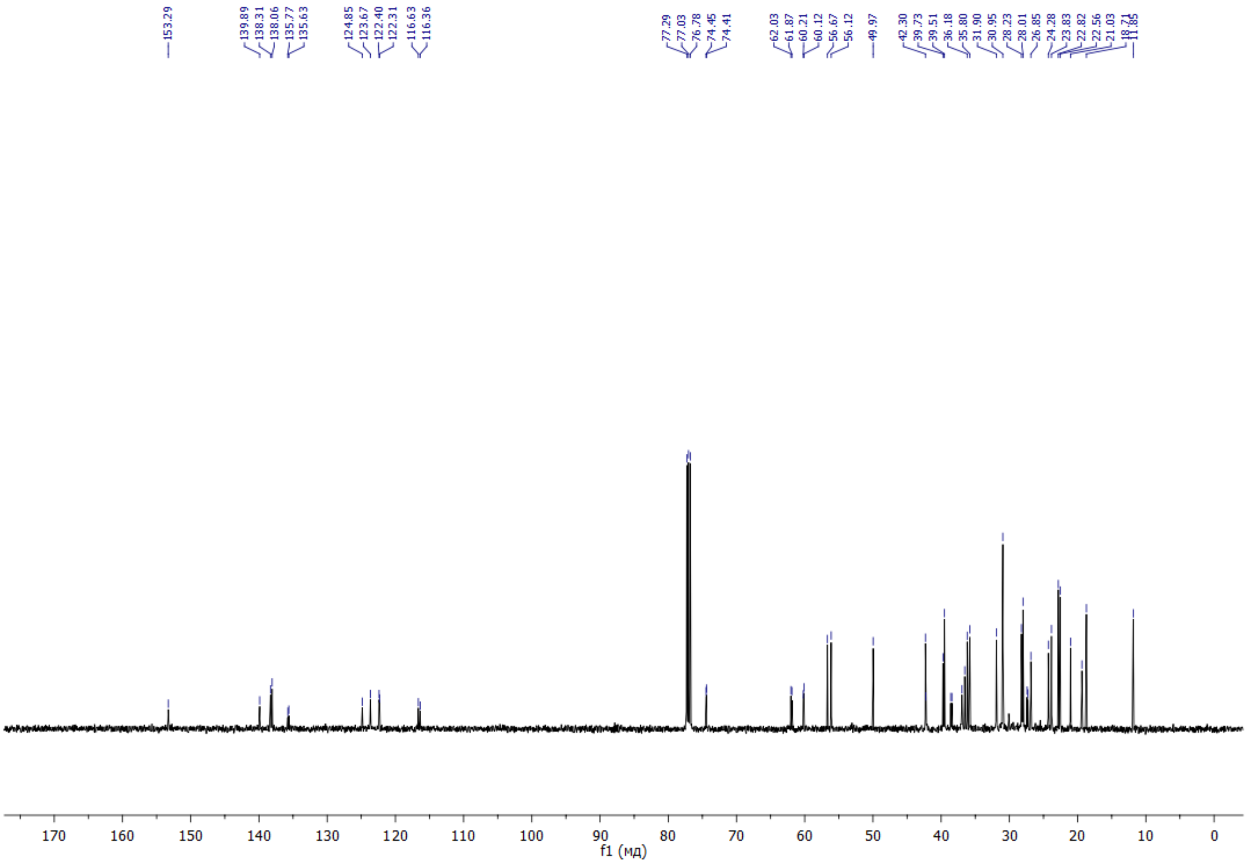

Figure S79. ^{1}H NMR Spectrum of compound **4m** (500 MHz, $CDCl_3$)

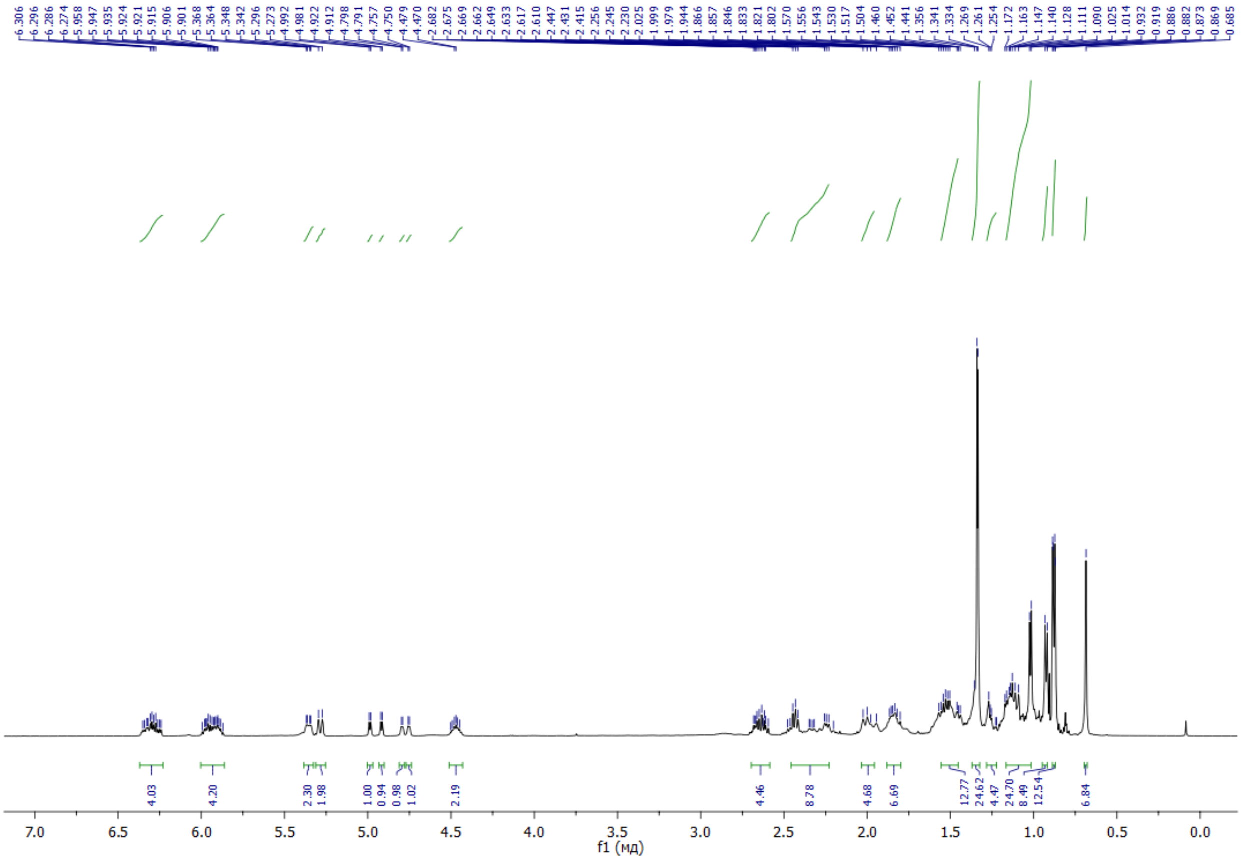

Figure S80. ^{13}C NMR Spectrum of compound **4n** (125 MHz, $CDCl_3$)

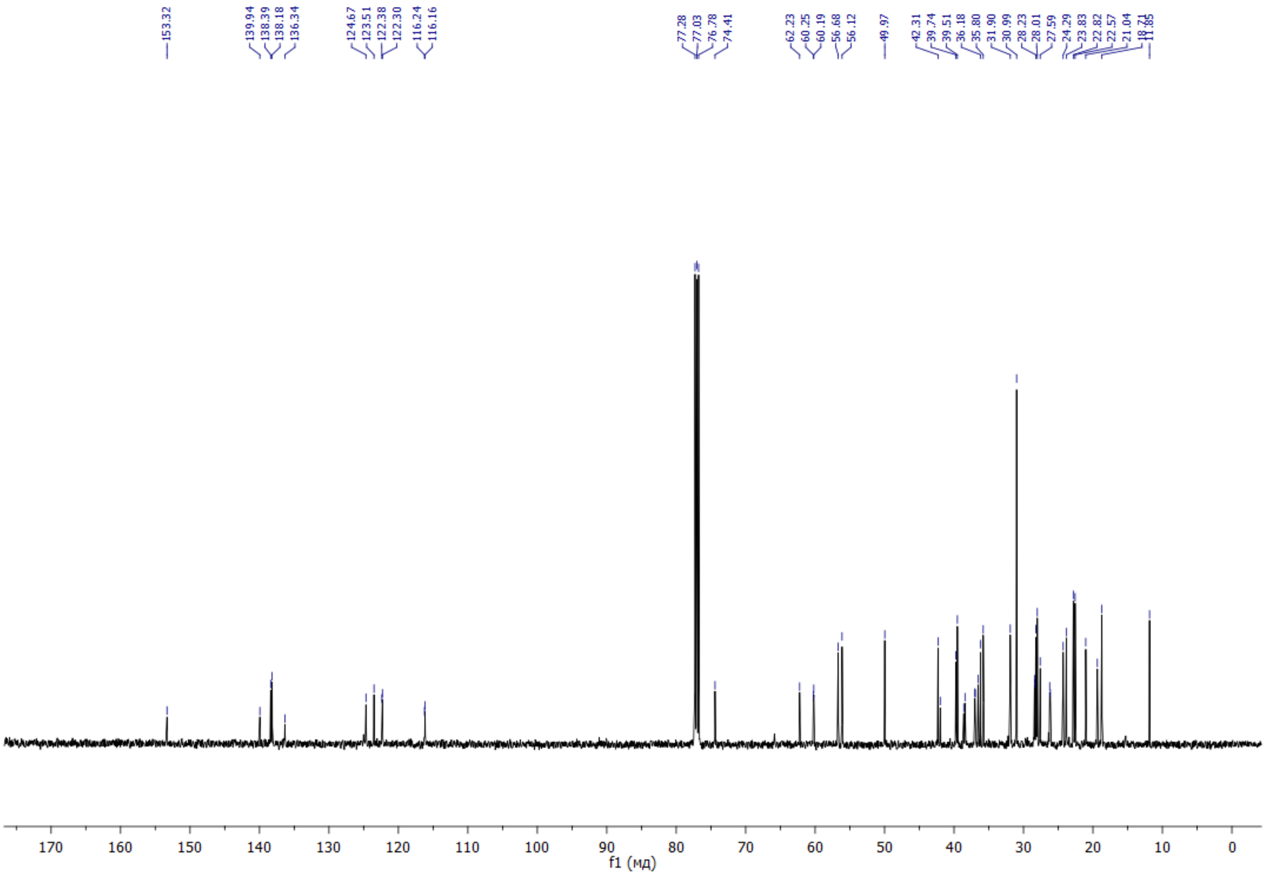

Figure S81. ^{1}H NMR Spectrum of compound **4n** (500 MHz, $CDCl_3$)

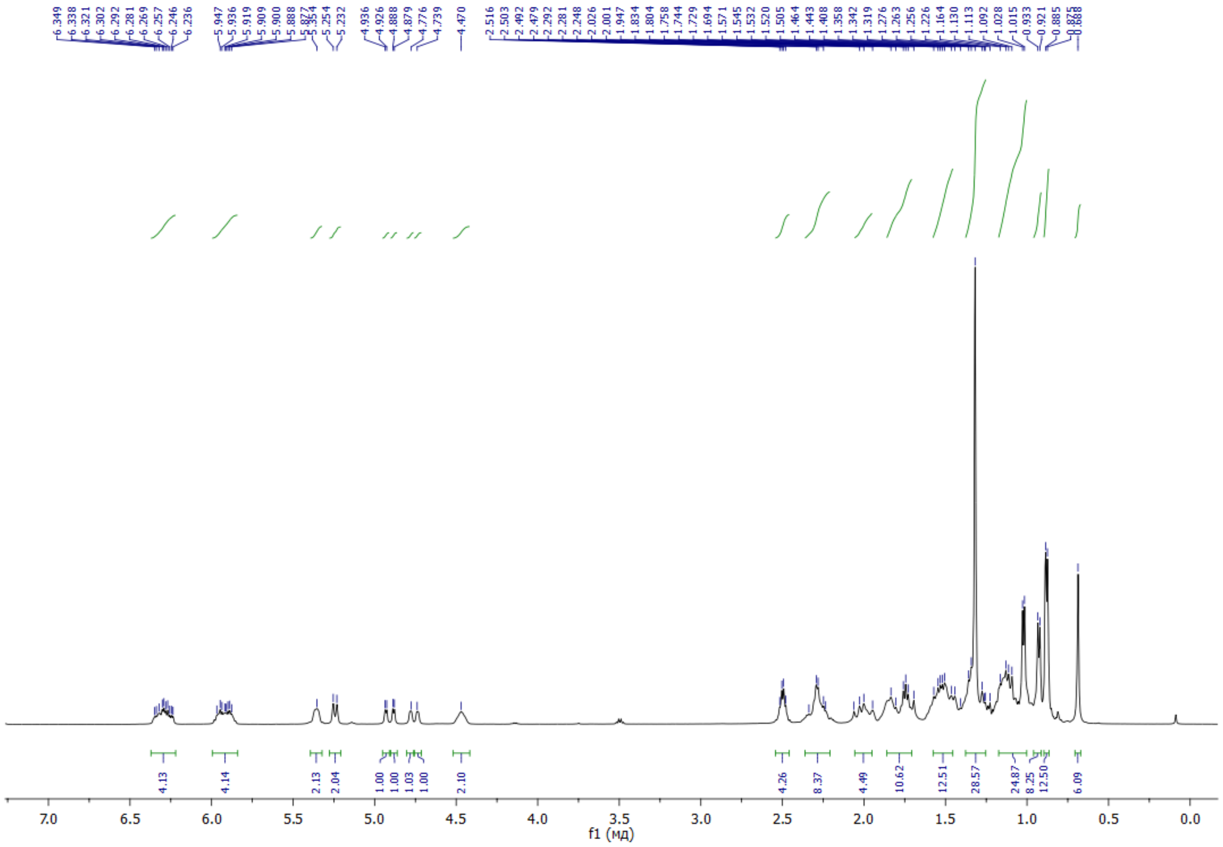

Figure S82. NOESY Spectrum of compound **4n** (500 MHz, $CDCl_3$)

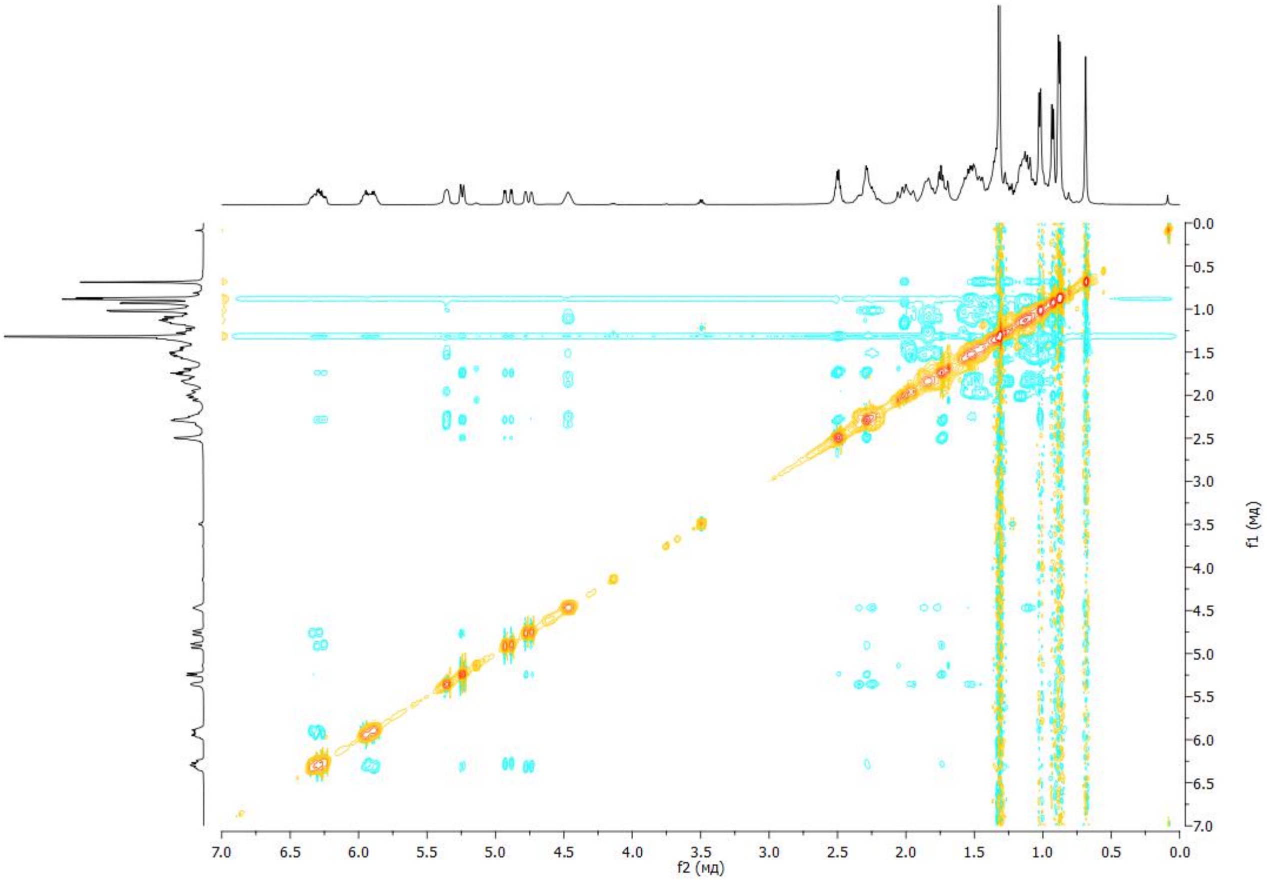

Figure S83. COSY Spectrum of compound **4n** (500 MHz, $CDCl_3$)

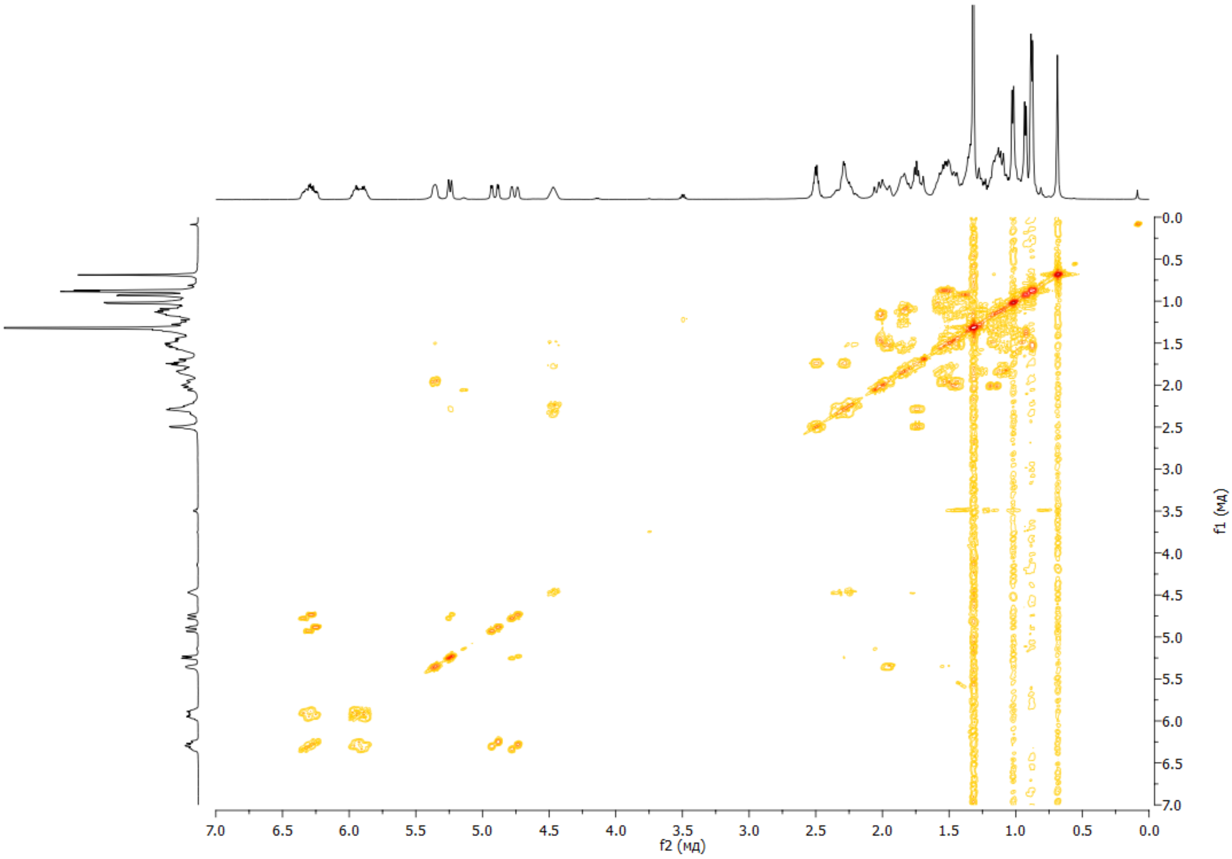

Figure S84. HSQC spectrum of compound **4n** (500 MHz, $CDCl_3$)

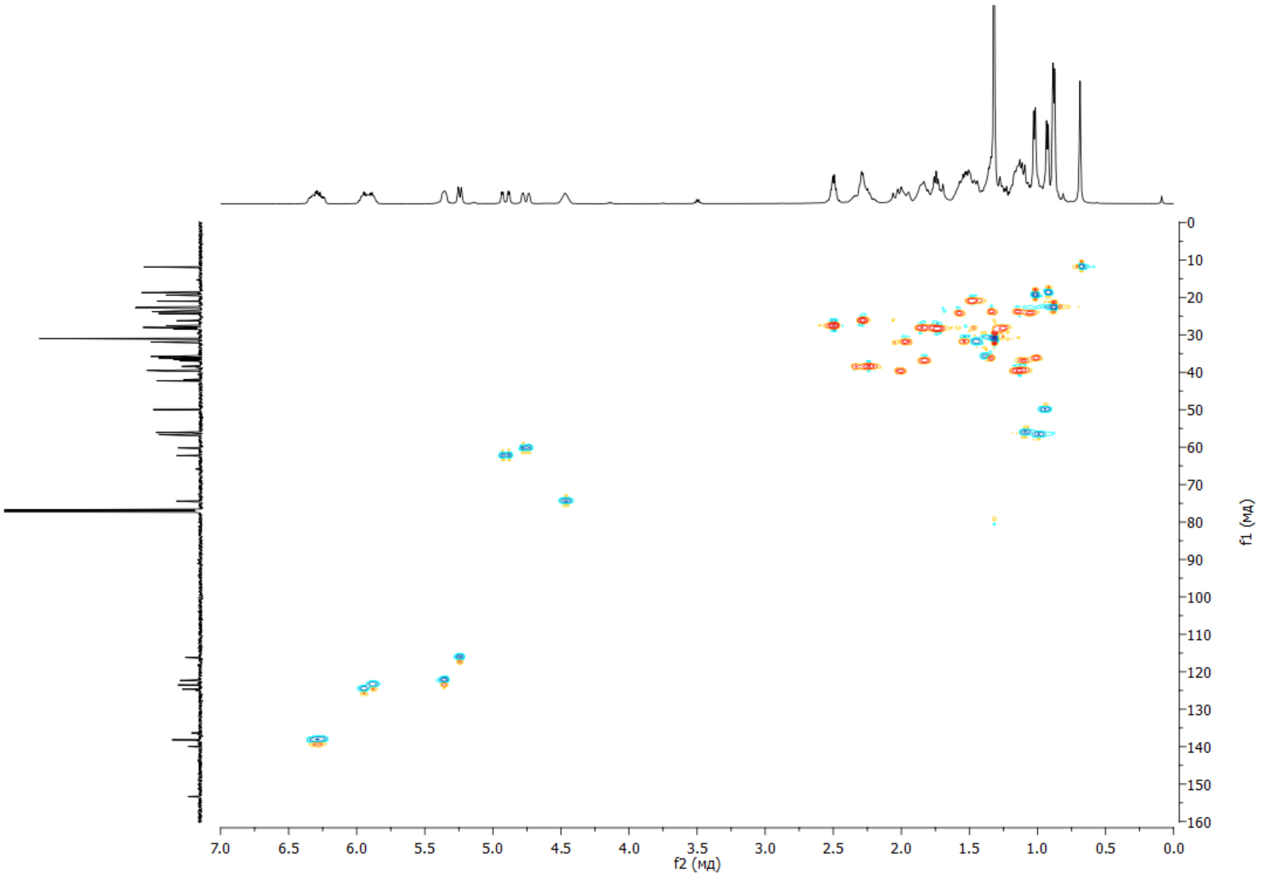

Figure S85. HMBC spectrum of compound **4n** (500 MHz, $CDCl_3$)

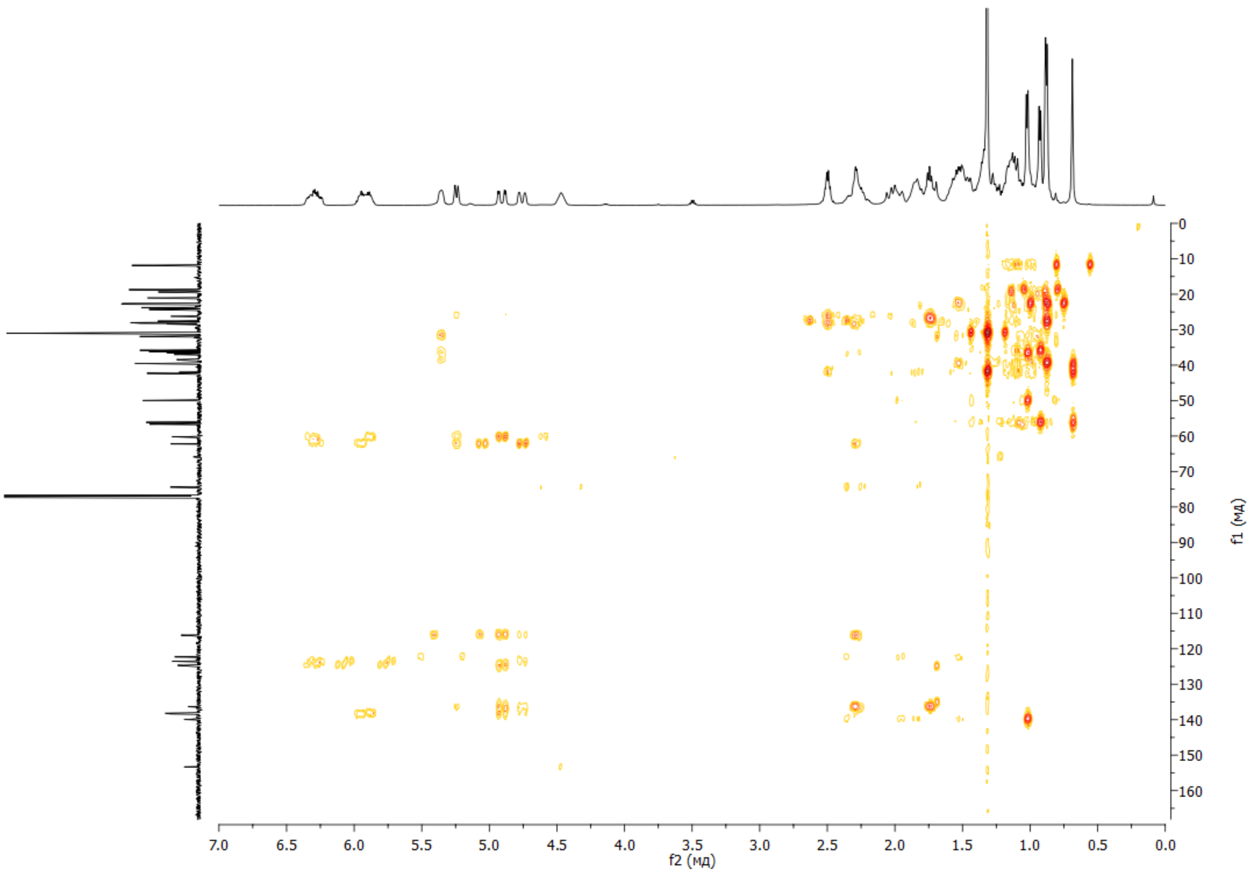

Figure S86. ^{13}C NMR Spectrum of compound **4o** (125 MHz, $CDCl_3$)

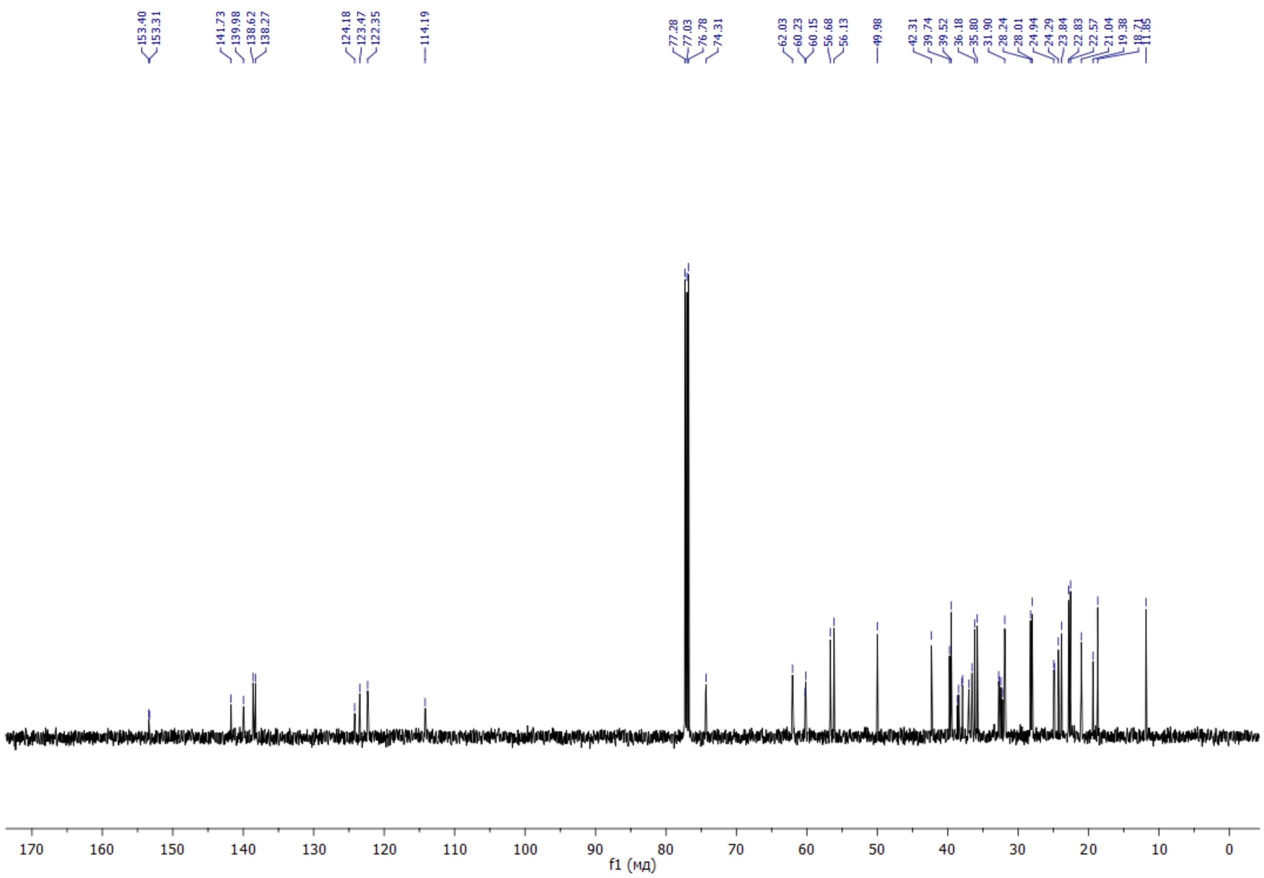

Figure S87. ^{1}H NMR Spectrum of compound **4o** (500 MHz, $CDCl_3$)

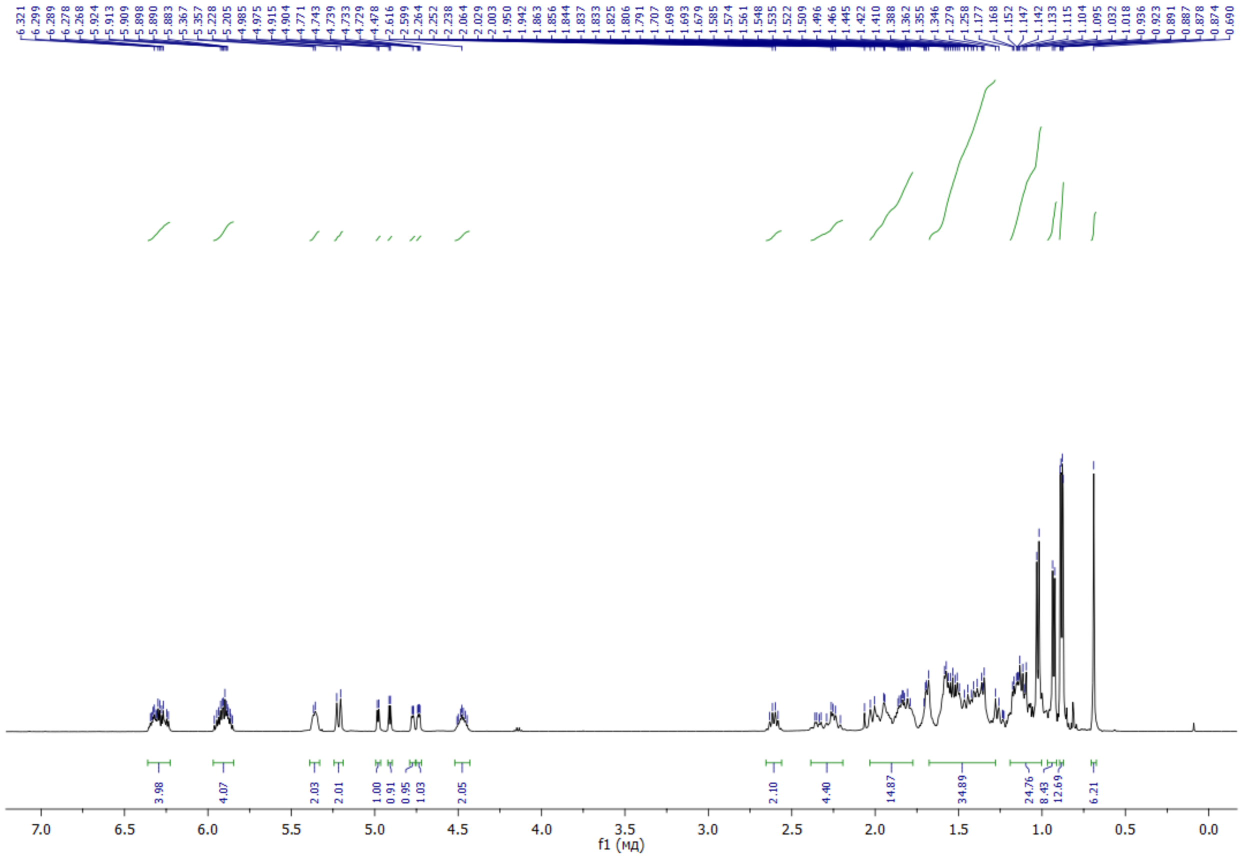

Figure S88. ^{13}C NMR Spectrum of compound **4p** (125 MHz, $CDCl_3$)

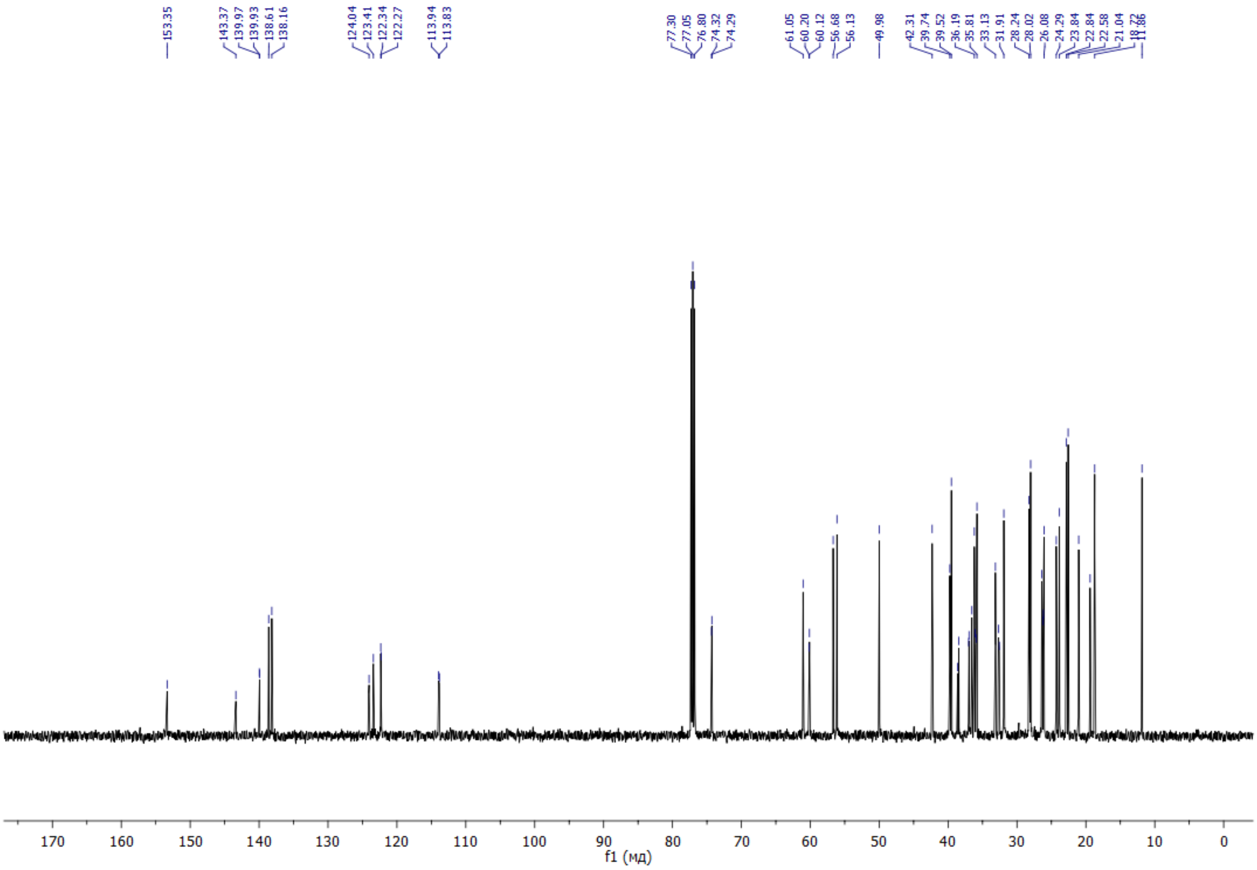

Figure S89. ^{1}H NMR Spectrum of compound **4p** (500 MHz, $CDCl_3$)

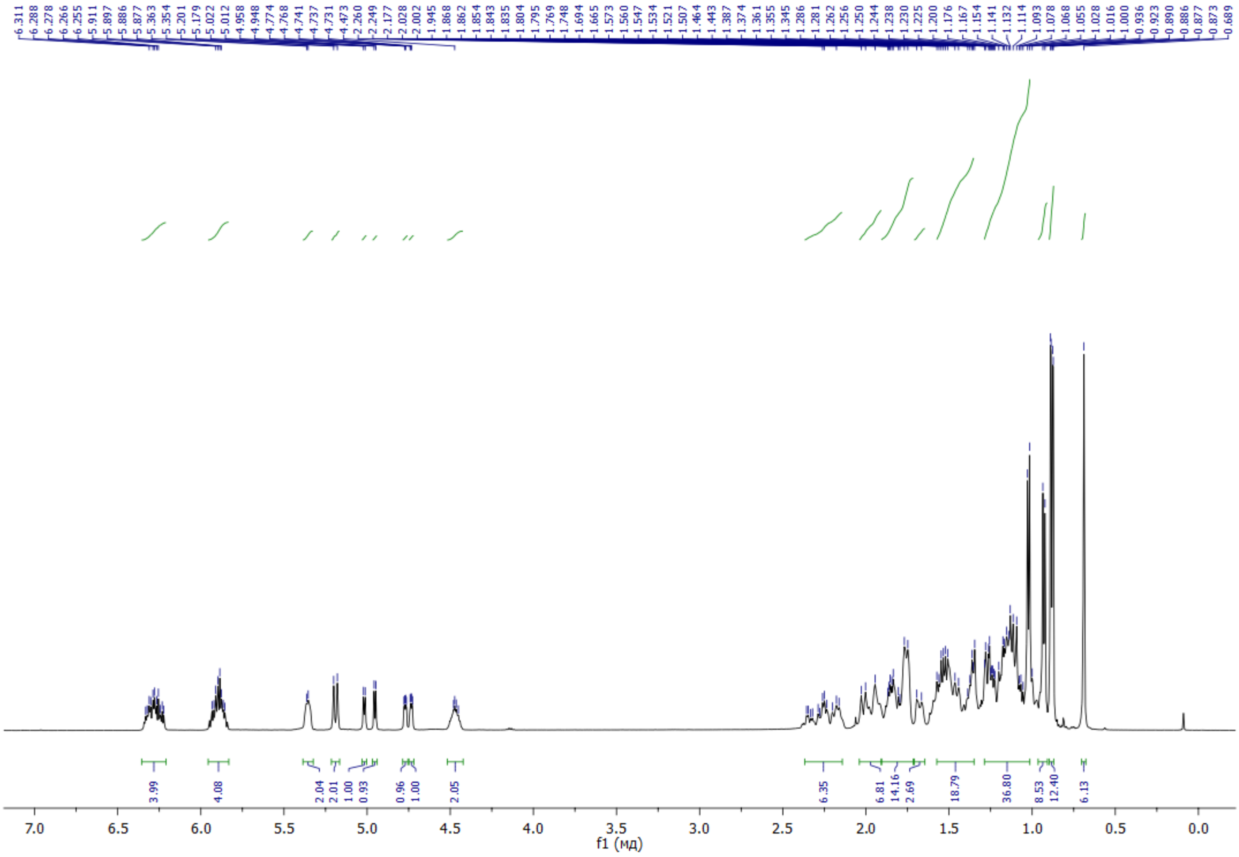

Figure S90. COSY Spectrum of compound **4p** (500 MHz, $CDCl_3$)

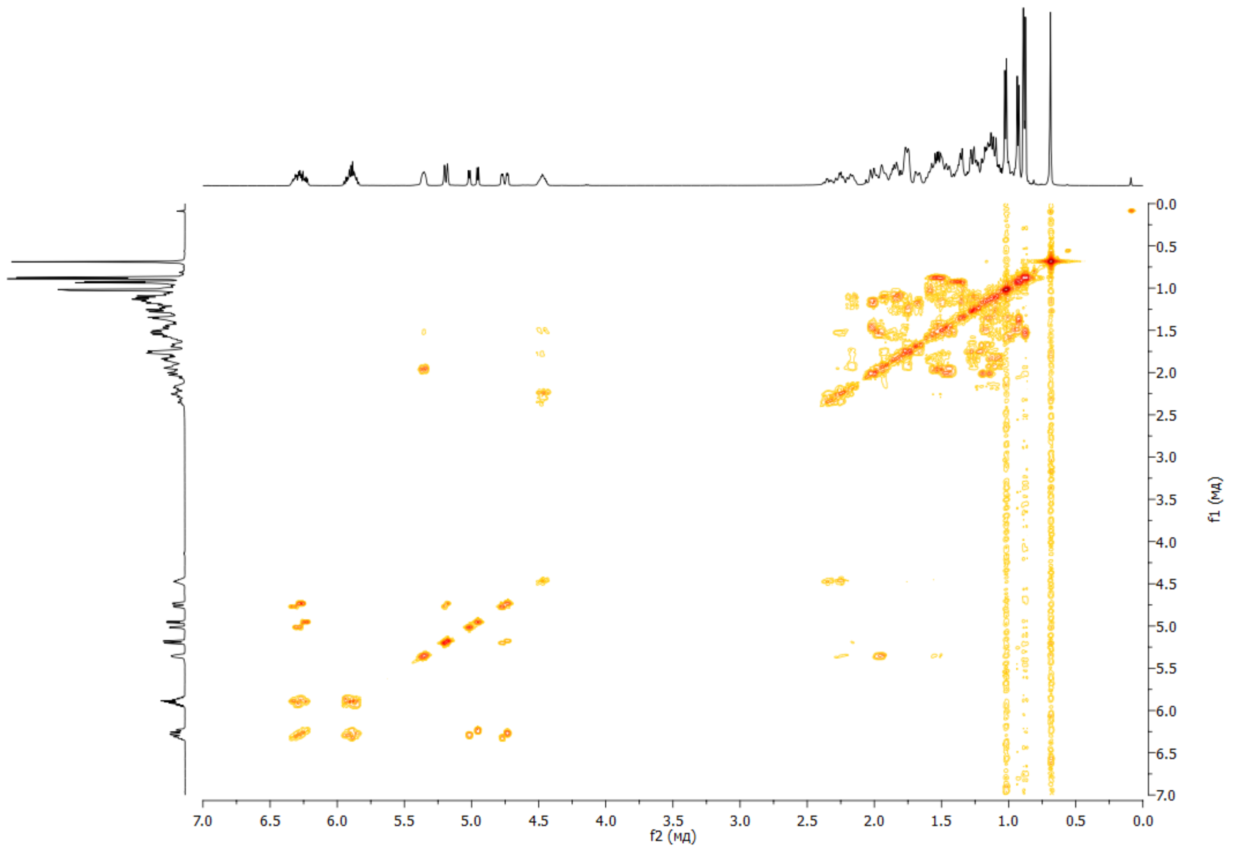

Figure S91. HSQC spectrum of compound **4p** (500 MHz, $CDCl_3$)

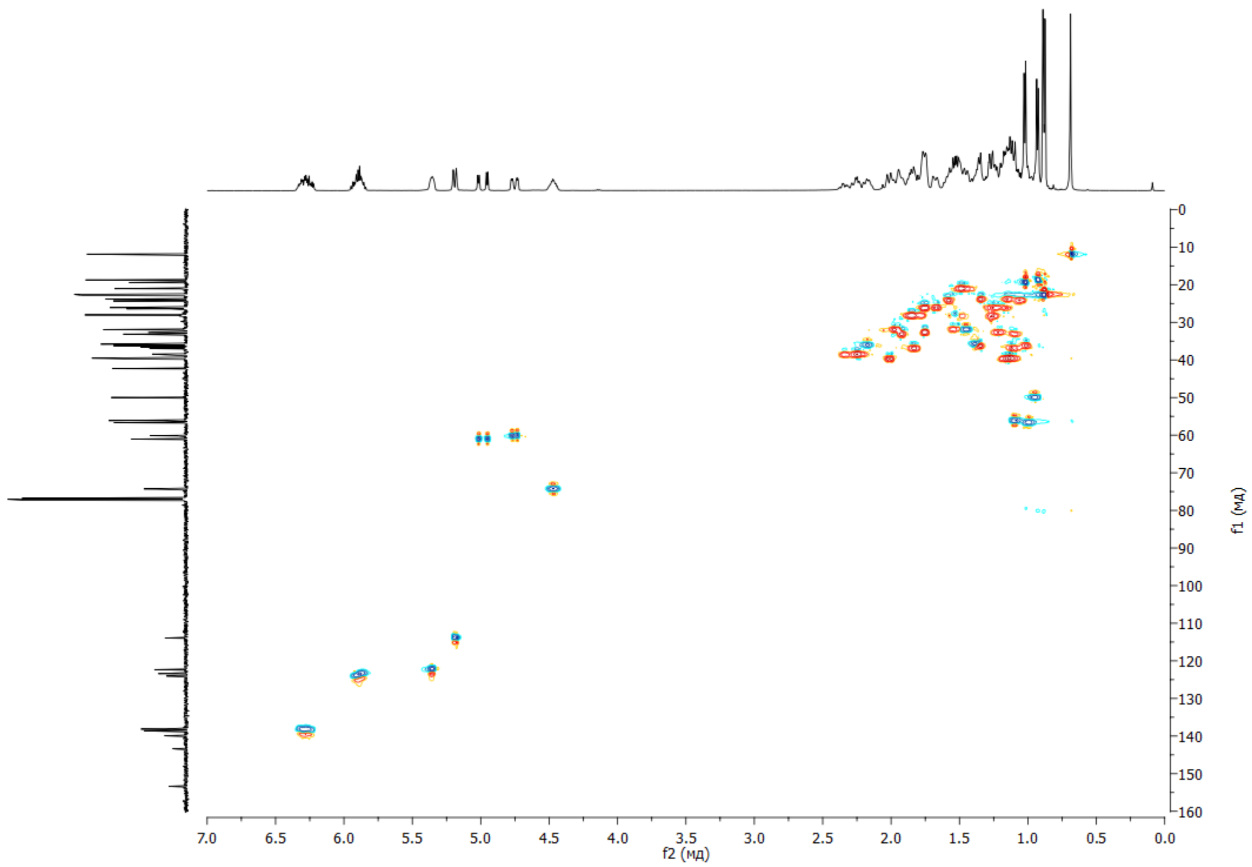

Figure S92. HMBC spectrum of compound **4p** (500 MHz, $CDCl_3$)

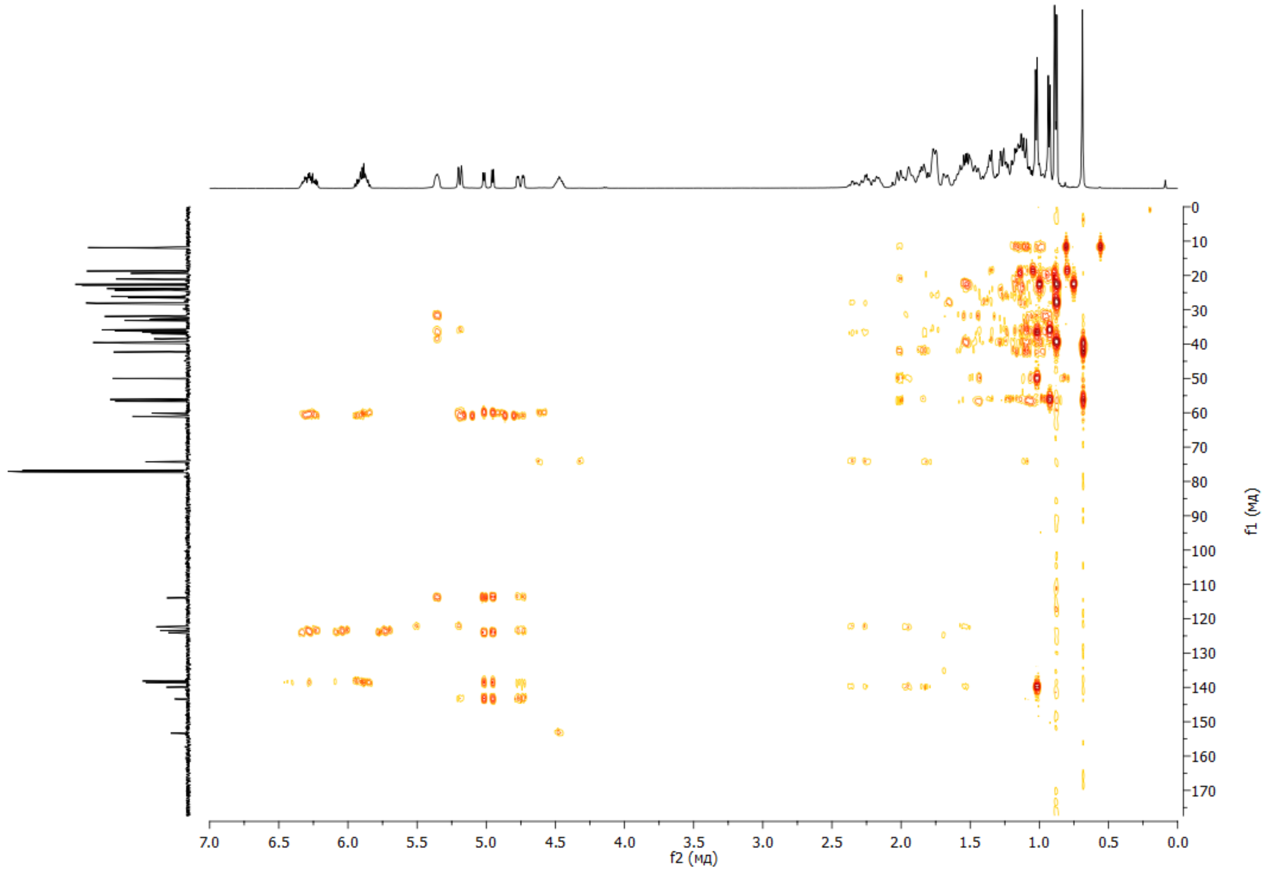

Figure S93. ^{13}C NMR Spectrum of compound **4q** (125 MHz, $CDCl_3$)

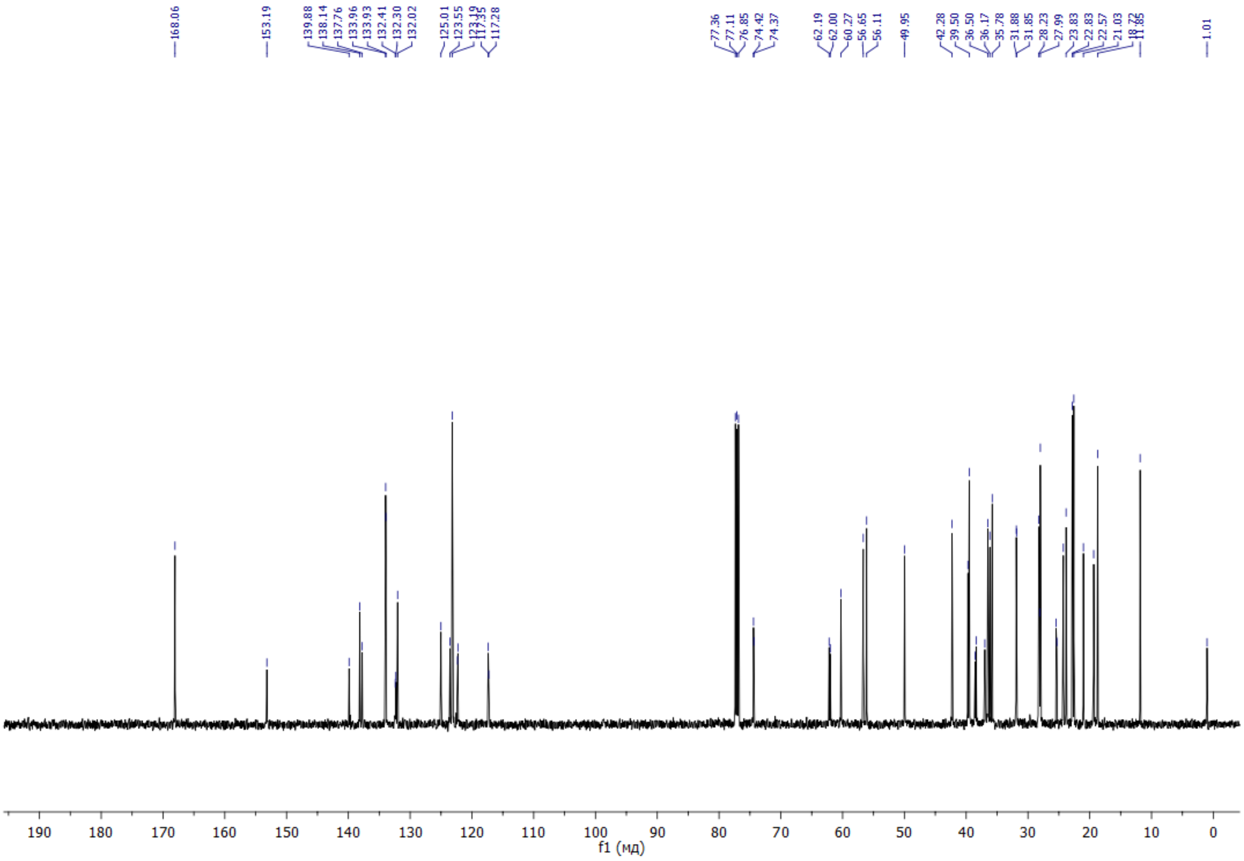

Figure S94. ^{1}H NMR Spectrum of compound **4q** (500 MHz, $CDCl_3$)

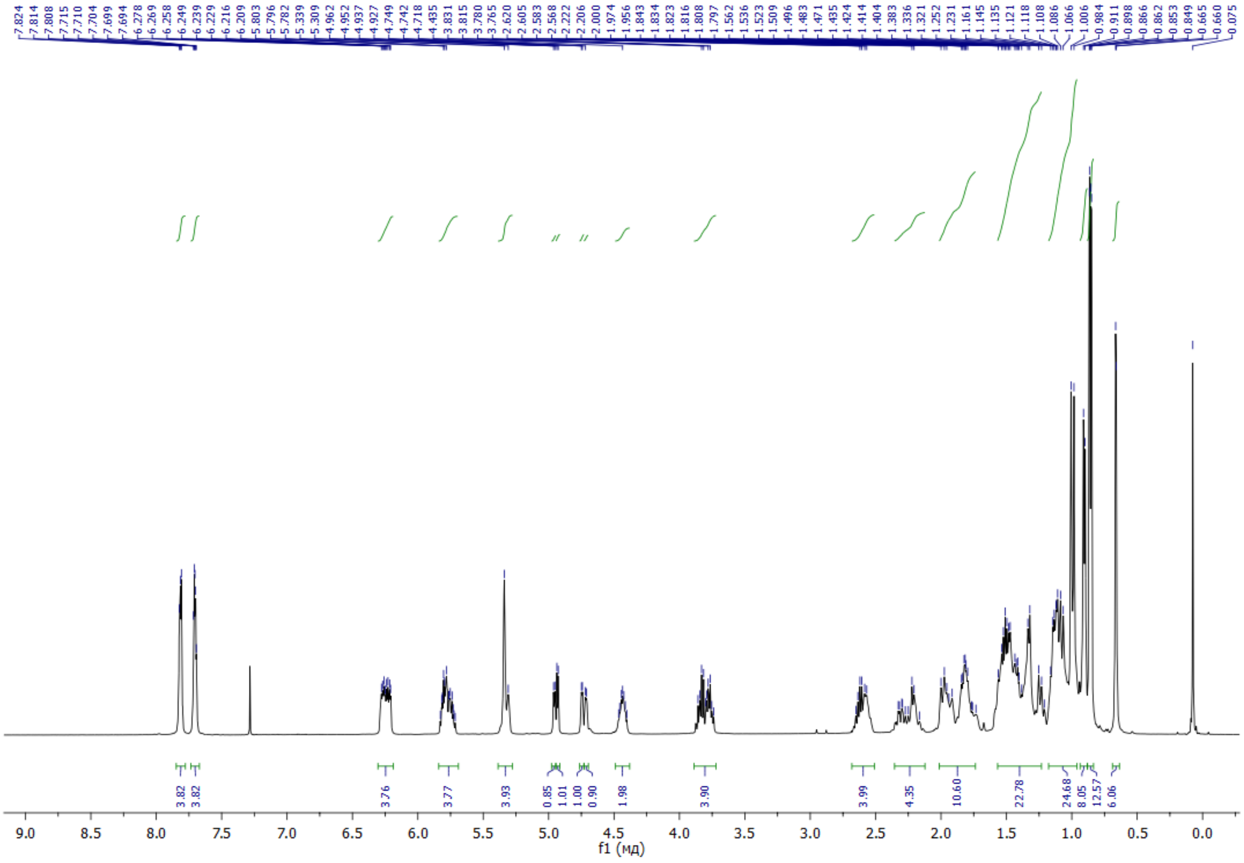

Figure S95. COSY Spectrum of compound **4q** (500 MHz, $CDCl_3$)

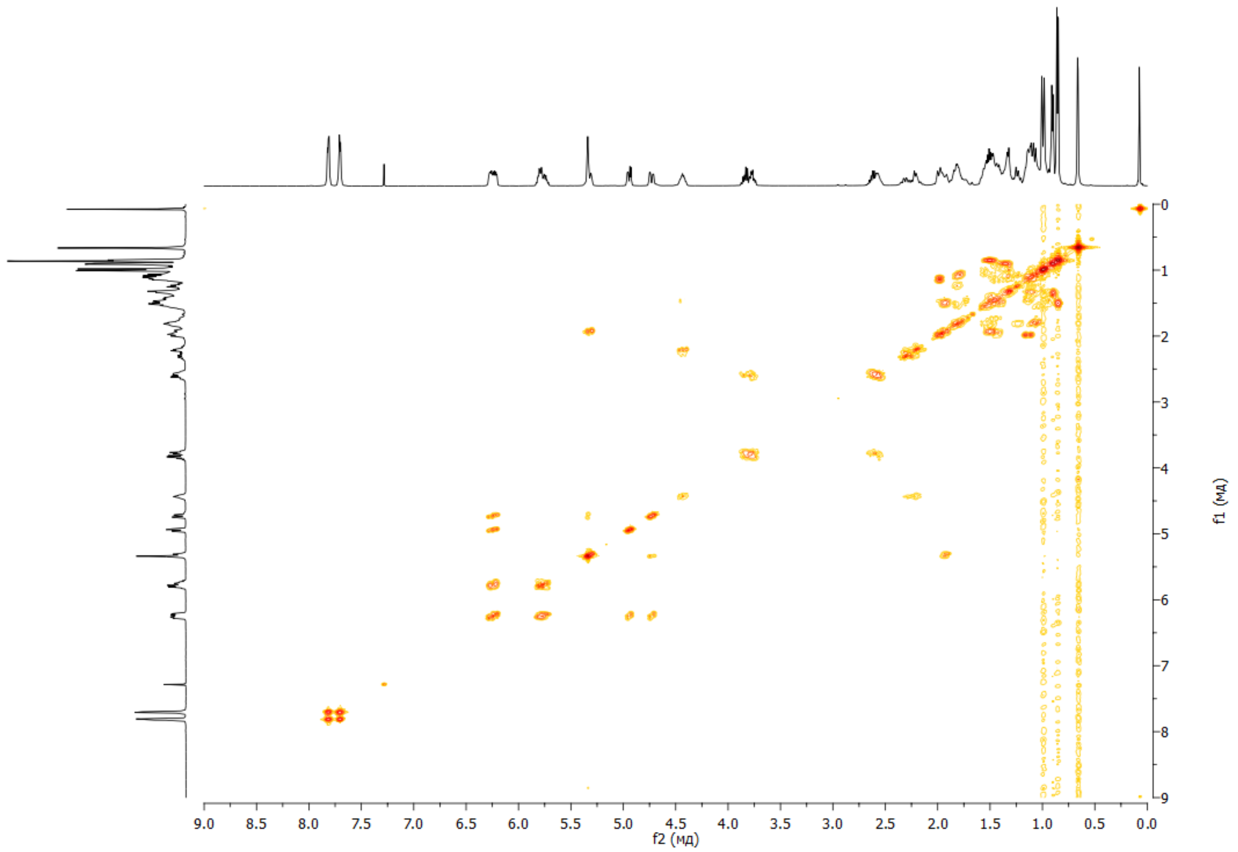

Figure S96. HSQC spectrum of compound **4q** (500 MHz, $CDCl_3$)

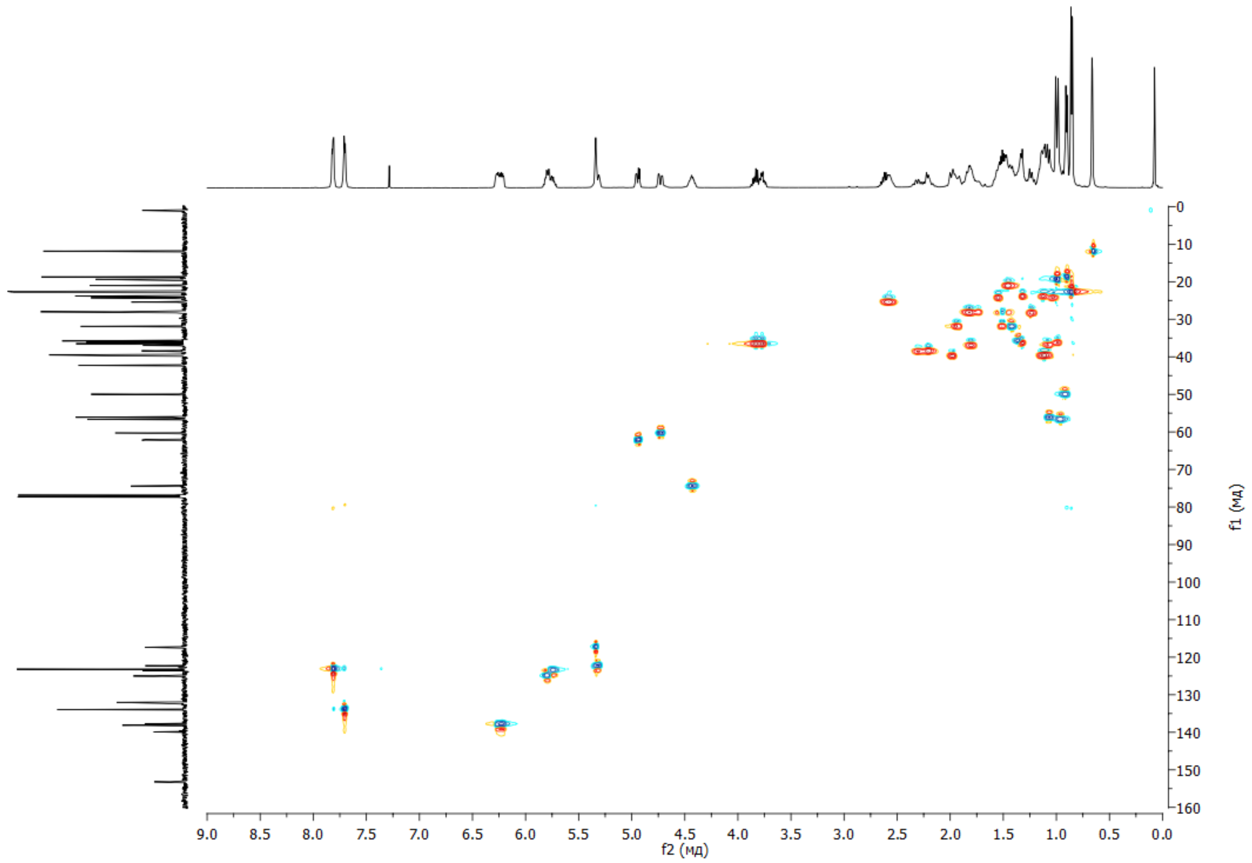

Figure S97. HMBC spectrum of compound **4q** (500 MHz, $CDCl_3$)

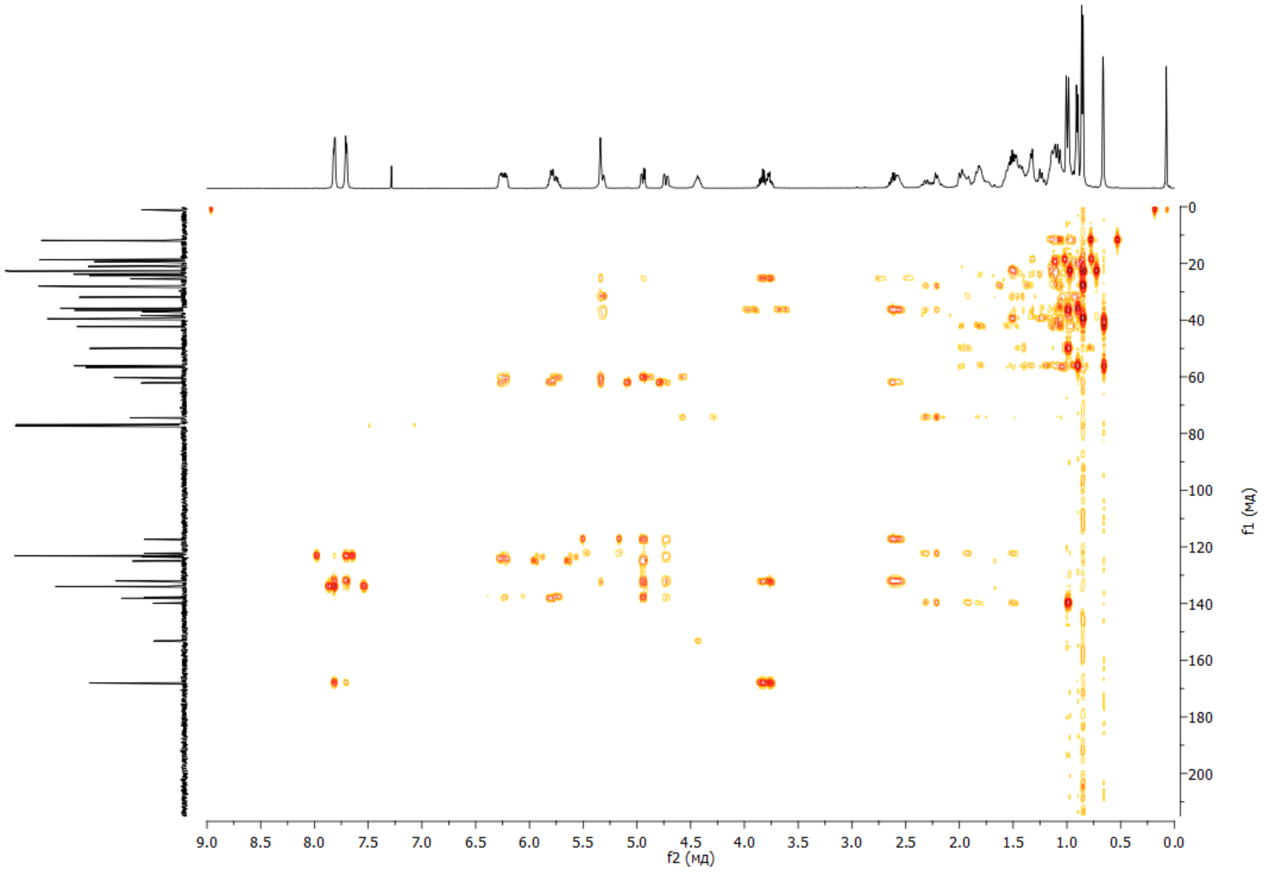

Figure S98. ^{13}C NMR Spectrum of compound **4r** (125 MHz, $CDCl_3$)

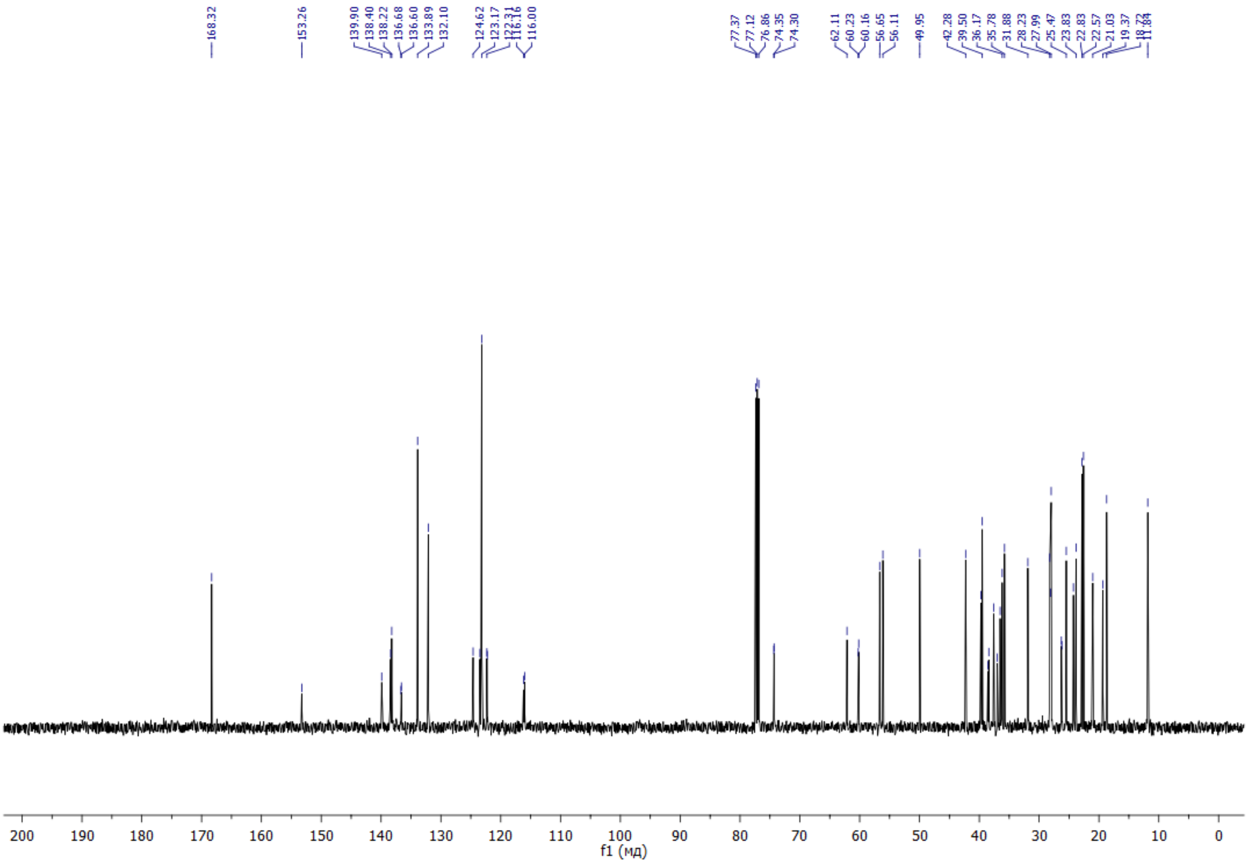

Figure S99. ^{1}H NMR Spectrum of compound **4r** (500 MHz, $CDCl_3$)

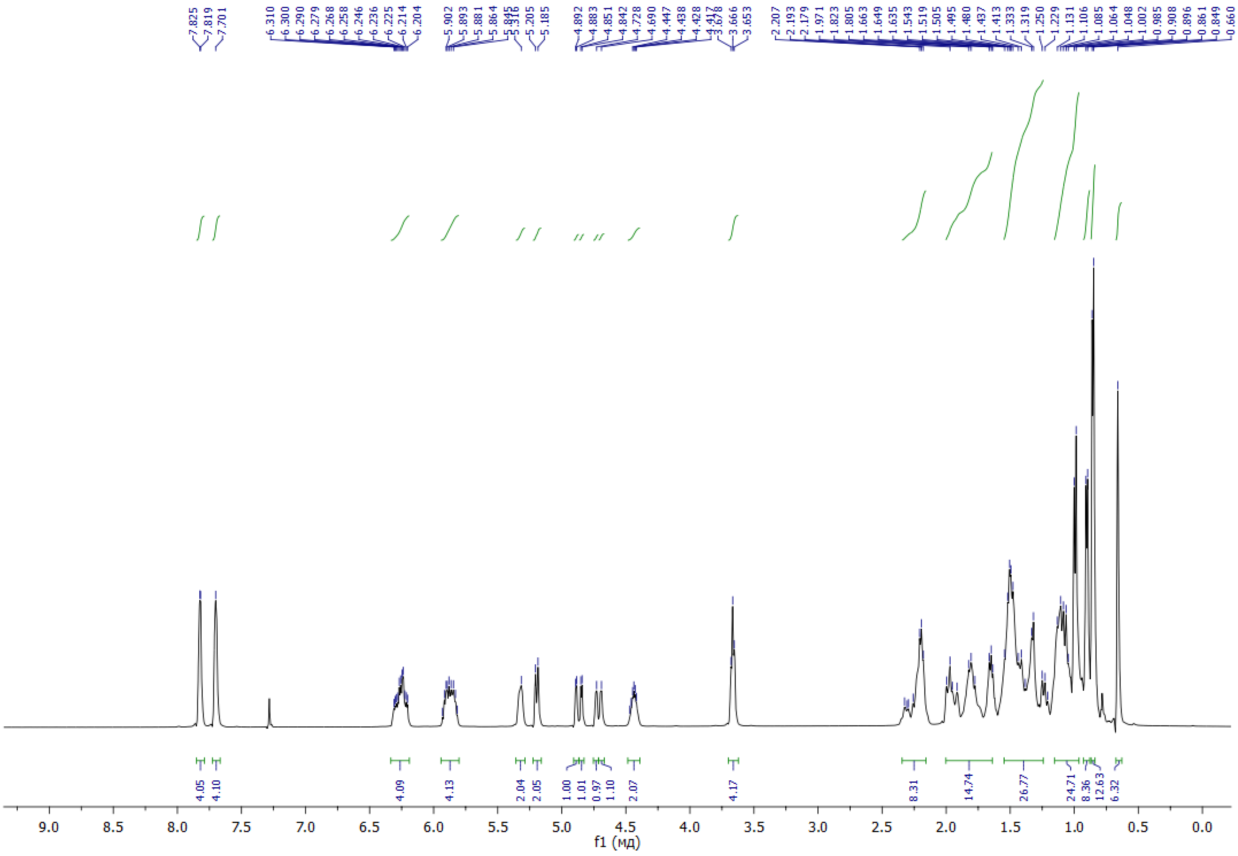

Figure S100. COSY Spectrum of compound **4r** (500 MHz, $CDCl_3$)

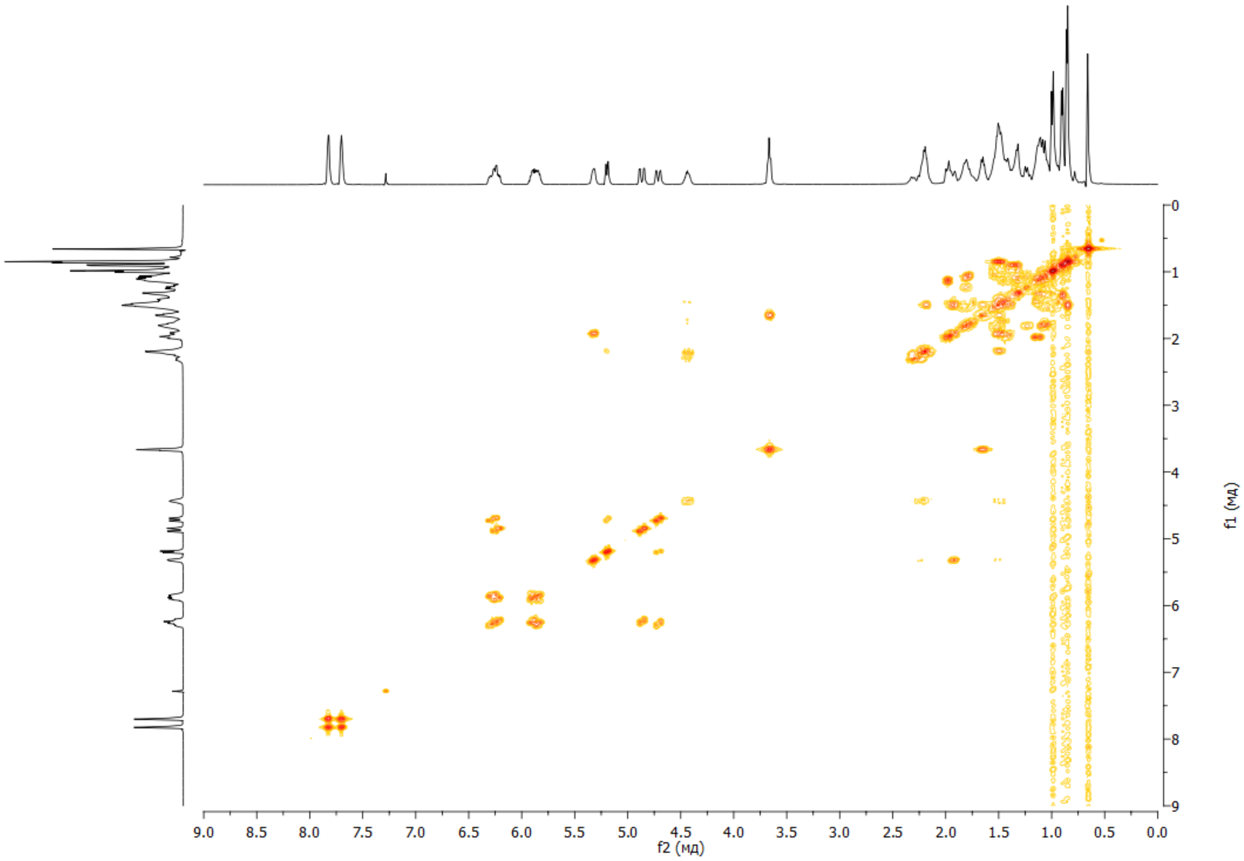

Figure S101. HSQC spectrum of compound **4r** (500 MHz, $CDCl_3$)

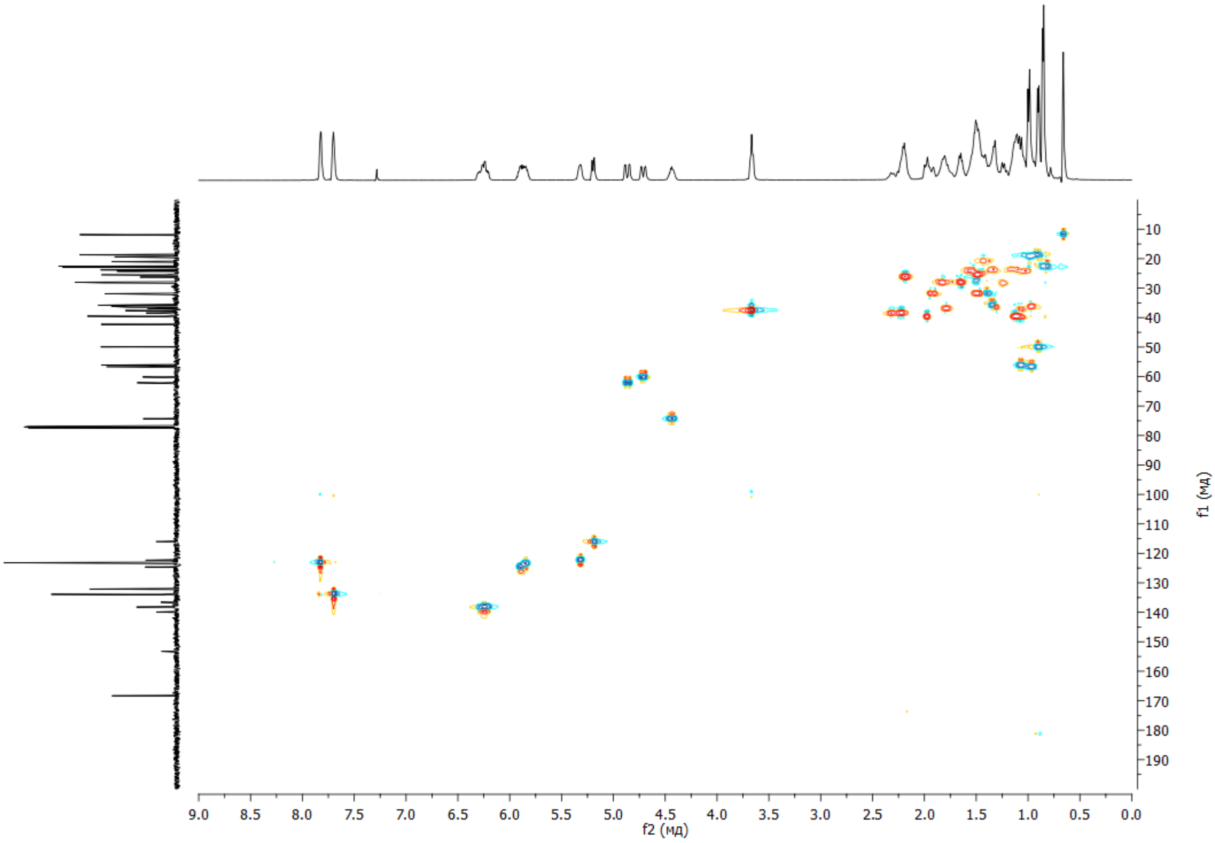

Figure S102. HMBC spectrum of compound **4r** (500 MHz, $CDCl_3$)

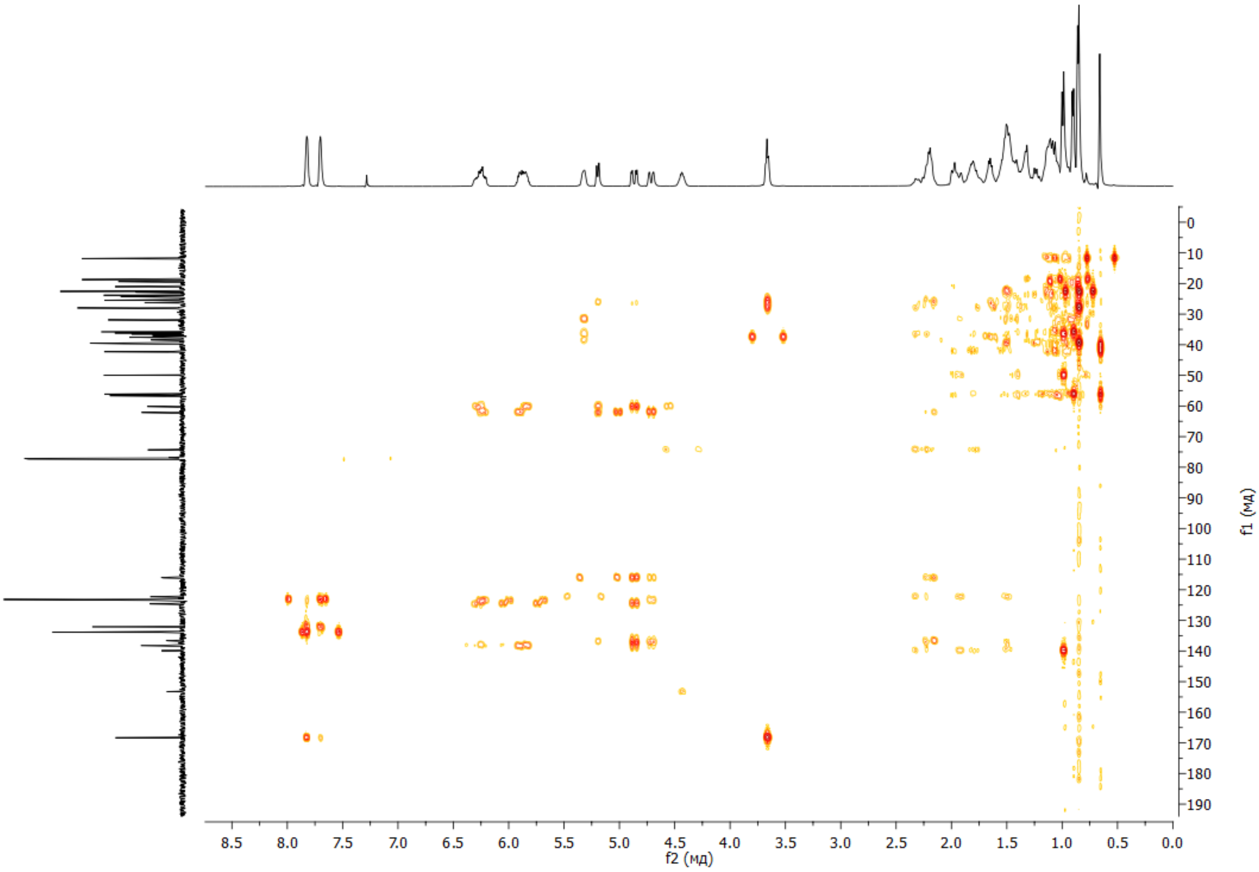

Figure S103. ^{13}C NMR Spectrum of compound **4s** (125 MHz, $CDCl_3$)

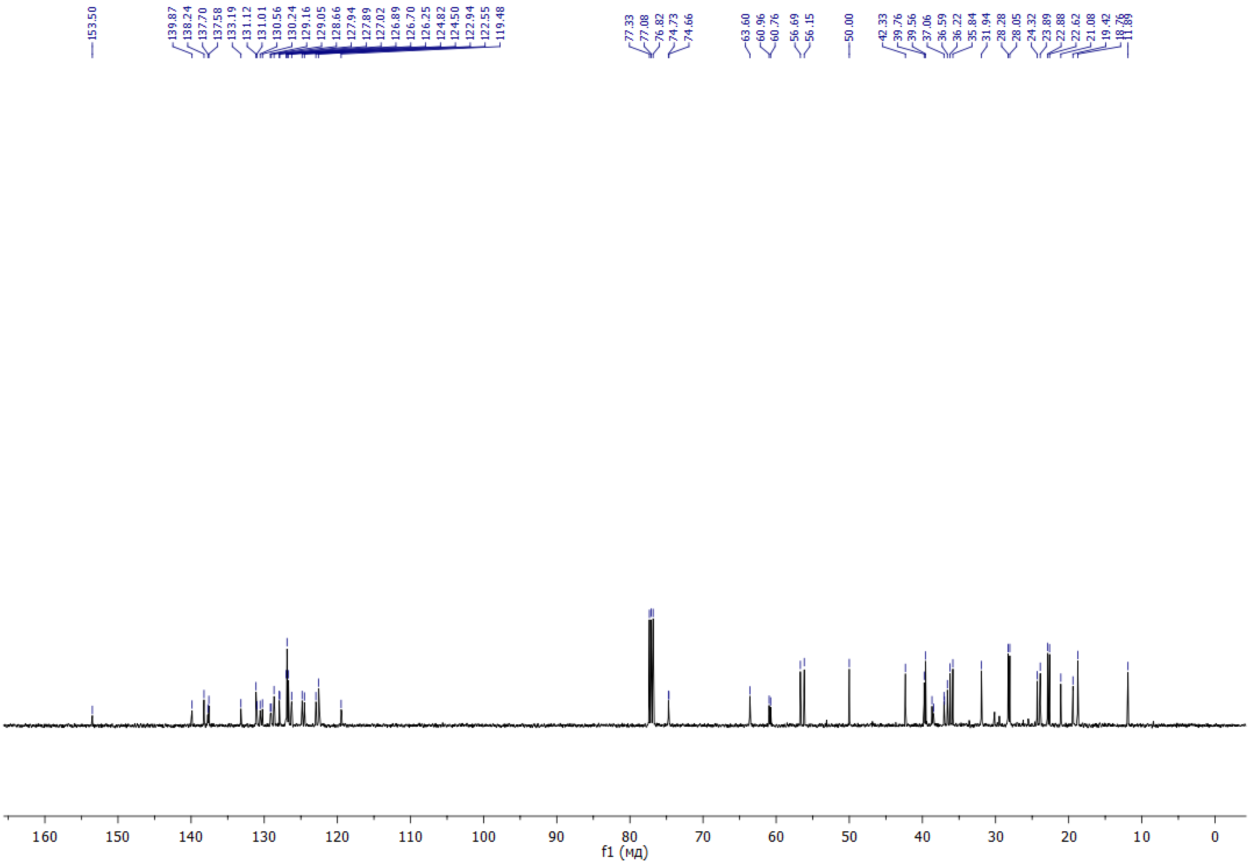

Figure S104. ^{1}H NMR Spectrum of compound **4s** (500 MHz, $CDCl_3$)

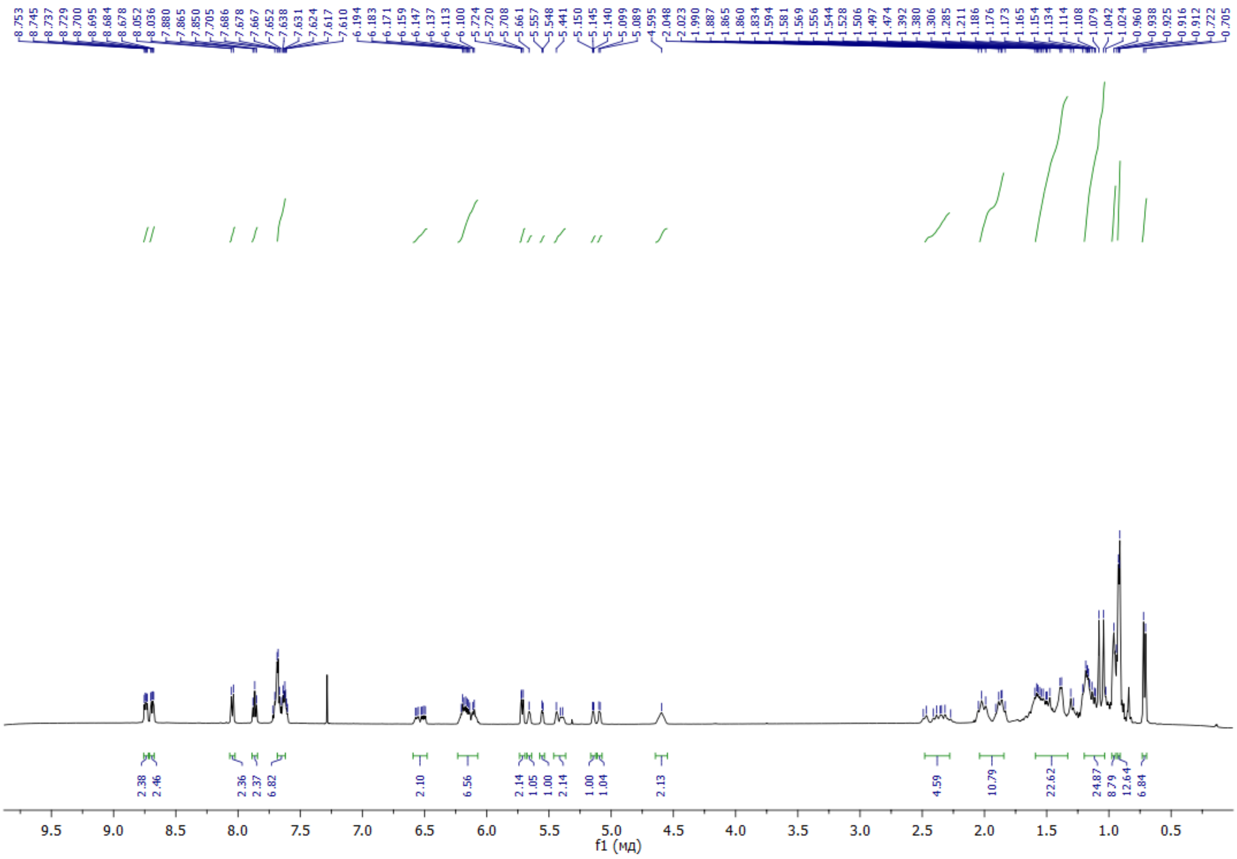

Figure S105. COSY Spectrum of compound **4s** (500 MHz, $CDCl_3$)

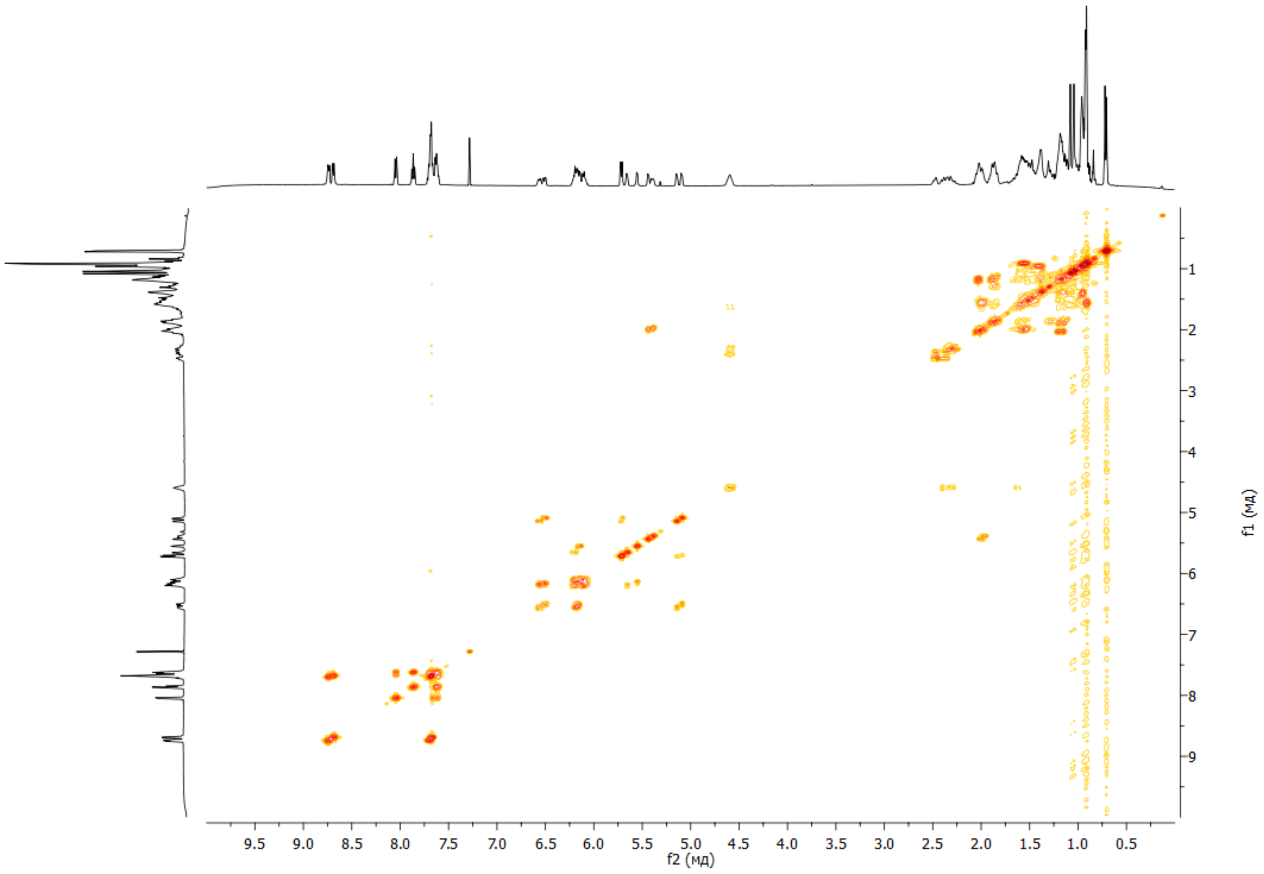

Figure S106. HSQC spectrum of compound **4s** (500 MHz, $CDCl_3$)

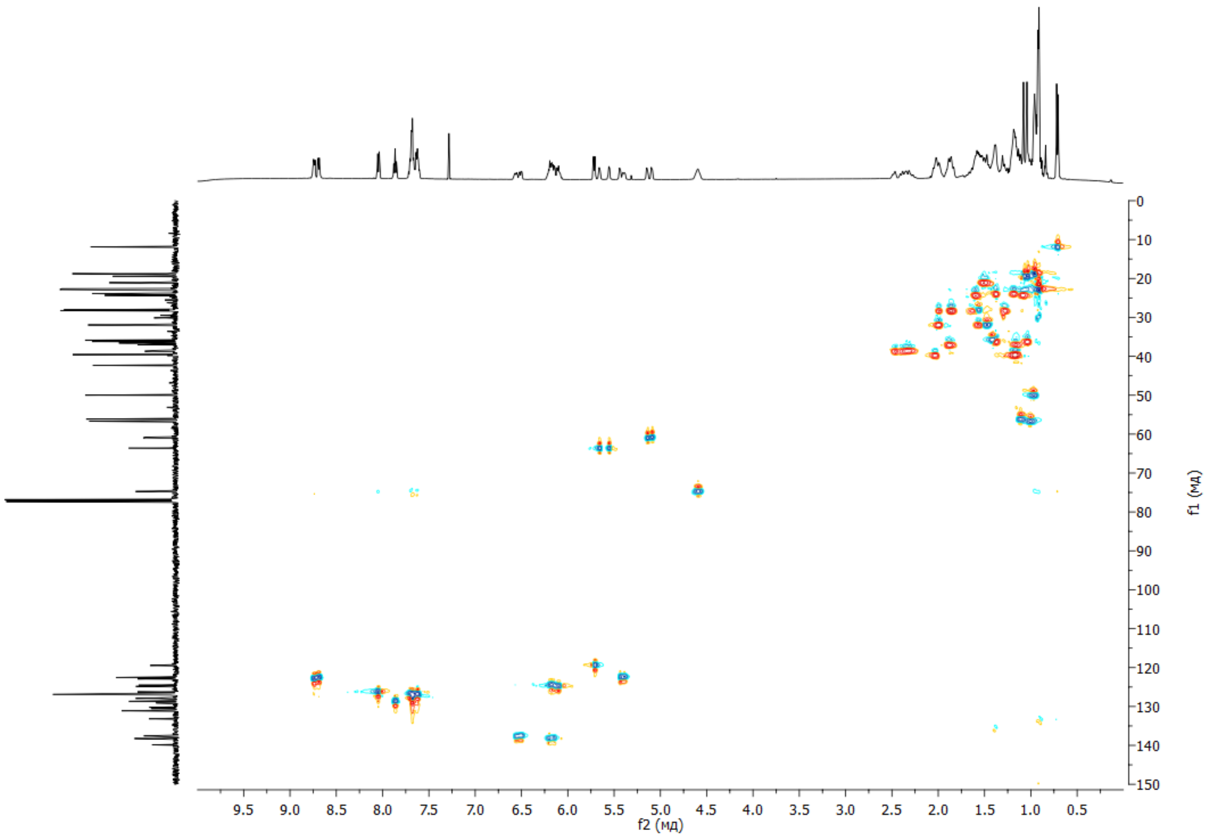

Figure S107. HMBC spectrum of compound **4s** (500 MHz, $CDCl_3$)

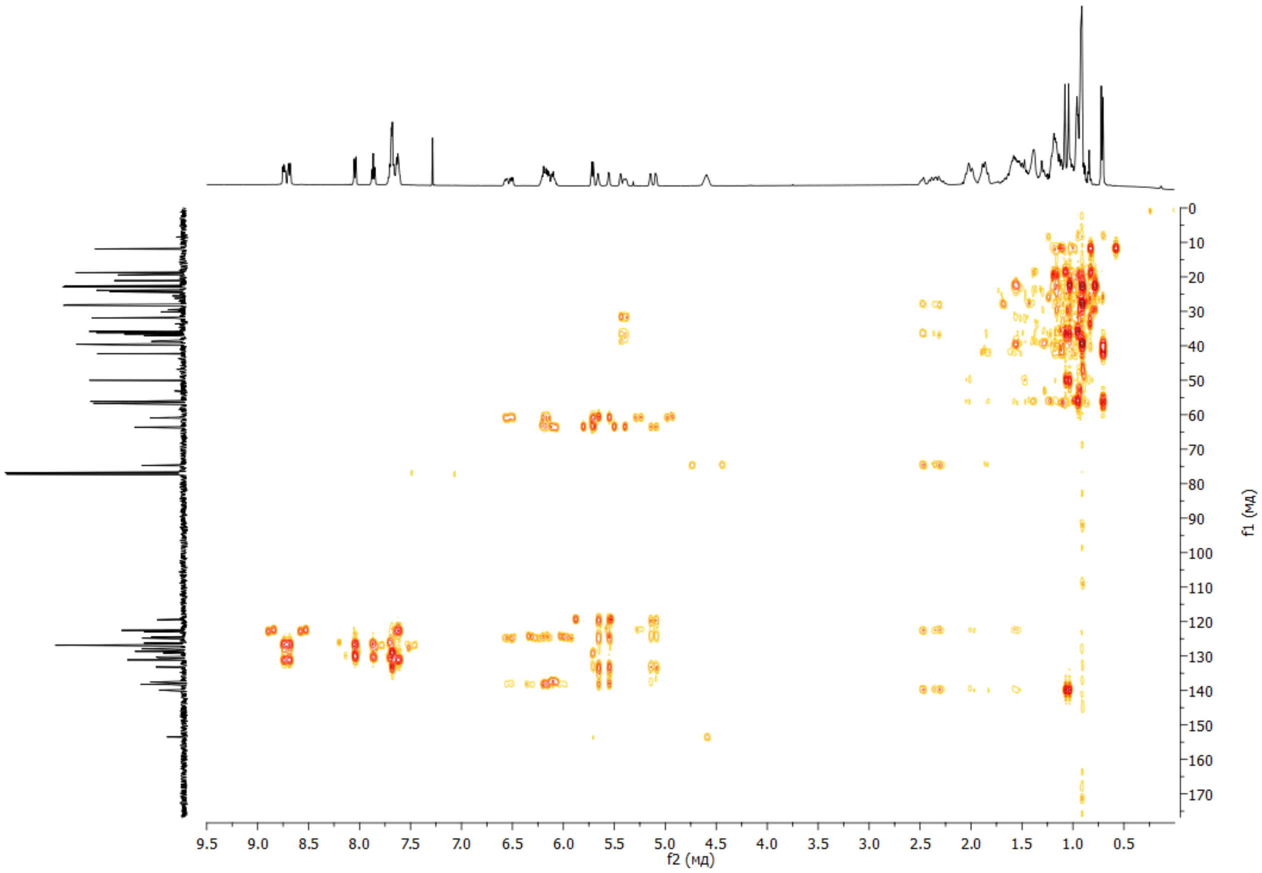

Figure S108. ^{13}C NMR Spectrum of compound **4t** (125 MHz, $CDCl_3$)

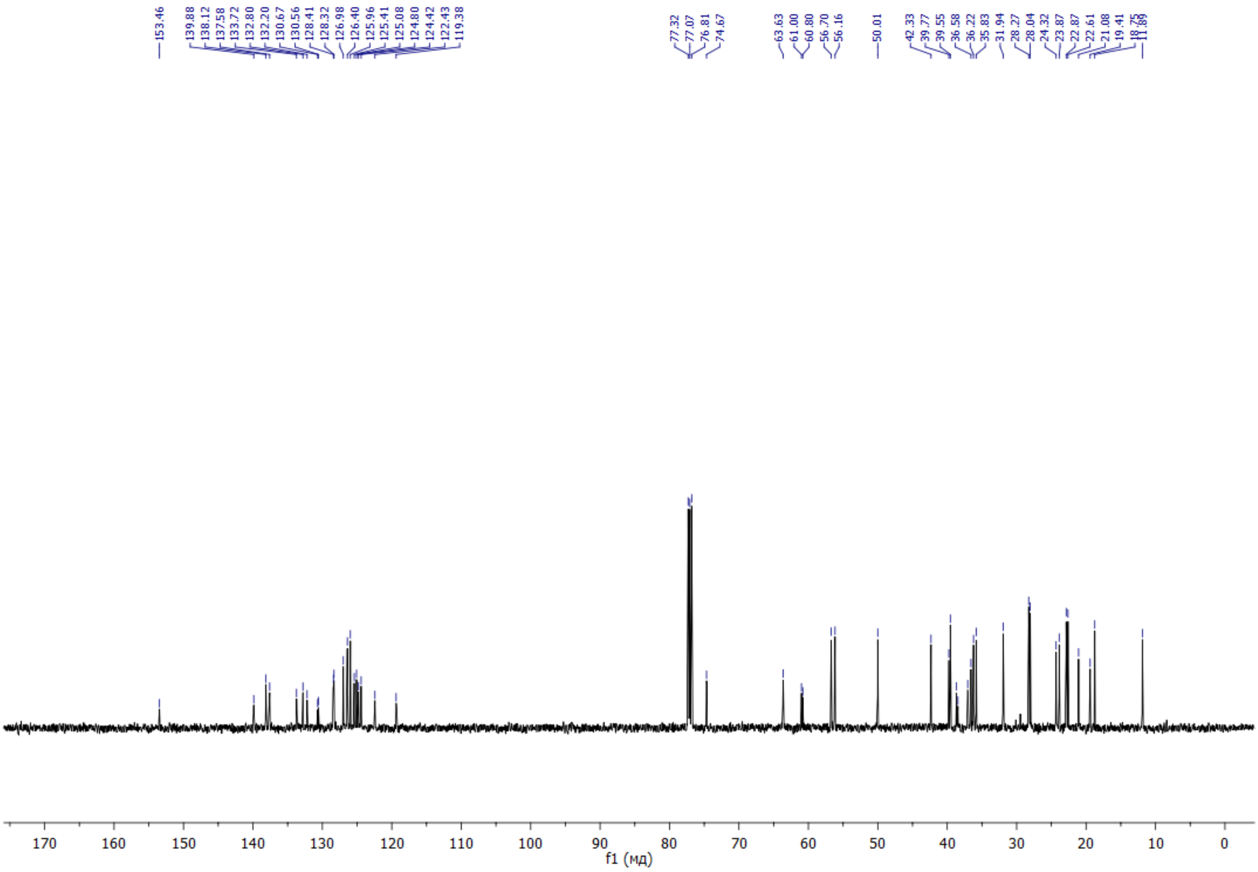

Figure S109. ^{1}H NMR Spectrum of compound **4t** (500 MHz, $CDCl_3$)

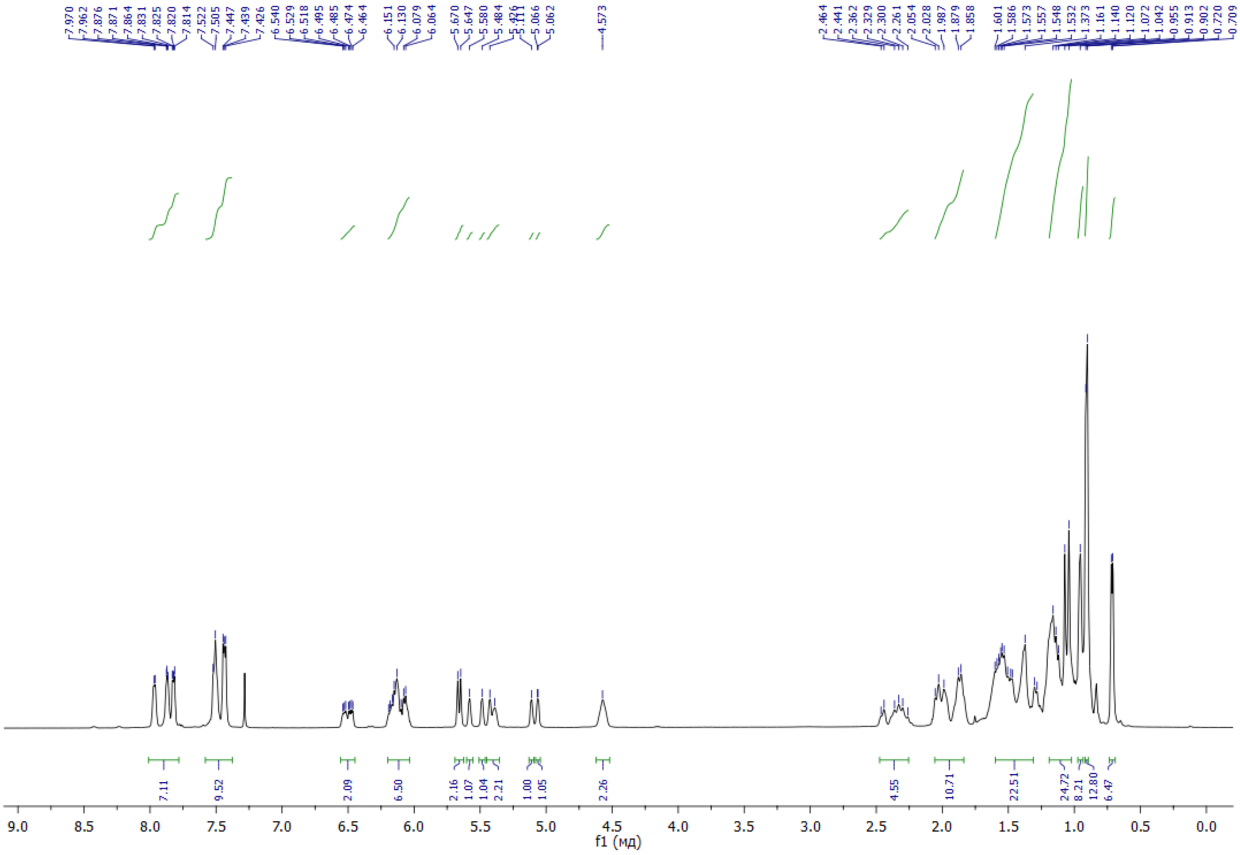

Figure S110. COSY Spectrum of compound **4t** (500 MHz, $CDCl_3$)

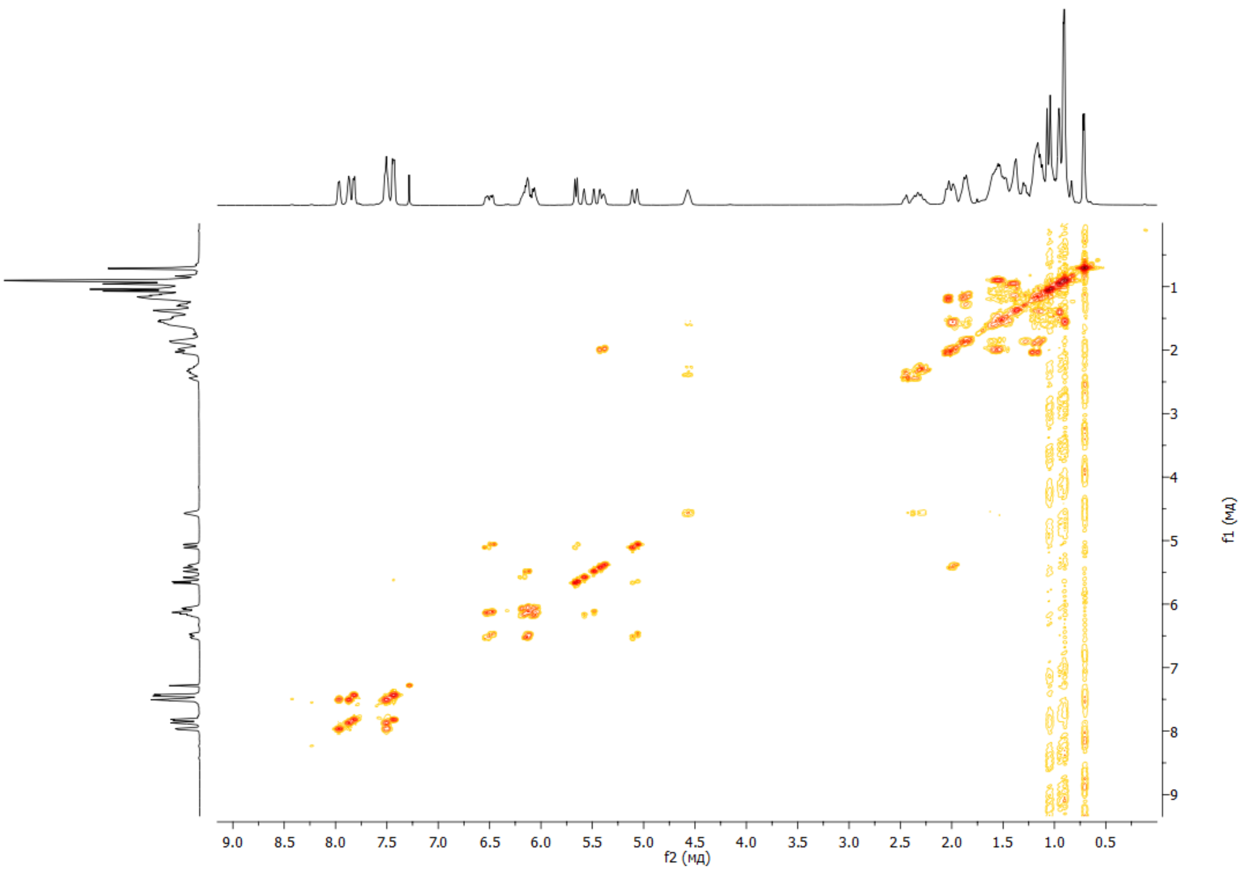

Figure S111. HSQC spectrum of compound **4t** (500 MHz, $CDCl_3$)

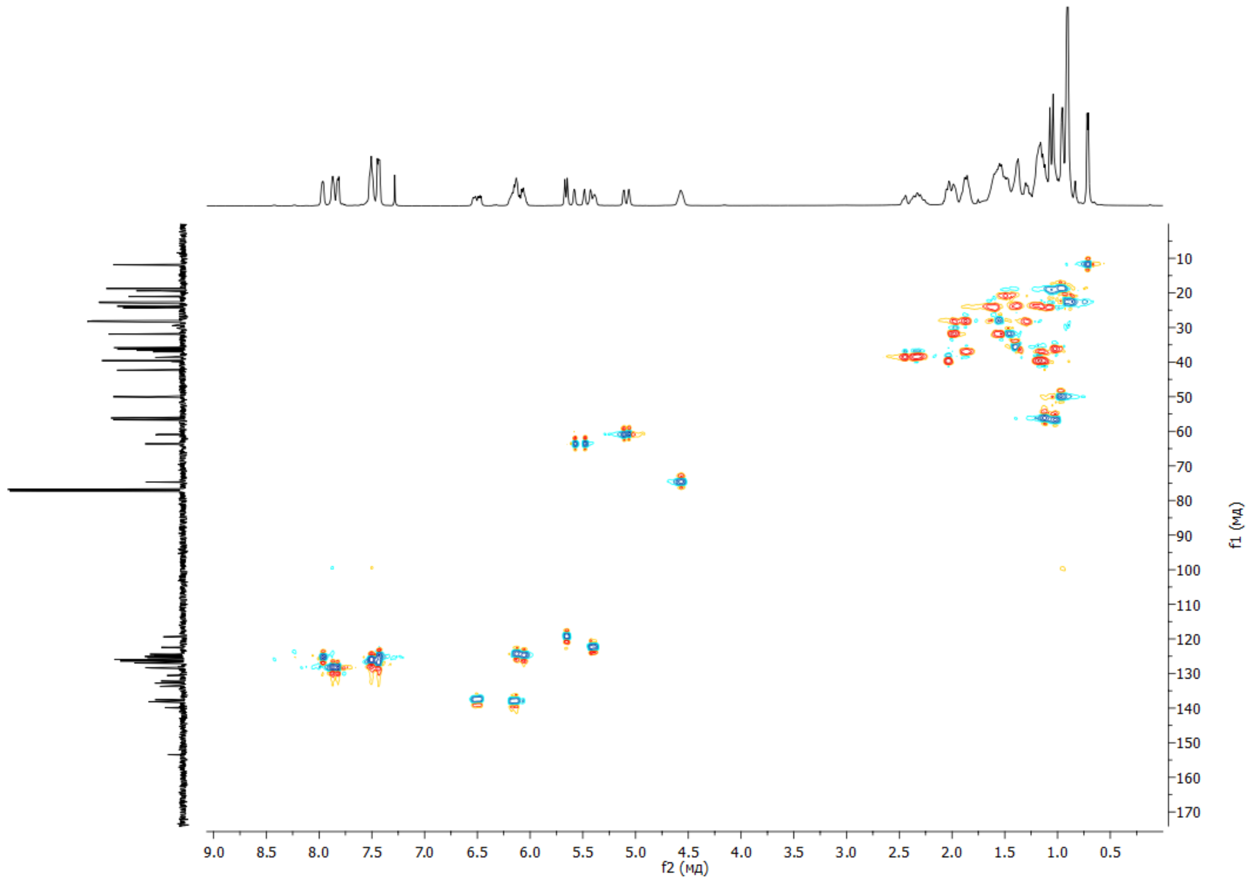

Figure S112. HMBC spectrum of compound **4t** (500 MHz, $CDCl_3$)

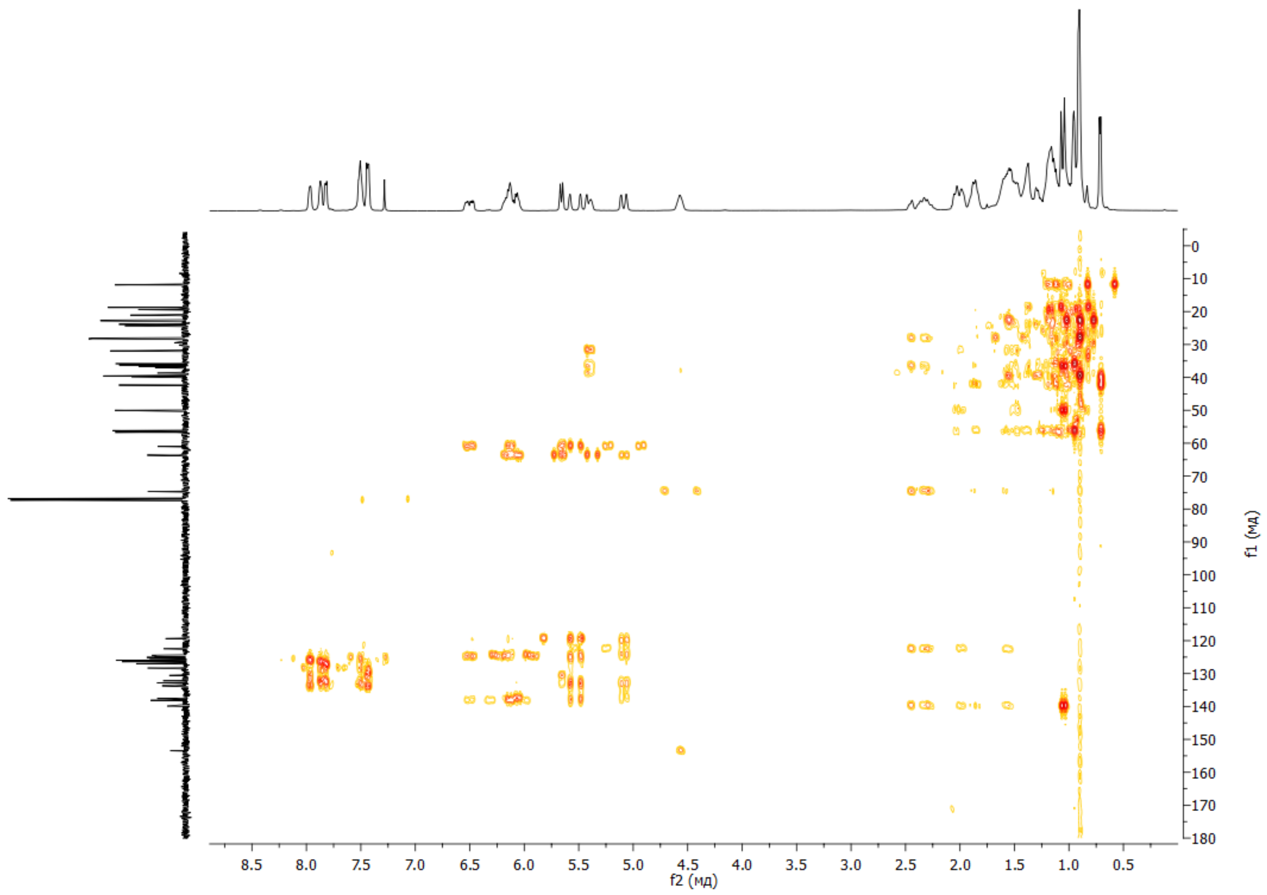

Figure S113. ^{13}C NMR Spectrum of compound **6** (125 MHz, $CDCl_3$)

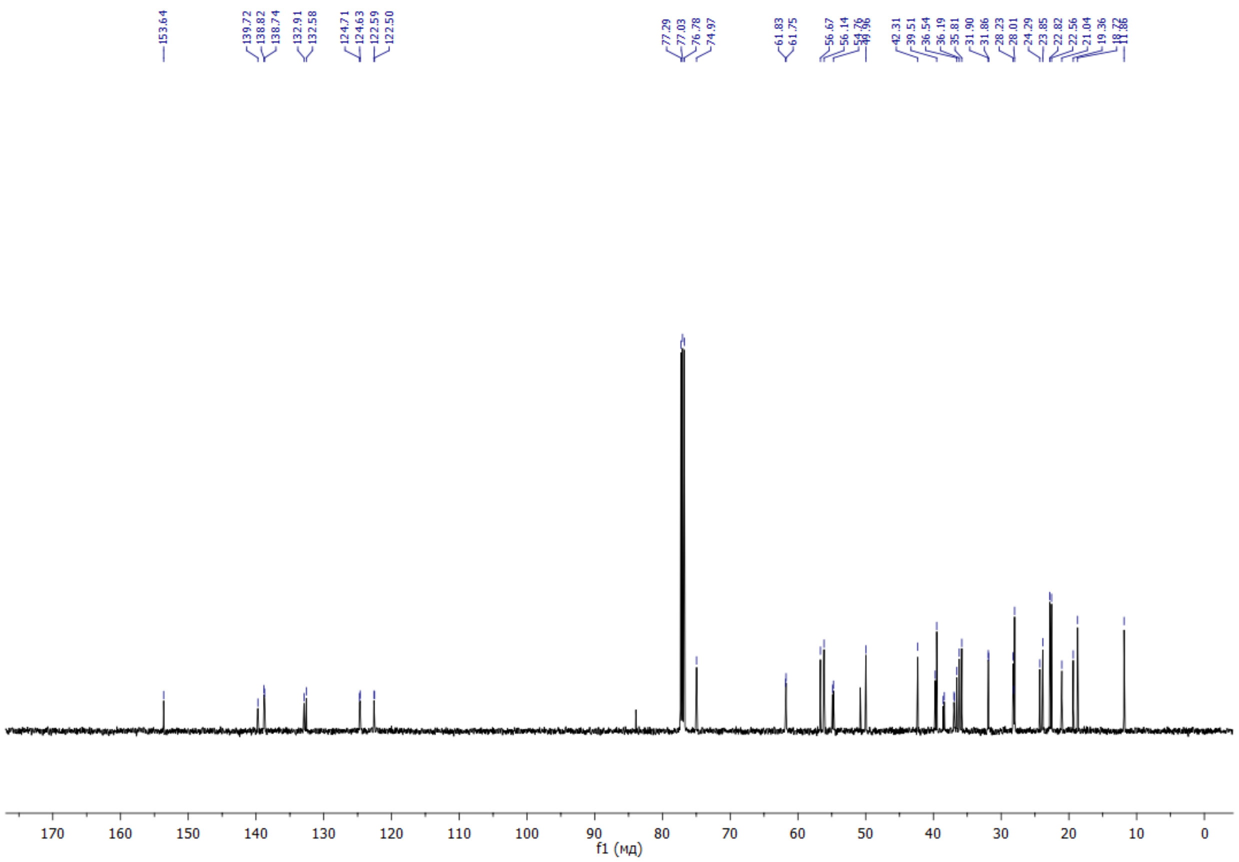

Figure S114. ^{1}H NMR Spectrum of compound **6** (500 MHz, $CDCl_3$)

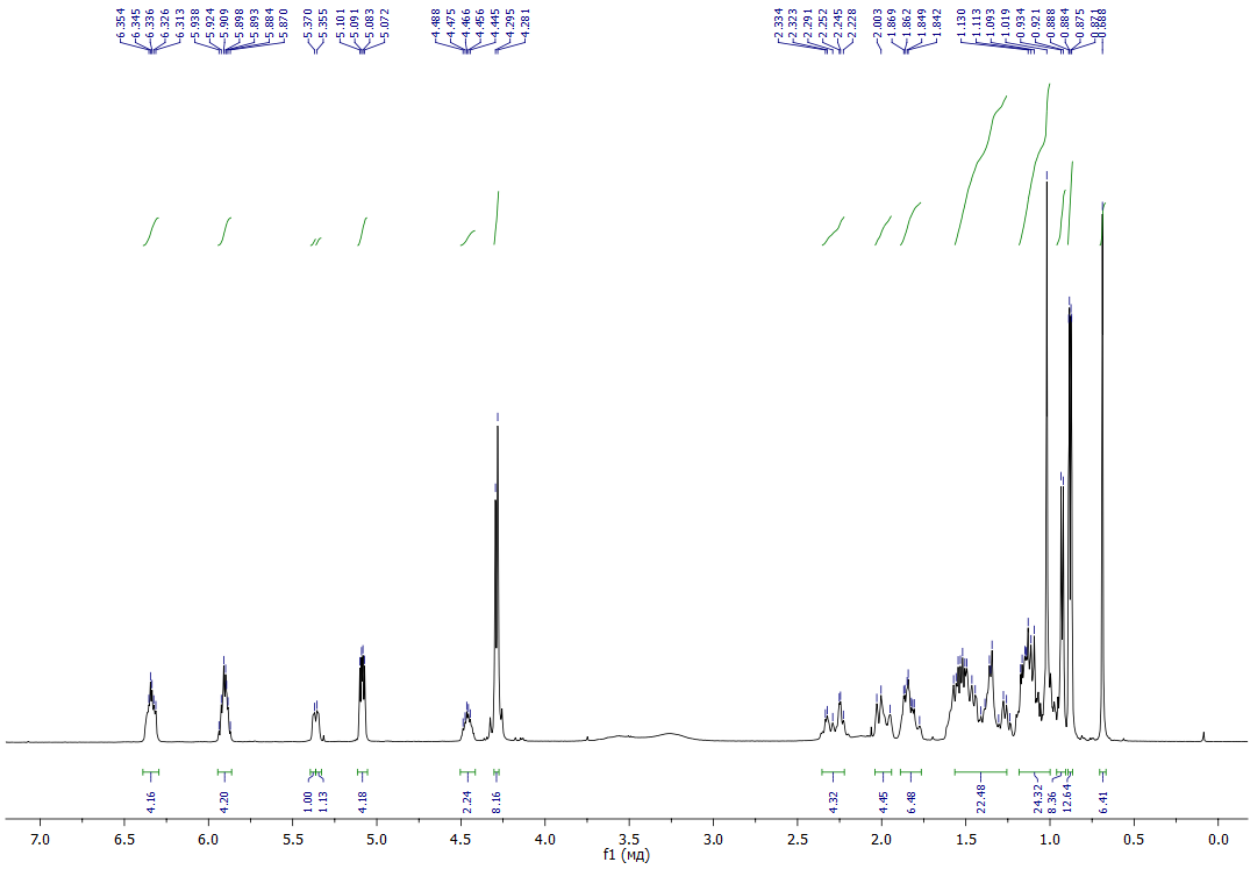

Figure S115. COSY Spectrum of compound **6** (500 MHz, $CDCl_3$)

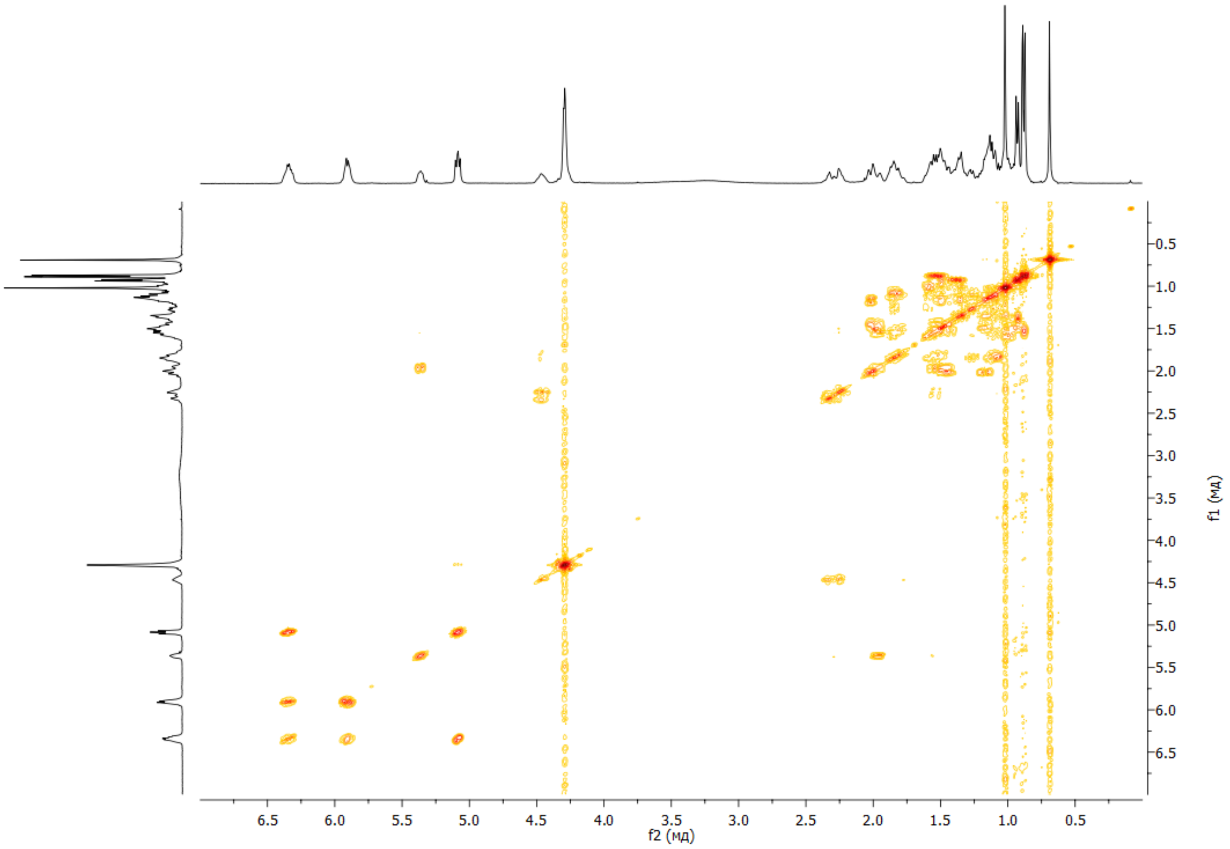

Figure S116. HSQC spectrum of compound **6** (500 MHz, $CDCl_3$)

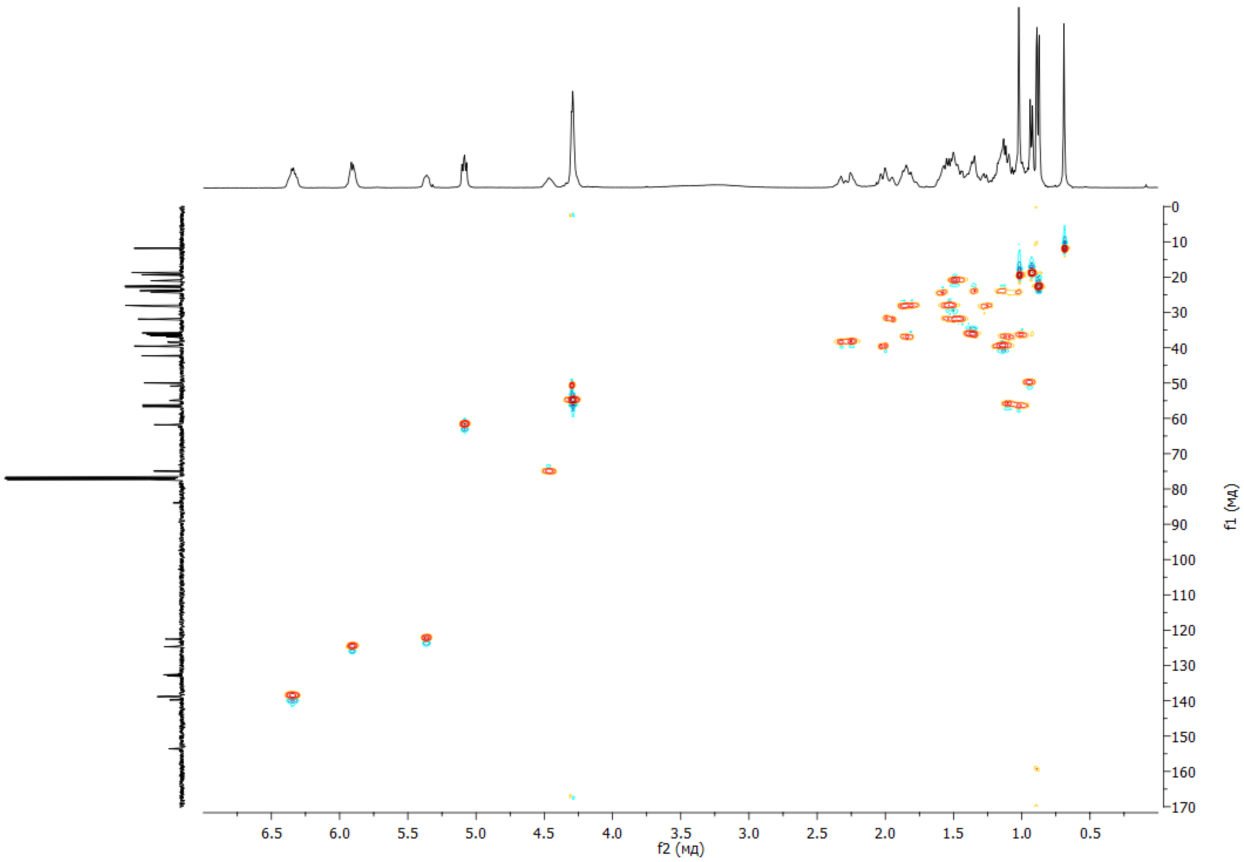

Figure S117. HMBC spectrum of compound **6** (500 MHz, $CDCl_3$)

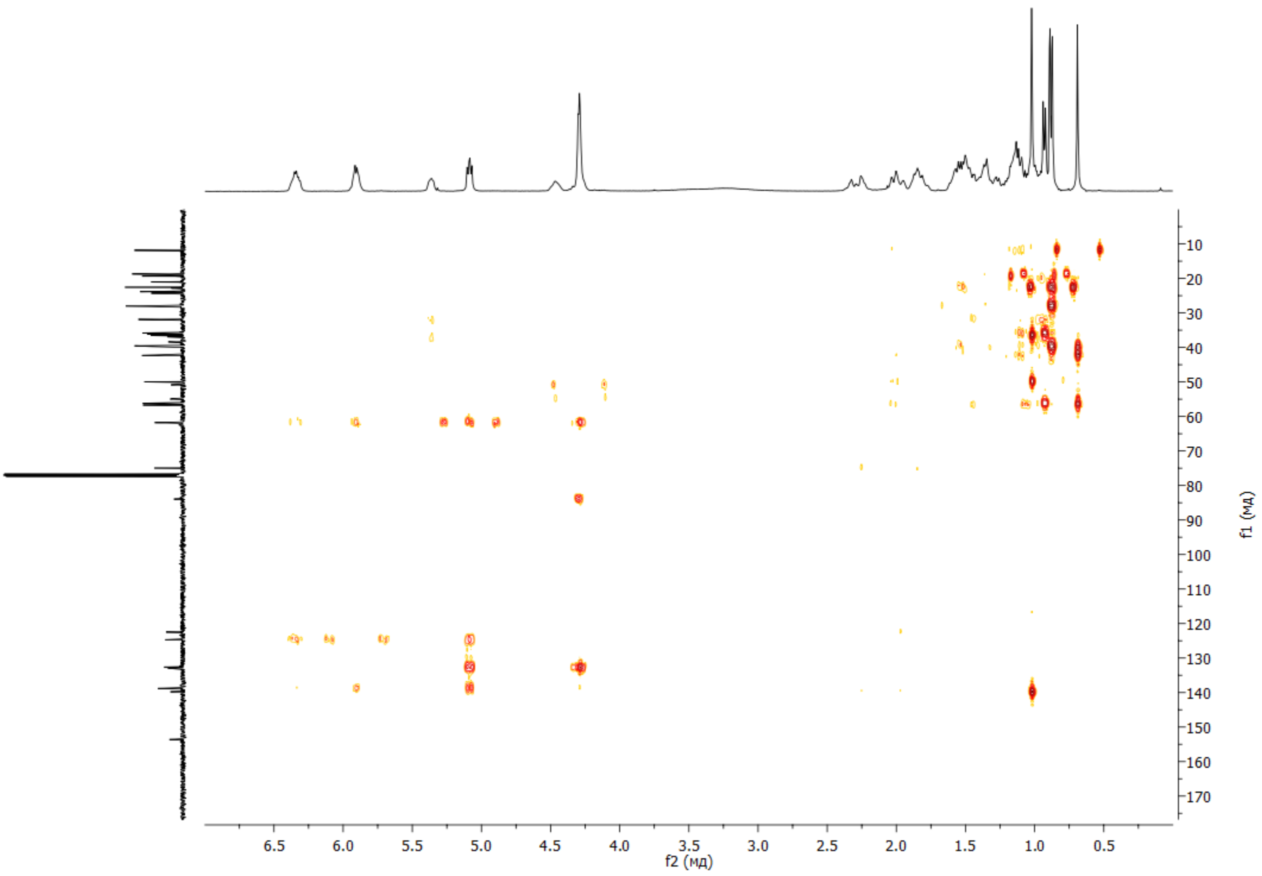

Figure S118. ^{13}C NMR Spectrum of compound **4r** (temperature 298 K, 125 MHz, C_7D_8)

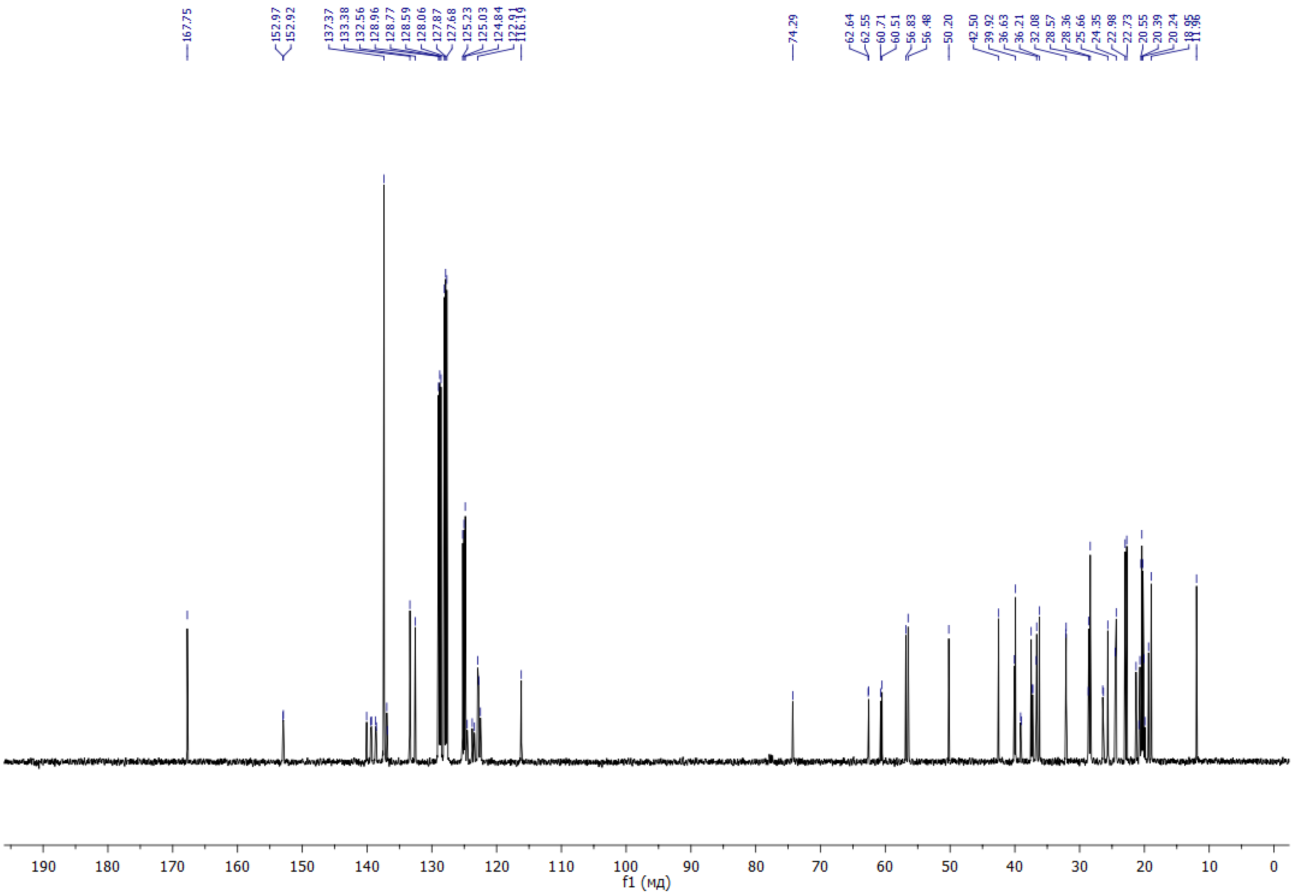

Figure S119. ^{1}H NMR Spectrum of compound **4r** (temperature 298 K, 500 MHz, C_7D_8)

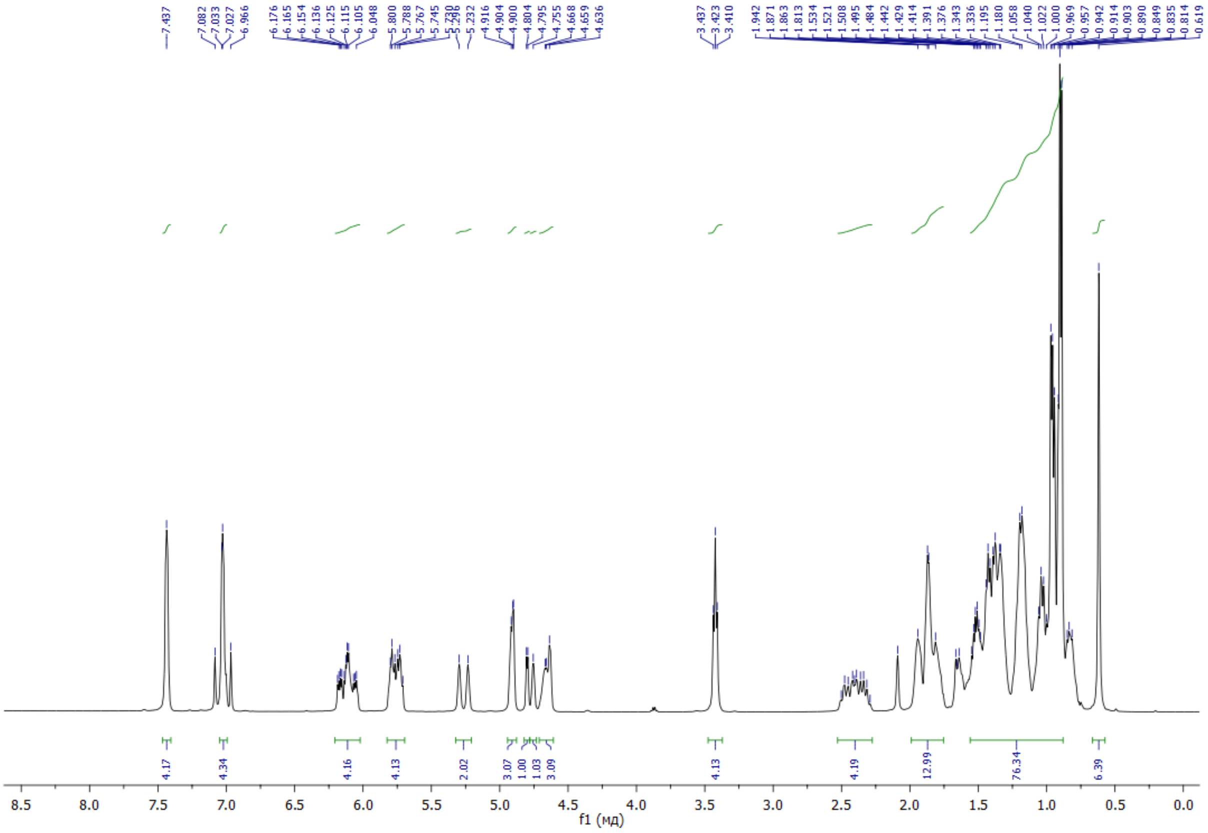

Figure S120. ^{13}C NMR Spectrum of compound **4r (**temperature 333 K, 125 MHz, C_7D_8)

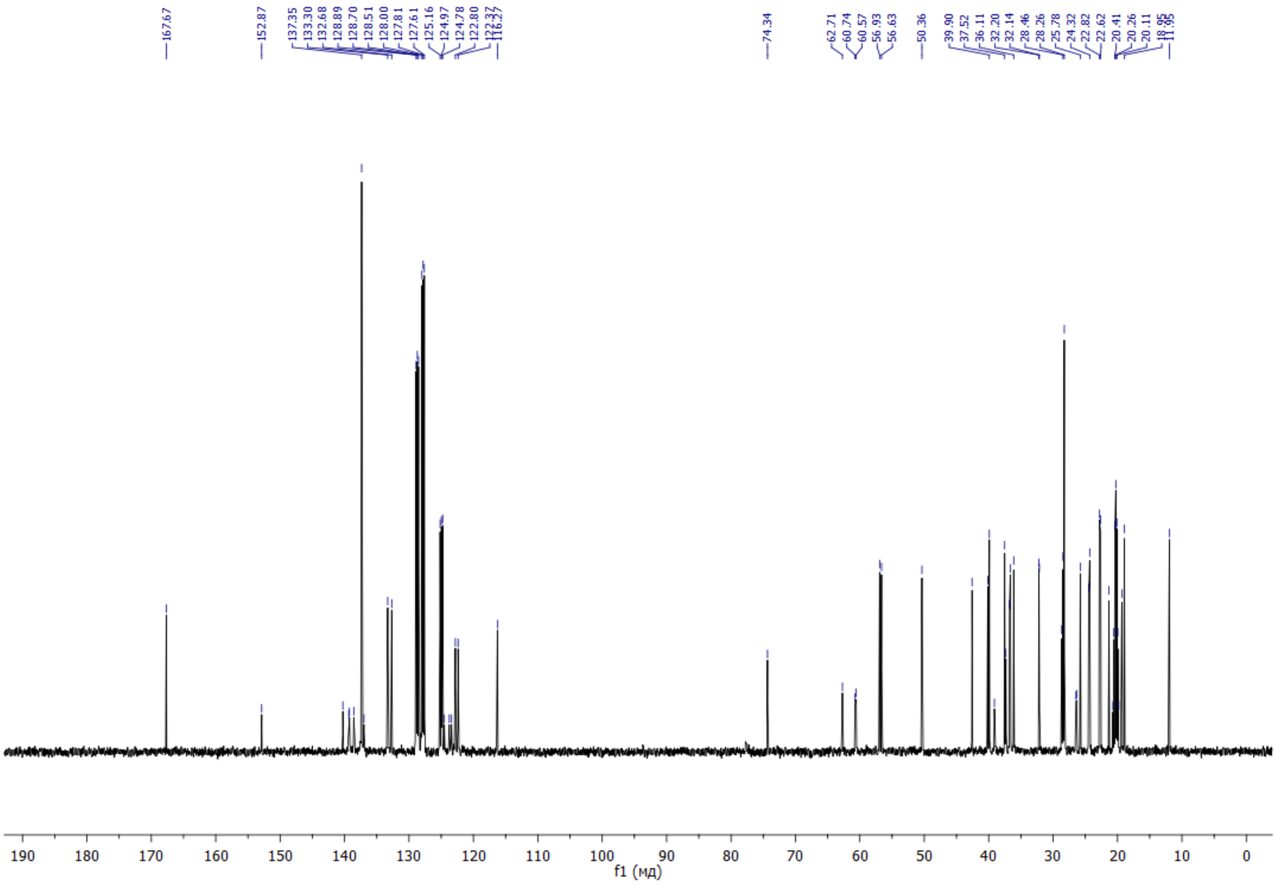

Figure S121. ^{1}H NMR Spectrum of compound **4r** (temperature 333 K, 500 MHz, C_7D_8)

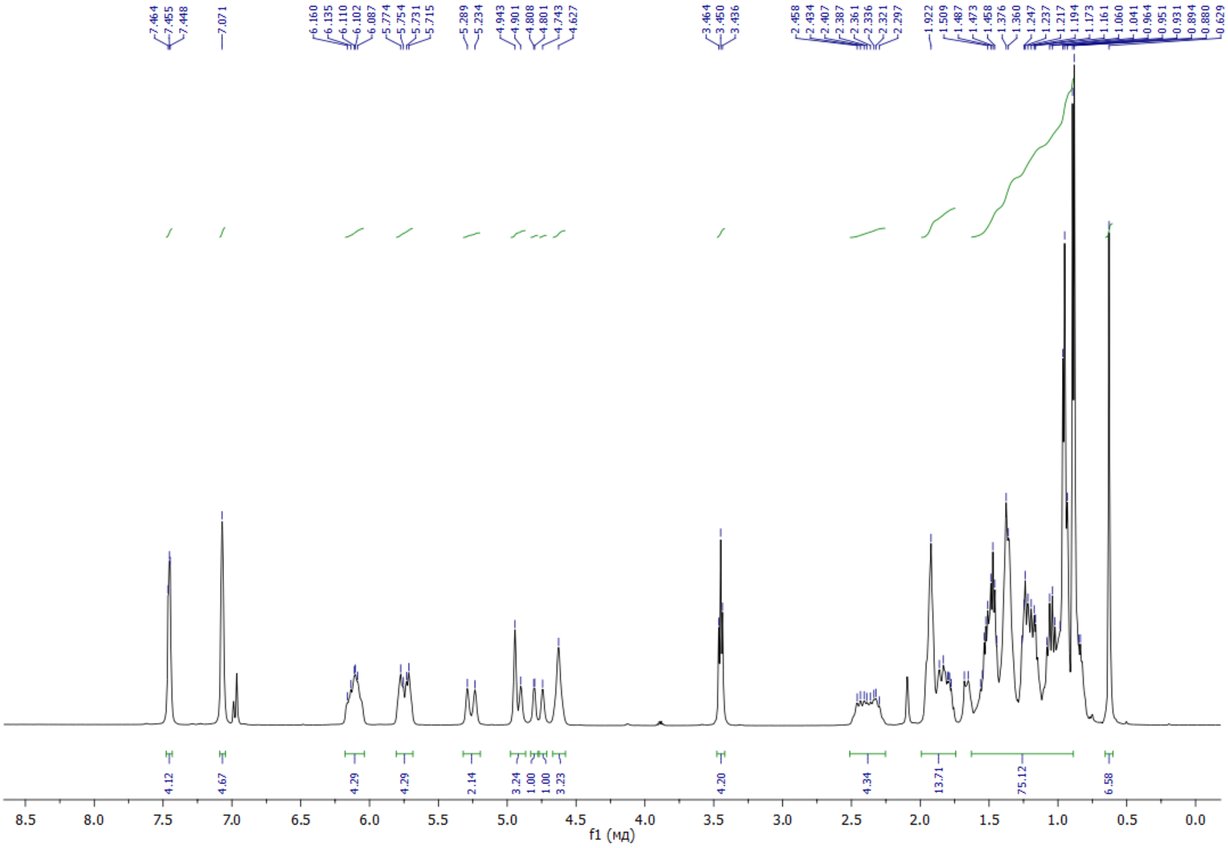

Supplement: Supplementary file 1 [file molecules-26-02932-s001.zip › molecules-1182617-supplentary-final-2.pdf]
